# Supplementary material for: Molecular basis of dimer formation during the biosynthesis of benzofluorene-containing atypical angucyclines
Source: Nat Commun. 2018 May 25;9:2088. doi: 10.1038/s41467-018-04487-z (PMC5970136; doi:10.1038/s41467-018-04487-z)
Supplement: Supplementary file 1 — Supplementary Information [file 41467_2018_4487_MOESM1_ESM.pdf]

Supplementary Information

Molecular Basis of Dimer Formation during the Biosynthesis of Benzofluorene-Containing Atypical Angucyclines

Huang et al.

Supplementary Figures

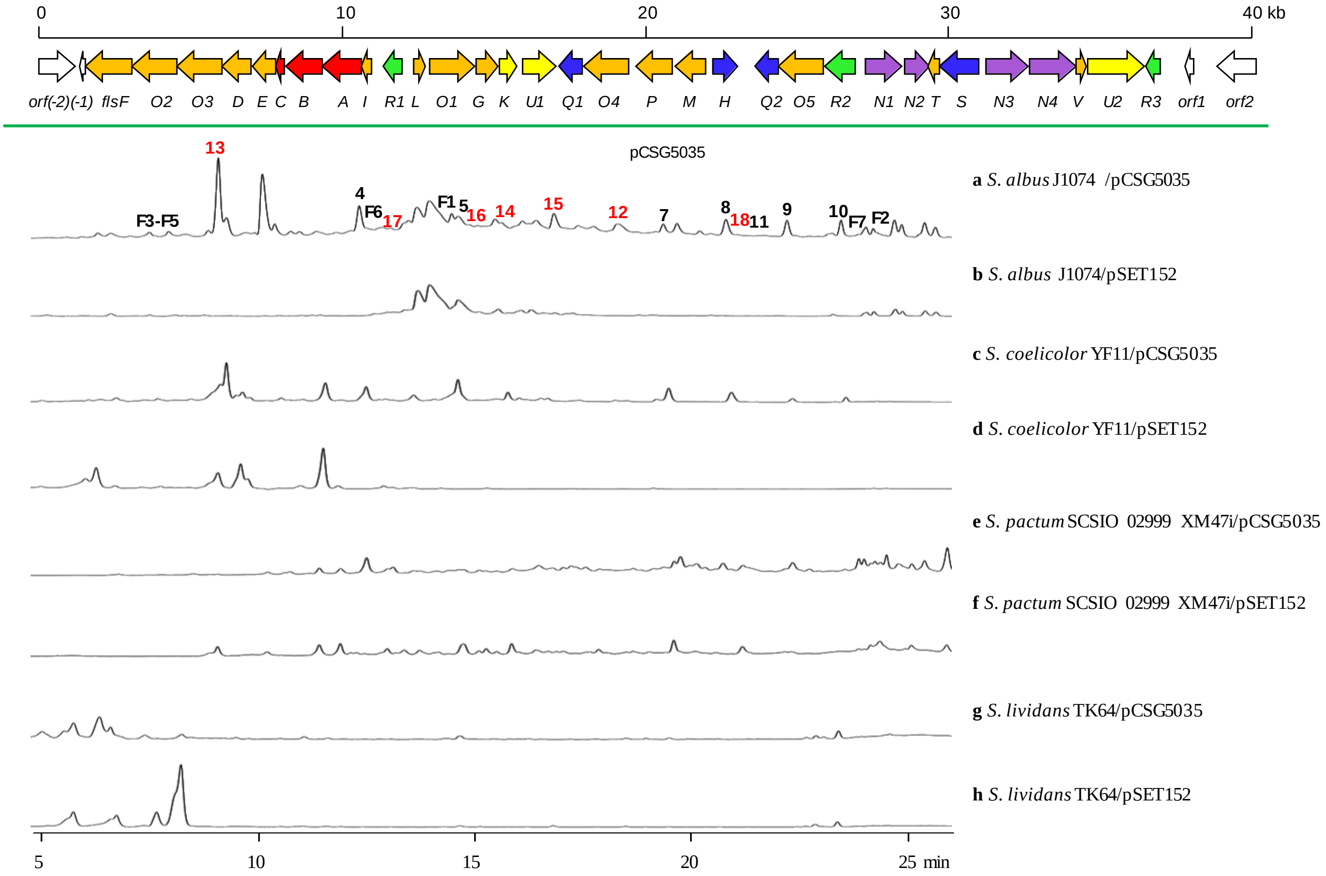

Supplementary Figure 1. HPLC analysis of metabolite profile of expressing *fls*-gene cluster in different hosts in the presence of 3% crude sea salts. a *S. albus* J1074/pCSG5033; **b** *S. albus* J1074/pSET152; **c** *S. coelicolior* YF11/pCSG5033; **d** *S. coelicolior* YF11/pSET152; **e** *S. pactum* SCSIO 02999 XM47i/pCSG5033; **f** *S. pactum* SCSIO 02999 XM47i/pSET152; **g** *S. lividans* TK64/pCSG5033; **h** *S. lividans* TK64/pSET152. Structures for compounds **F1**−**F7**, **4**, **5**, **7**−**18** were shown in Supplementary Figure 2.

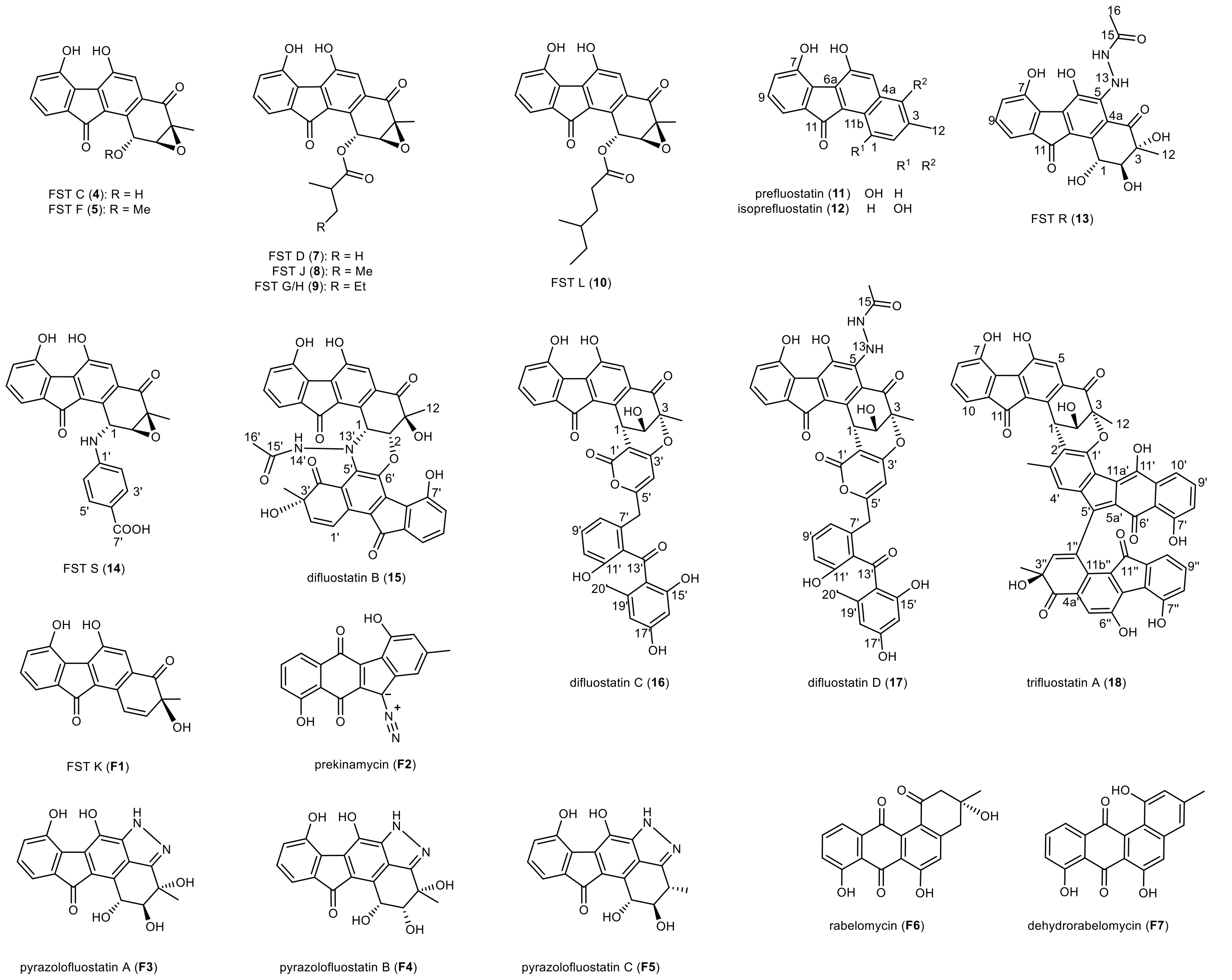

Supplementary Figure 2. Chemical structures for 21 FST-related compounds isolated from *S. albus* J1074/pCSG5033.

a HRESIMS

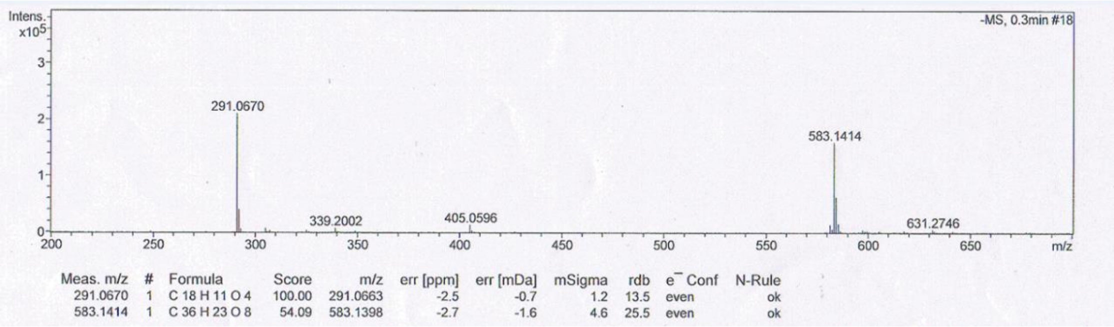

b IR

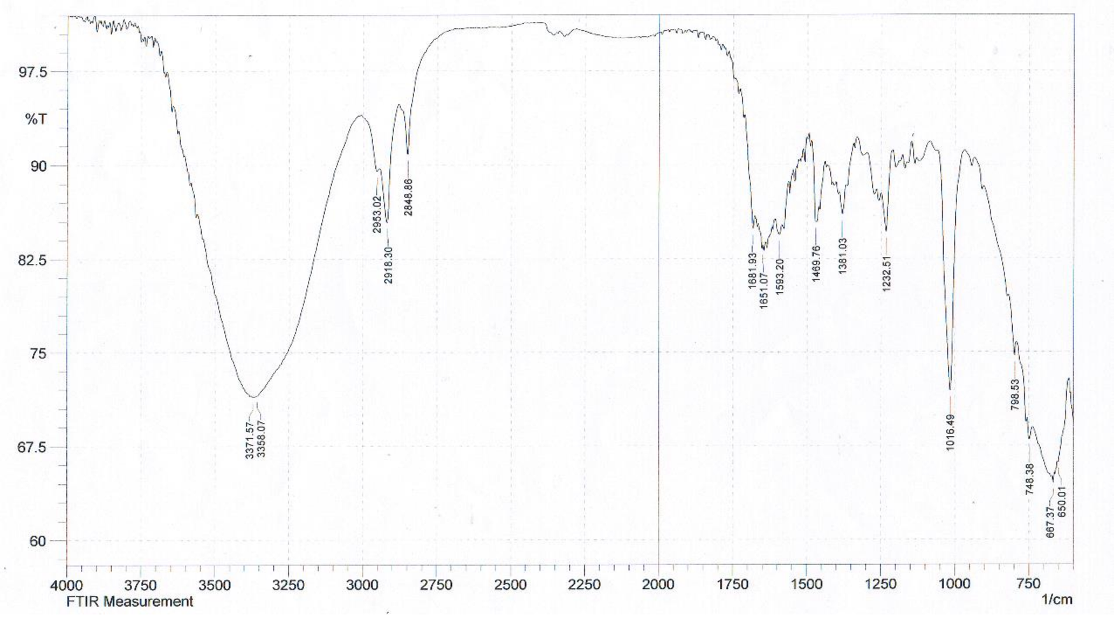

c UV

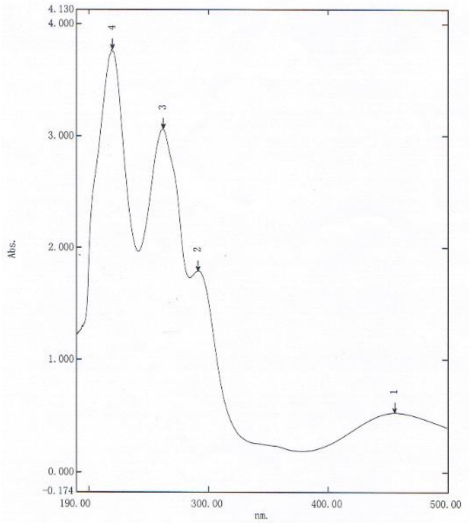

Supplementary Figure 3. Spectroscopic data for isoprefluostatin (12). a HRESIMS, **b** IR, and **c** UV spectra.

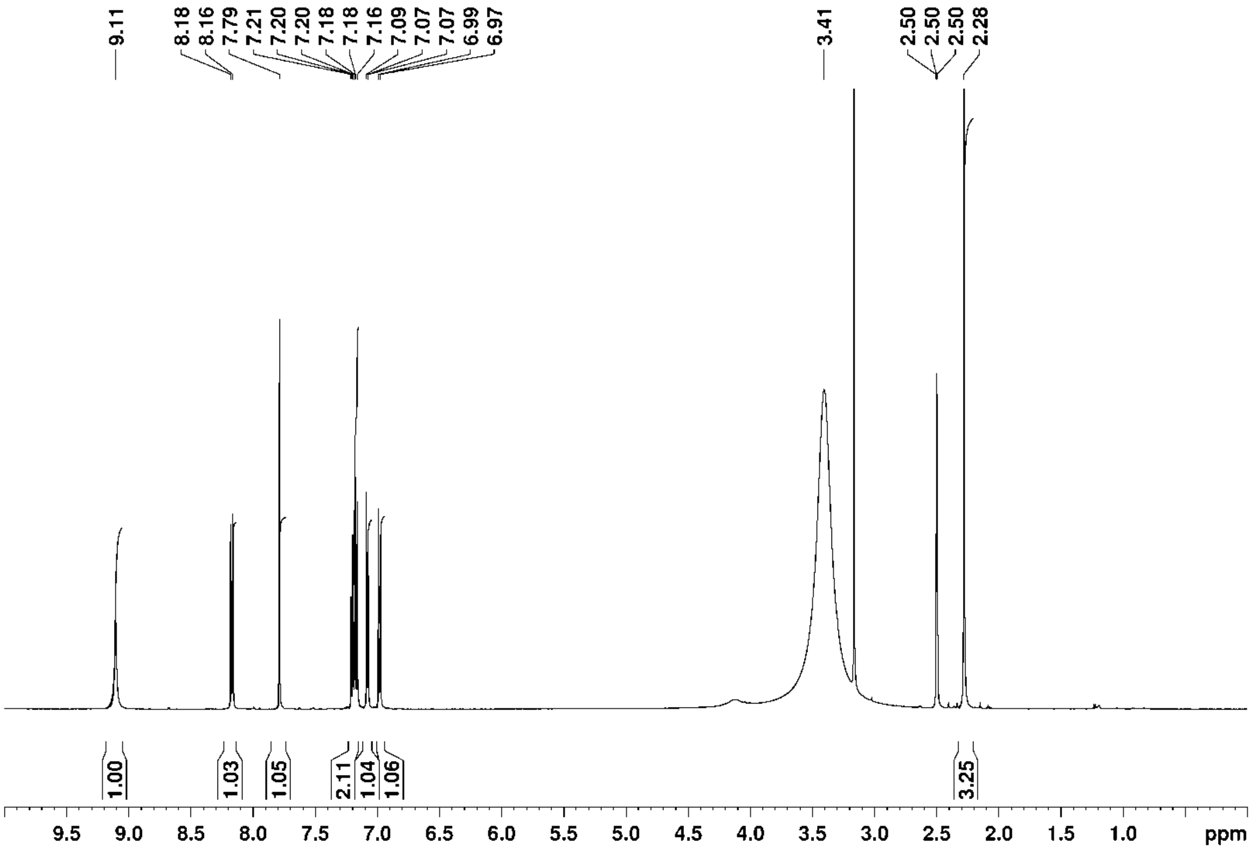

Supplementary Figure 4. The ^{1}H NMR spectrum of isoprefluostatin (**12**) in DMSO-d_6.

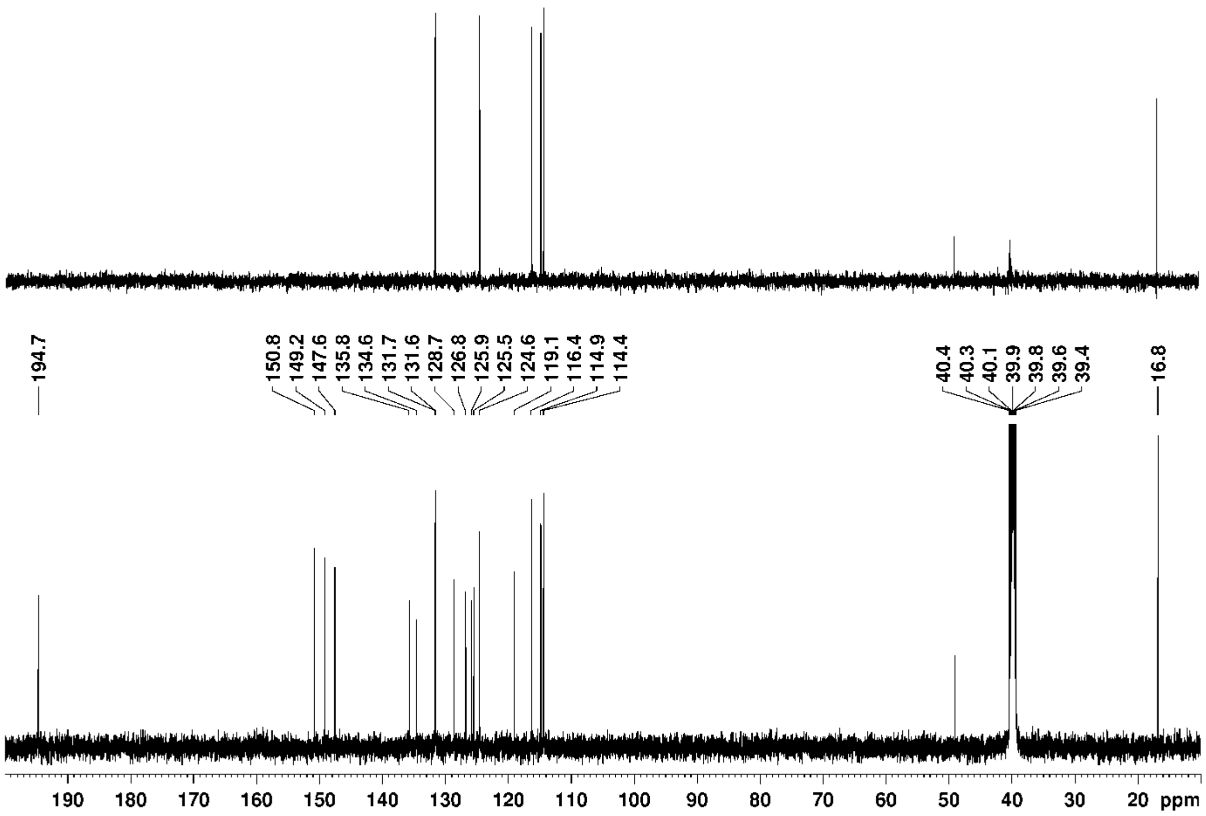

Supplementary Figure 5. The ^{13}C and DEPT 135 NMR spectrum of isoprefluostatin (**12**) in DMSO-d_6.

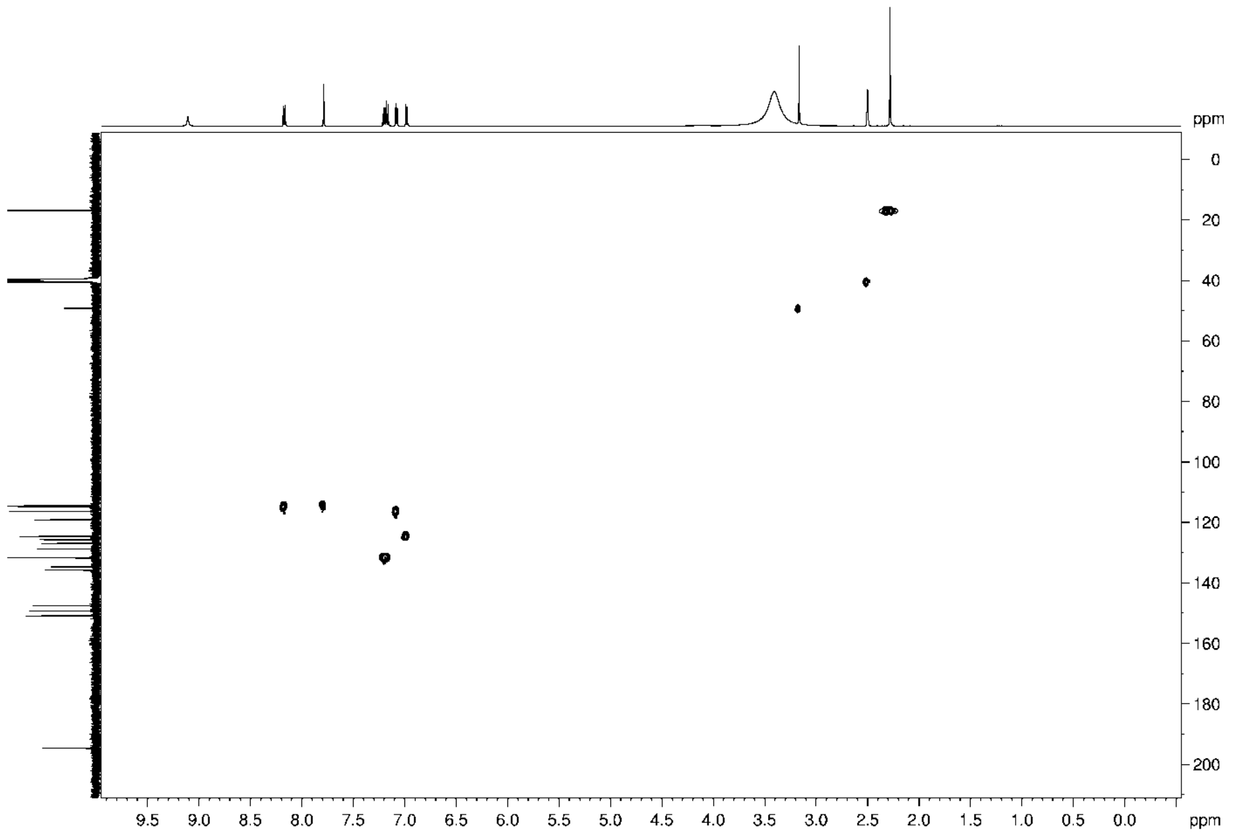

Supplementary Figure 6. The HSQC spectrum of isoprefluostatin (**12**) in DMSO-d_6.

a

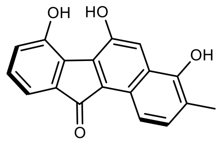

b

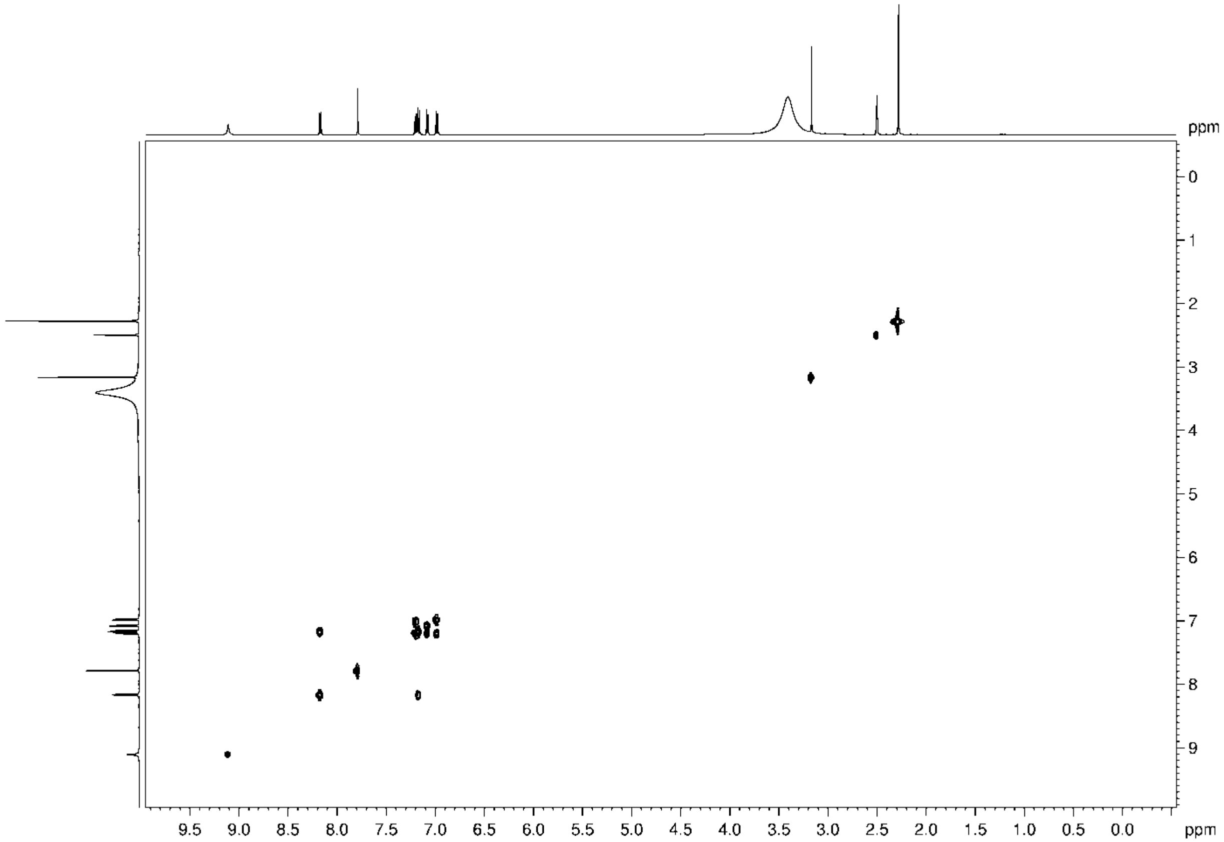

Supplementary Figure 7. The COSY spectrum of isoprefluostatin (**12**) in DMSO-d_6. **a** COSY correlations are indicated by boldface bonds. **b** The COSY spectrum.

a **b**

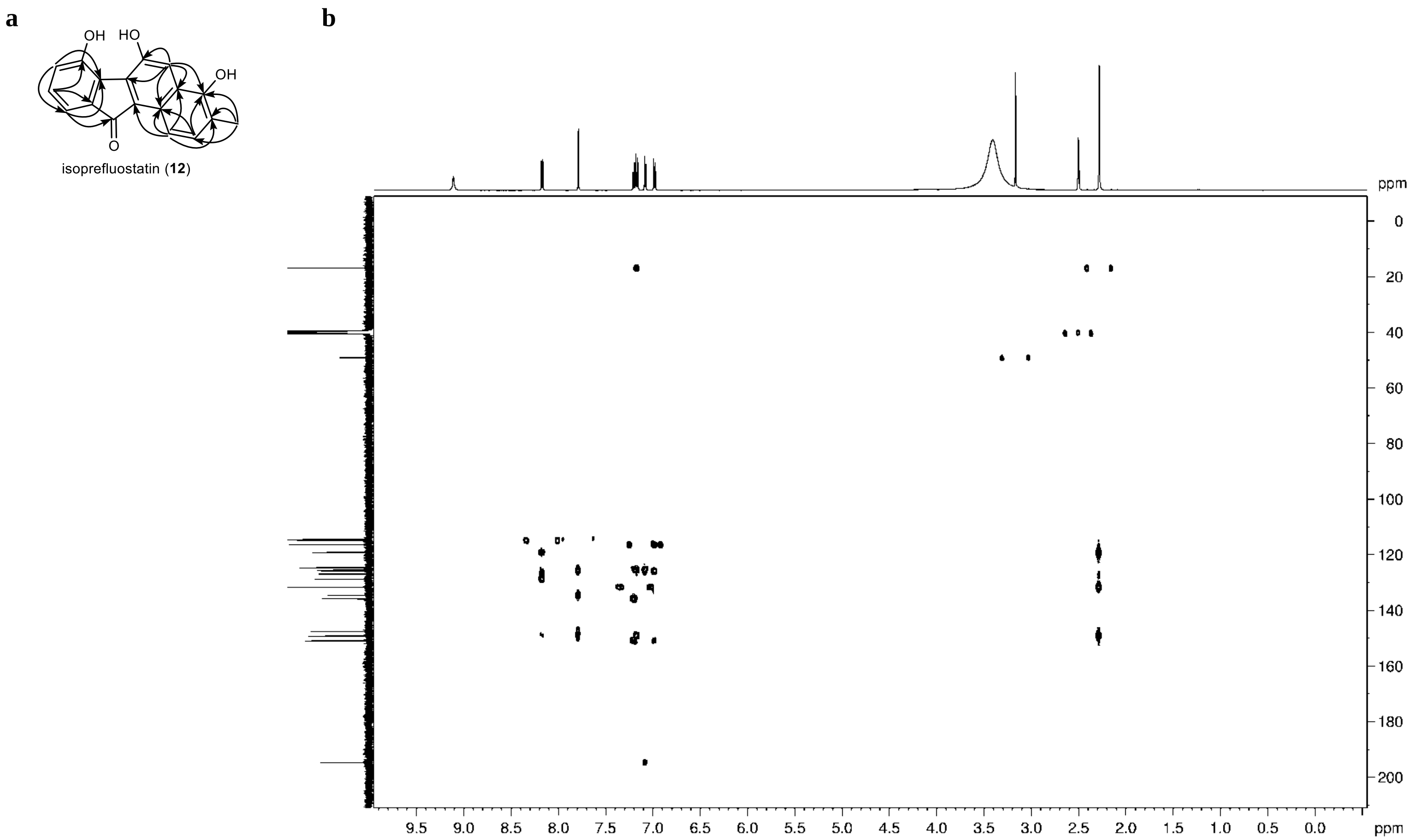

Supplementary Figure 8. The HMBC spectrum of isoprefluostatin (**12**) in DMSO-d_6. **a** Selected key HMBC correlations are indicated by the curved arrows. **b** The HMBC spectrum.

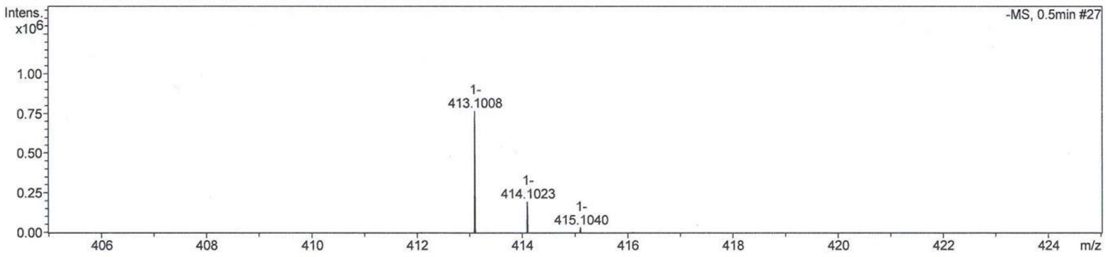

Meas. m/z	#	Ion Formula	Score	m/z	err [ppm]	err [mDa]	mSigma	rdb	e⁻ Conf	N-Rule
413.100793	1	C21H13N6O4	100.00	413.100377	-1.0	-0.4	5.1	18.5	even	ok
	2	C20H17N2O8	37.71	413.099039	-4.2	-1.8	15.3	13.5	even	ok
827.203664	1	C40H35N4O16	100.00	827.205355	2.0	1.7	11.0	25.5	even	ok
	2	C42H27N12O8	7.04	827.208029	5.3	4.4	12.9	35.5	even	ok

Supplementary Figure 9. HRESIMS spectrum of FST R (**13**).

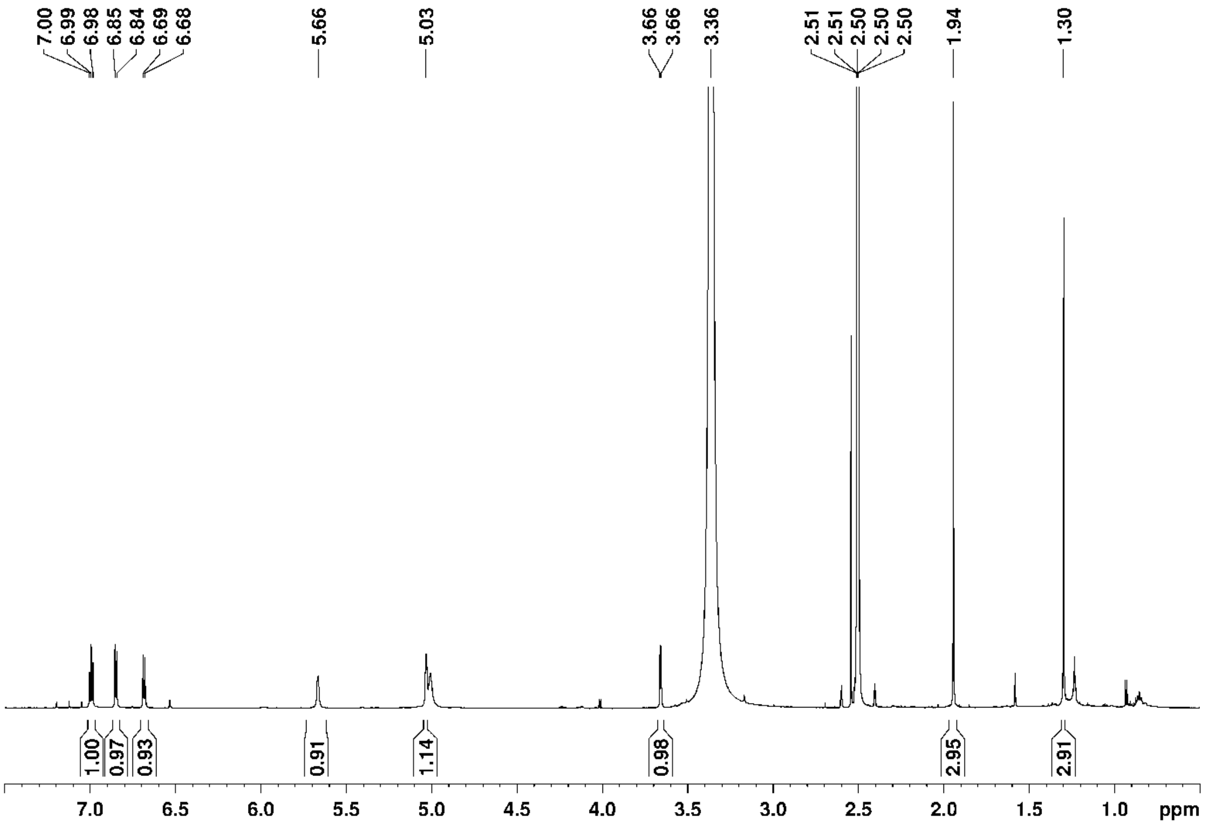

Supplementary Figure 10. The ^{1}H NMR spectrum of FST R (**13**) in DMSO-d_6

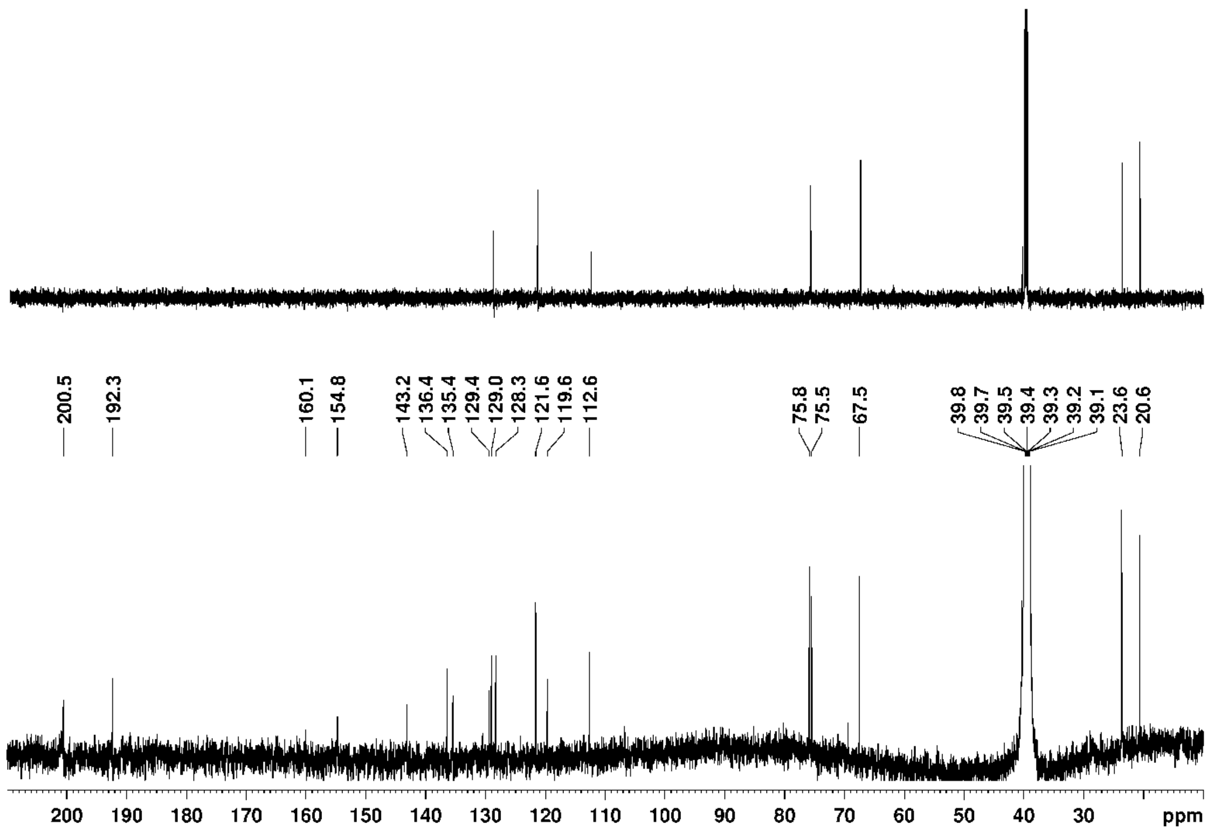

Supplementary Figure 11. The ^{13}C and DEPT 135 NMR spectrum of FST R (**13**) in DMSO-d_6.

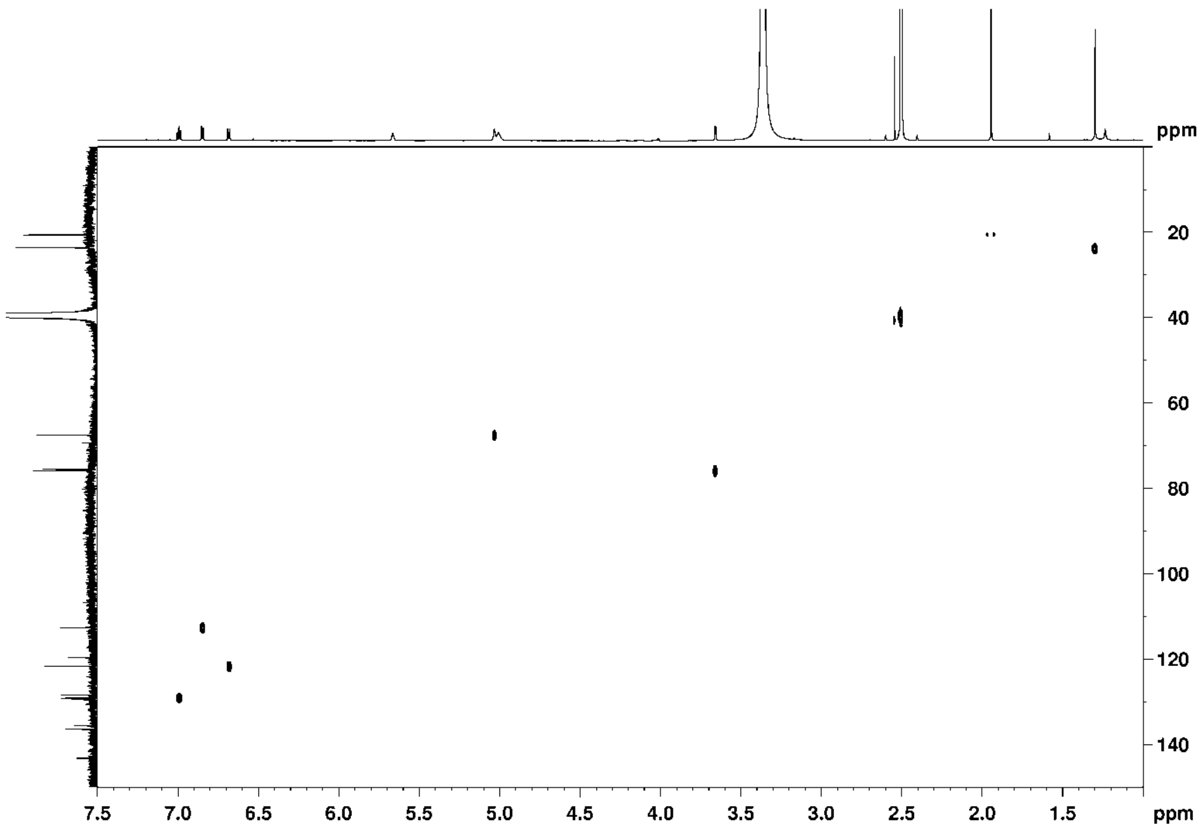

Supplementary Figure 12. The HSQC spectrum of FST R (**13**) in DMSO-d_6.

a

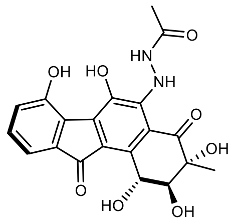

FST R (**13**)

b

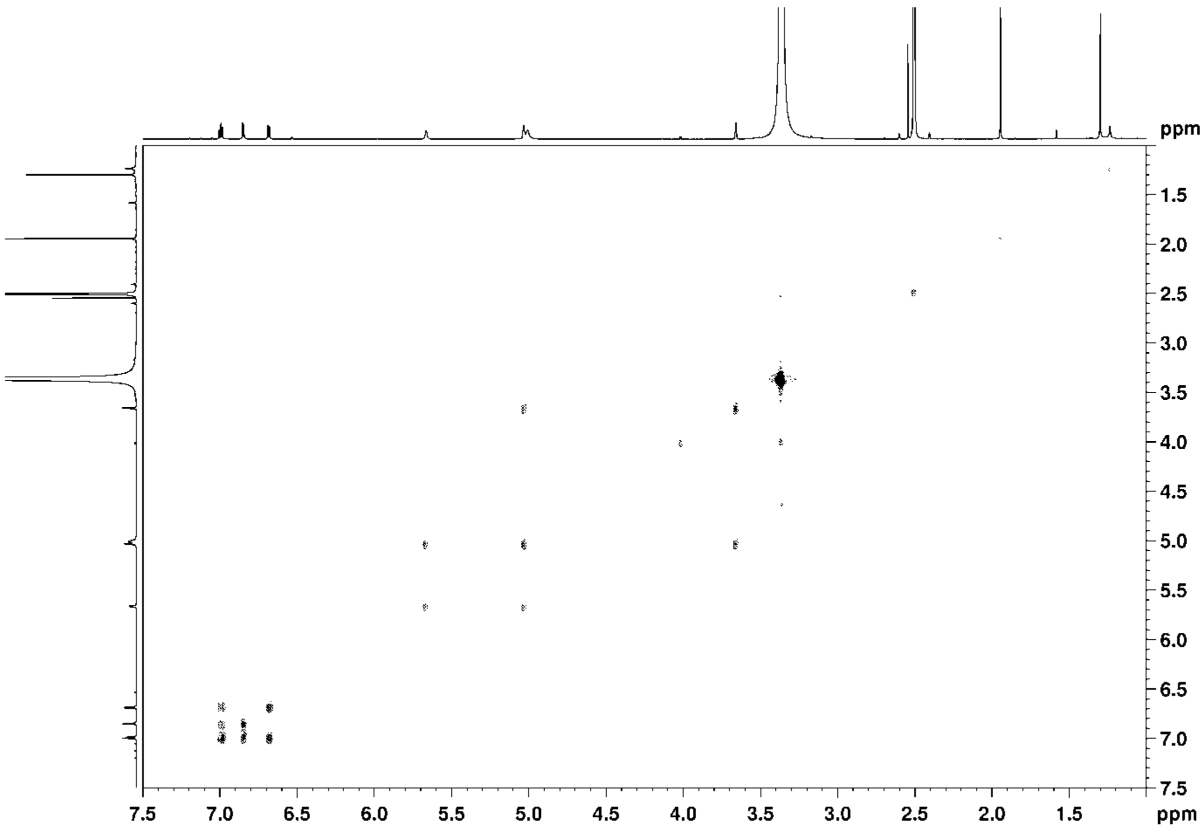

Supplementary Figure 13. The COSY spectrum of FST R (**13**) in DMSO-d_6. **a** COSY correlations are indicated by boldface bonds. **b** The COSY spectrum.

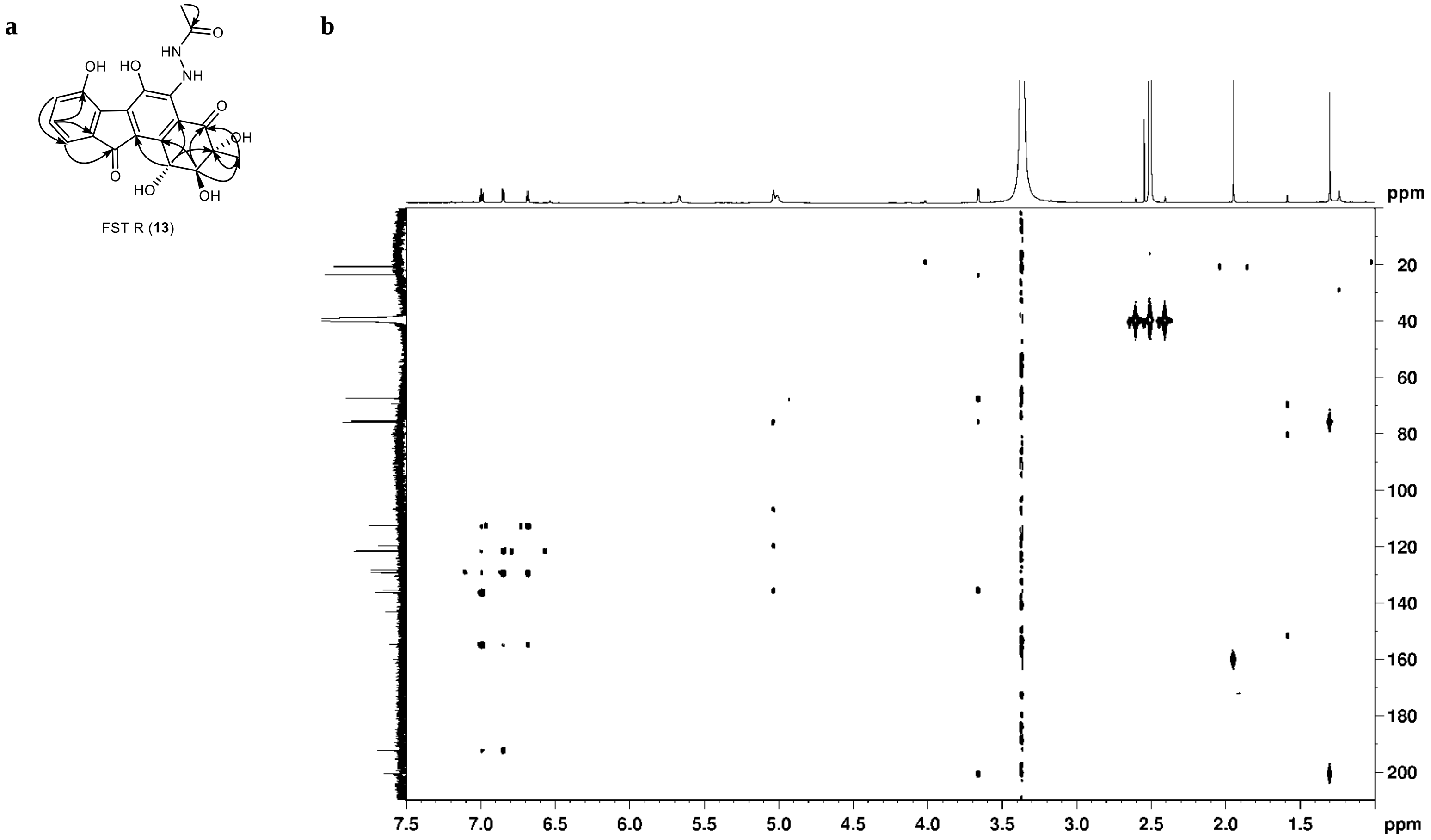

Supplementary Figure 14. The HMBC spectrum of FST R (**13**) in DMSO-d_6. **a** Selected key HMBC correlations are indicated by the curved arrows. **b** The HMBC spectrum.

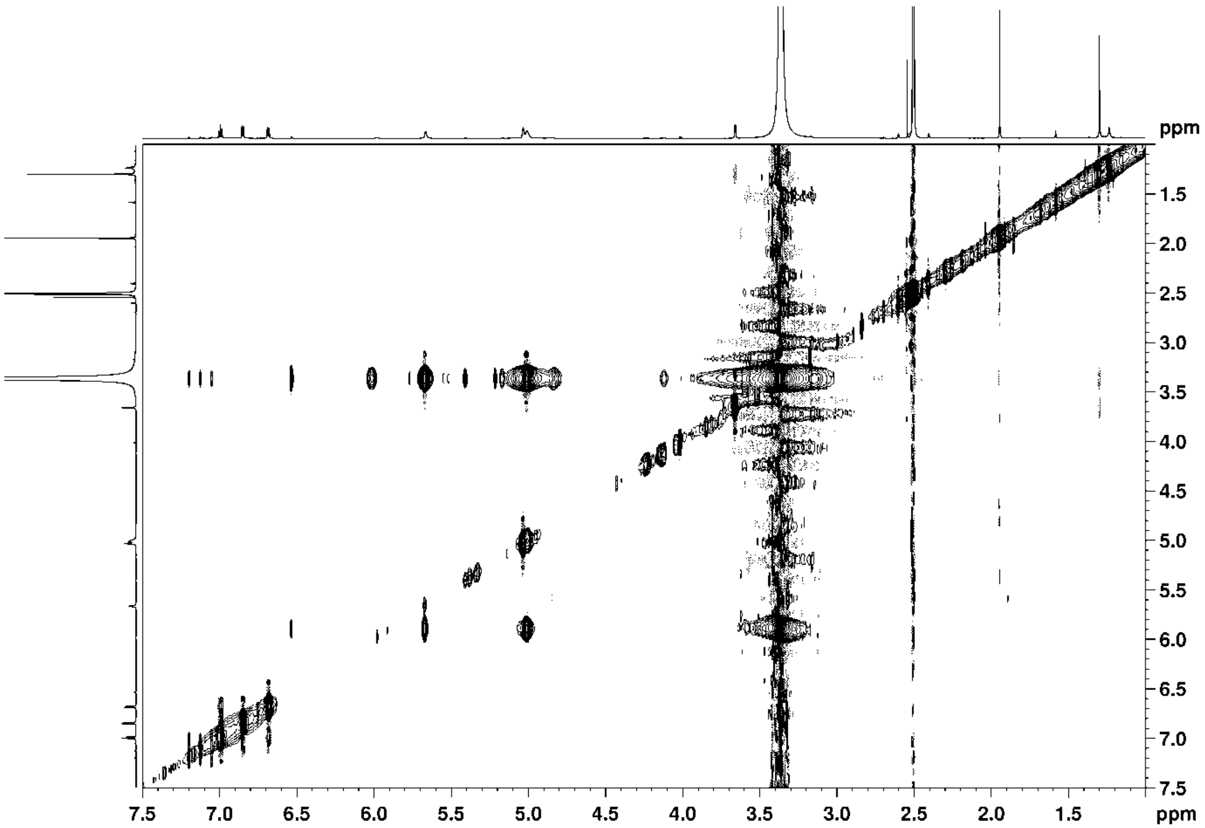

Supplementary Figure 15. The NOESY spectrum of FST R (**13**) in DMSO-d_6.

a HRESIMS

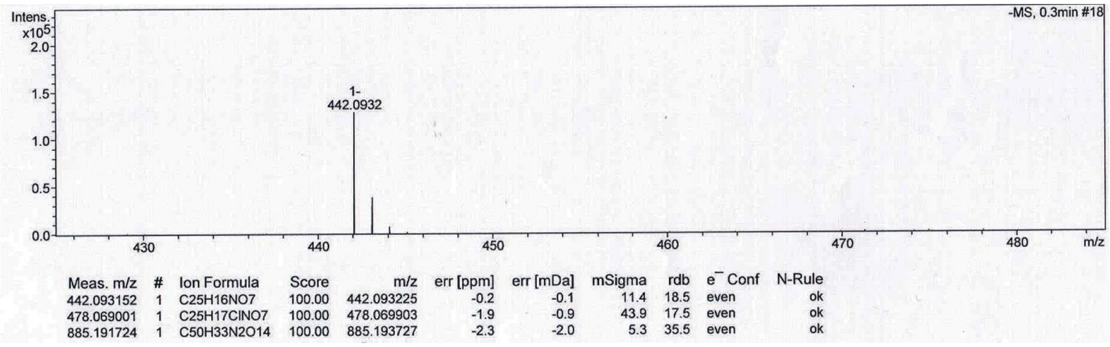

Meas. m/z	#	Ion Formula	Score	m/z	err [ppm]	err [mDa]	mSigma	rdb	e⁻ Conf	N-Rule
442.093152	1	C25H16NO7	100.00	442.093225	-0.2	-0.1	11.4	18.5	even	ok
478.069001	1	C25H17ClNO7	100.00	478.069903	-1.9	-0.9	43.9	17.5	even	ok
885.191724	1	C50H33N2O14	100.00	885.193727	-2.3	-2.0	5.3	35.5	even	ok

b IR

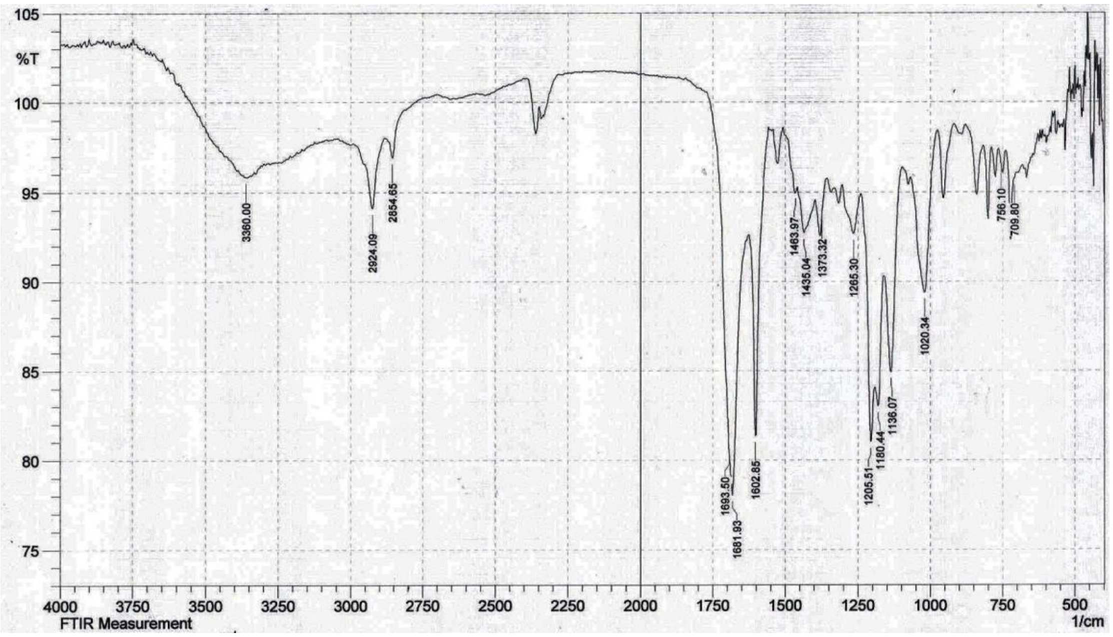

c UV

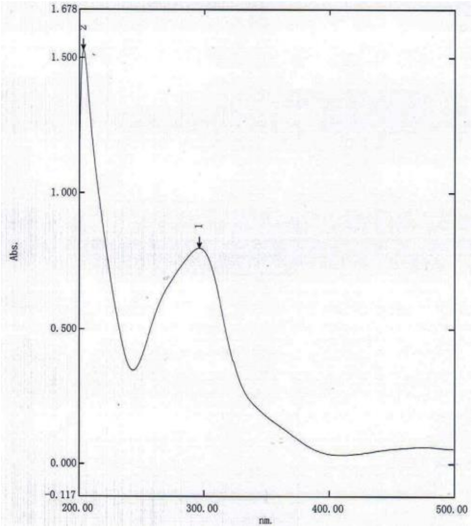

Supplementary Figure 16. Spectroscopic data for FST S (14). a HRESIMS, **b** IR, and **c** UV spectra of FST S (**14**).

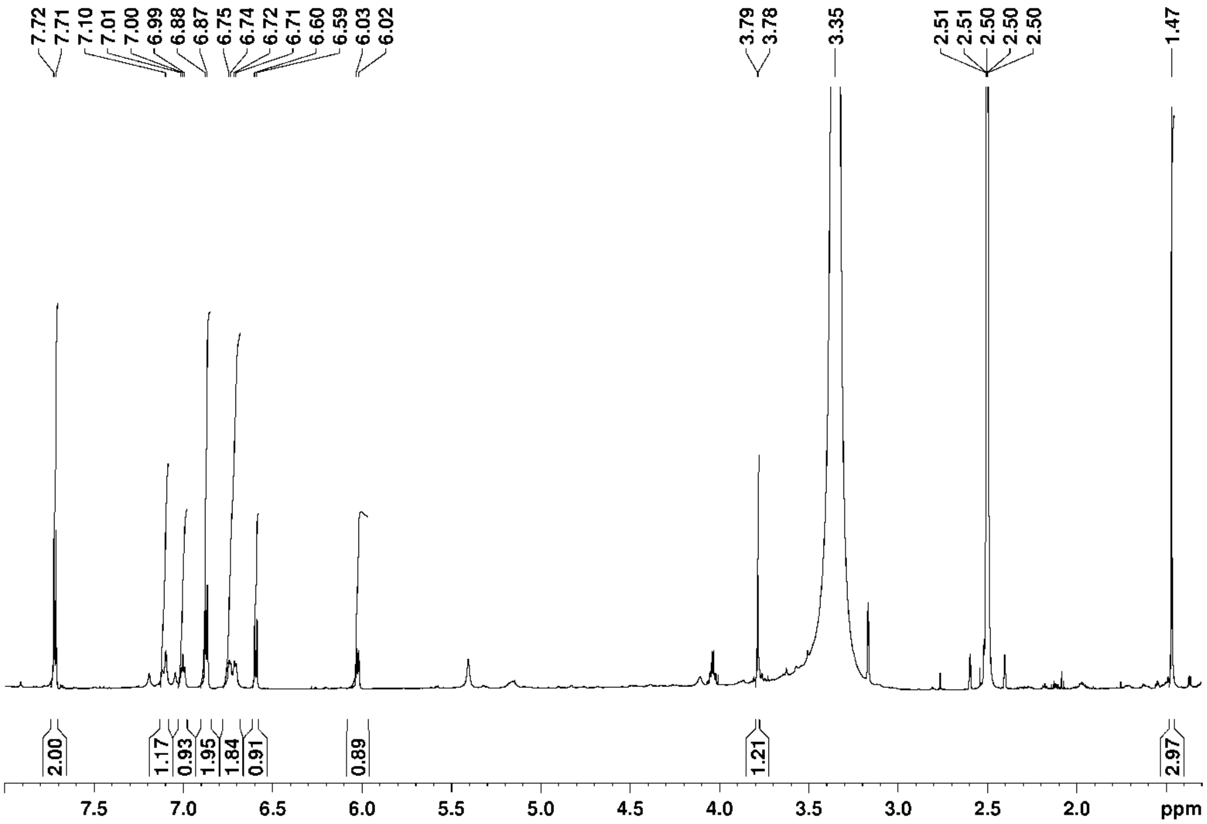

Supplementary Figure 17. The ^{1}H NMR spectrum of FST S (**14**) in DMSO-d_6.

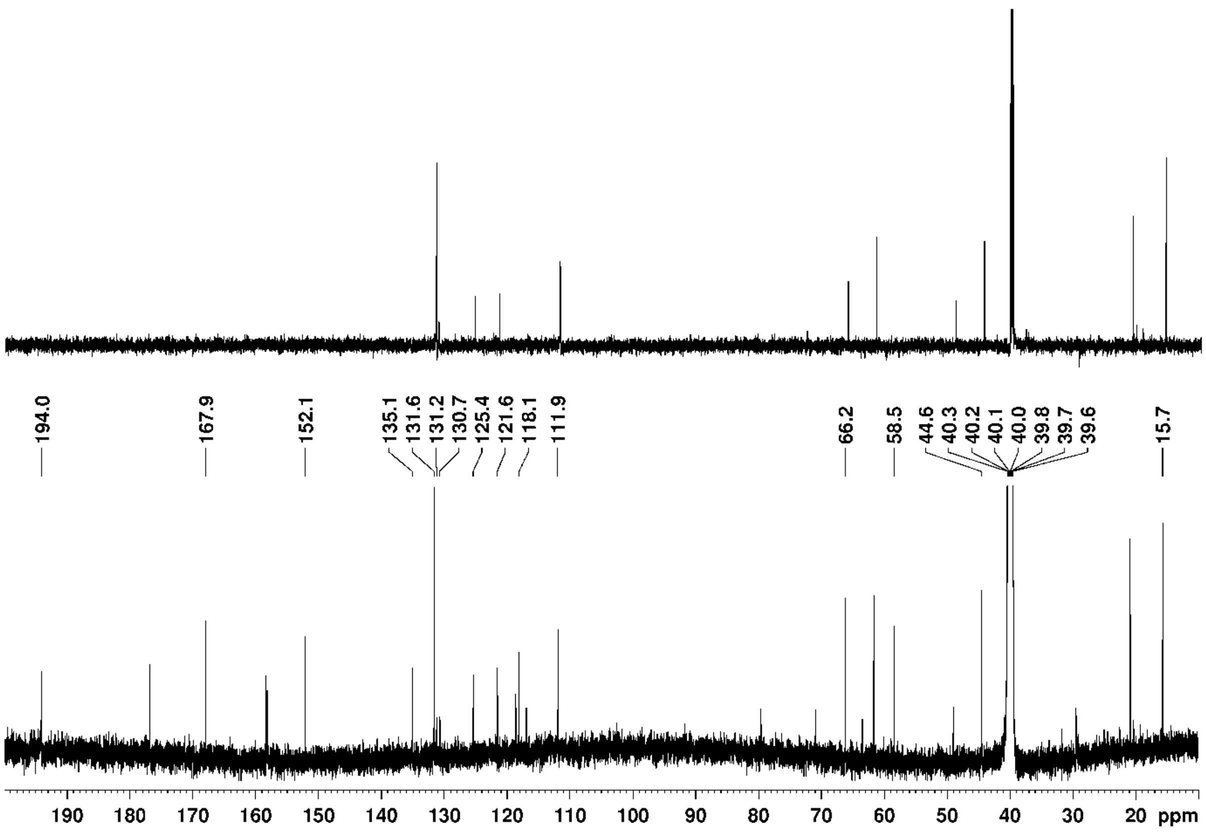

Supplementary Figure 18. The ^{13}C and DEPT 135 NMR spectrum of FST S (**14**) in DMSO-d_6.

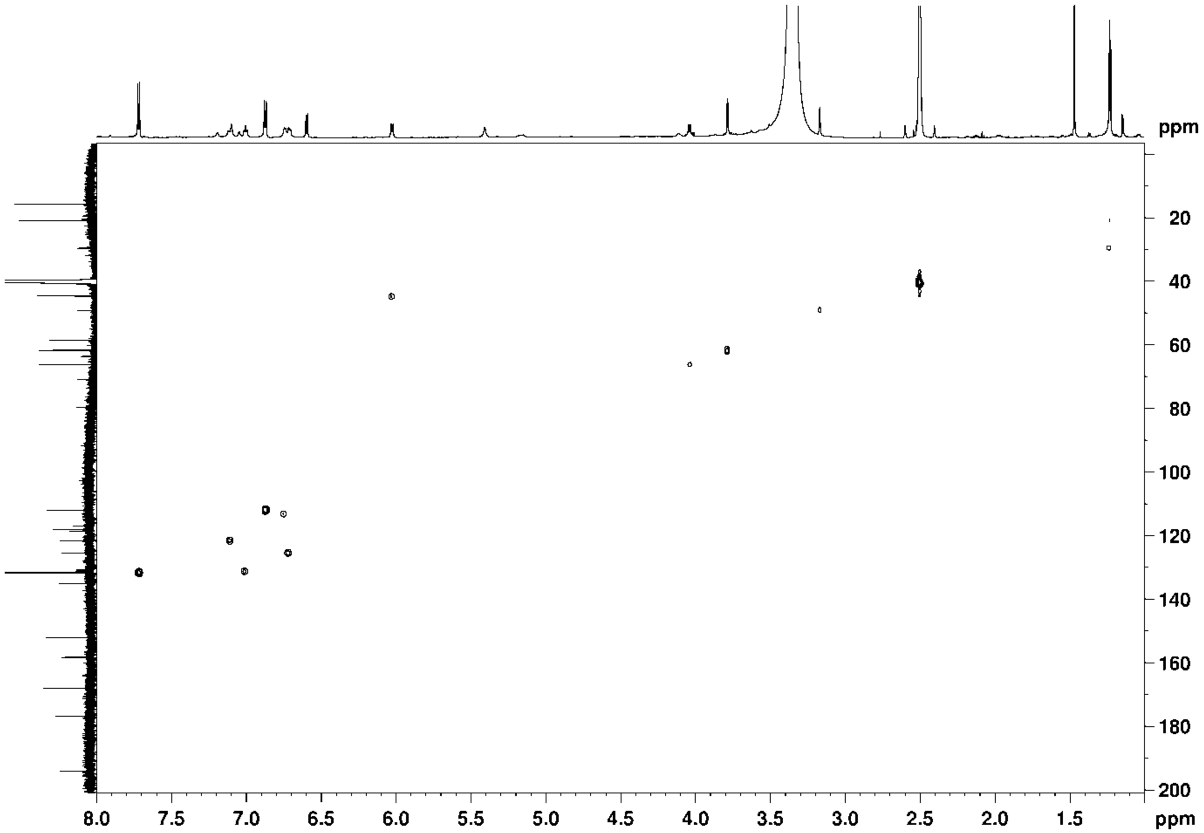

Supplementary Figure 19. The HSQC spectrum of FST S (**14**) in DMSO-d_6.

a

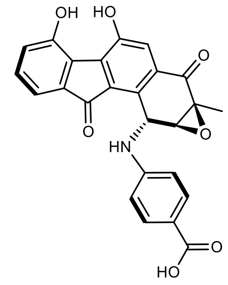

FST S (**14**)

b

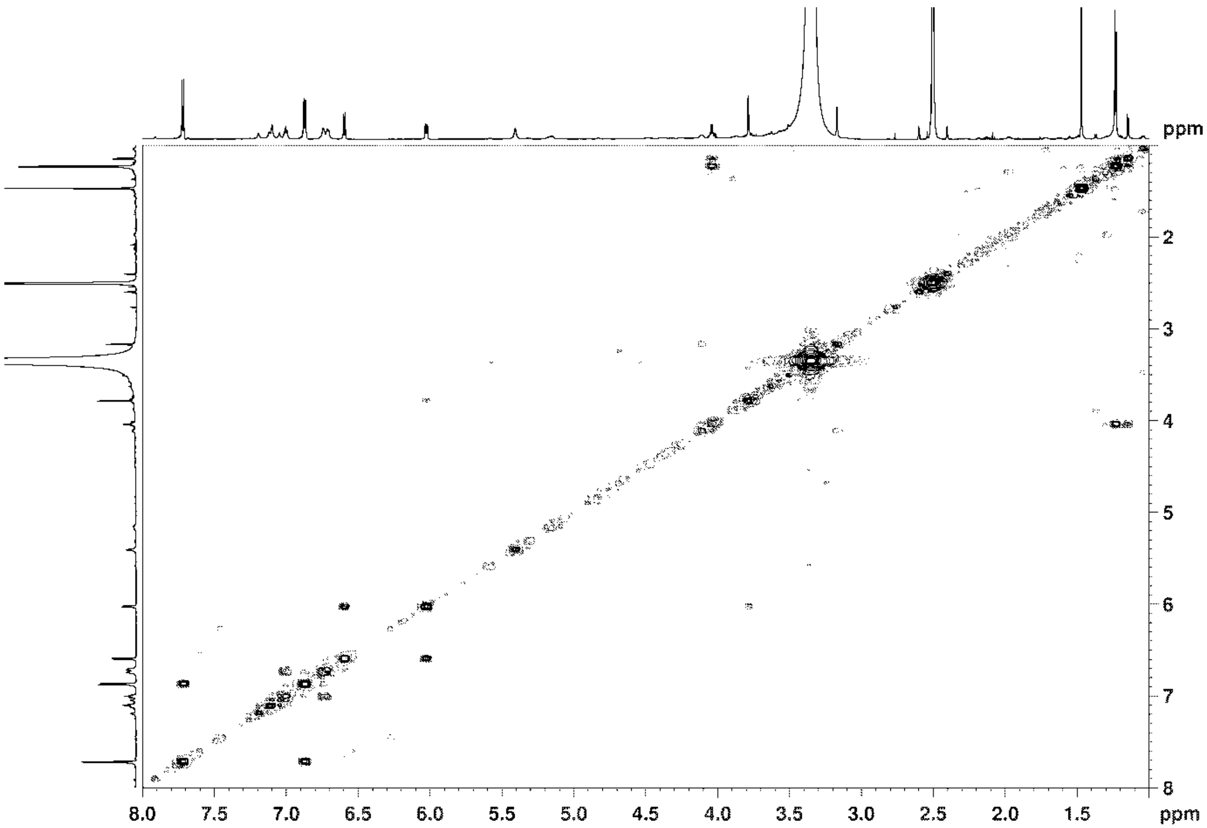

Supplementary Figure 20. The COSY spectrum of FST S (**14**) in DMSO-d_6. **a** COSY correlations are indicated by boldface bonds. **b** The COSY spectrum.

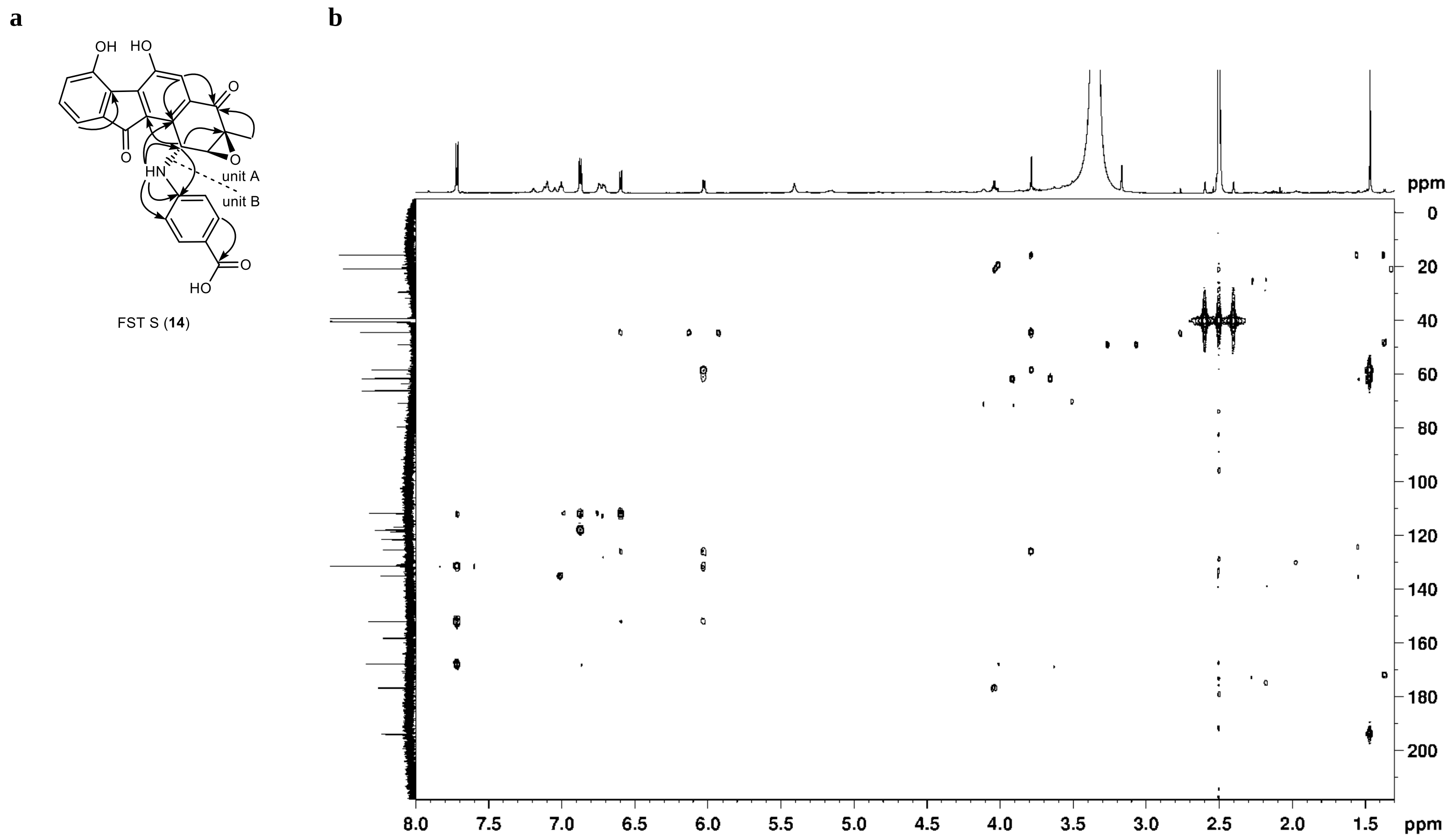

Supplementary Figure 21. The HMBC spectrum of FST S (**14**) in DMSO-d_6. **a** Selected key HMBC correlations are indicated by the curved arrows. **b** The HMBC spectrum.

a **b**

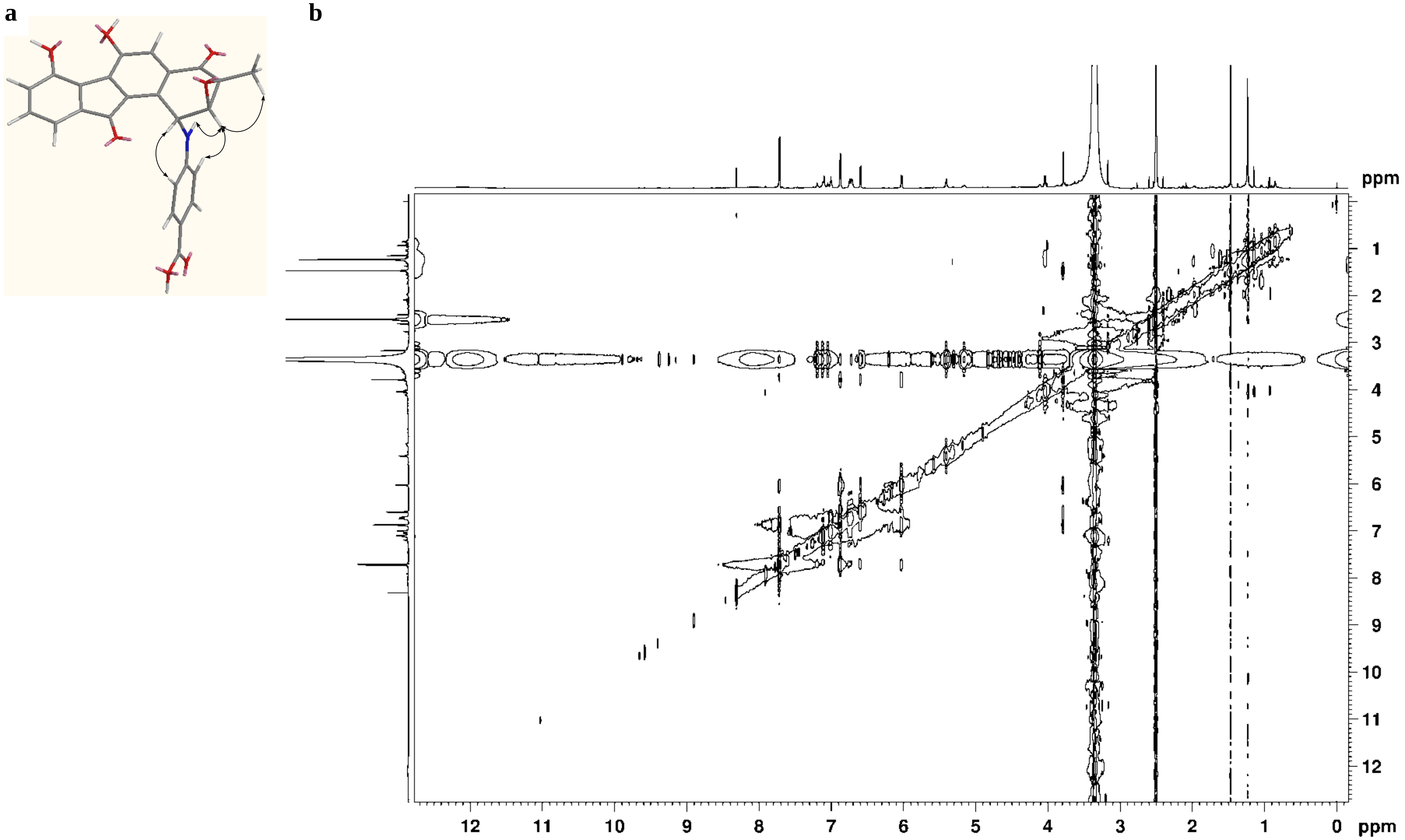

Supplementary Figure 22. The NOESY spectrum of FST S (**14**) in DMSO-d_6. **a** Selected key NOESY correlations are indicated by the curved, double-headed arrows. **b** The NOESY spectrum.

a HRESIMS

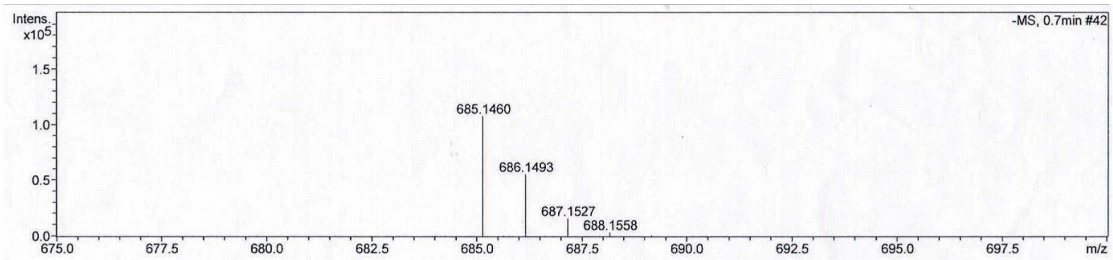

Meas. m/z	#	Formula	Score	m/z	err [ppm]	err [mDa]	mSigma	rdb	e⁻ Conf	N-Rule
685.1460	1	C 40 H 17 N 10 O 3	21.02	685.1491	4.5	3.1	29.5	37.5	even	ok
	2	C 39 H 21 N 6 O 7	48.39	685.1477	2.5	1.7	41.0	32.5	even	ok
	3	C 36 H 13 N 16 O	100.00	685.1464	0.6	0.4	43.2	38.5	even	ok
	4	C 38 H 25 N 2 O 11	79.47	685.1464	0.6	0.4	50.8	27.5	even	ok
	5	C 35 H 17 N 12 O 5	54.92	685.1450	-1.4	-0.9	53.7	33.5	even	ok
	6	C 34 H 21 N 8 O 9	14.76	685.1437	-3.3	-2.3	65.4	28.5	even	ok

b IR

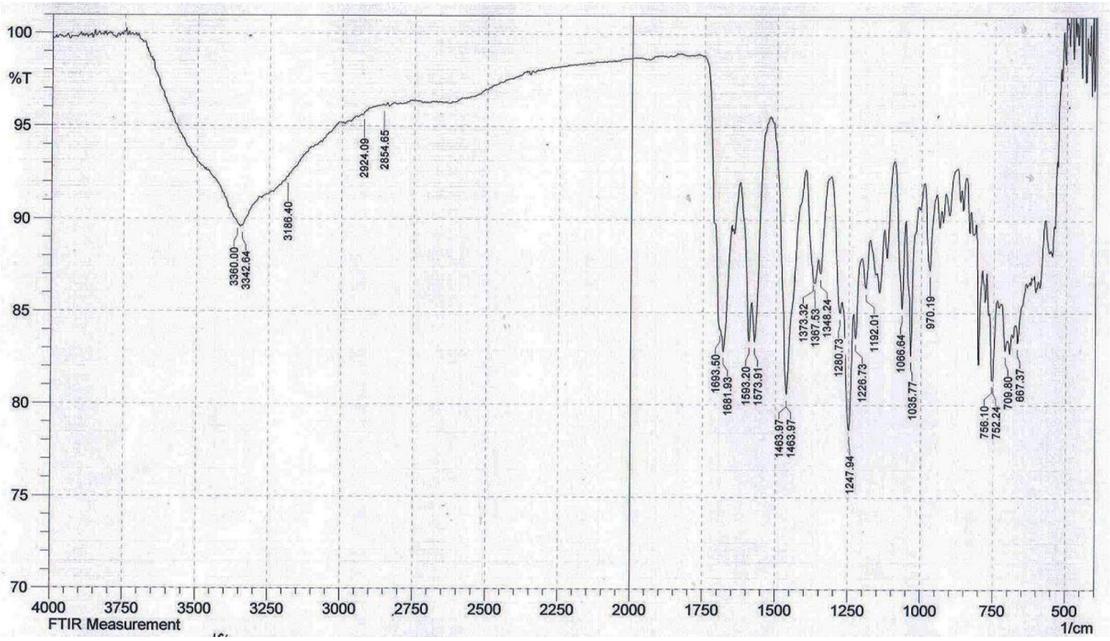

c UV

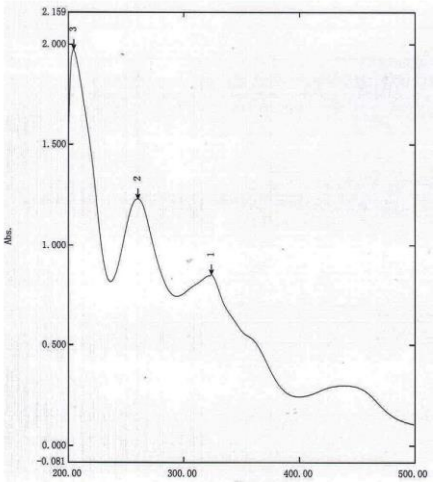

Supplementary Figure 23. Spectroscopic data for difluostatin B (15). a HRESIMS, **b** IR, and **c** UV spectra of difluostatin B (**15**).

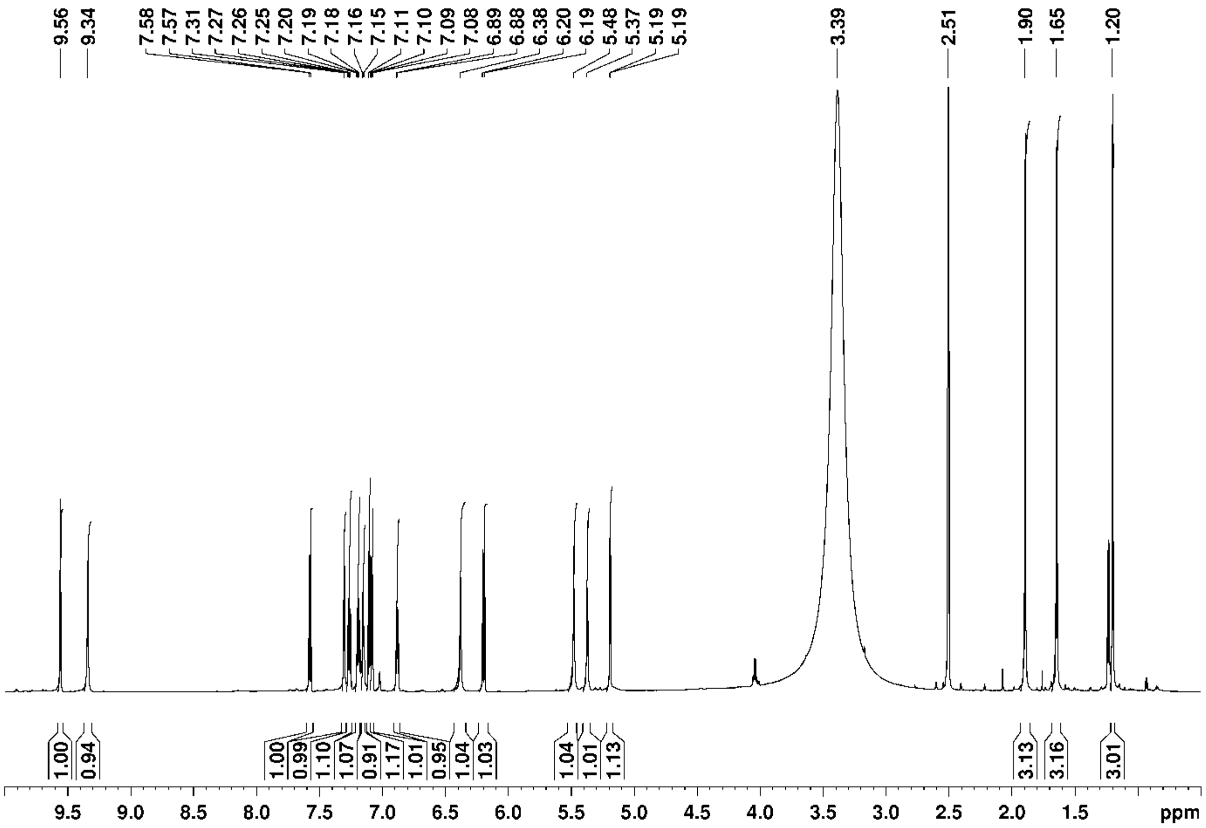

Supplementary Figure 24. The ^{1}H NMR spectrum of difluostatin B (**15**) in DMSO-d_6.

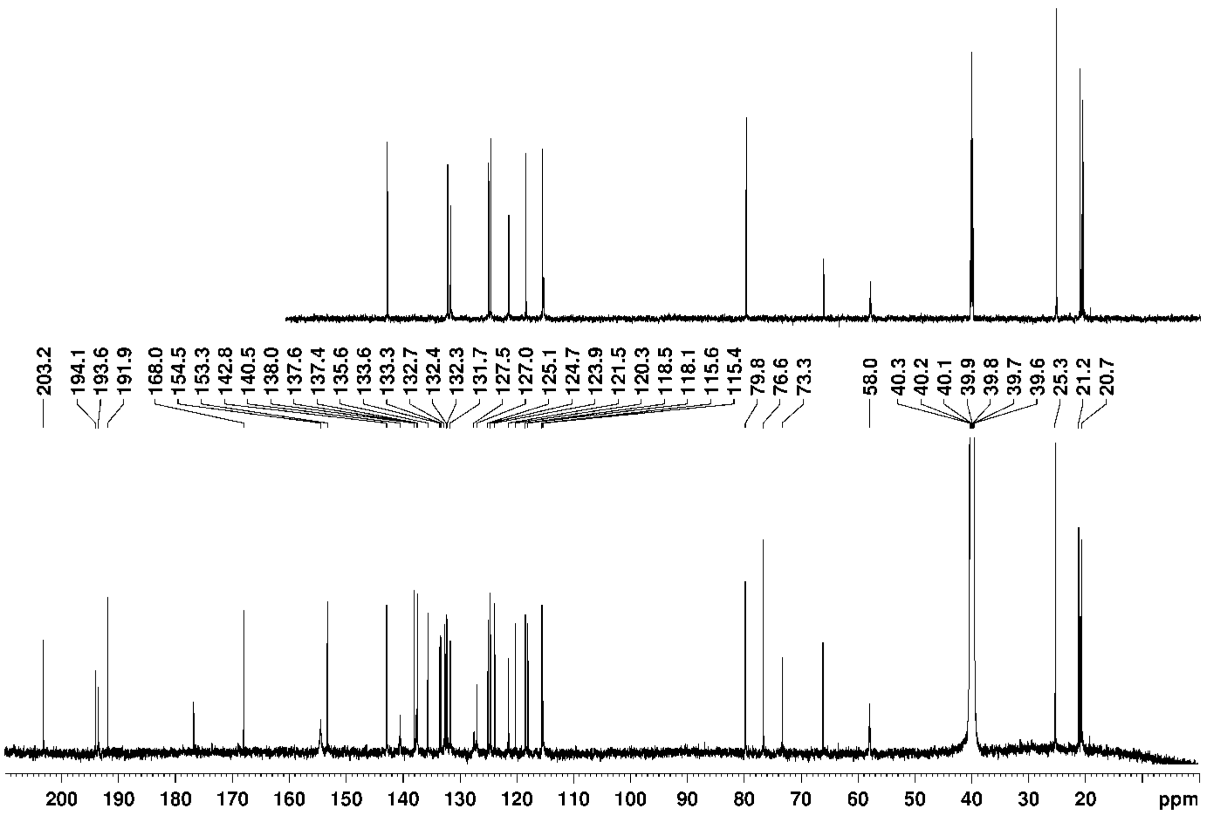

Supplementary Figure 25. The ^{13}C and DEPT 135 NMR spectrum of difluostatin B (**15**) in DMSO-d_6.

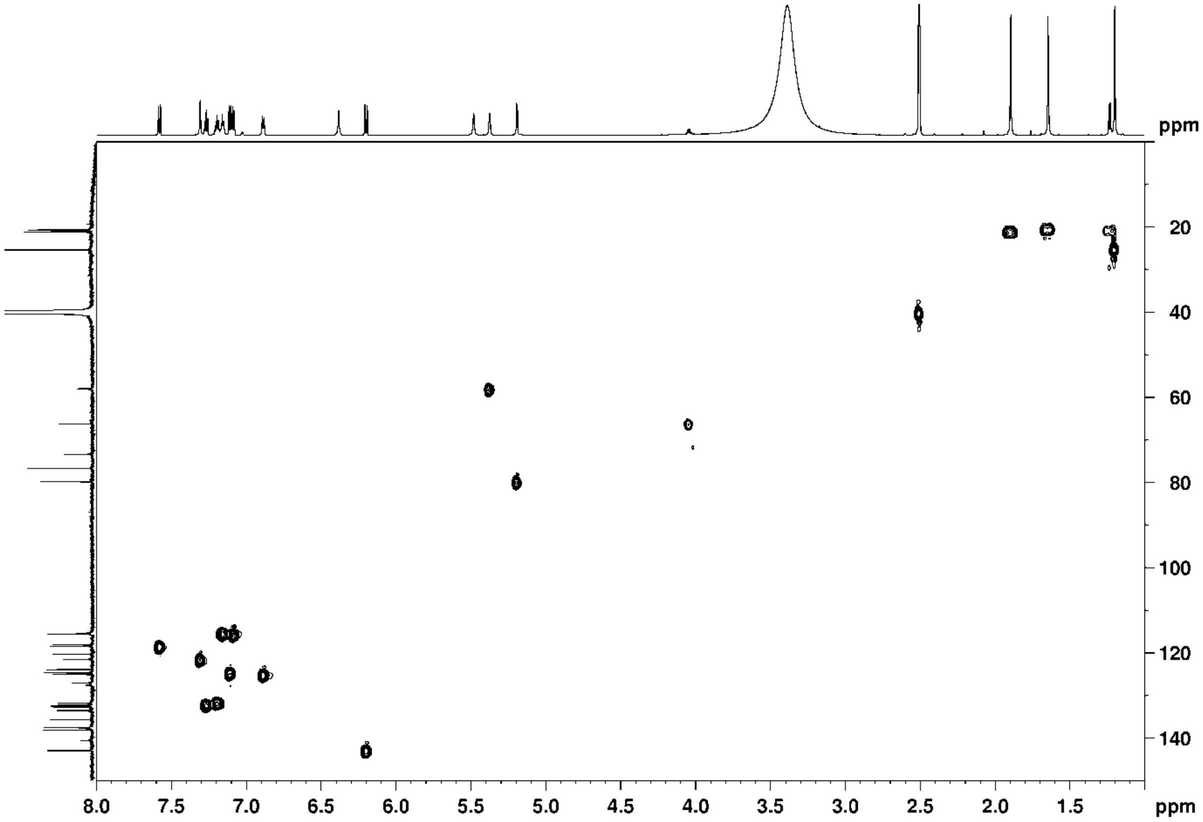

Supplementary Figure 26. The HSQC spectrum of difluostatin B (**15**) in DMSO-d_6.

a

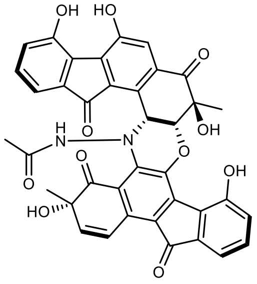

difluostatin B (**15**)

b

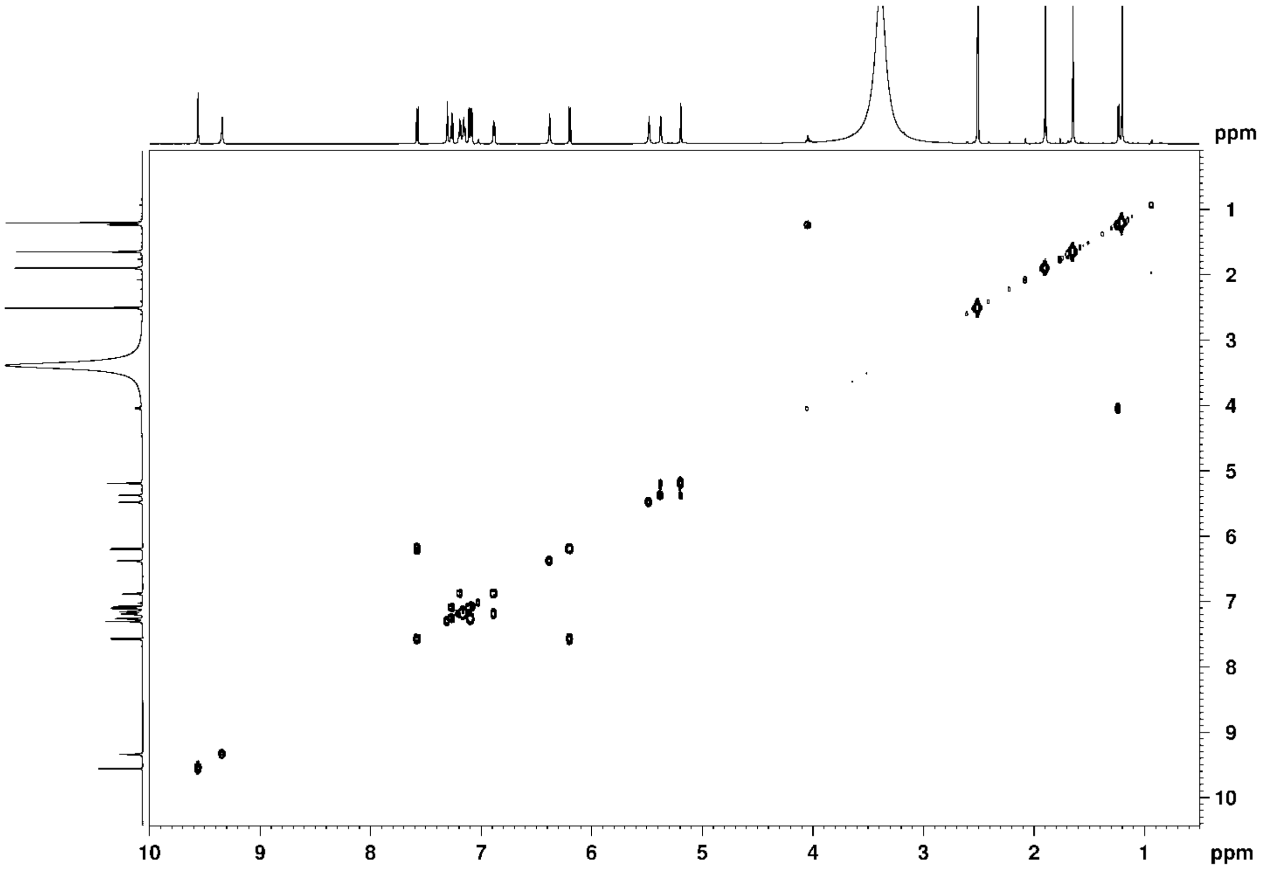

Supplementary Figure 27. The COSY spectrum of difluostatin B (**15**) in DMSO-d_6. **a** COSY correlations are indicated by boldface bonds. **b** The COSY spectrum.

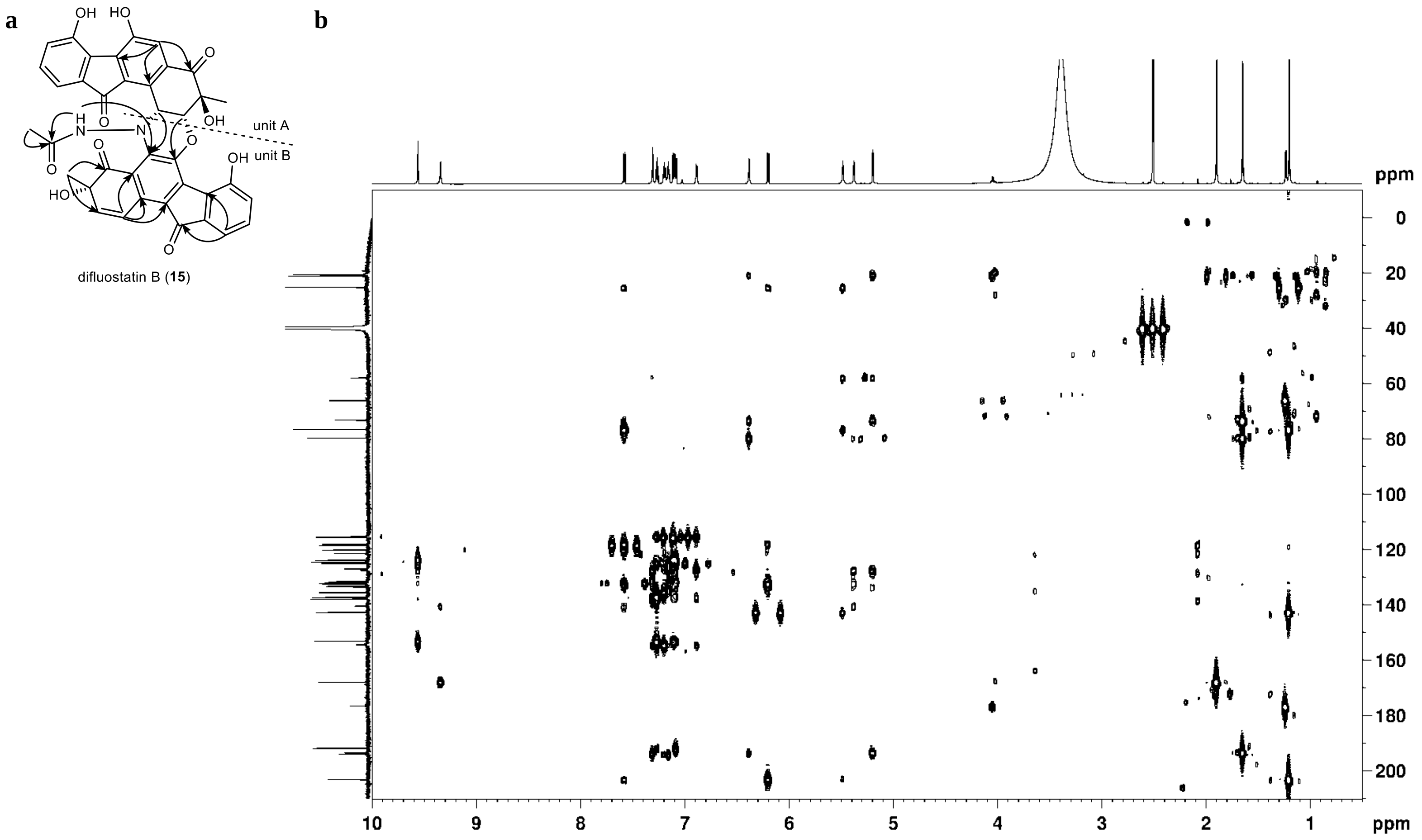

Supplementary Figure 28. The HMBC spectrum of difluostatin B (**15**) in DMSO-d_6. **a** Selected key HMBC correlations are indicated by the curved arrows. **b** The HMBC spectrum.

a

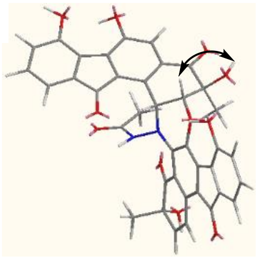

b

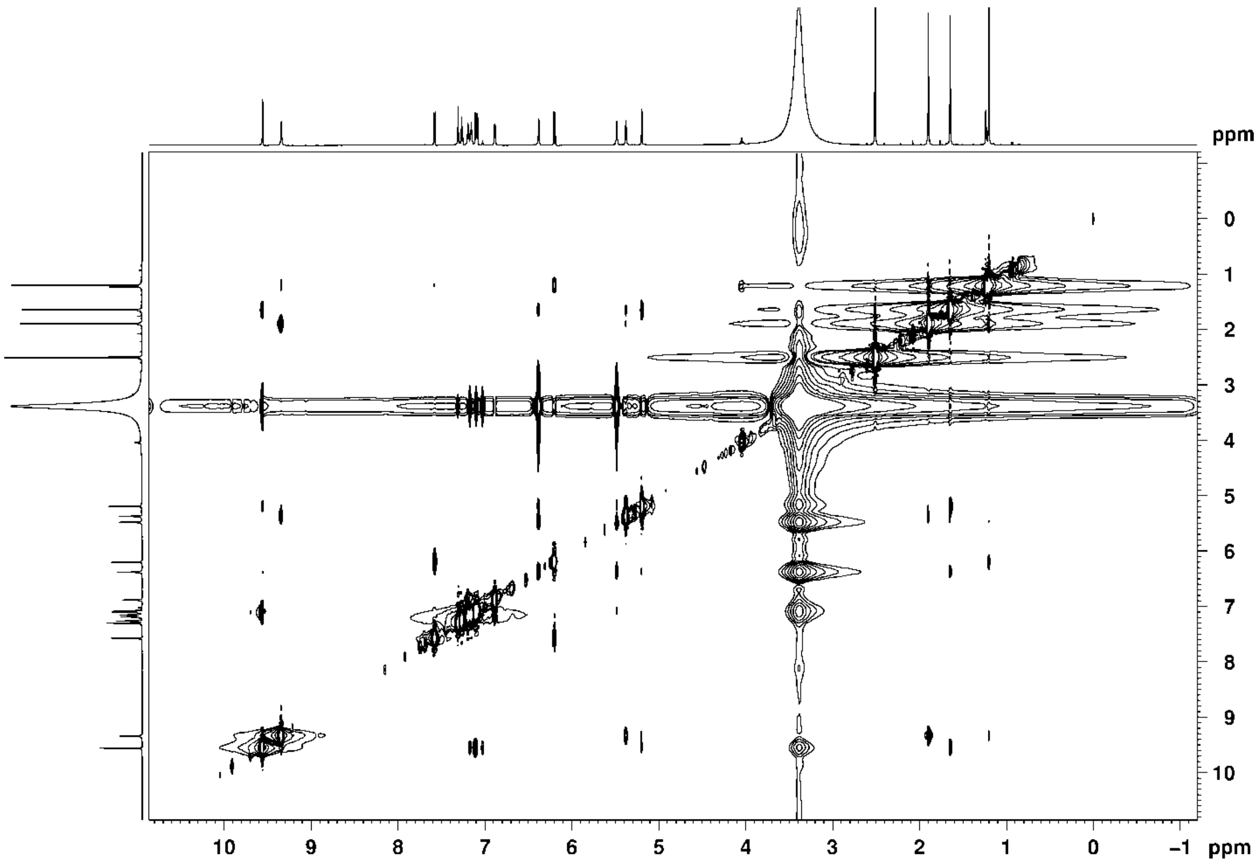

Supplementary Figure 29. The NOESY spectrum of difluostatin B (**15**) in DMSO-d_6. **a** Selected key NOESY correlations are indicated by the curved, double-headed arrows. **b** The NOESY spectrum.

a HRESIMS

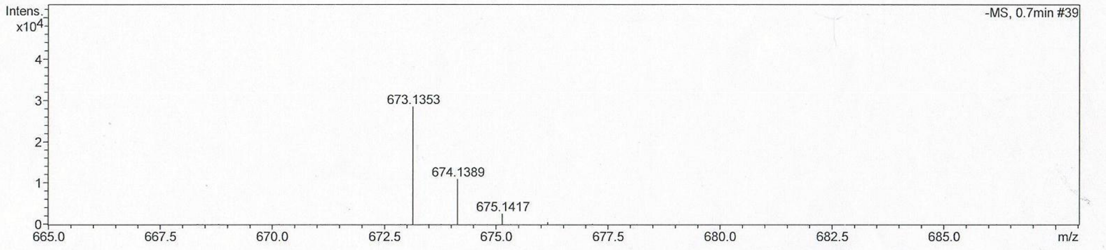

Meas. m/z	#	Formula	Score	m/z	err [ppm]	err [mDa]	mSigma	rdb	e⁻ Conf	N-Rule
673.1353	1	C 34 H 21 N 6 O 10	21.82	673.1325	-4.1	-2.8	6.0	27.5	even	ok
	2	C 35 H 17 N 10 O 6	51.62	673.1338	-2.2	-1.4	16.2	32.5	even	ok
	3	C 32 H 9 N 20	17.02	673.1325	-4.2	-2.8	18.4	38.5	even	ok
	4	C 38 H 25 O 12	100.00	673.1351	-0.2	-0.1	18.9	26.5	even	ok
	5	C 39 H 21 N 4 O 8	45.25	673.1365	1.8	1.2	28.3	31.5	even	ok
	6	C 36 H 13 N 14 O 2	79.34	673.1351	-0.2	-0.1	28.9	37.5	even	ok
	7	C 27 H 25 N 6 O 15	9.40	673.1383	4.6	3.1	32.3	18.5	even	ok
	8	C 40 H 17 N 8 O 4	12.11	673.1378	3.8	2.6	40.9	36.5	even	ok

b IR

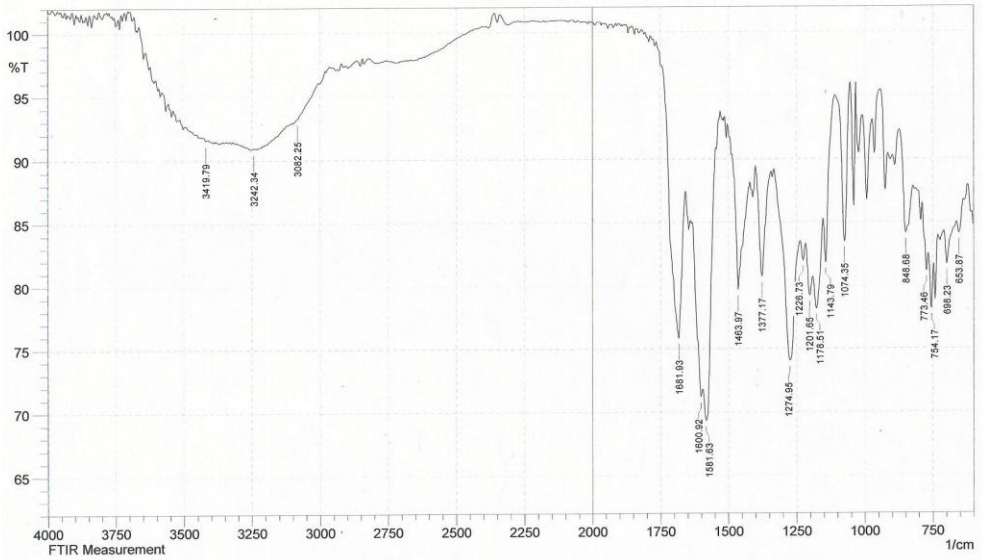

c UV

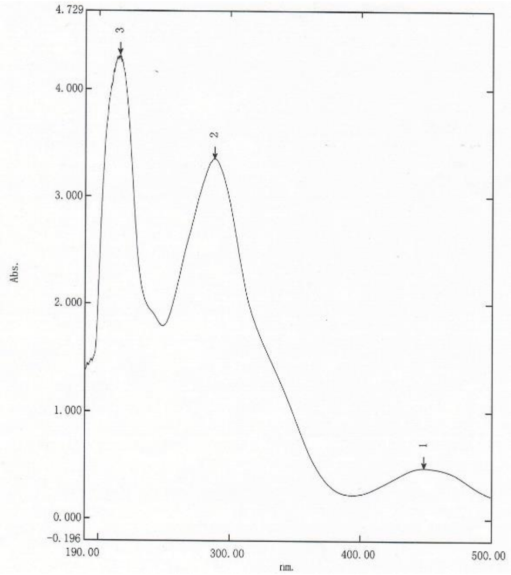

Supplementary Figure 30. Spectroscopic data for difluostatin C (16). a HRESIMS, **b** IR, and **c** UV spectra of difluostatin C (**16**).

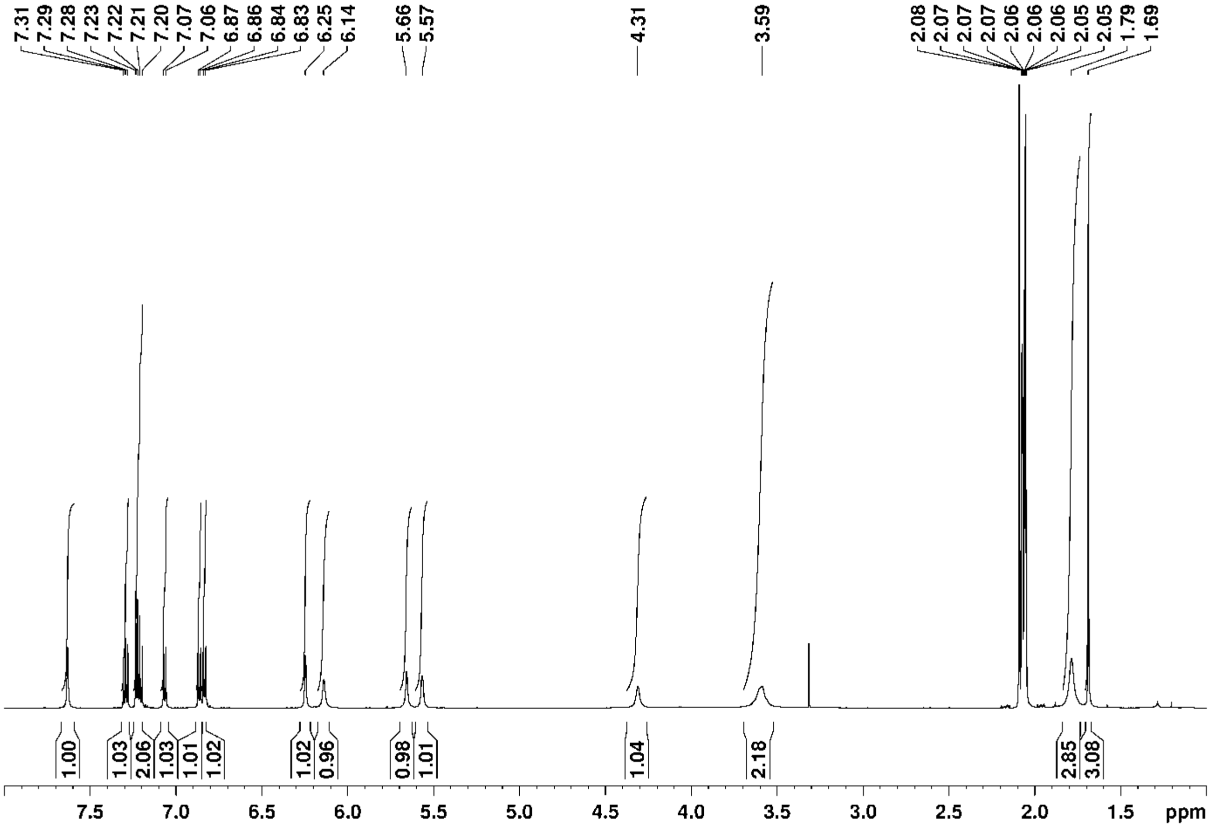

Supplementary Figure 31. The ^{1}H NMR spectrum of difluostatin C (**16**) in acetone-d_6.

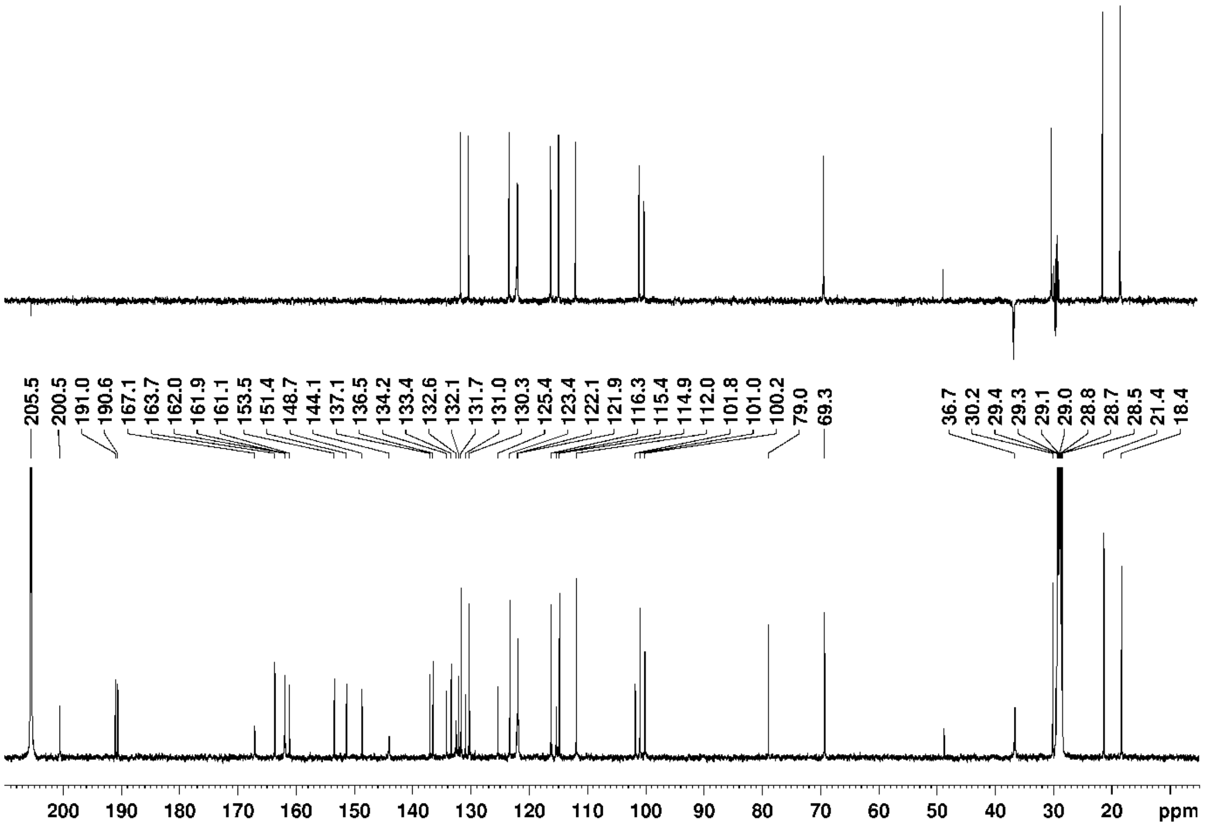

Supplementary Figure 32. The ^{13}C and DEPT 135 NMR spectrum of difluostatin C (**16**) in acetone-d_6.

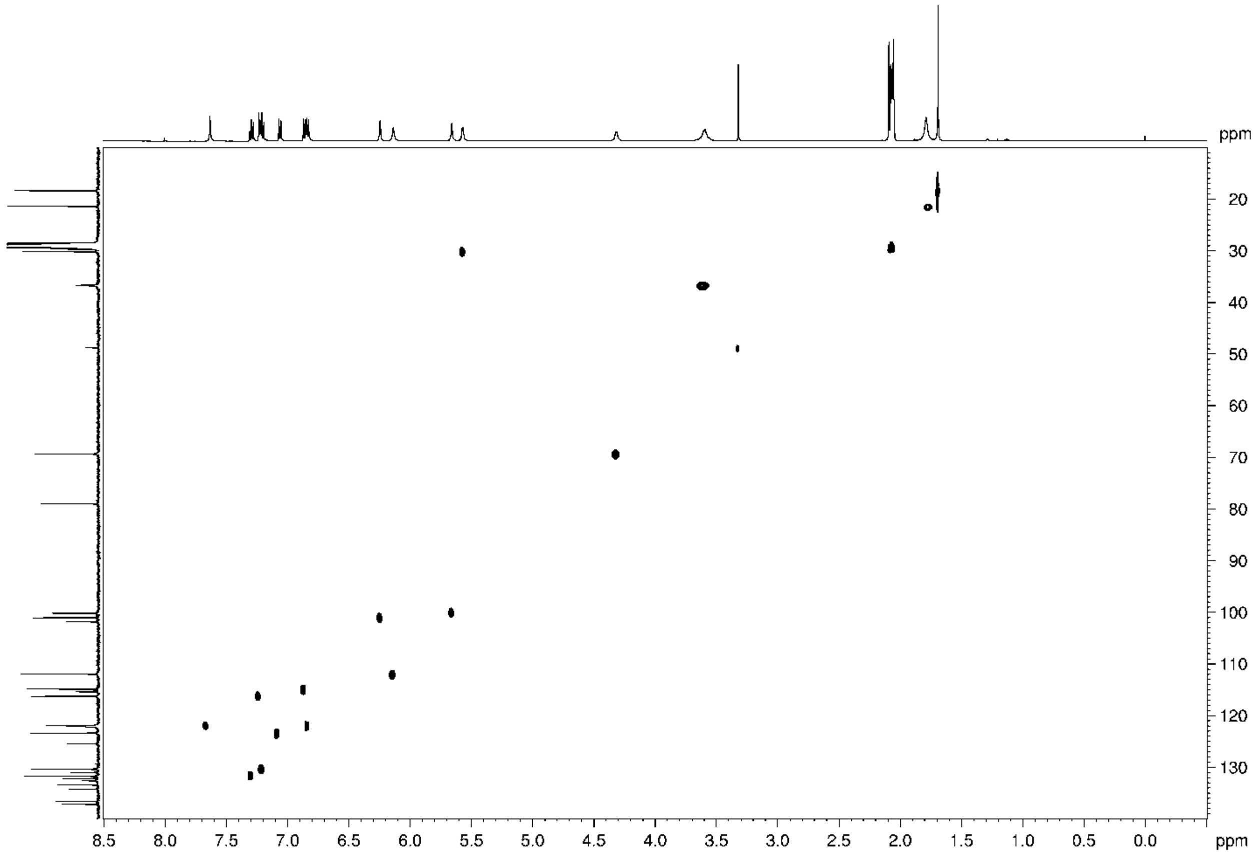

Supplementary Figure 33. The HSQC spectrum of difluostatin C (**16**) in acetone-d_6.

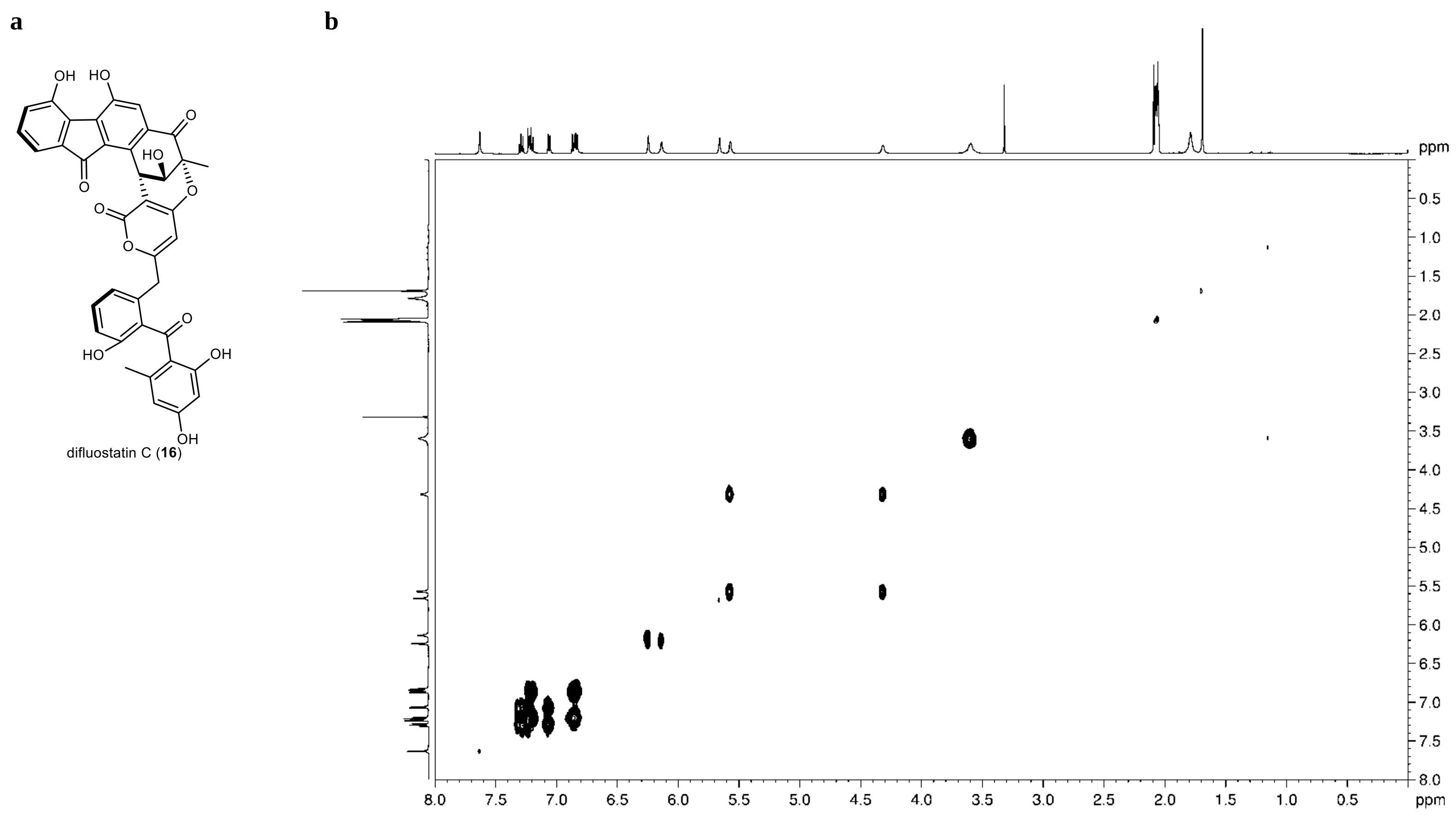

Supplementary Figure 34. The COSY spectrum of difluostatin C (**16**) in acetone-d_6. **a** COSY correlations are indicated by boldface bonds. **b** The COSY spectrum.

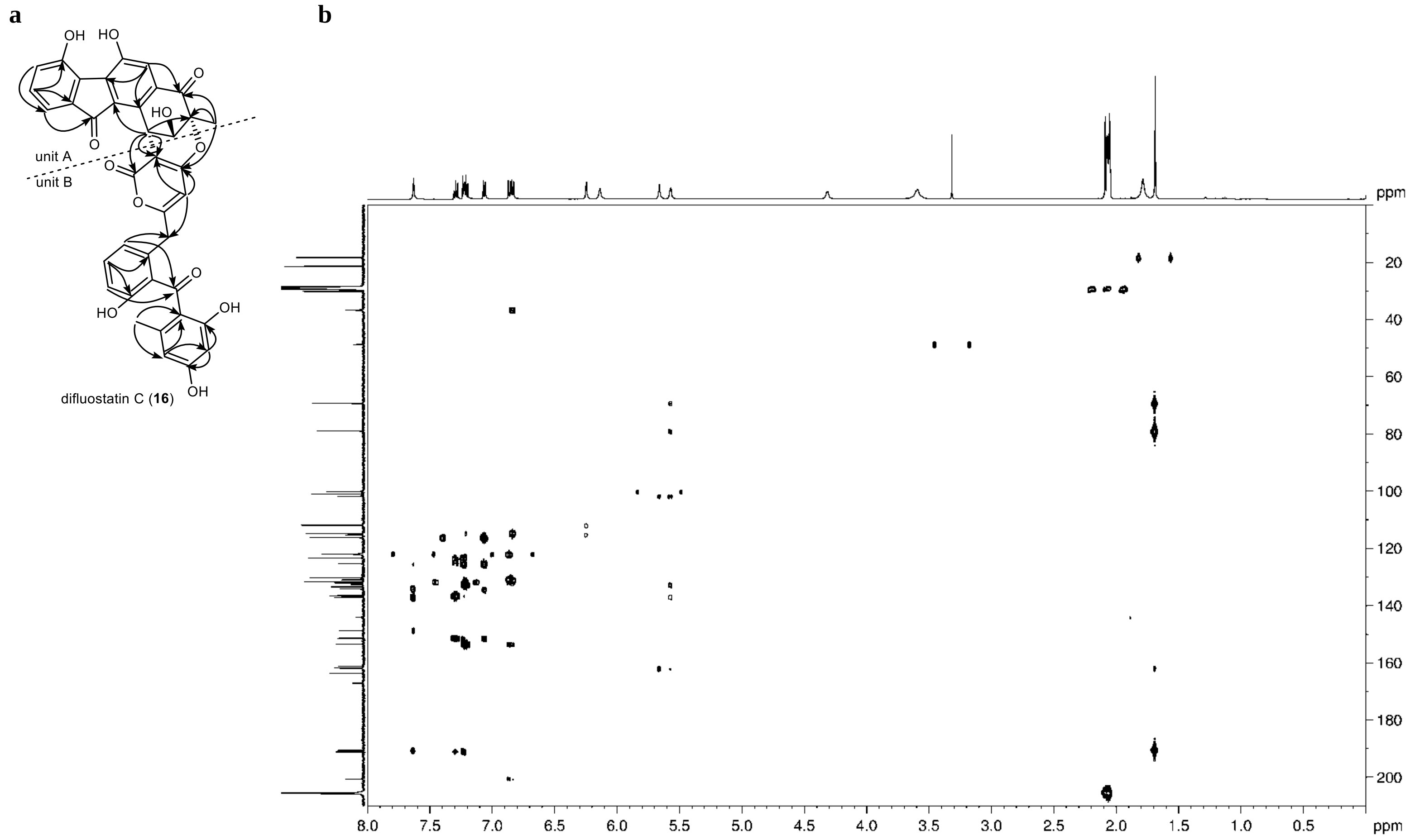

Supplementary Figure 35. The HMBC spectrum of difluostatin C (**16**) in acetone-d_6. **a** Selected key HMBC correlations are indicated by the curved arrows. **b** The HMBC spectrum.

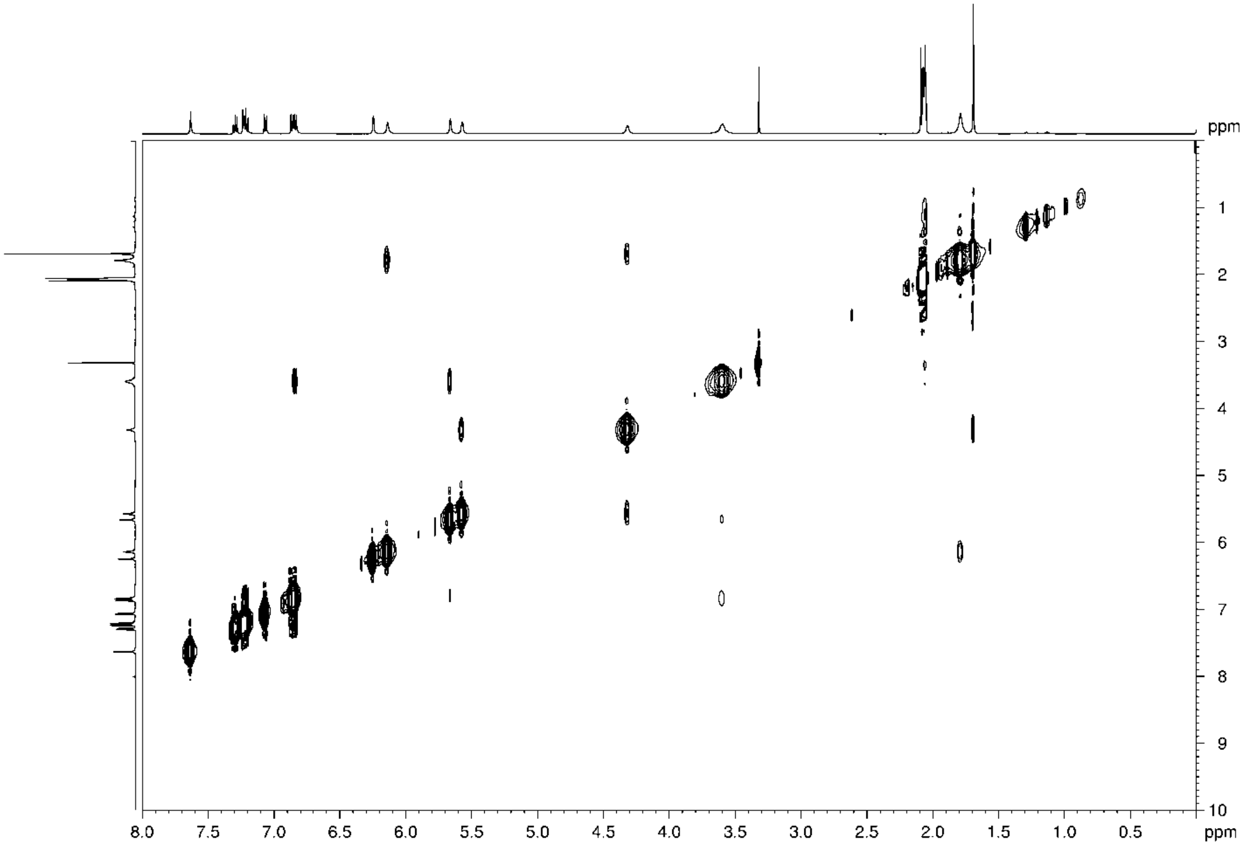

Supplementary Figure 36. The NOESY spectrum of difluostatin C (**16**) in acetone-d_6.

a HRESIMS

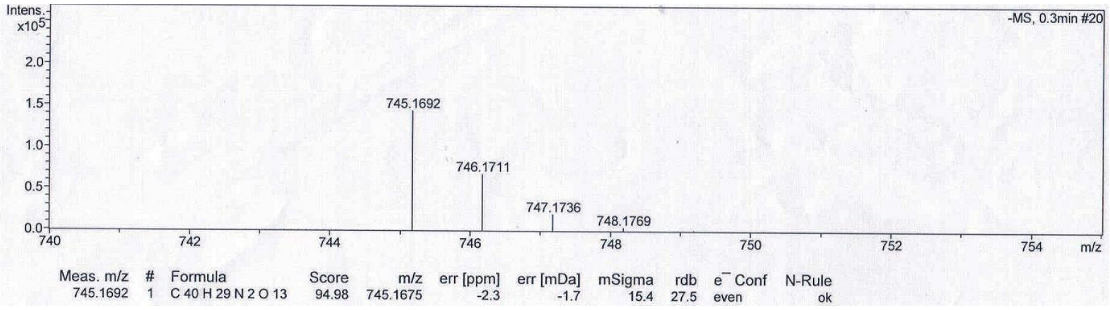

b IR

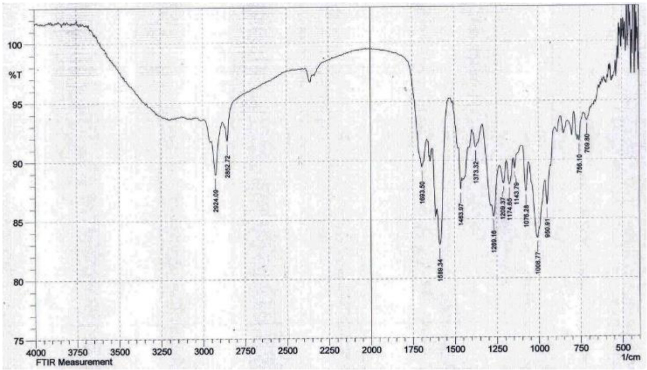

c UV

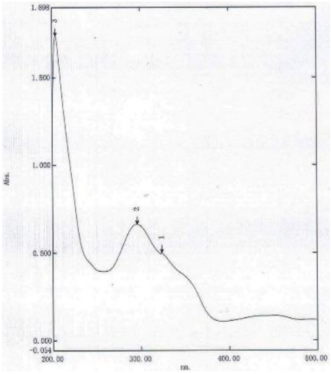

d experimental ECD spectrum

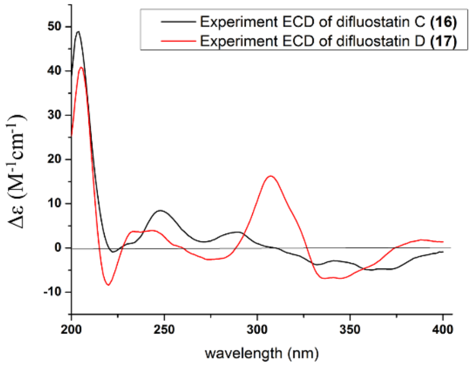

Supplementary Figure 37. Spectroscopic data for difluostatin D (17). a HRESIMS, **b** IR, **c** UV spectra of difluostatin D (**17**), and **d** experimental ECD spectrum of difluostatin C (**16**) and difluostatin D (**17**).

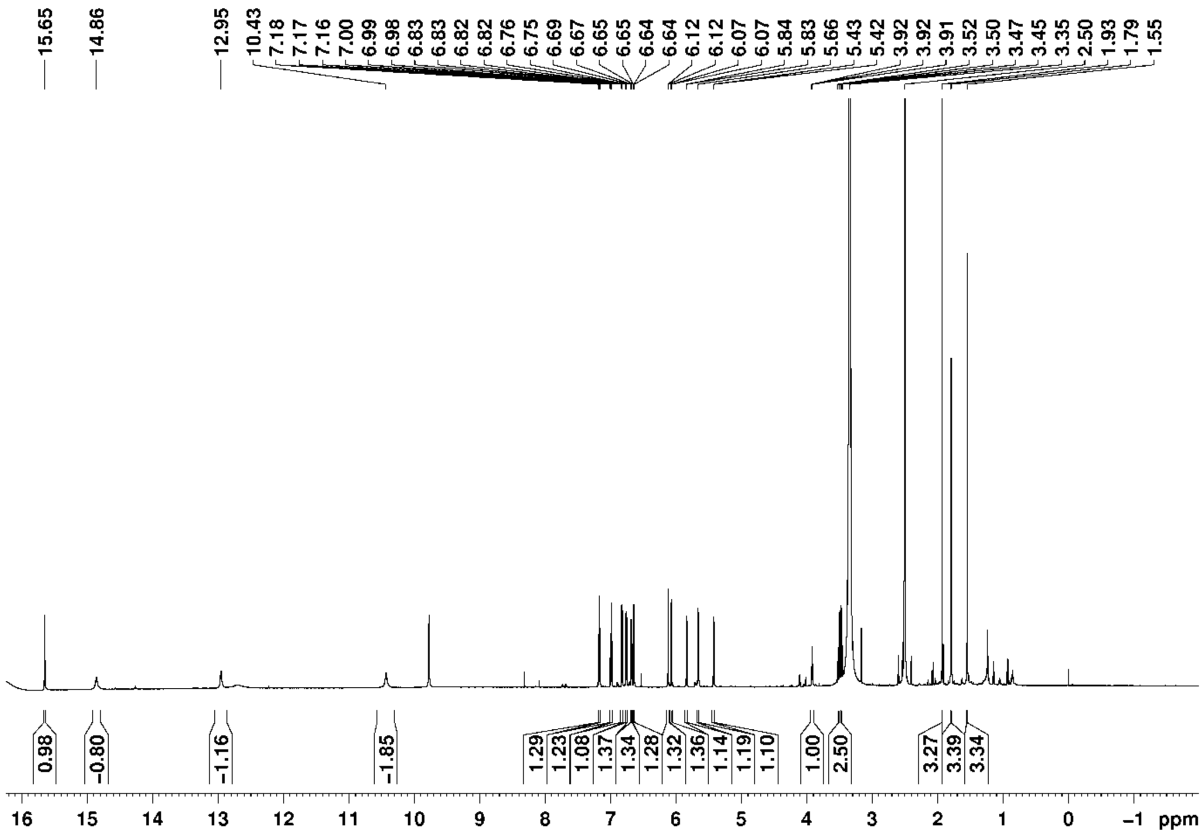

Supplementary Figure 38. The ^{1}H NMR spectrum of difluostatin D (**17**) in DMSO-d_6.

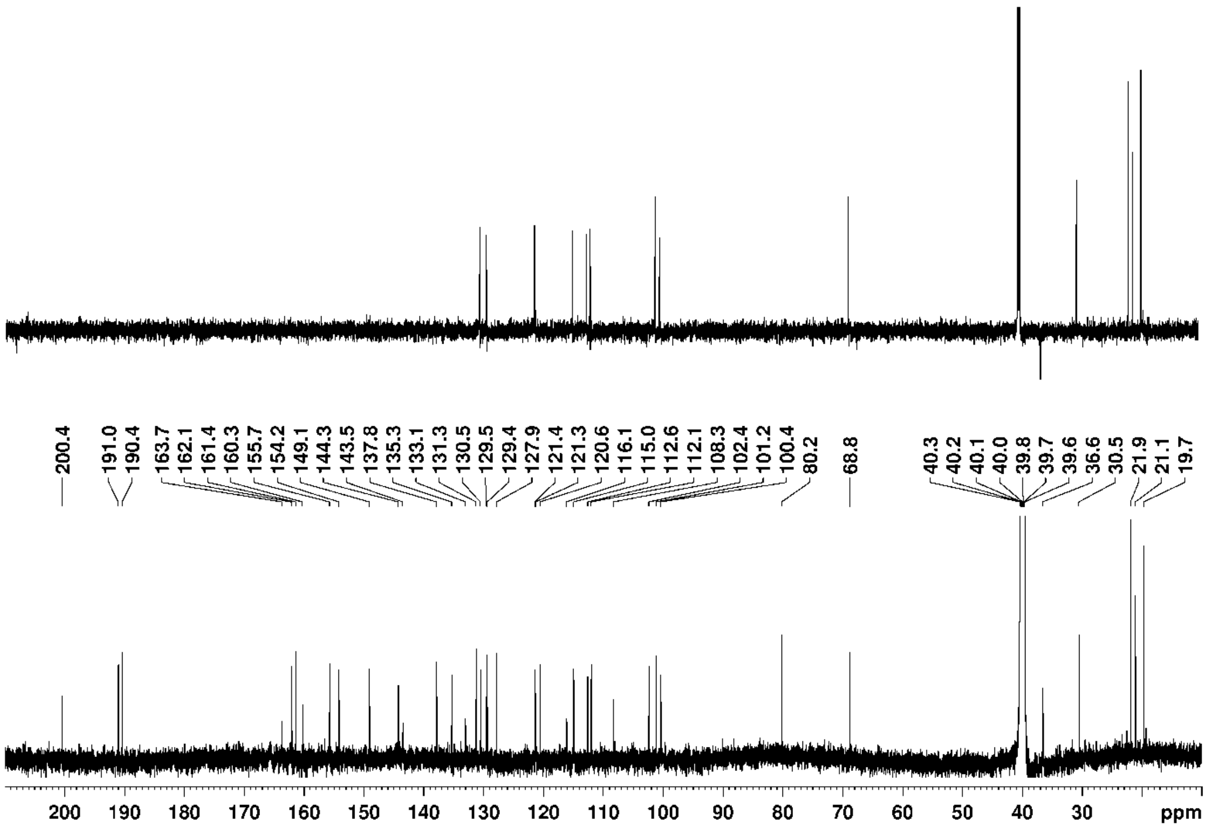

Supplementary Figure 39. The ^{13}C and DEPT 135 NMR spectrum of difluostatin D (**17**) in DMSO-d_6.

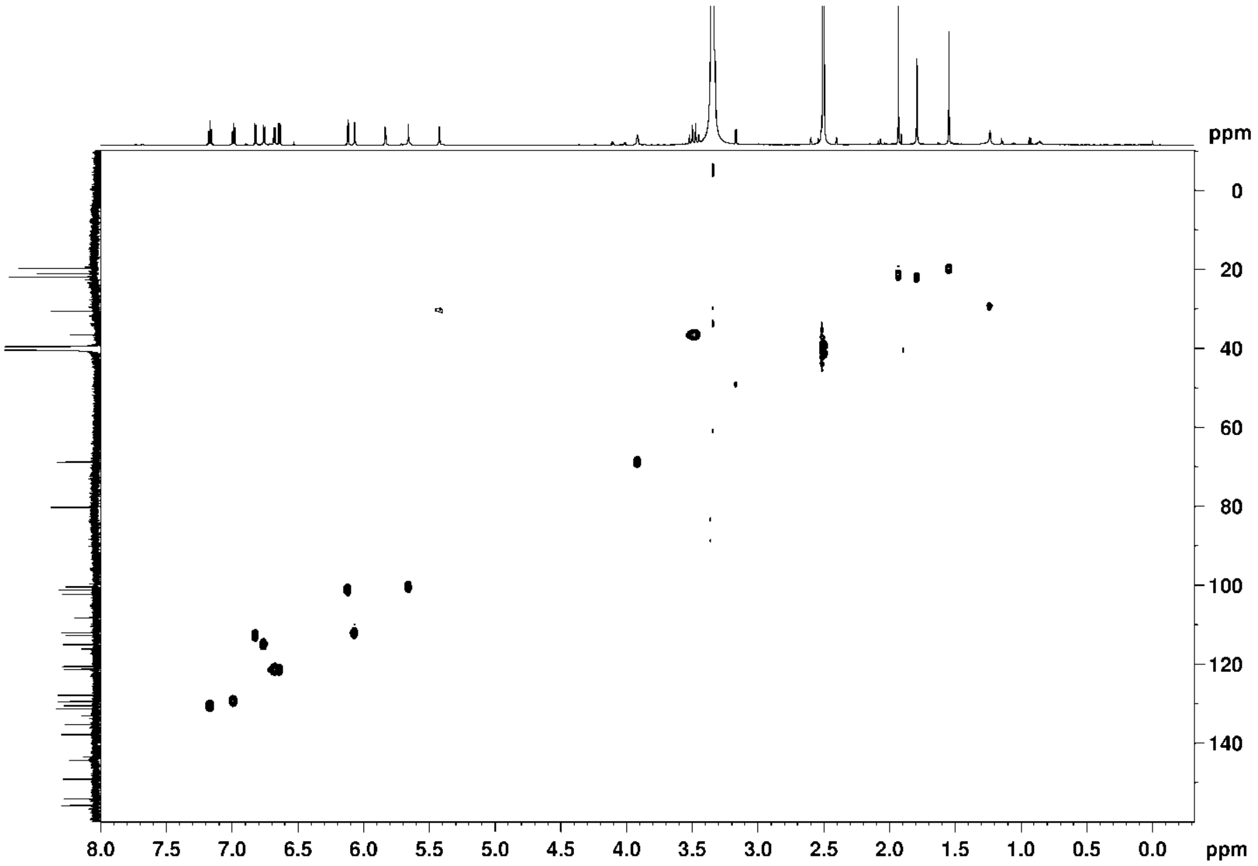

Supplementary Figure 40. The HSQC spectrum of difluostatin D (**17**) in DMSO-d_6.

a

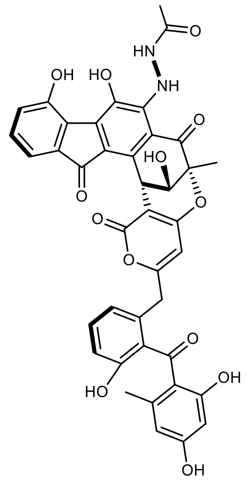

difluostatin D (**17**)

b

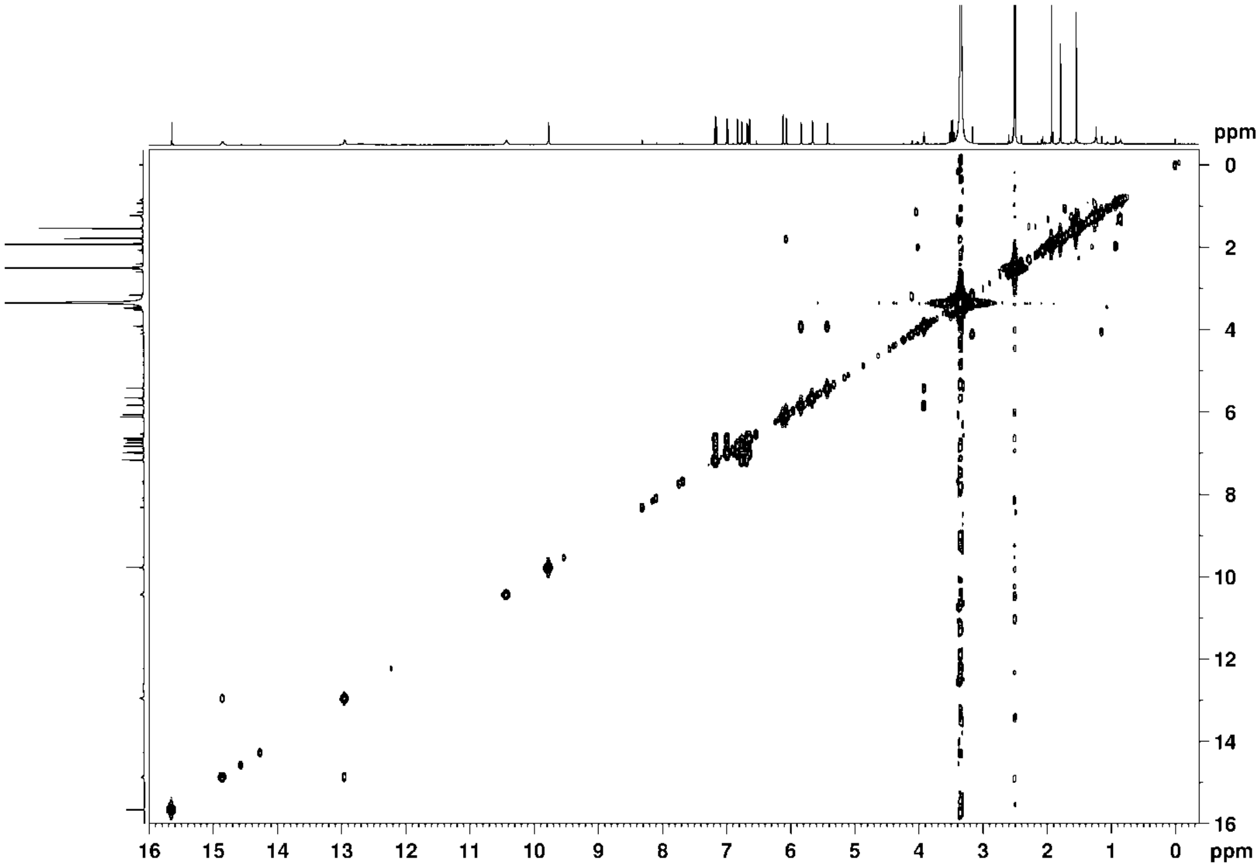

Supplementary Figure 41. The COSY spectrum of difluostatin D (**17**) in DMSO-d_6. **a** COSY correlations are indicated by boldface bonds. **b** The COSY spectrum.

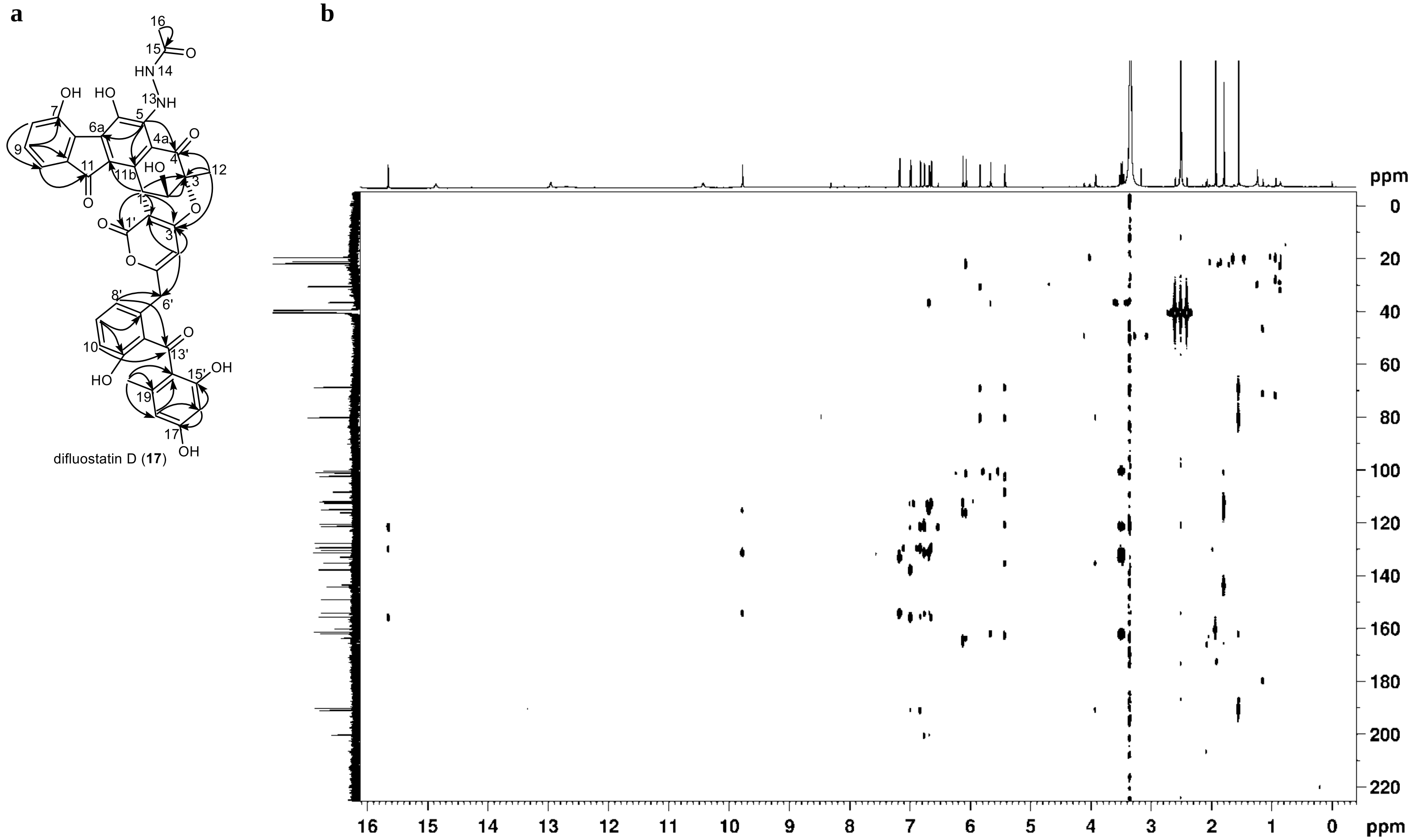

Supplementary Figure 42. The HMBC spectrum of difluostatin D (**17**) in DMSO-d_6. **a** Selected key HMBC correlations are indicated by the curved arrows. **b** The HMBC spectrum.

a **b**

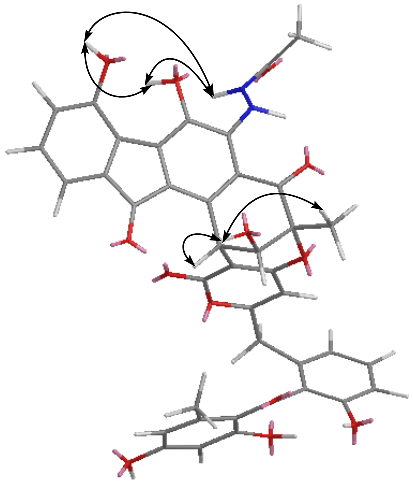

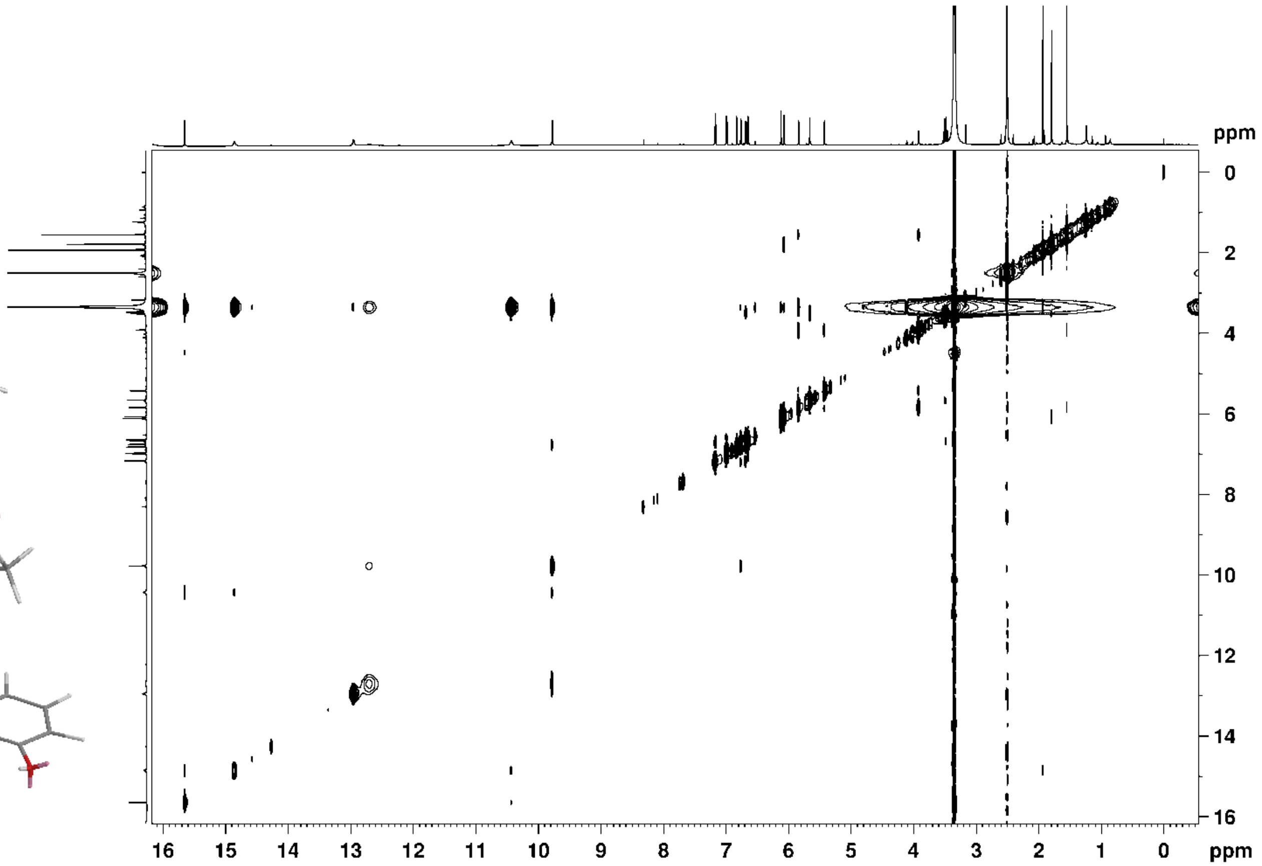

Supplementary Figure 43. The NOESY spectrum of difluostatin D (**17**) in DMSO-d_6. **a** Selected key NOESY correlations are indicated by the curved, double-headed arrows. **b** The NOESY spectrum.

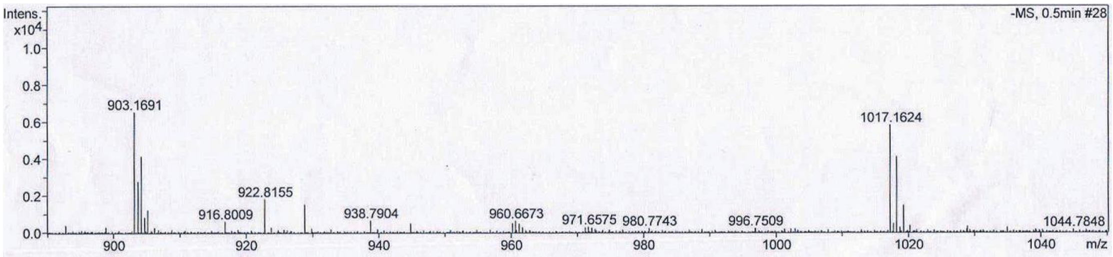

Meas. m/z	#	Formula	Score	m/z	err [ppm]	err [mDa]	mSigma	rdb	e⁻ Conf	N-Rule
903.1691	1	C 54 H 31 O 14	44.70	903.1719	3.1	2.8	19.9	39.5	even	ok
	2	C 49 H 31 N 2 O 16	100.00	903.1679	-1.3	-1.2	39.3	35.5	even	ok
	3	C 45 H 27 N 8 O 14	5.48	903.1652	-4.3	-3.9	57.9	36.5	even	ok

Supplementary Figure 44. HRESIMS spectrum of trifluostatin A (**18**).

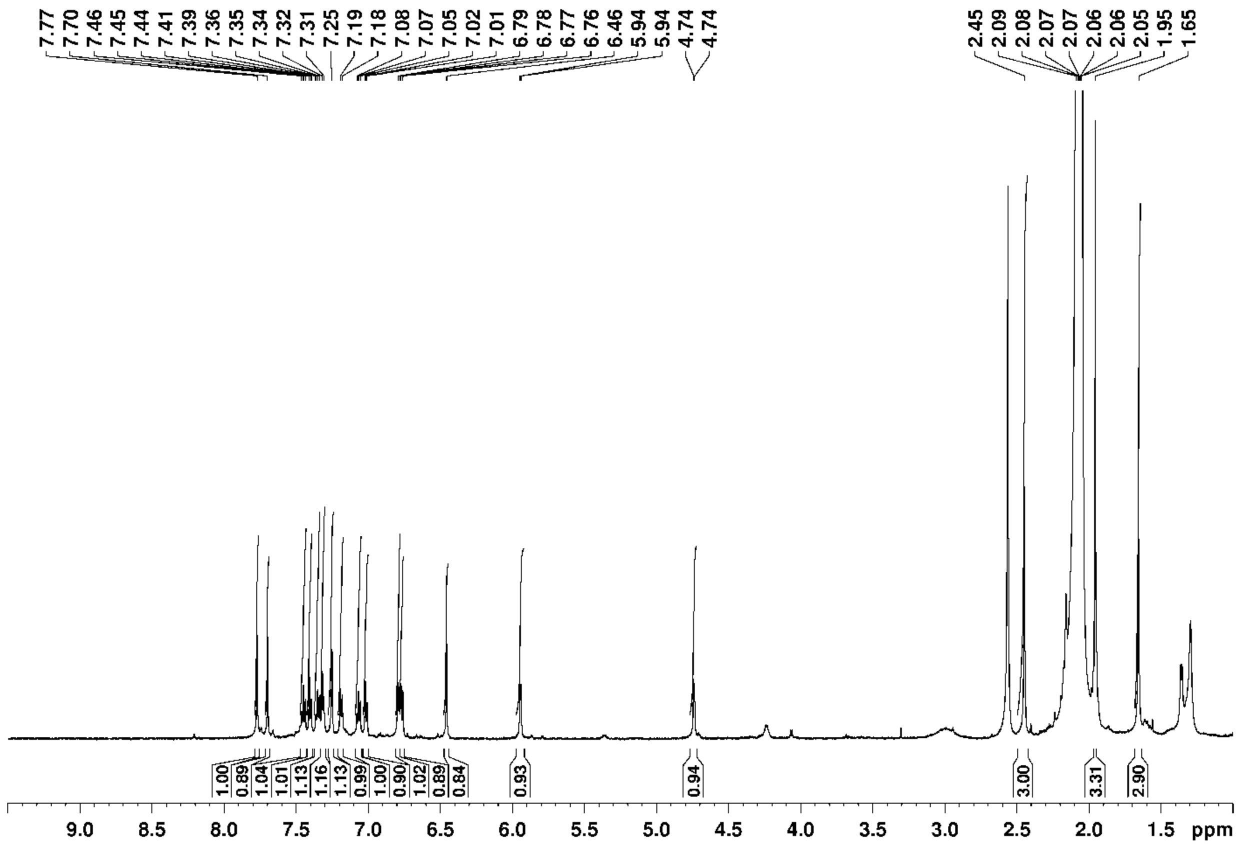

Supplementary Figure 45. The ^{1}H NMR spectrum of trifluostatin A (**18**) in acetone-d_6.

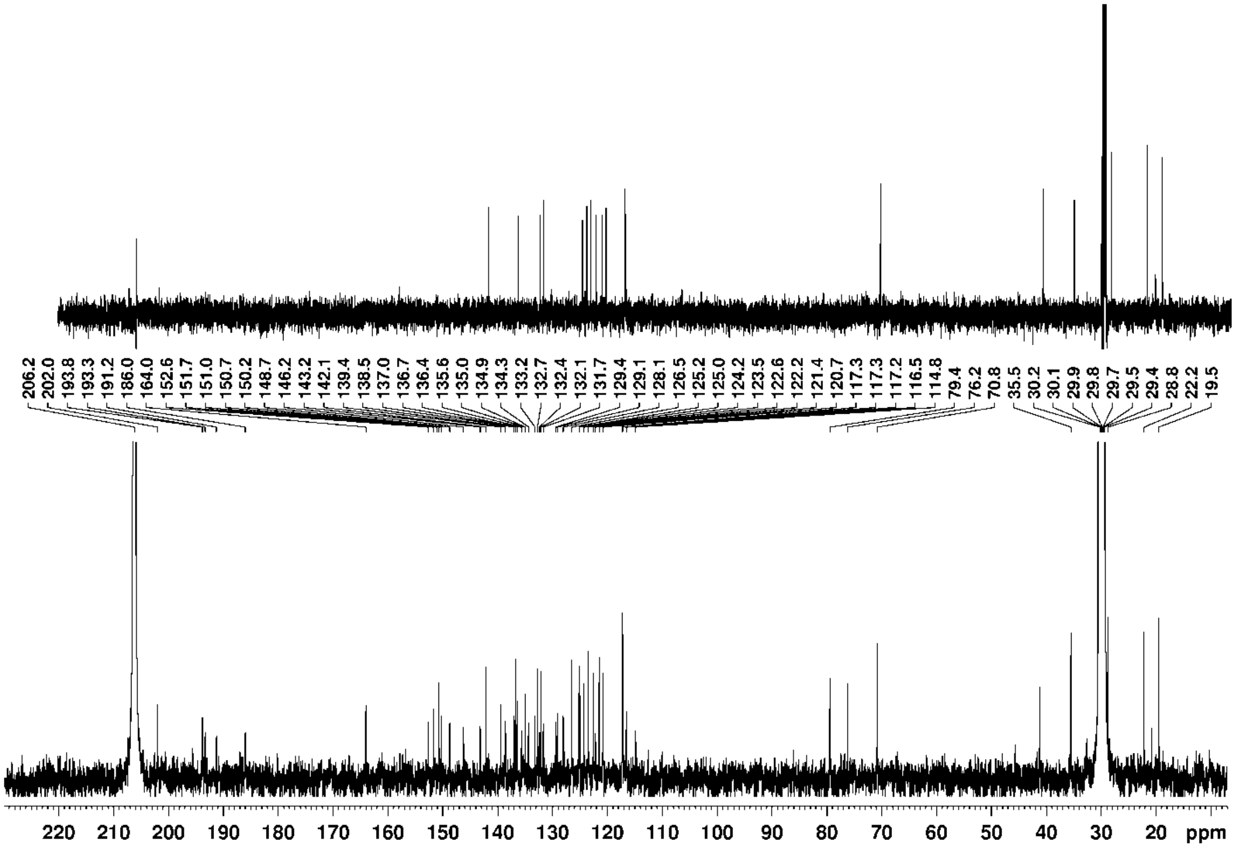

Supplementary Figure 46. The ^{13}C and DEPT 135 NMR spectrum of trifluostatin A **(18)** in acetone-d_6.

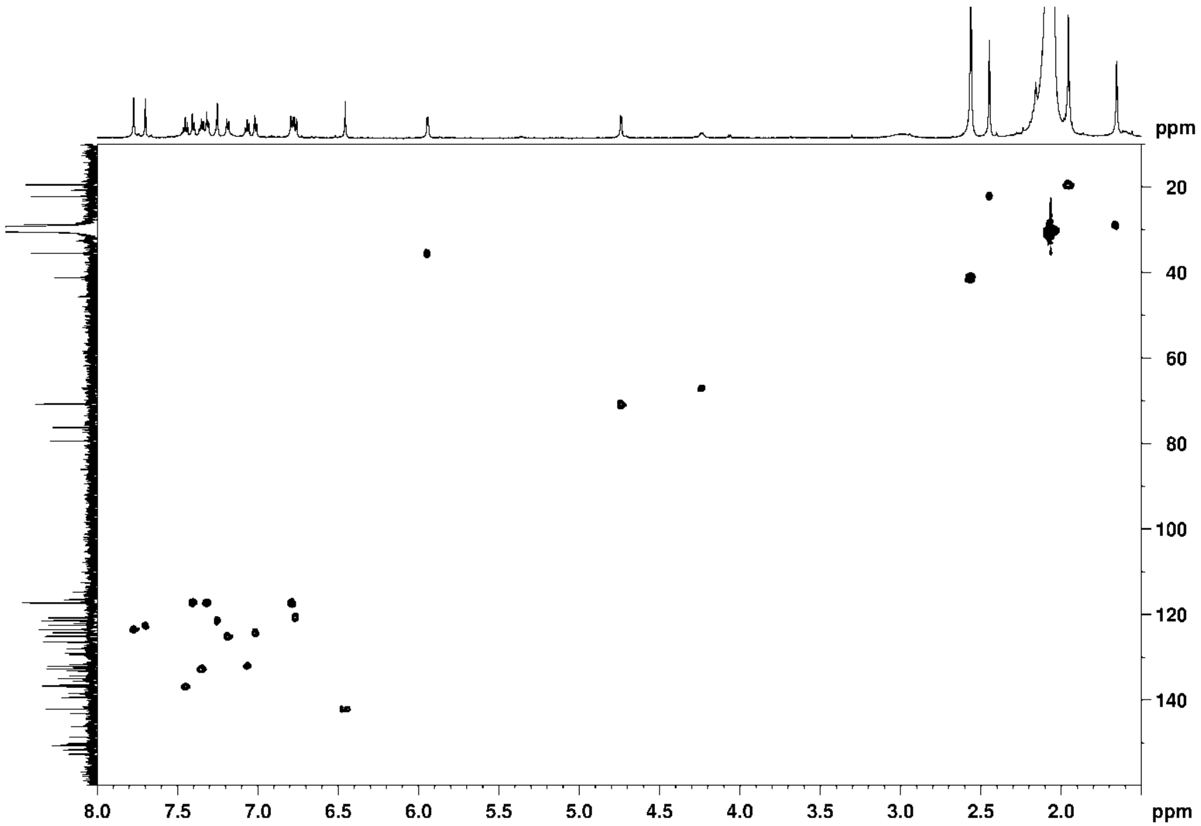

Supplementary Figure 47. The HSQC spectrum of trifluostatin A **(18)** in acetone-d_6.

a

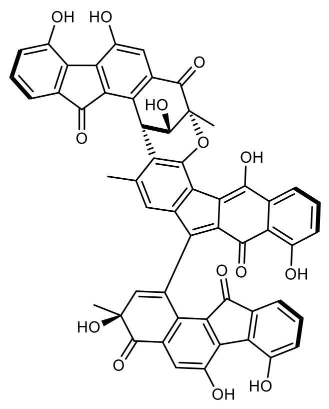

trifluostatin A (**18**)

b

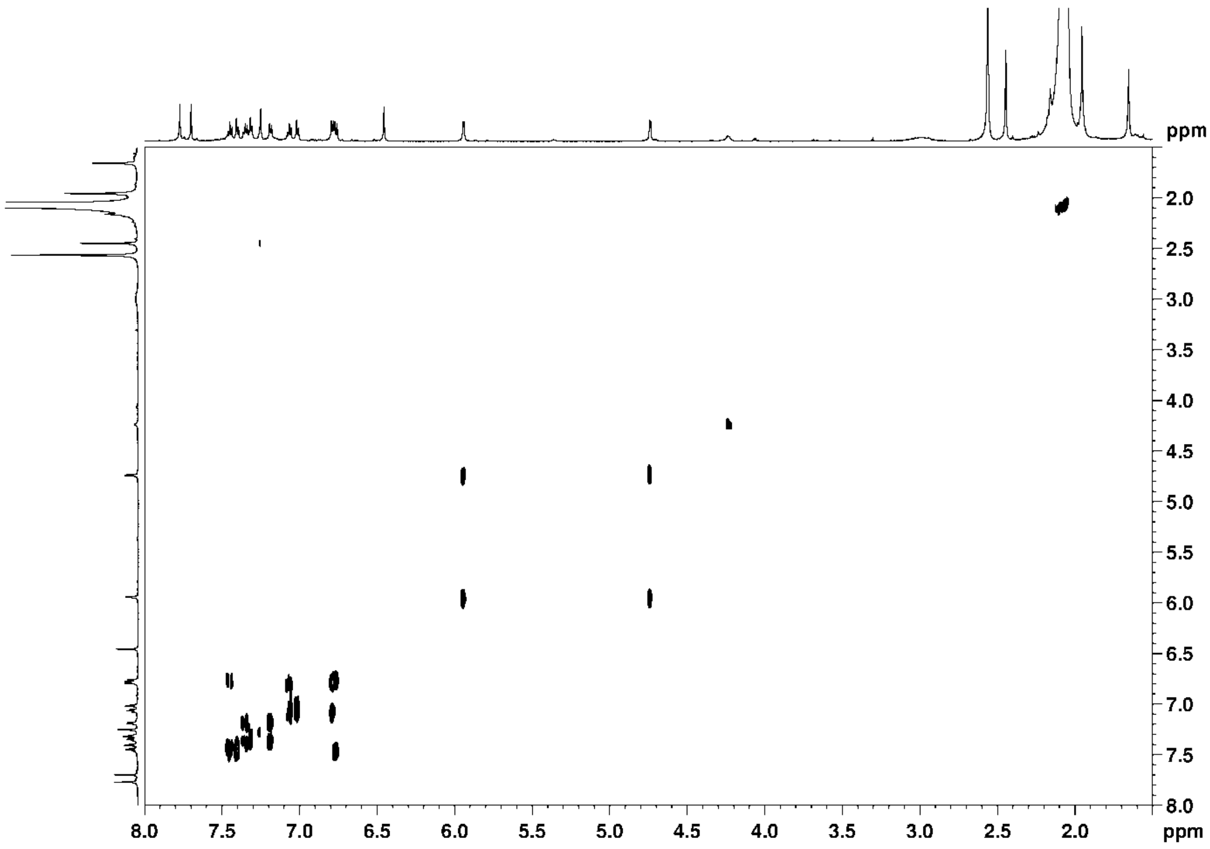

Supplementary Figure 48. The COSY spectrum of trifluostatin A **(18)** in acetone-d_6. **a** COSY correlations are indicated by boldface bonds. **b** The COSY spectrum.

a

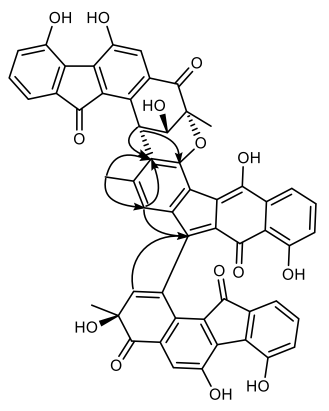

trifluostatin A (**18**)

b

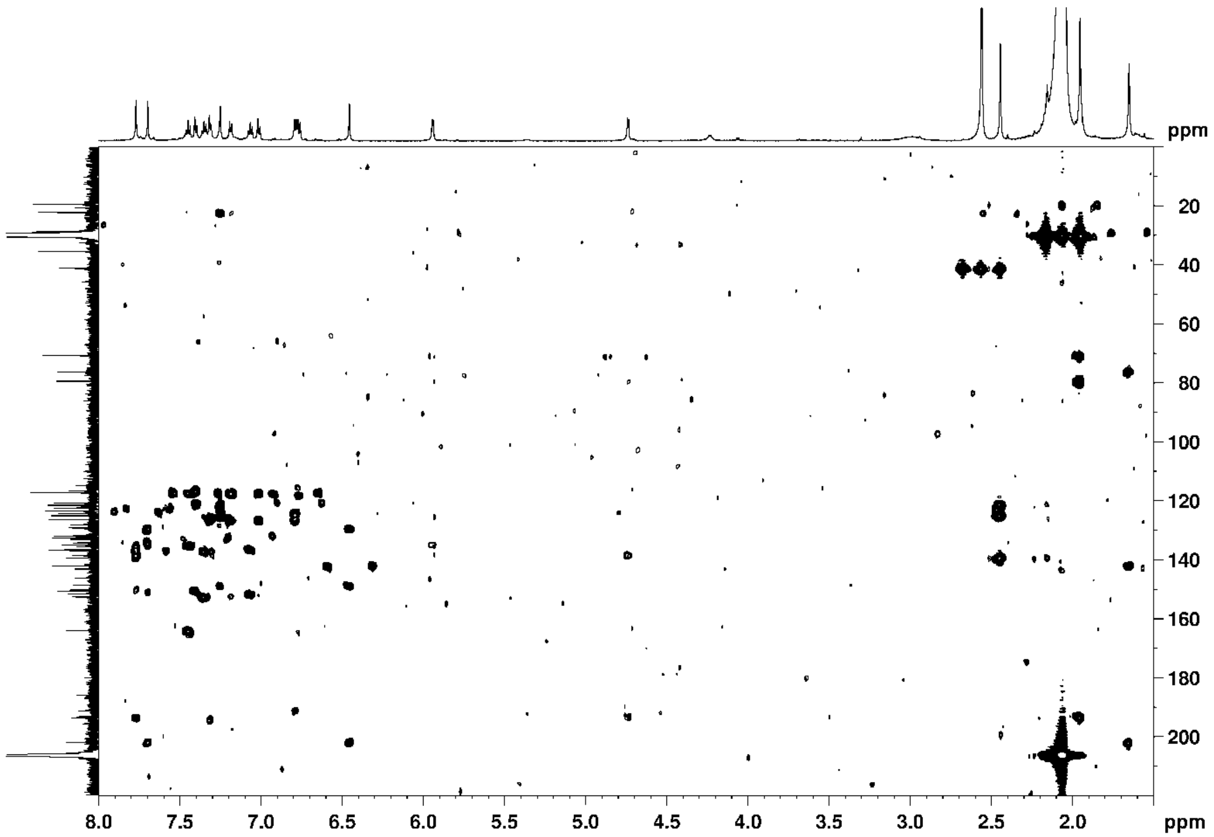

Supplementary Figure 49. The HMBC spectrum of trifluostatin A **(18)** in acetone-d_6.**a** Selected key HMBC correlations are indicated by the curved arrows. **b** The HMBC spectrum.

a **b**

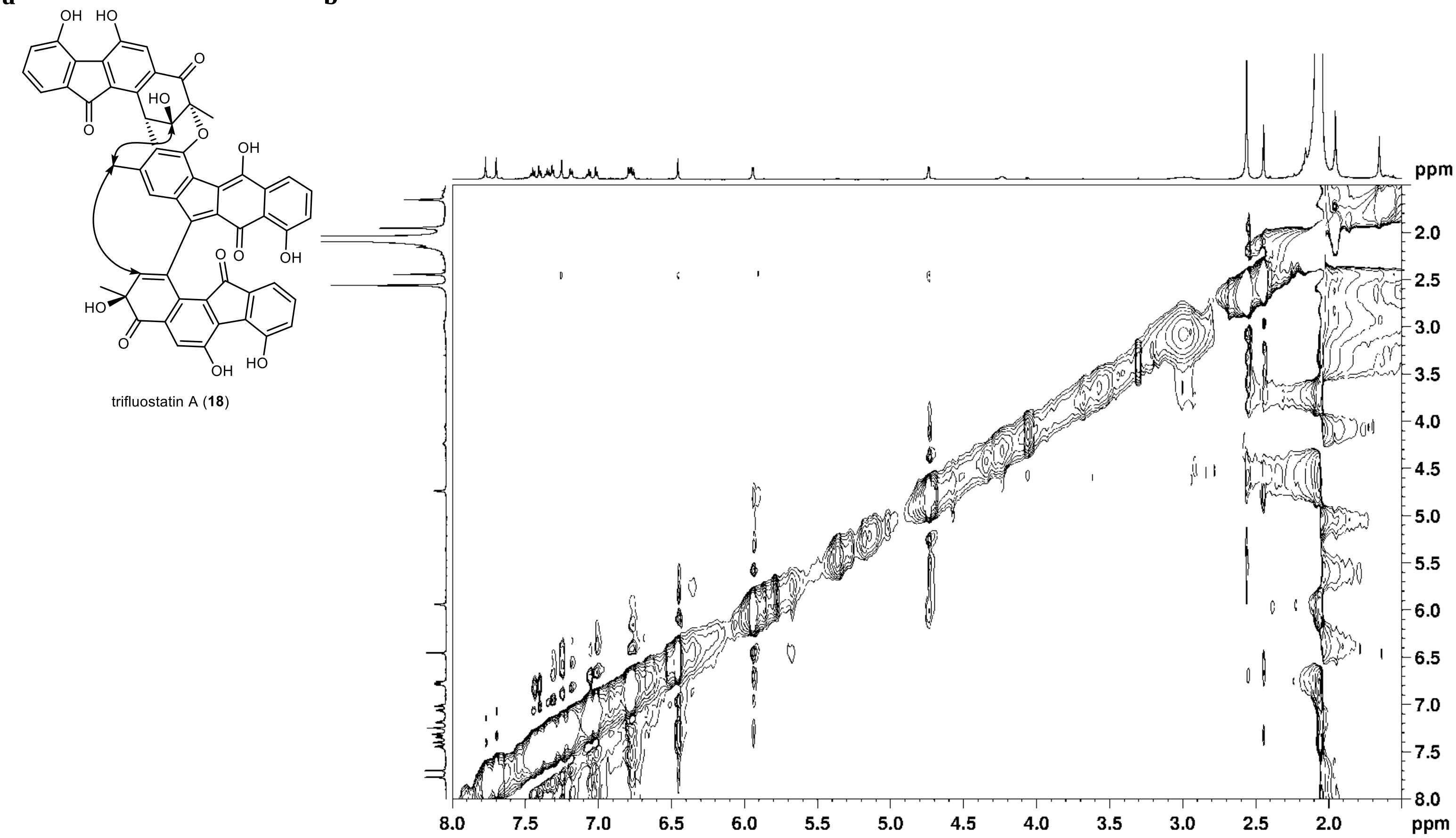

Supplementary Figure 50. The NOESY spectrum of trifluostatin A **(18)** in acetone-d_6. **a** Selected key NOESY correlations are indicated by the curved, double-headed arrows. **b** The NOESY spectrum.

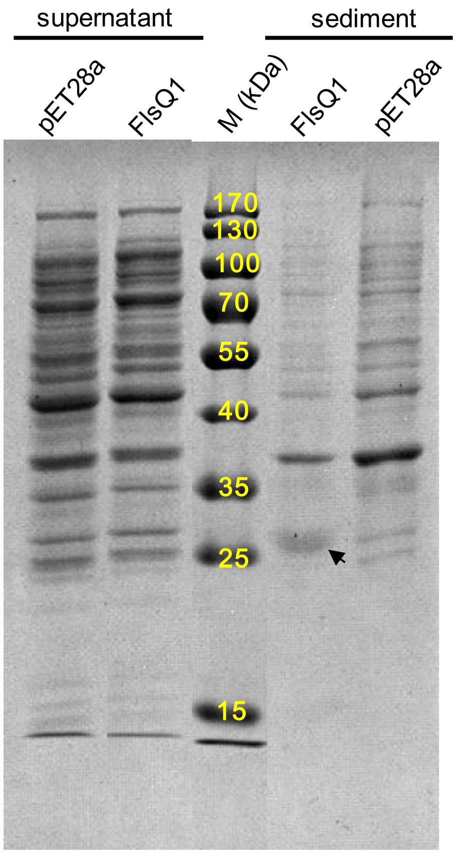

Supplementary Figure 51. The SDS-PAGE analysis of the expression of *flsQ1* in *E. coli* BL21(DE3); unfortunately, FlsQ1 was produced as an insoluble protein in *E. coli*.

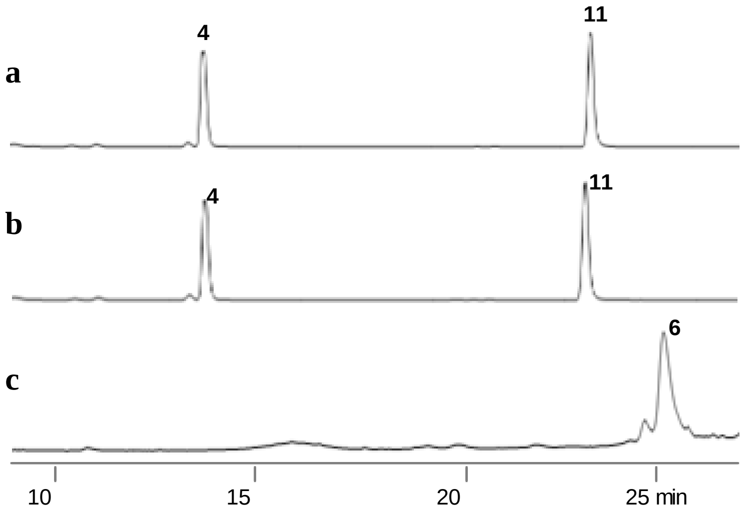

Supplementary Figure 52. HPLC analysis of biotransformation of FST C (4) and prefluostatin (11) by *E. coli* BL21(DE3). a *E. coli* BL21(DE3)/pET28a feeding with **4** and **11**; **b** *E. coli* BL21(DE3)/pCSG5209 (for the expression of *flsQ1*) feeding with **4** and **11**; **c** **6** std.

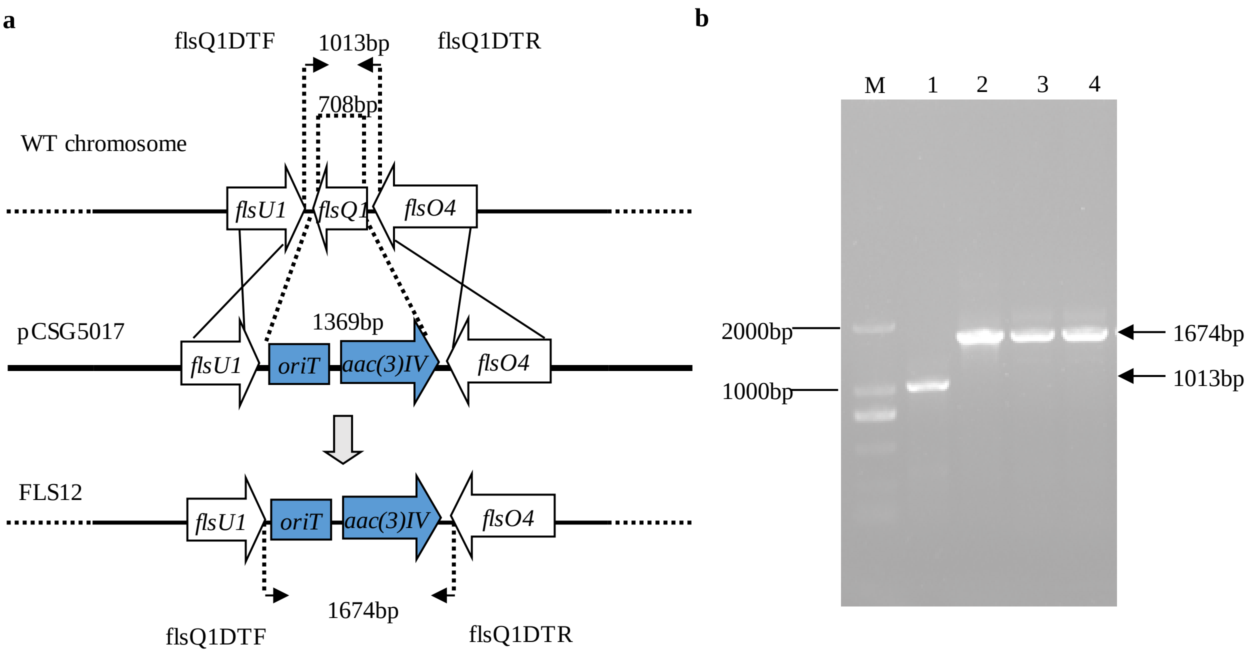

Supplementary Figure 53. Construction *flsQ1* inactivation mutant FLS12. a Description of *flsQ1* disruption. FLS12 was constructed by replacing a 708 bp internal *flsQ1* fragment with a 1369 bp DNA fragment containing *oriT* and *acc3(IV)* in pCSG5017. The location of the diagnostic PCR primers were indicated. Sizes of PCR products were also indicated: 1013 bp for the wild type strain SCSIO N160 and 1674 bp for the mutant FLS12. **b** Gel electrophoresis of PCR products. DNA templates were from: pCSG5001 (negative control, lane 1), pCSG5017 (positive control, lane 2), Δ*flsQ1* clone #1 (lane 3), Δ*flsQ1* clone #2 (lane 4) and DNA marker D2000 (GenStar, lane M).

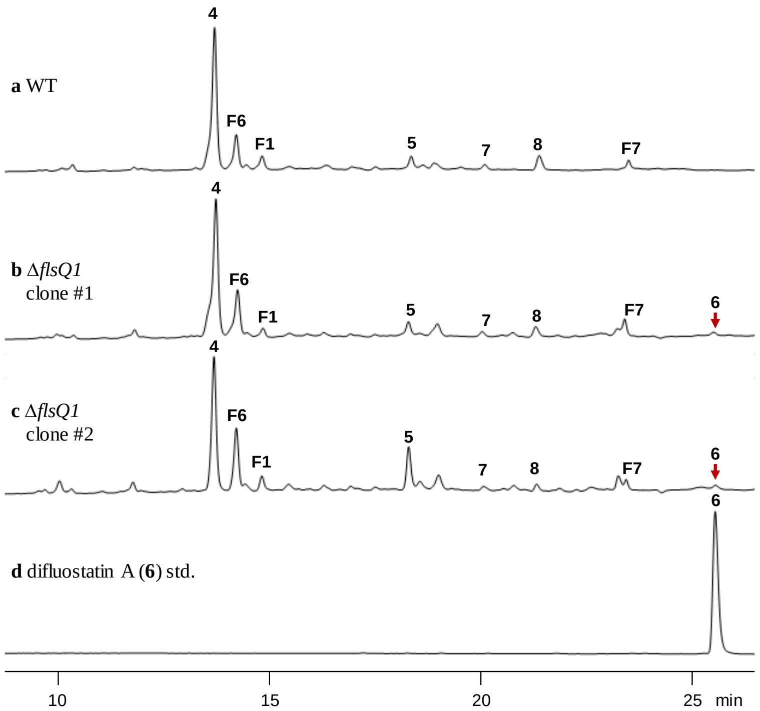

Supplementary Figure 54. HPLC analysis of the production profiles of *M. rosaria* SCSIO N160 and Δ*flsQ1* strains. a wild type (WT) strain, **b** FLS12 (Δ*flsQ1*) clone #1, **c** FLS12 (Δ*flsQ1*) clone #2, **d** difluostatin A (**6**) standard. The compound structures are shown in Supplementary Figure 2.

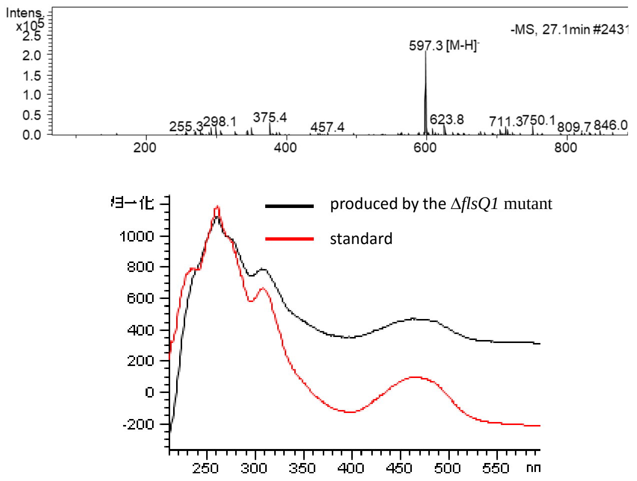

Supplementary Figure 55. Characterization of difluostatin A (**6**) produced by the FLS12 strain (Δ*flsQ1*) by LC-MS analysis and UV comparison with the standard.

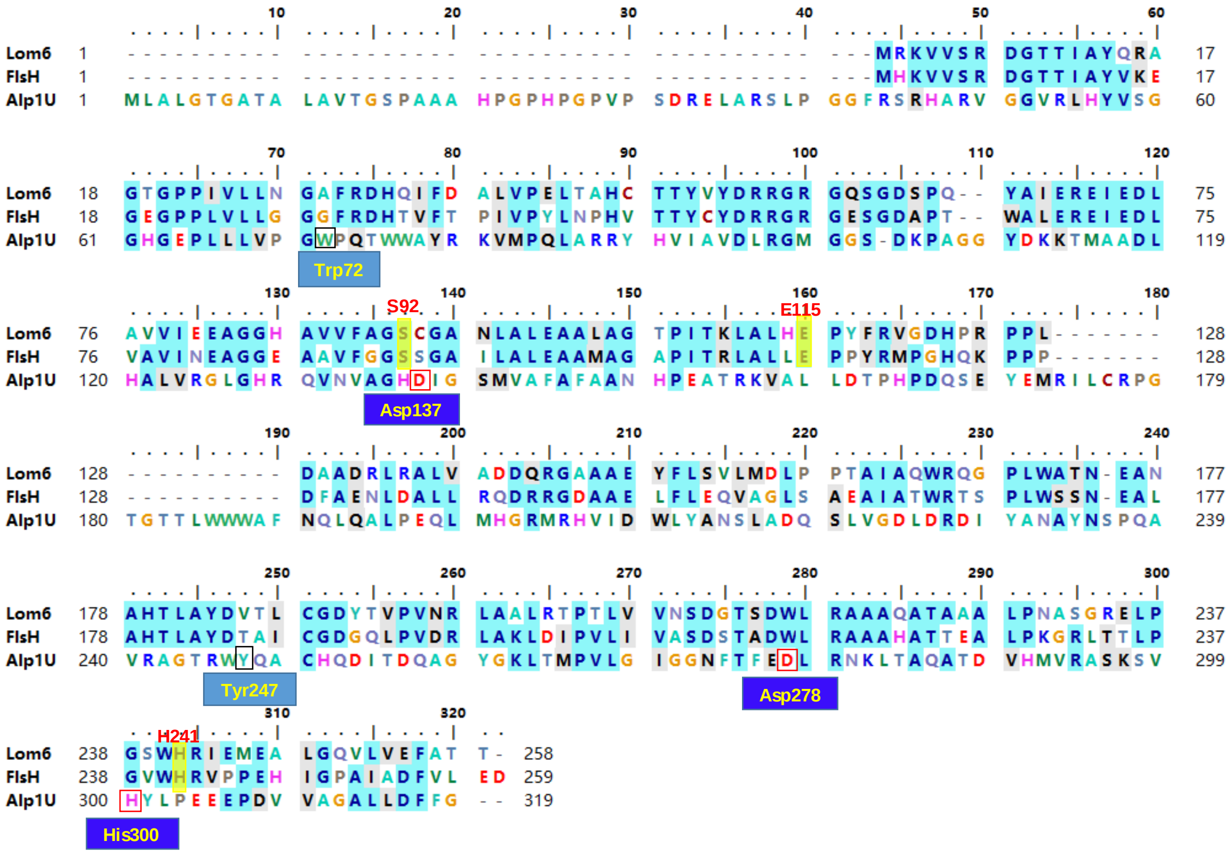

Supplementary Figure 56. Sequence alignment of Lom6, FlsH and Alp1U. The positions of catalytic triads in Alp1U (yellow letters in blue background) and FlsH, and Lom6 (red letters S92, E115 and H241) were marked.

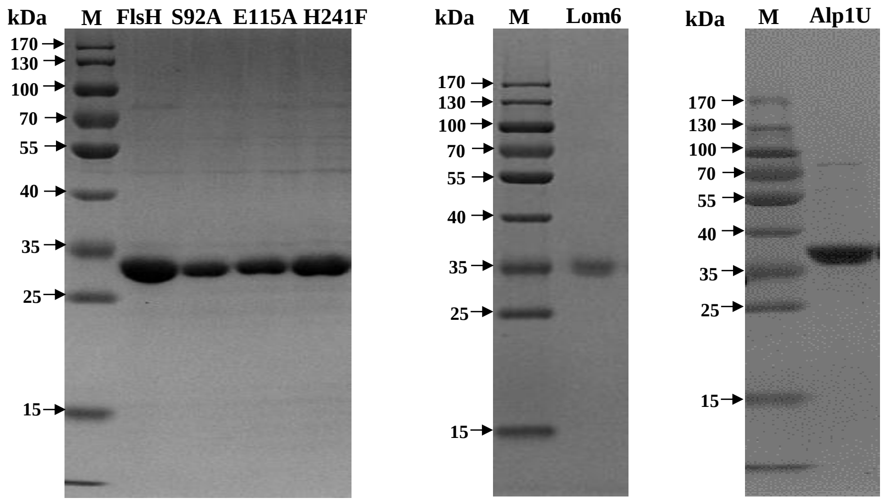

Supplementary Figure 57. The SDS-PAGE analysis of purified recombinant proteins. Lane M, protein molecular weight marker. The acrylamide percentage of SDS-PAGE gels is 12%.

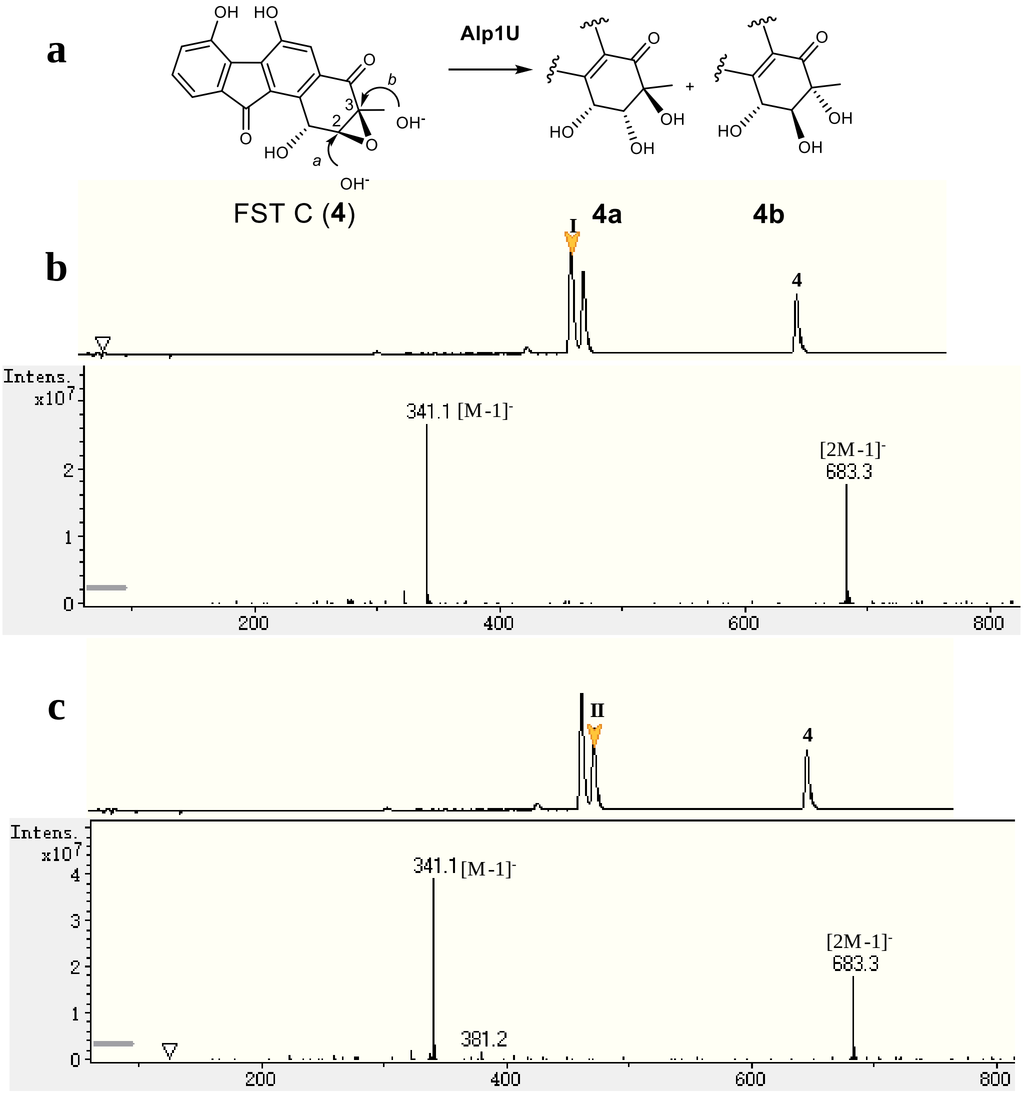

Supplementary Figure 58. LCMS analysis of Alp1U-catalyzed epoxide hydrolyzing reaction. a Scheme for Alp1U catalyzed reaction. **b** Negative mode ESI-MS data for peak **I**; **c** Negative mode ESI-MS data for peak **II**.

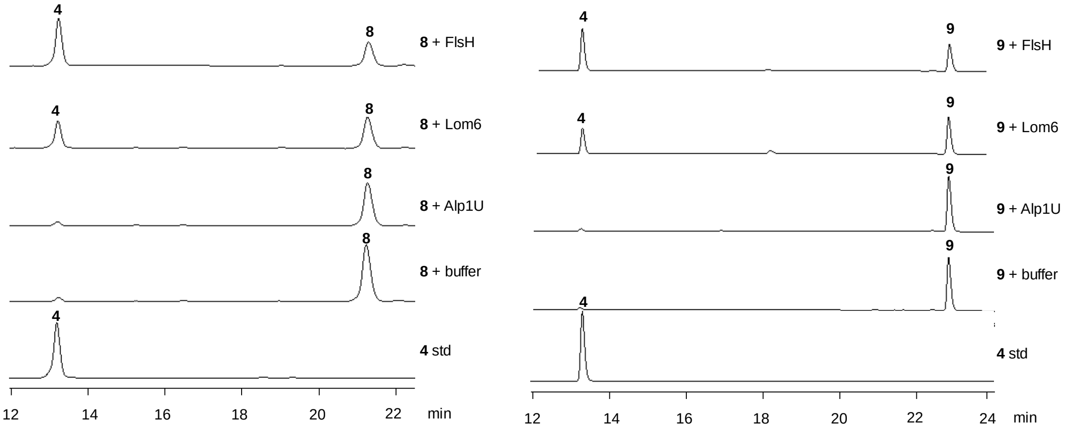

Supplementary Figure 59. HPLC analyses of enzyme assays for FlsH, Lom6 and Alp1U against acyl FSTs. Each enzyme assay was performed in 50 µL of 50 mM phosphate buffer (pH 7.0) containing 100 µM substrate (**8**, or **9**) and 10 µM enzyme (FlsH, Lom6 or Alp1U) for 30 min at 30 ºC.

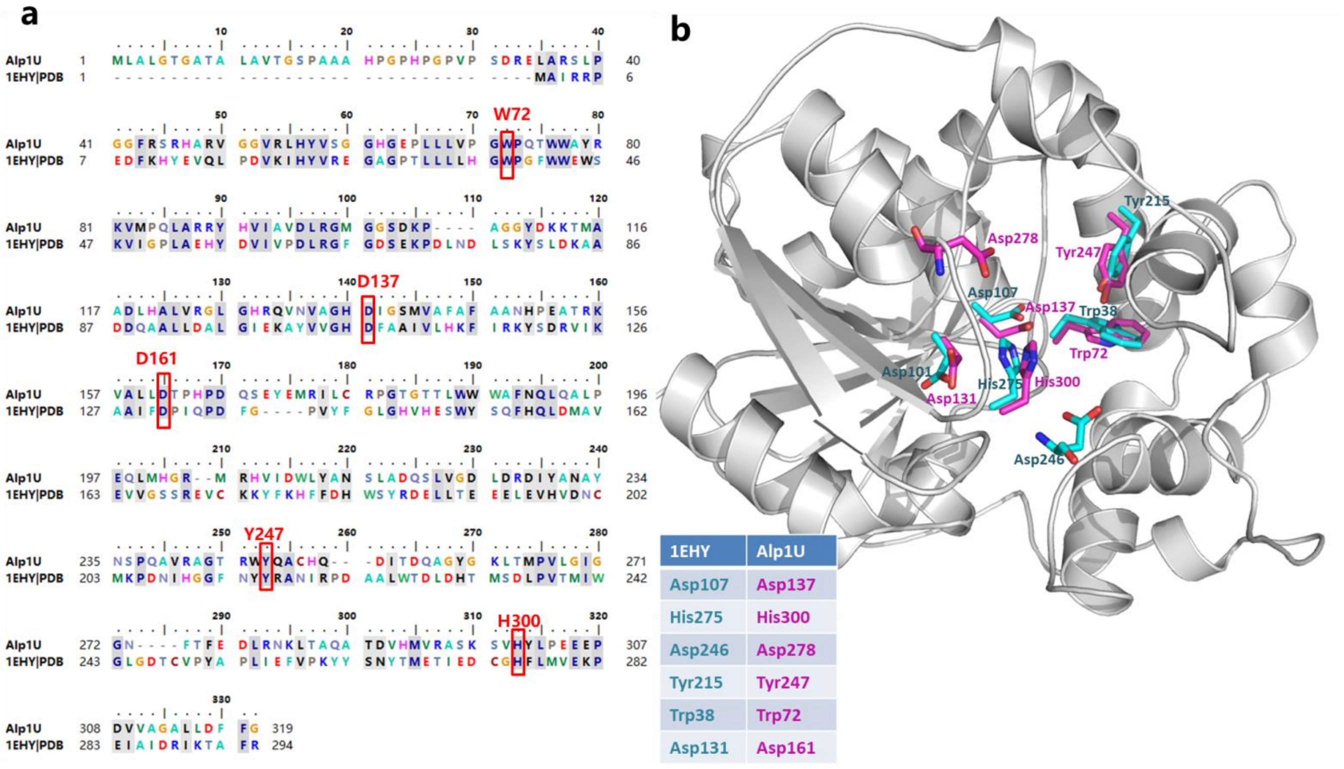

Supplementary Figure 60. Structure model of Alp1U as an epoxide hydrolase. a Sequence alignment of Alp1U with the epoxide hydrolase from *Agrobacterium radiobacter* Ad1 (PDB ID: 1EHY), which share 29% identity with Alp1U. The conserved catalytic residues (W72, D137, D161, H300 and Y247 of Alp1U) are marked in red boxes; and the identical and similar residues are highlighted in grey background. **b** The Alp1U model (shown as grey carton) was constructed on the structural basis of an epoxide hydrolase (PDB ID:1EHY), the closest homolog of Alp1U. Corresponding amino acid residues in the active pocket (purple for Alp1U and blue for 1EHY) are well overlapped, except for one amino acid (Asp278 in Alp1U and Asp246 in 1EHY).

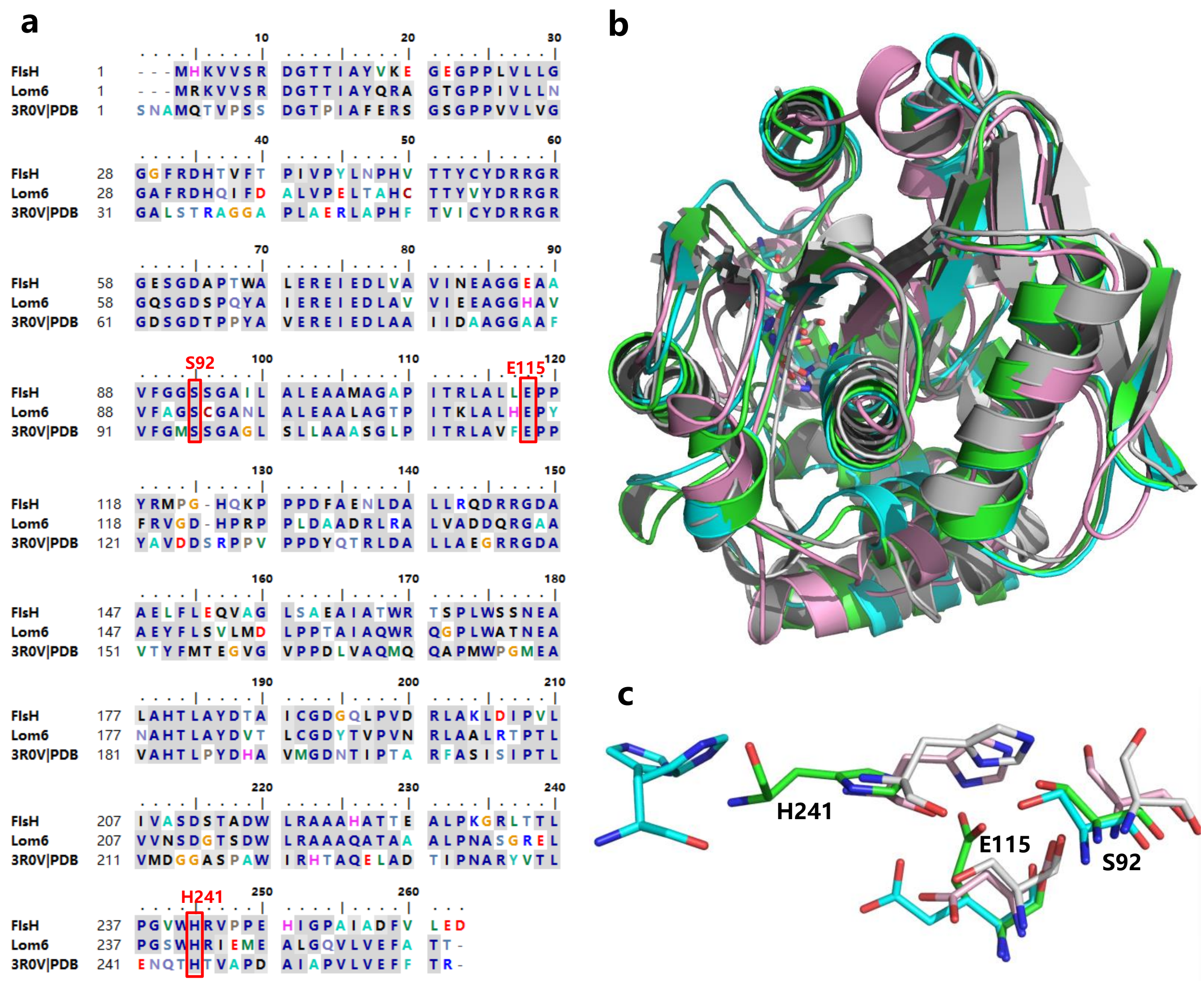

Supplementary Figure 61. Structure model of FlsH as an alpha/beta hydrolase. **a** Amino acid sequence alignment of FlsH and Lom6 with the alpha/beta-hydrolase (PDB no. 3R0V) from *Sphaerobacter thermophilus* DSM 20745, which share 45% identity for 98% covered sequences with FlsH. The three conserved catalytic residues (S92, E115, H241 of FlsH) are marked in red boxes, and the identical and similar residues are highlighted in grey background. **b** The alignment of FlsH model (colored in green) with 3 typical structures that were used as templates for FlsH homology modeling, including Est816 (PDB: 5EGN, colored in grey) from uncultured bacterium, TtEst (PDB: 4UHC, colored in pink) from *Thermogutta terrifontis* DSM 26237, and MhpC (PDB: 3R0V, colored in cyan) from *Sphaerobacter thermophilus* str. DSM 20745. **c** The predicted catalytic triad of FlsH (green sticks) aligned with those of 3R0V(cyan sticks), 5EGN2 (grey sticks) and 4UHC (pink sticks).

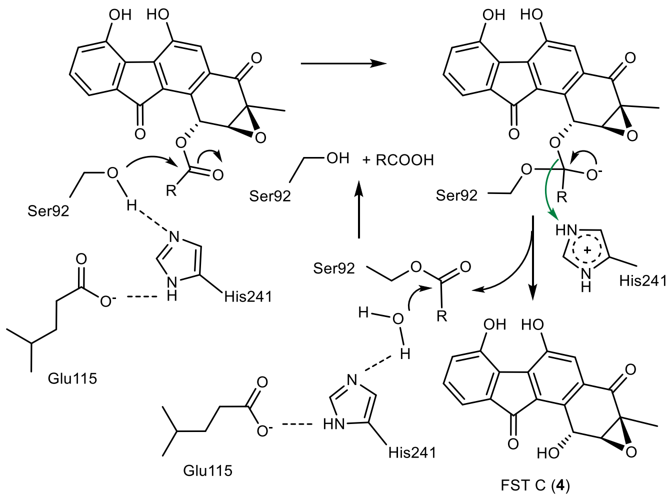

Supplementary Figure 62. The proposed mechanism of FlsH catalyzed deacylation. The abstraction of a proton from Ser92 by His241 helps a nucleophilic attack of Ser92 on the C13 carbonyl carbon of acyl FSTs to form a tetrahedral intermediate. This attack should be only efficiently occurred by the deprotonation of His241, which is achieved by stabilization of His241 via a hydrogen bond with Glu115. The resulting tetrahedral intermediate is unstable and decomposed to FST C (**4**) and Ser92-acyl enzyme. Subsequently, a nucleophilic attack of a water molecule on the Ser92-acyl enzyme occurs to generate a second tetrahedral intermediate, which decomposes into the native enzyme and a carboxylic acid to complete the catalytic cycle.

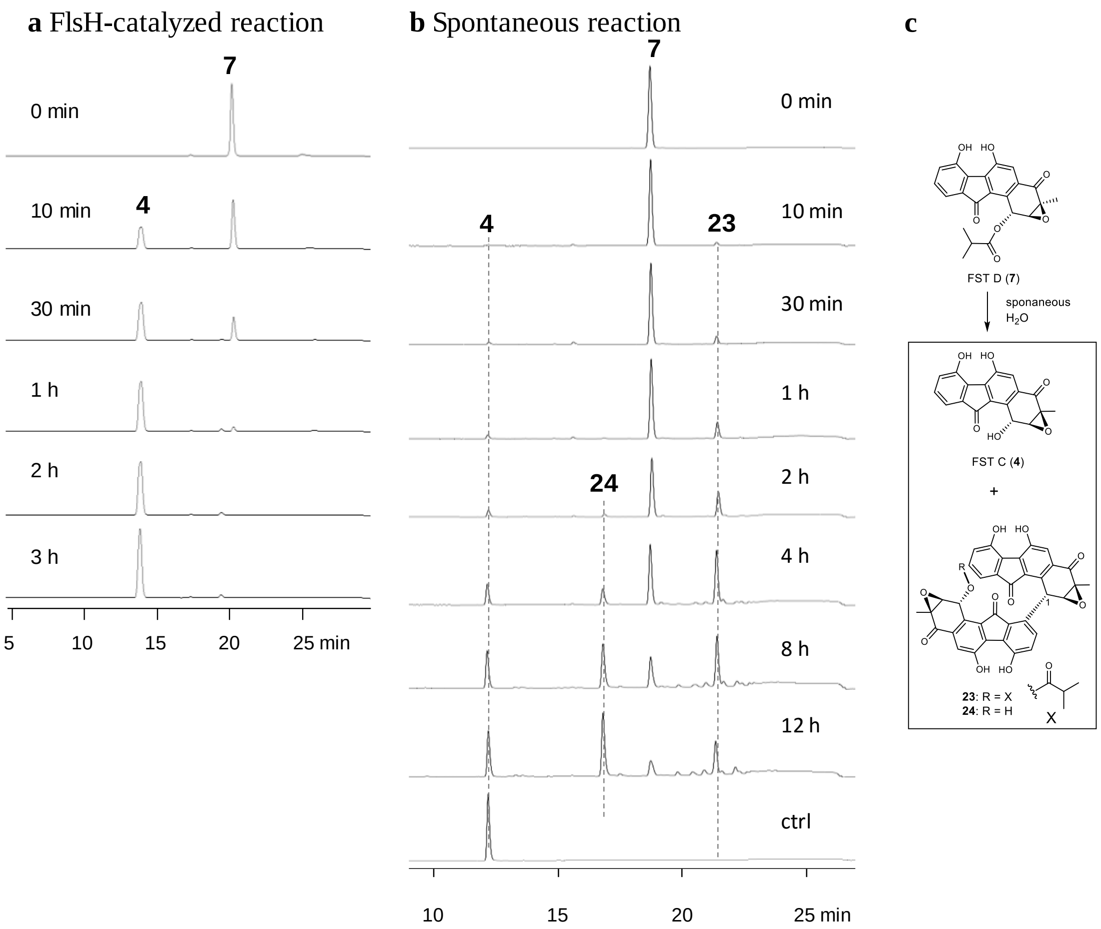

Supplementary Figure 63. Time course analyses of FlsH-catalyzed and spontaneous deacylation reactions. a FlsH-catalyzed reactions on FST D (**7**). Enzyme assays were performed in 50 μL phosphate buffer (50 mM, pH8.0) containing 100 μM FST D (**7**), 10 μM FlsH and incubated at 30 °C. **b** Incubation of FST D (**7**) in H_2O at 30 °C. **c** A scheme for spontaneous reaction of **7** in H_2O.

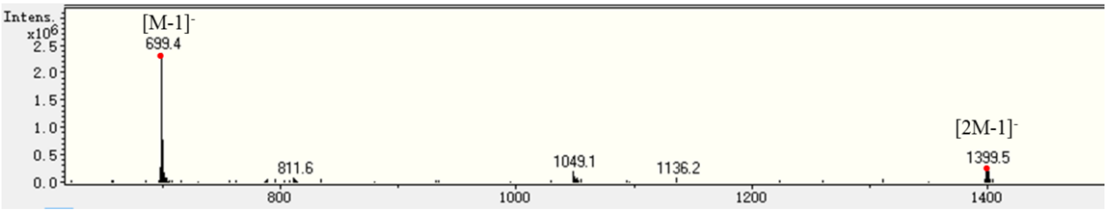

Supplementary Figure 64. Negative mode ESI-MS data for difluostatin E (**23**).

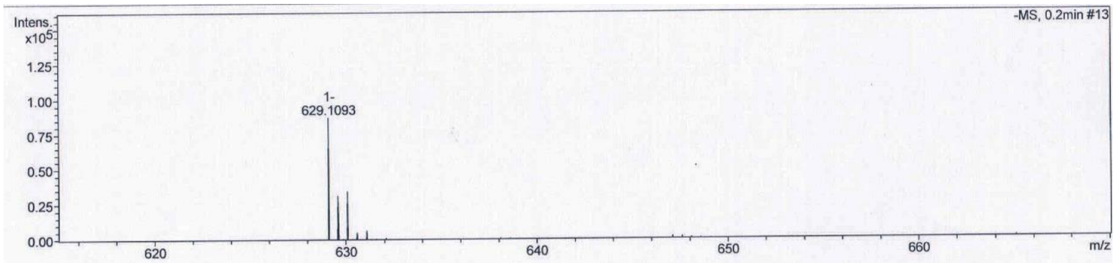

Meas. m/z	#	Ion Formula	Score	m/z	err [ppm]	err [mDa]	mSigma	rdb	e⁻ Conf	N-Rule
629.109268	1	C36H21O11	100.00	629.108935	0.5	0.3	12.9	26.5	even	ok
665.087782	1	C36H22ClO11	100.00	665.085613	-3.3	-2.2	35.5	25.5	even	ok

Supplementary Figure 65. HRESIMS spectrum of difluostatin F (**24**).

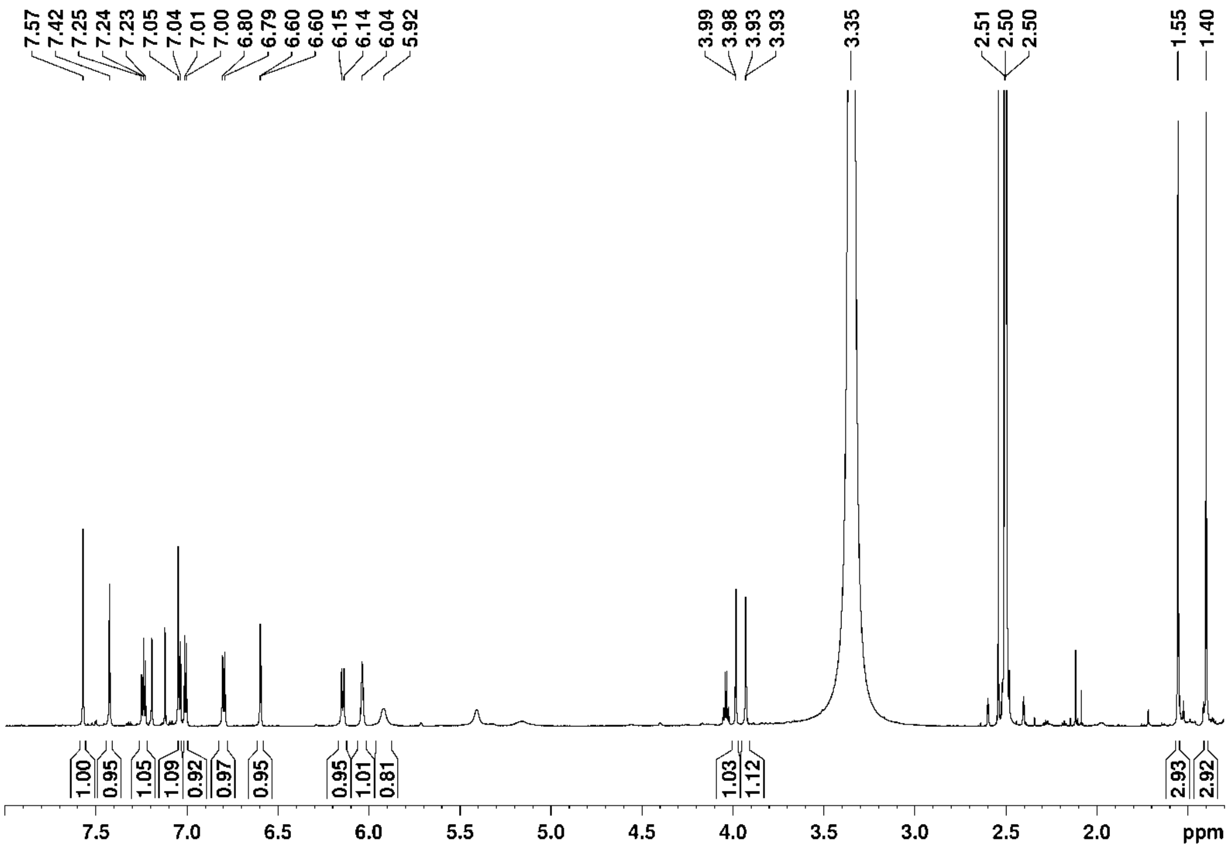

Supplementary Figure 66. The ^{1}H NMR spectrum of difluostatin F (**24**) in DMSO-d_6.

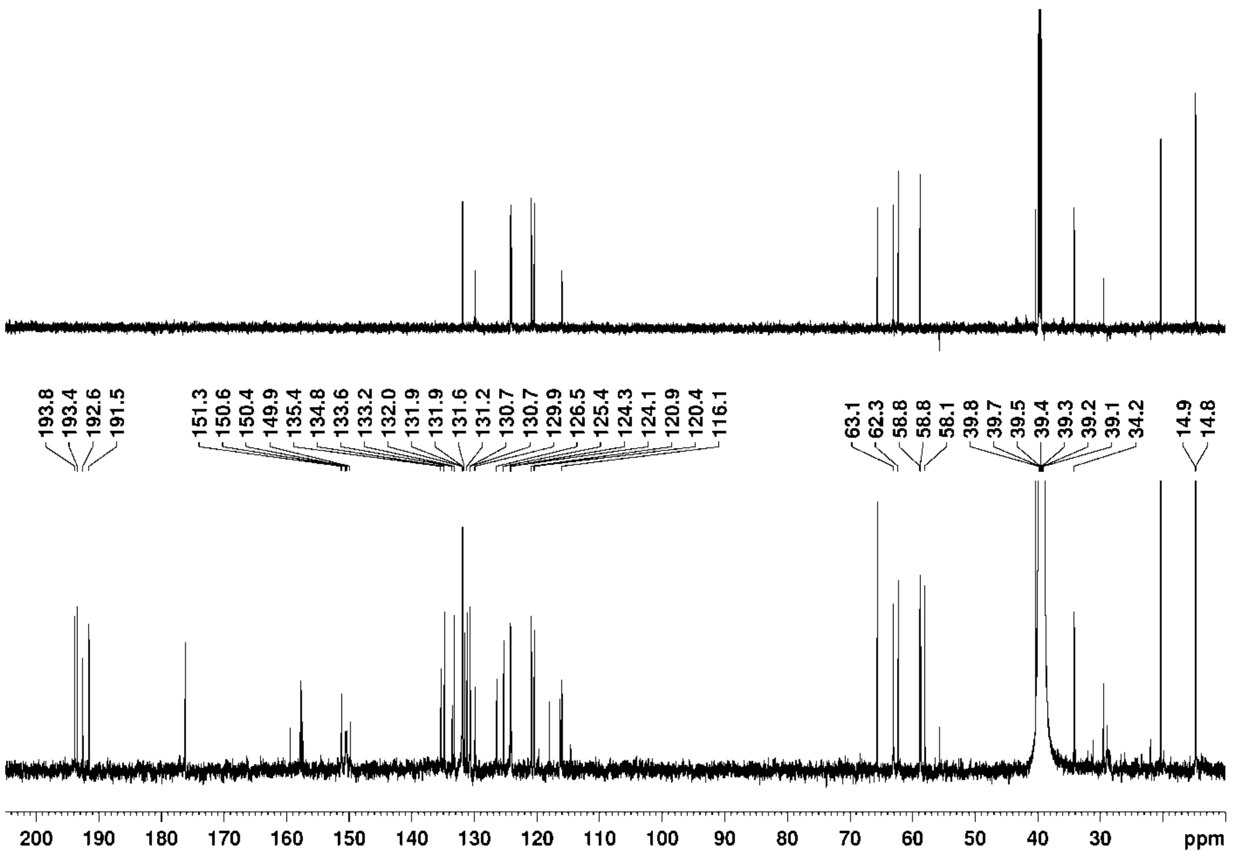

Supplementary Figure 67. The ^{13}C and DEPT 135 NMR spectrum of difluostatin F (**24**) in DMSO-d_6.

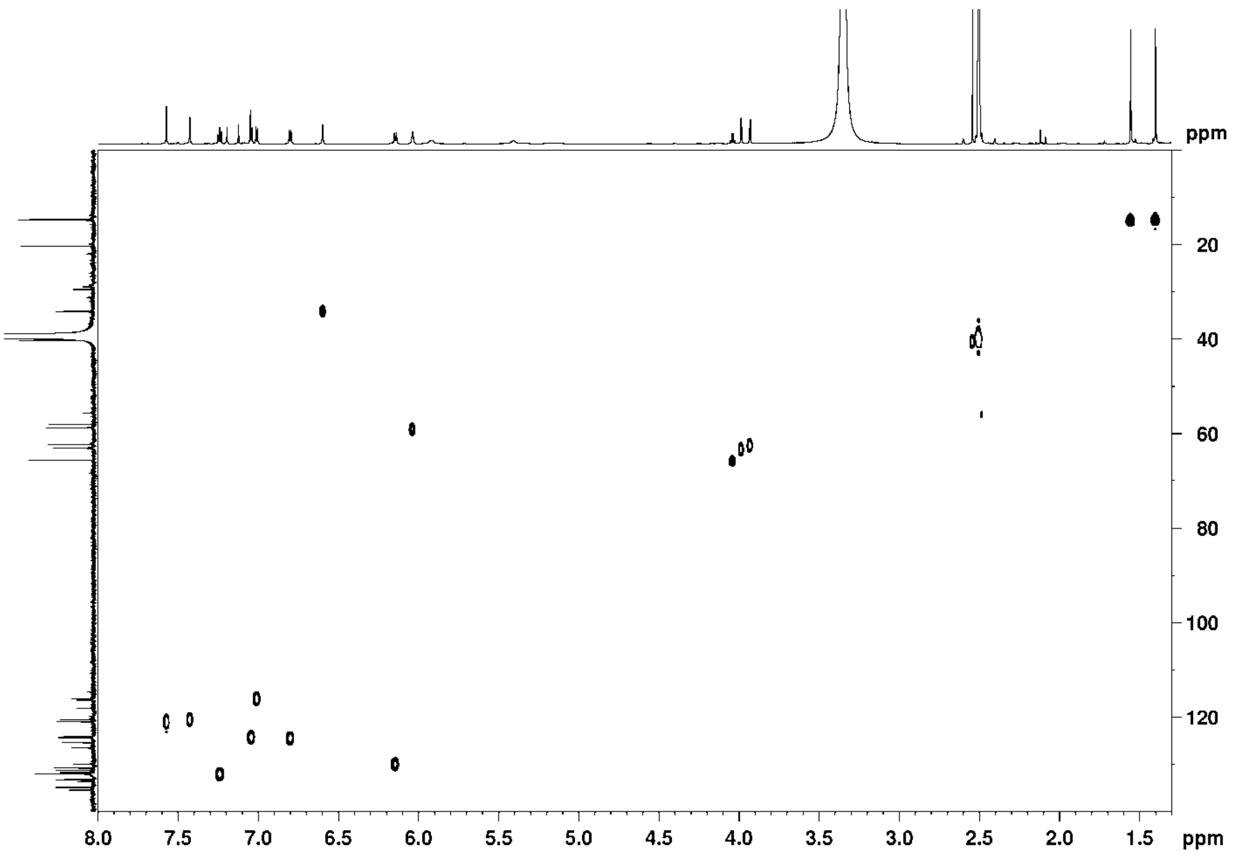

Supplementary Figure 68. The HSQC spectrum of difluostatin F (**24**) in DMSO-d_6.

a

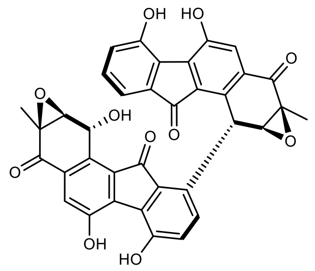

b

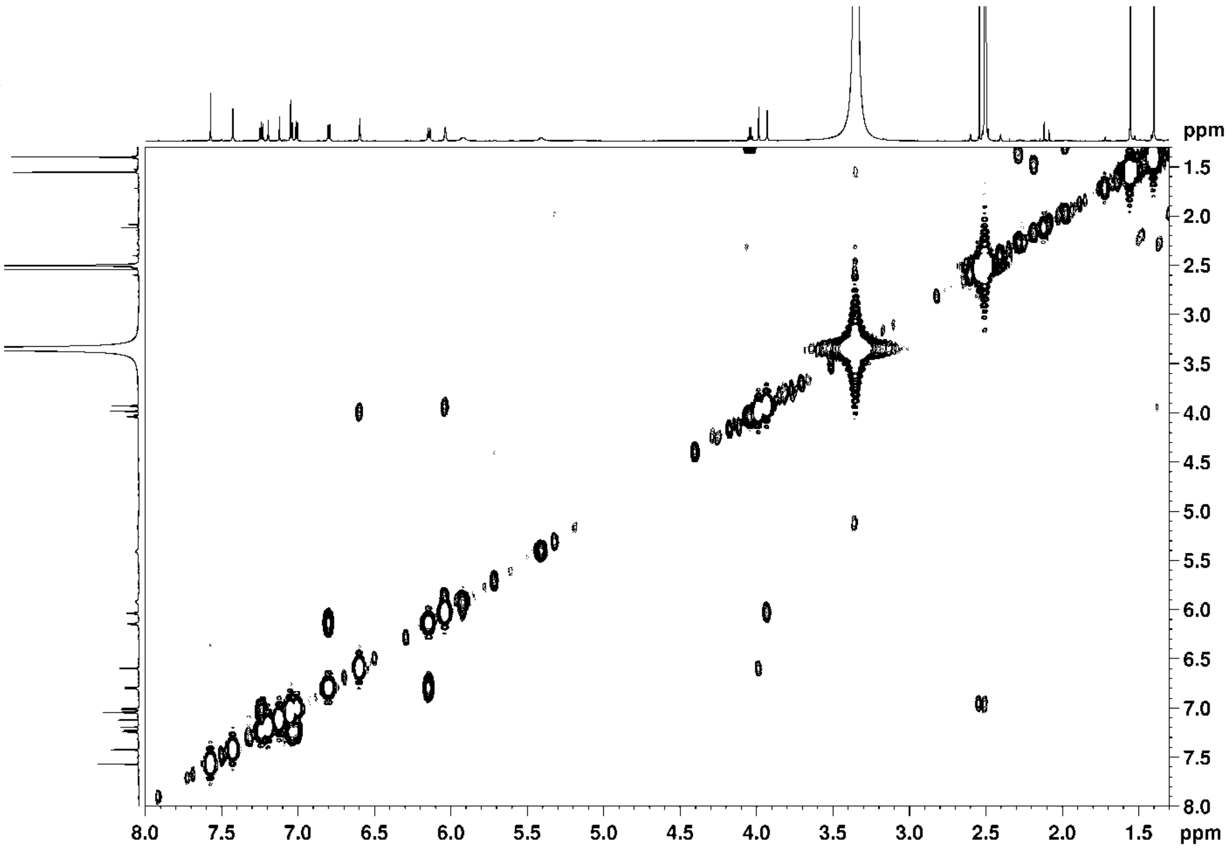

Supplementary Figure 69. The COSY spectrum of difluostatin F (**24**) in DMSO-d_6. **a** COSY correlations are indicated by boldface bonds. **b** The COSY spectrum.

a

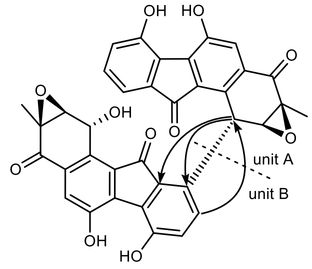

difluostatin F (**24**)

b

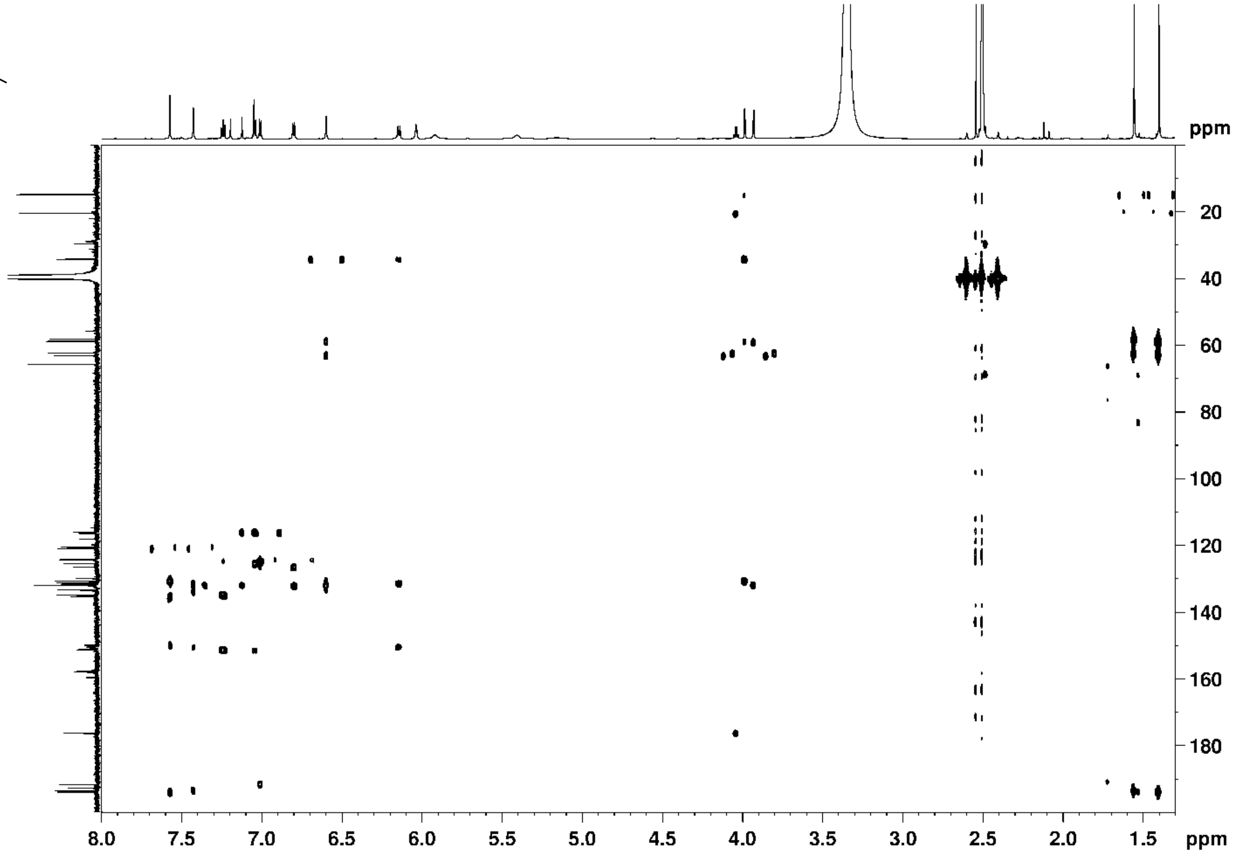

Supplementary Figure 70. The HMBC spectrum of difluostatin F (**24**) in DMSO-d_6. **a** Selected key HMBC correlations are indicated by the curved arrows. **b** The HMBC spectrum.

a

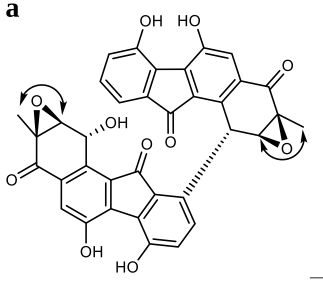

difluostatin F (**24**)

b

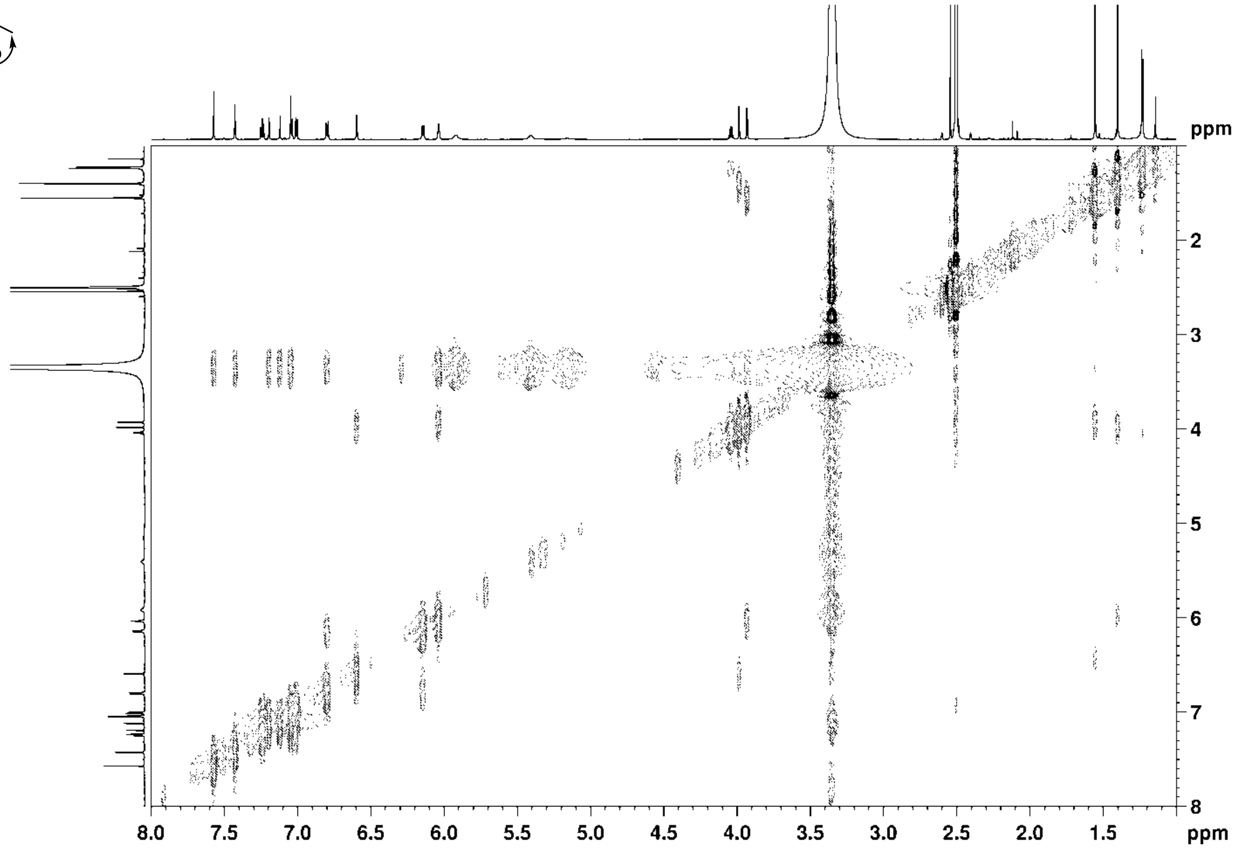

Supplementary Figure 71. The NOESY spectrum of difluostatin F (**24**) in DMSO-d_6. **a** Selected key NOESY correlations are indicated by the curved, double-headed arrows. **b** The NOESY spectrum.

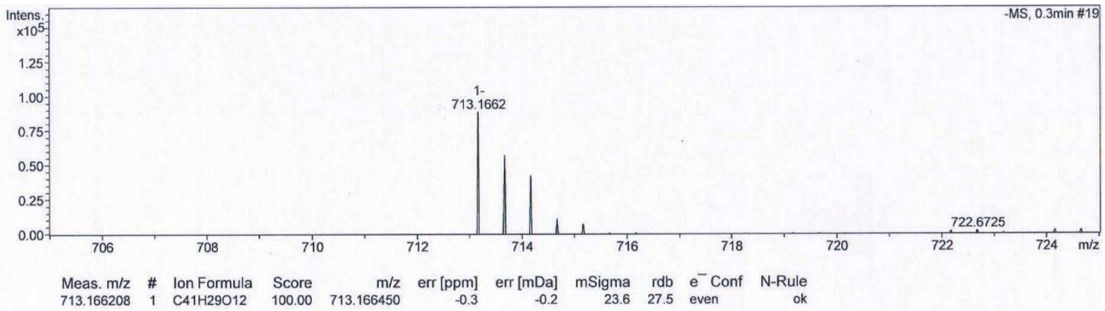

Supplementary Figure 72. HRESIMS spectrum of difluostatin G (**25**).

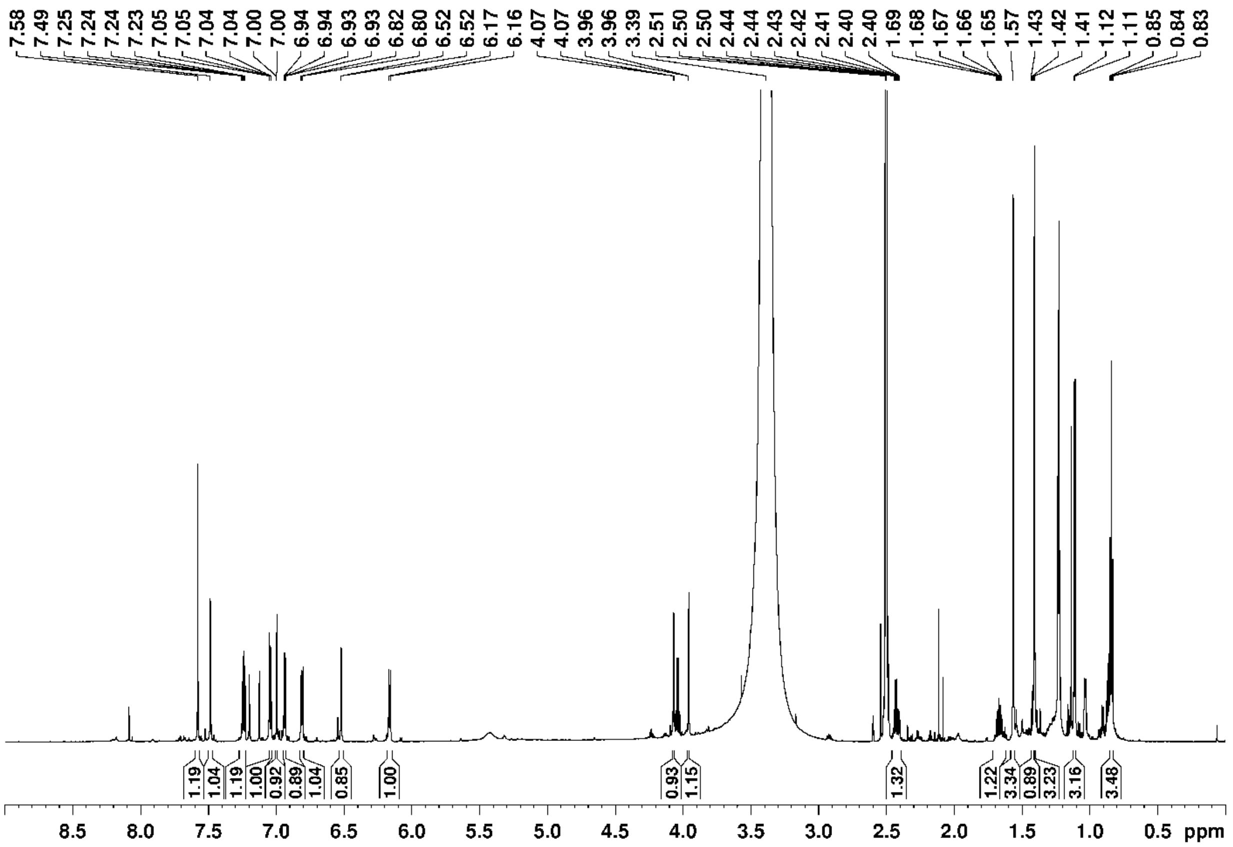

Supplementary Figure 73. The ^{1}H NMR spectrum of difluostatin G (**25**) in DMSO-d_6.

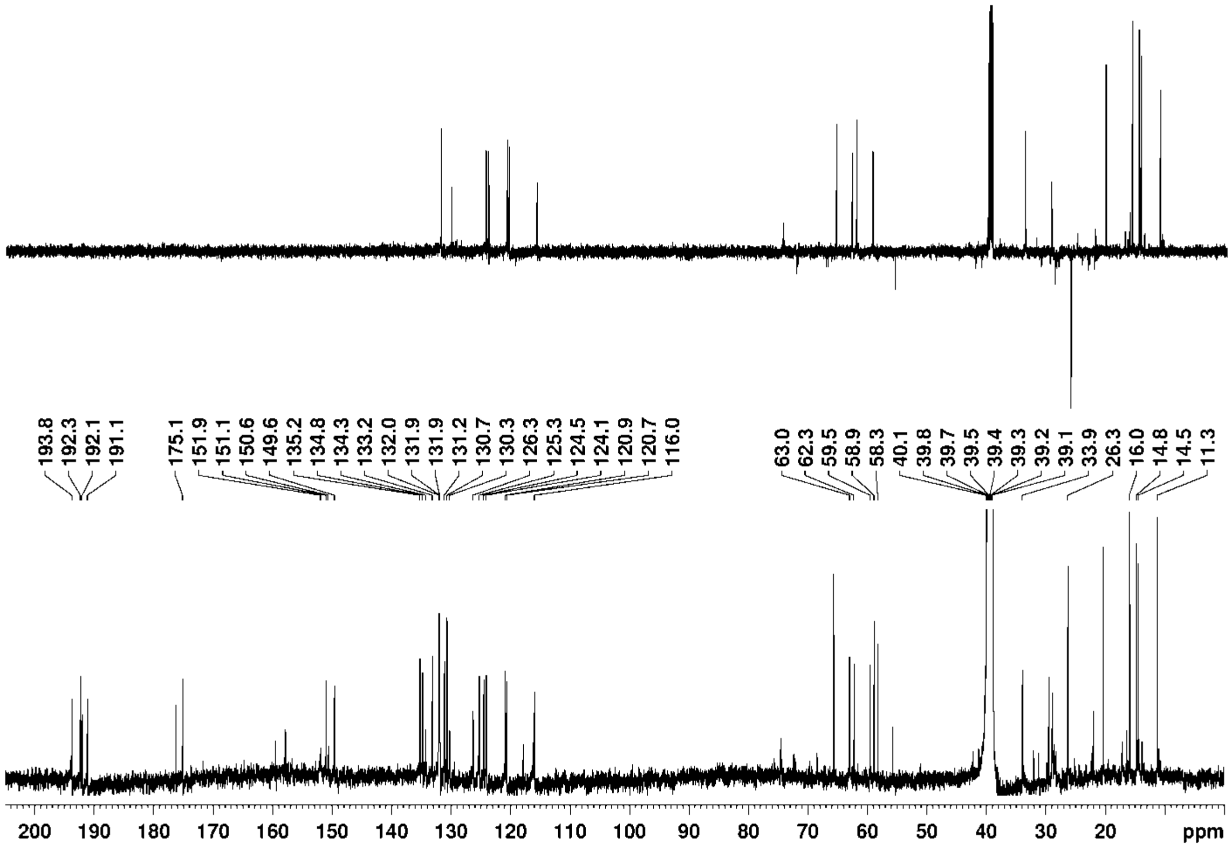

Supplementary Figure 74. The ^{13}C and DEPT 135 NMR spectrum of difluostatin G (**25**) in DMSO-d_6.

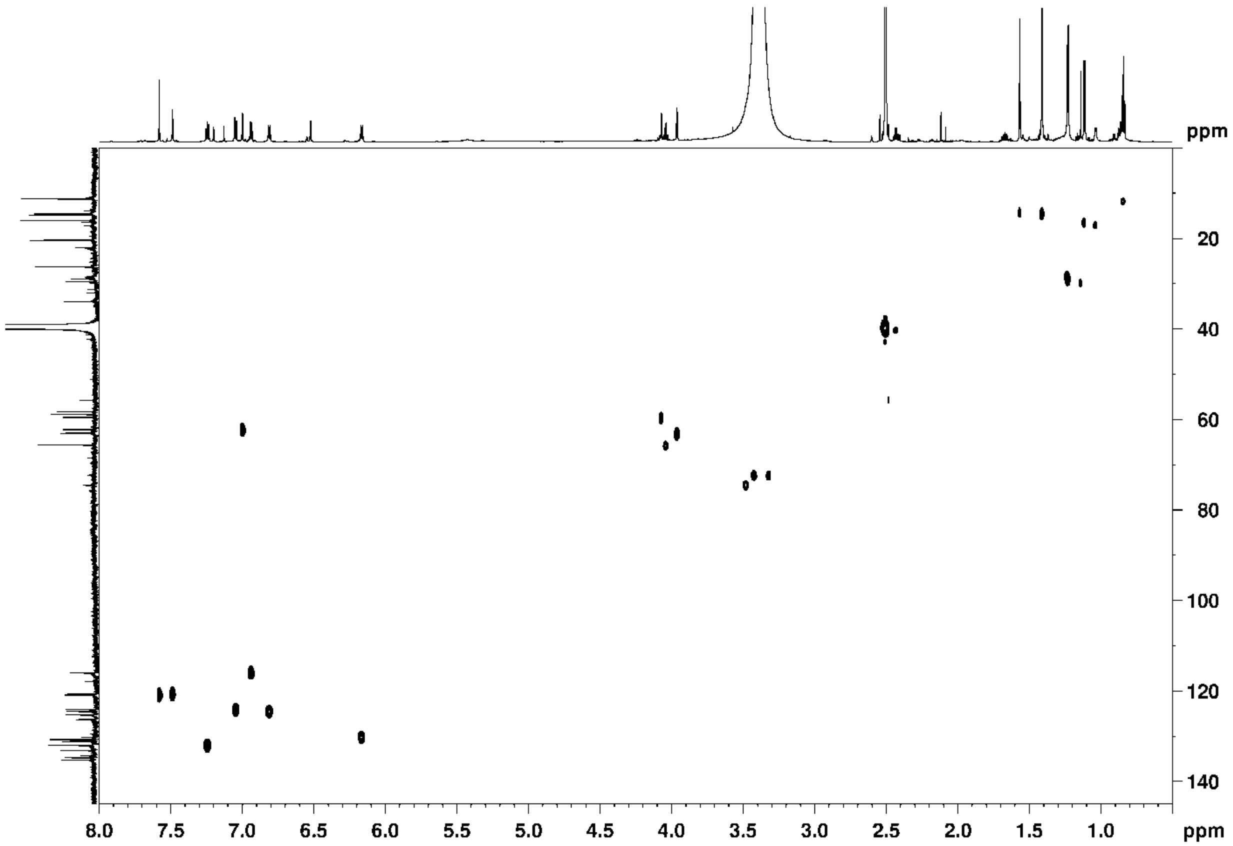

Supplementary Figure 75. The HSQC NMR spectrum of difluostatin G (**25**) in DMSO-d_6.

a

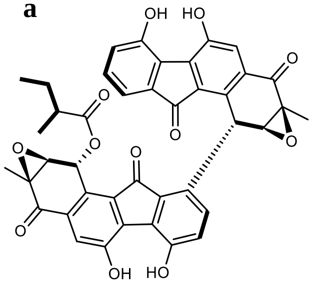

difluostatin G (**25**)

b

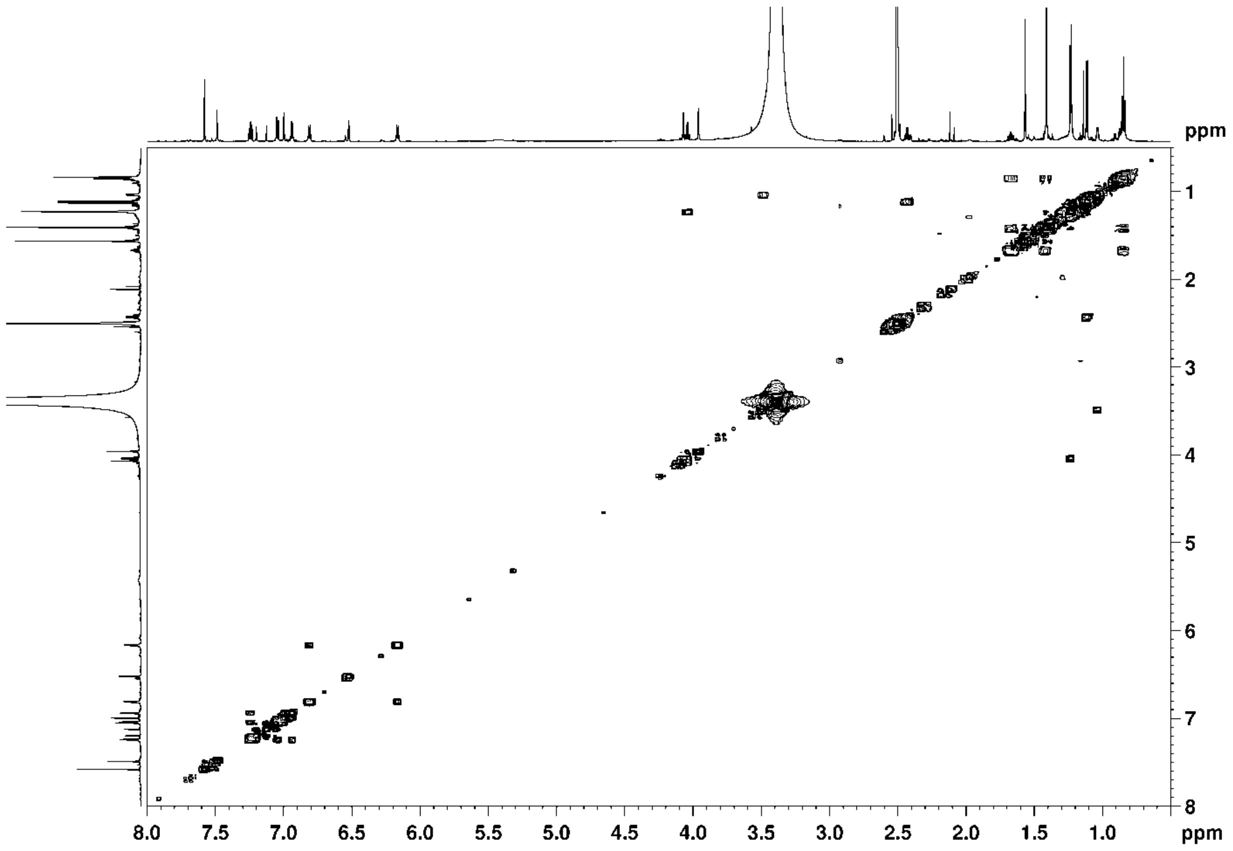

Supplementary Figure 76. The COSY spectrum of difluostatin G (**25**) in DMSO-d_6. **a** COSY correlations are indicated by boldface bonds. **b** The COSY spectrum.

a

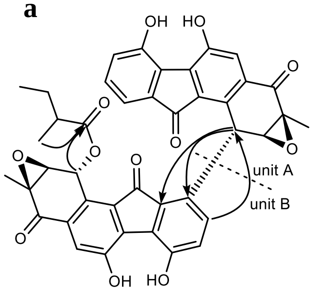

difluostatin G (**25**)

b

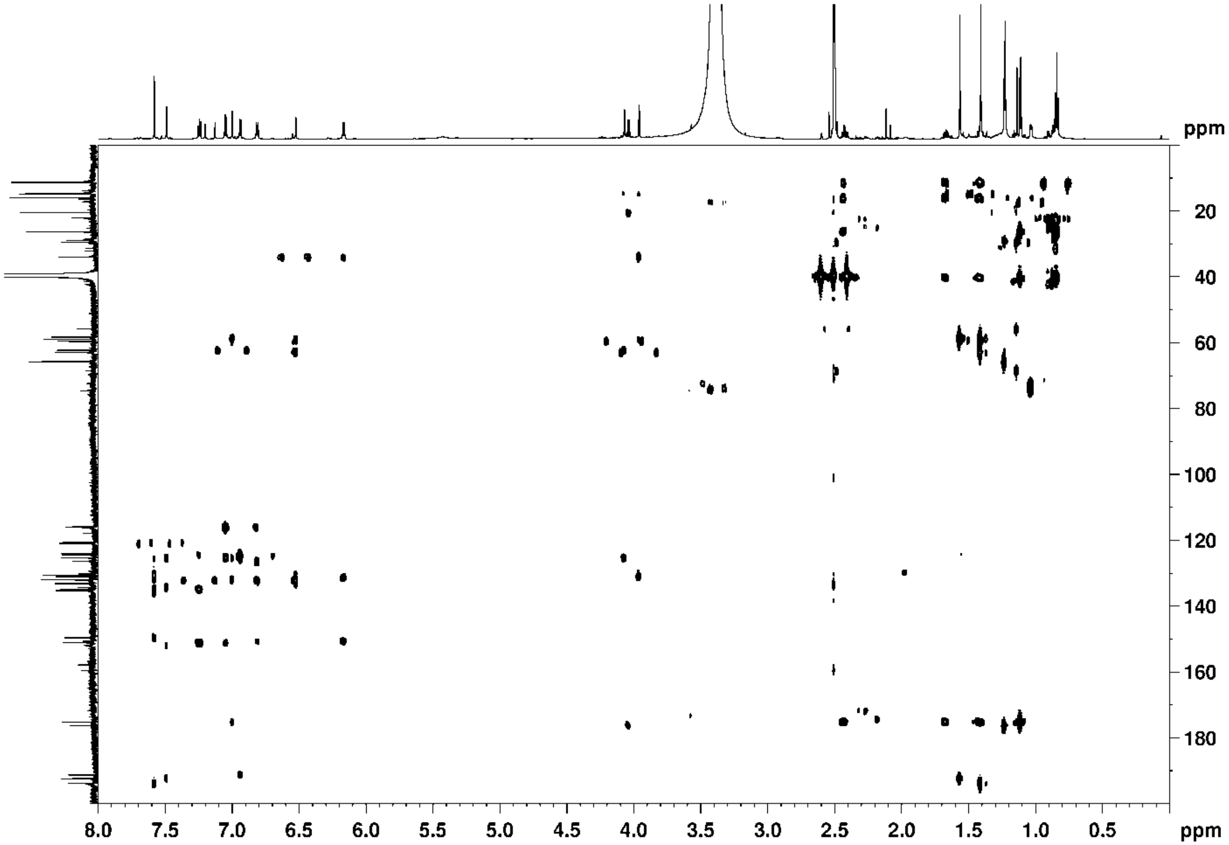

Supplementary Figure 77. The HMBC spectrum of difluostatin G (**25**) in DMSO-d_6. **a** Selected key HMBC correlations are indicated by the curved arrows. **b** The HMBC spectrum.

a

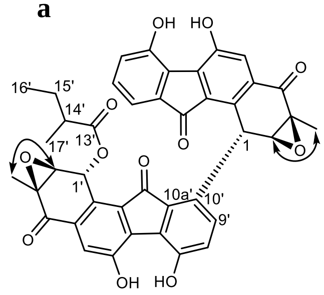

difluostatin G (**25**)

b

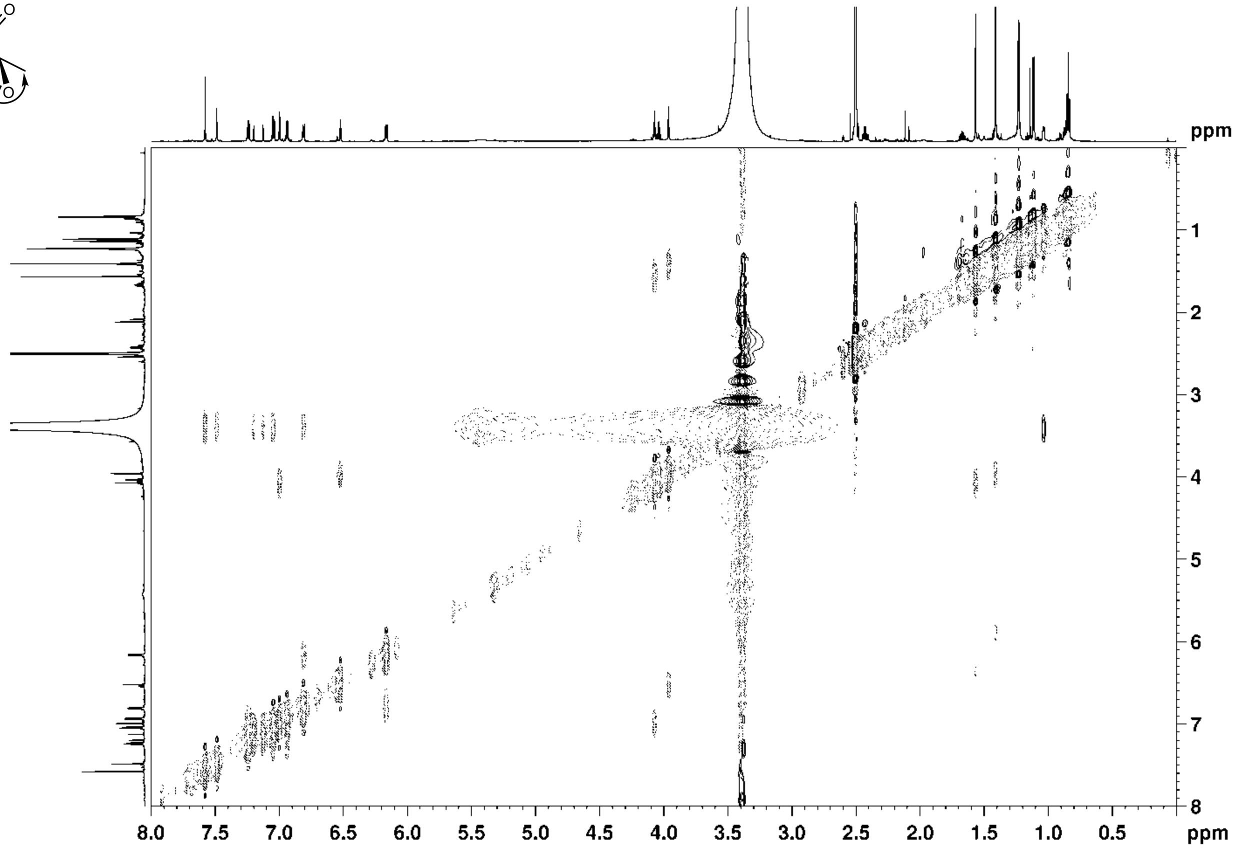

Supplementary Figure 78. The NOESY spectrum of difluostatin G (**25**) in DMSO-d_6. **a** Selected key NOESY correlations are indicated by the curved, double-headed arrows. **b** The NOESY spectrum.

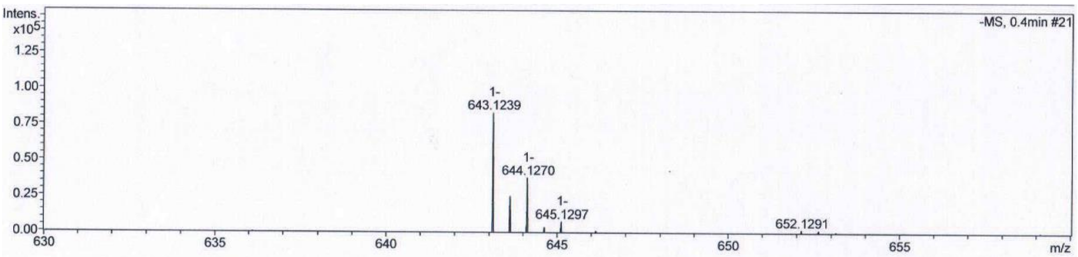

Meas. m/z	#	Ion Formula	Score	m/z	err [ppm]	err [mDa]	mSigma	rdb	e⁻ Conf	N-Rule
643.123886	1	C37H23O11	100.00	643.124585	1.1	0.7	25.2	26.5	even	ok

Supplementary Figure 79. HRESIMS spectrum of difluostatin H (**26**).

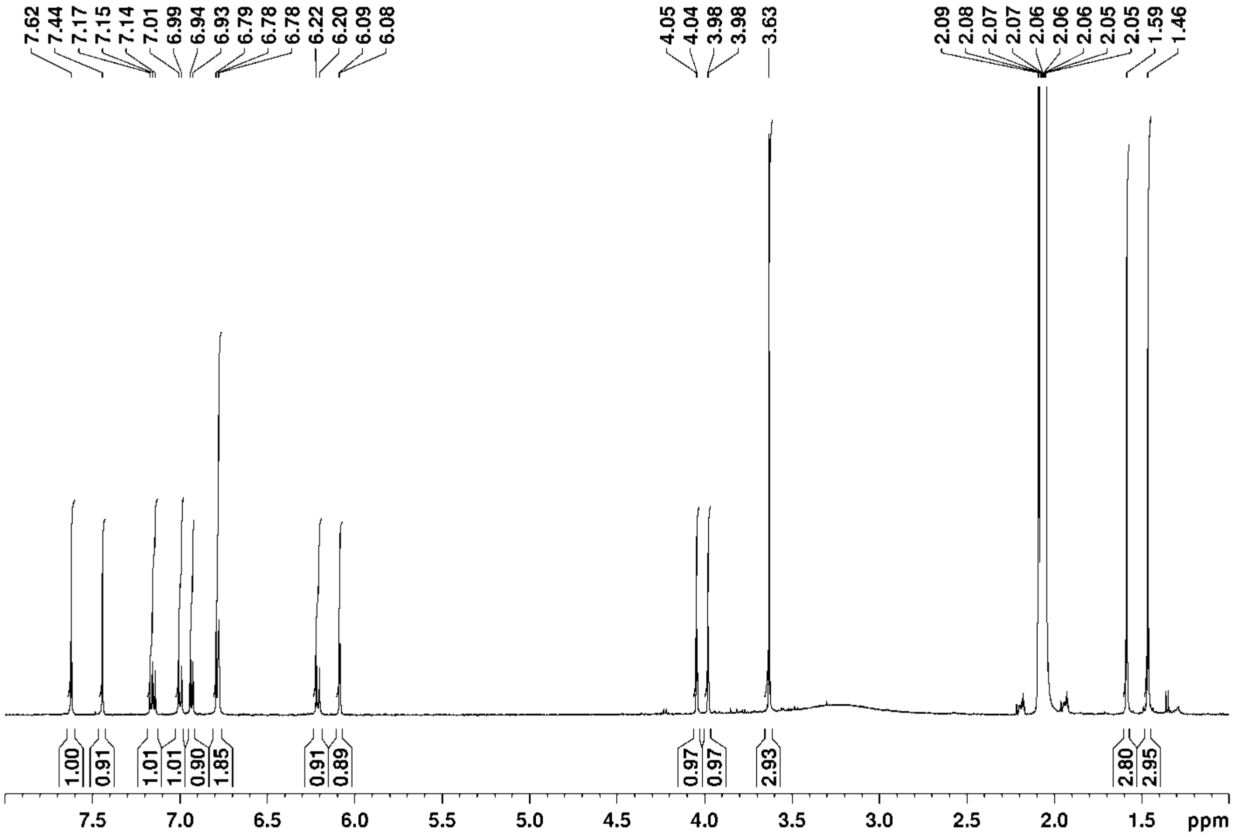

Supplementary Figure 80. The ^{1}H NMR spectrum of difluostatin H (**26**) in acetone-d_6.

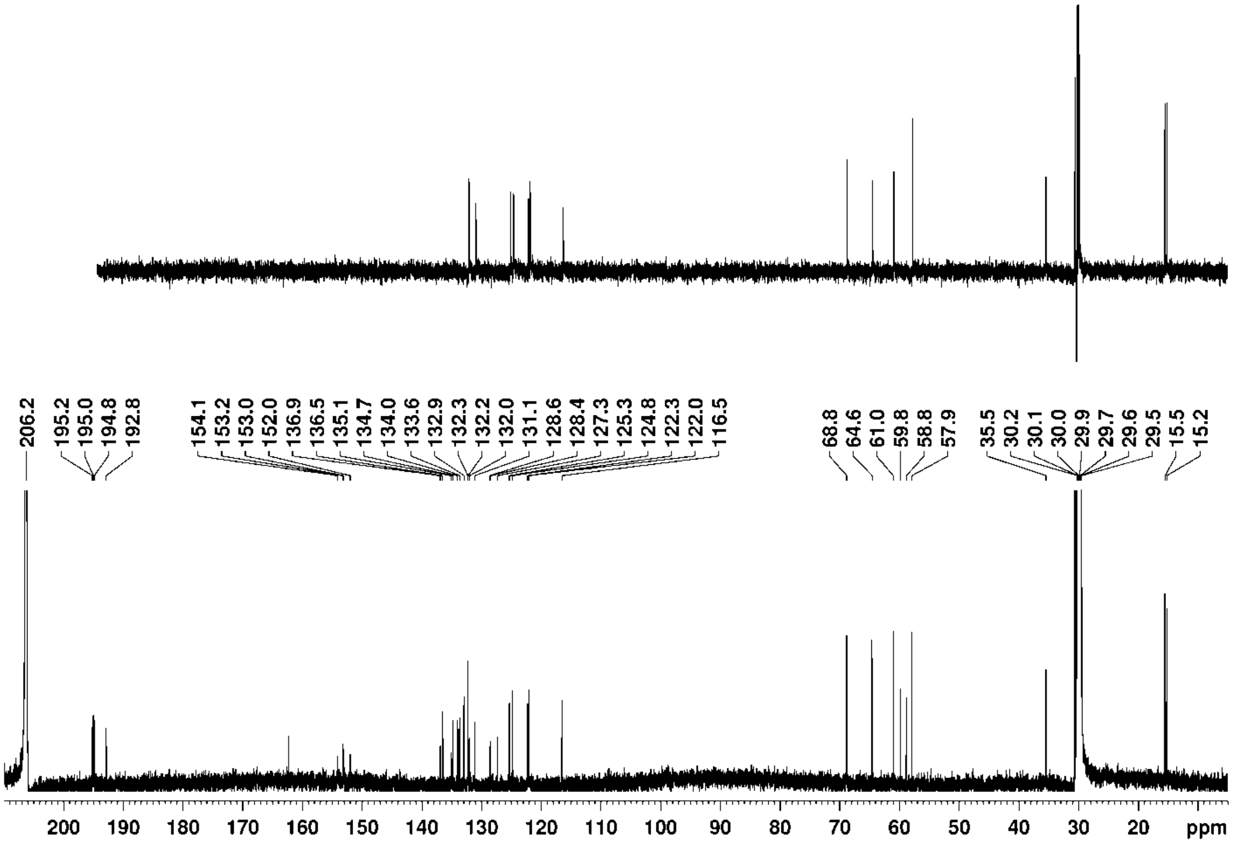

Supplementary Figure 81. The ^{13}C and DEPT 135 NMR spectrum of difluostatin H (**26**) in acetone-d_6.

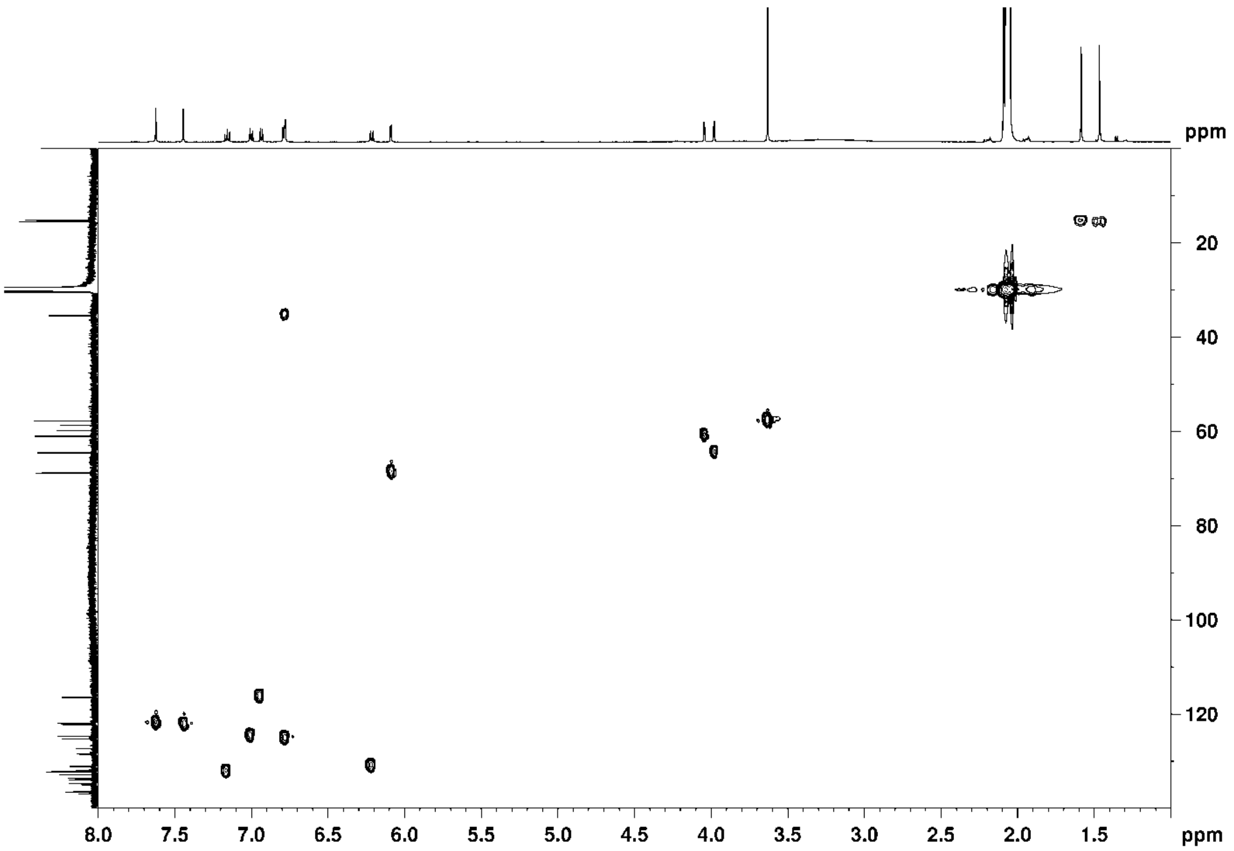

Supplementary Figure 82. The HSQC spectrum of difluostatin H (**26**) in acetone-d_6.

a

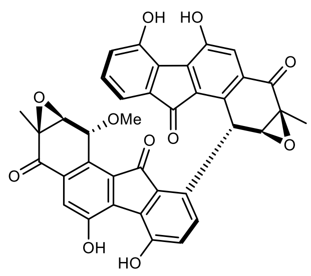

difluostatin H (**26**)

b

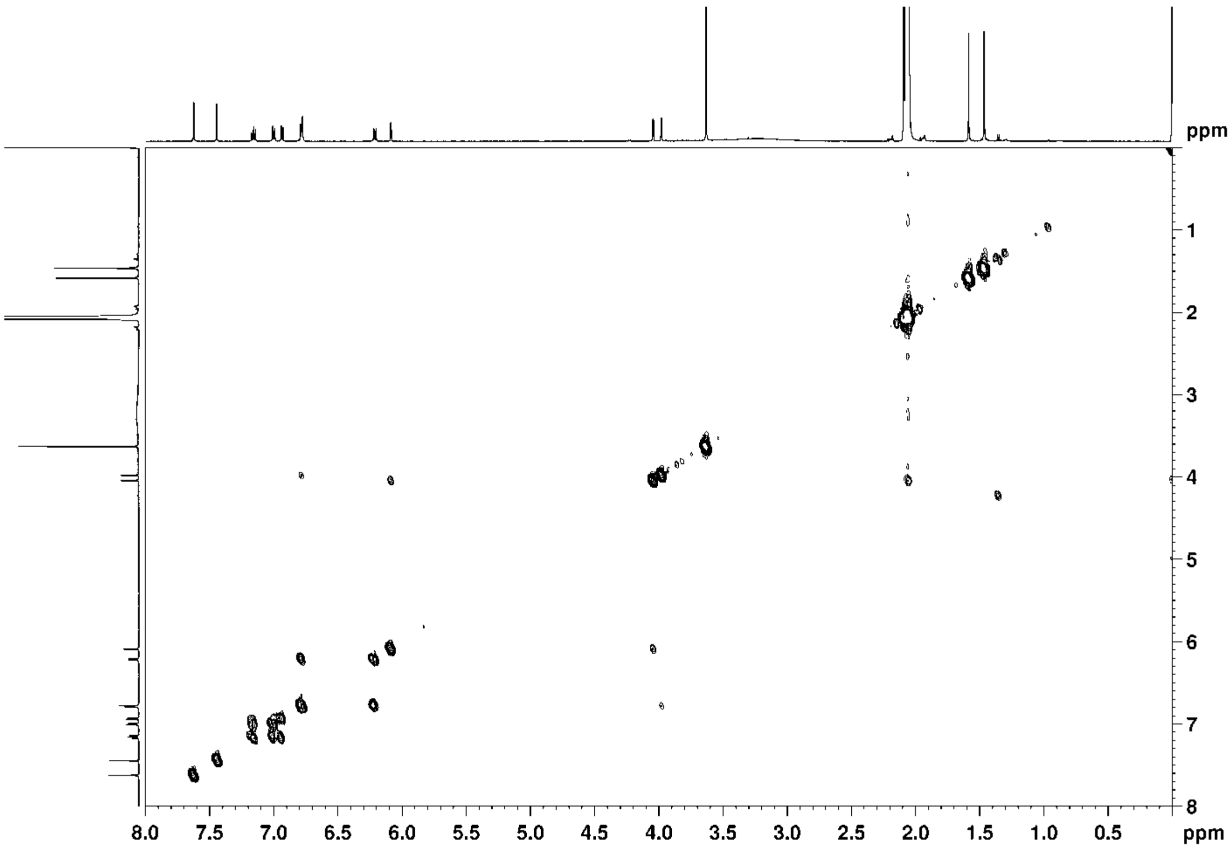

Supplementary Figure 83. The COSY spectrum of difluostatin H (**26**) in acetone-d_6. **a** COSY correlations are indicated by boldface bonds. **b** The COSY spectrum.

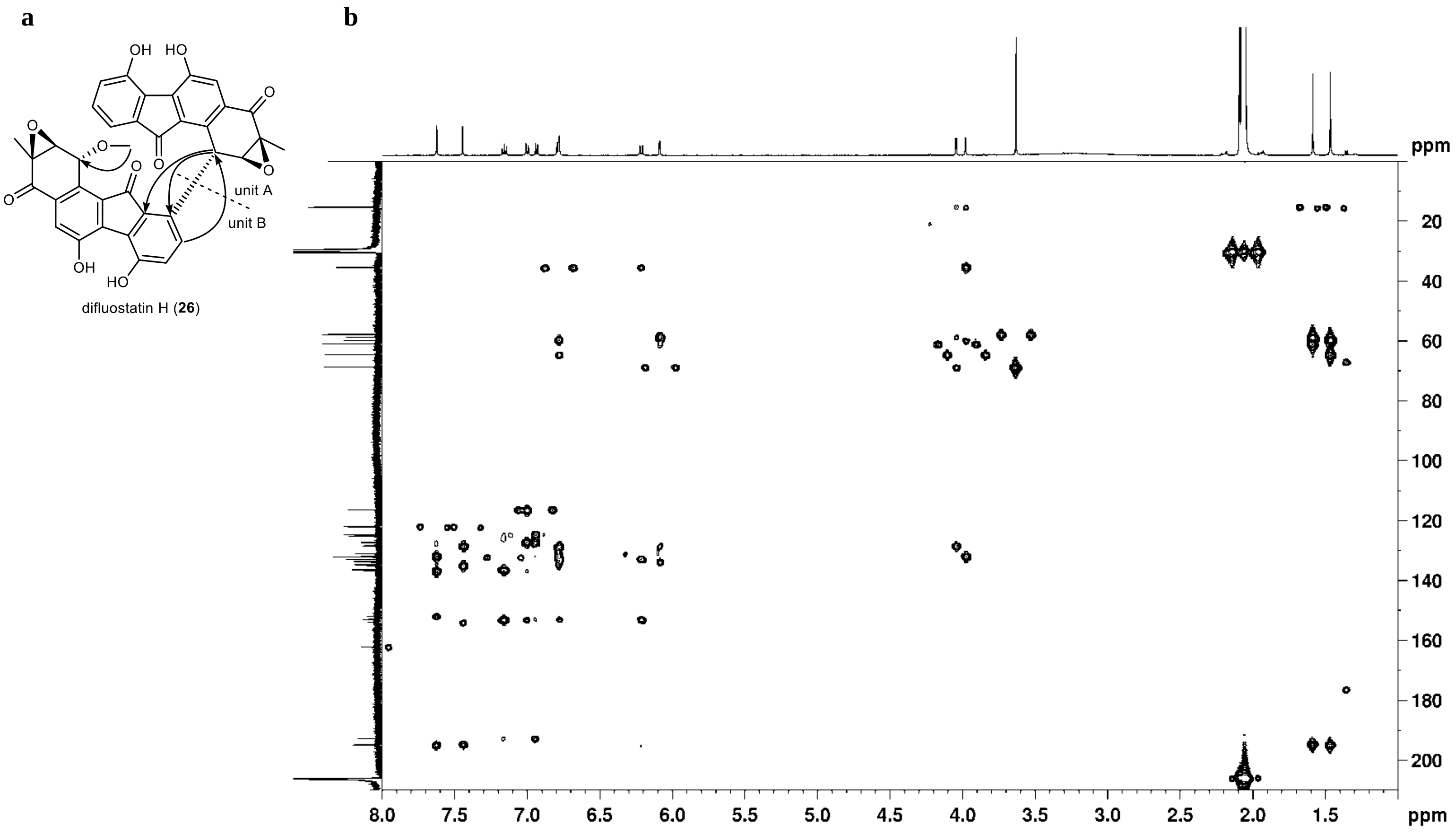

Supplementary Figure 84. The HMBC spectrum of difluostatin H (**26**) in acetone-d_6. **a** Selected key HMBC correlations are indicated by the curved arrows. **b** The HMBC spectrum.

a **b**

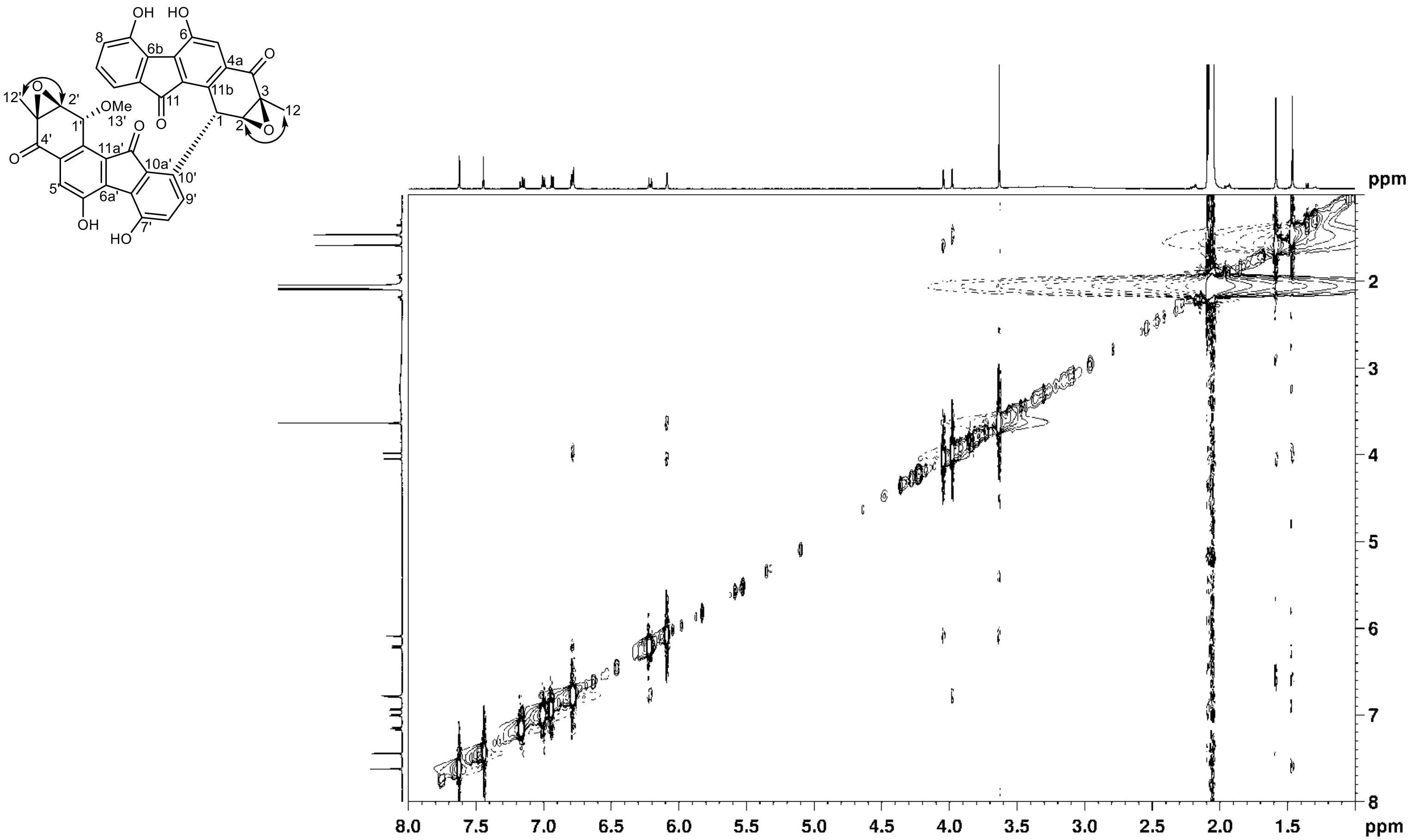

Supplementary Figure 85. The NOESY spectrum of difluostatin H (**26**) in acetone-d_6. **a** Selected key NOESY correlations are indicated by the curved, double-headed arrows. **b** The NOESY spectrum.

a

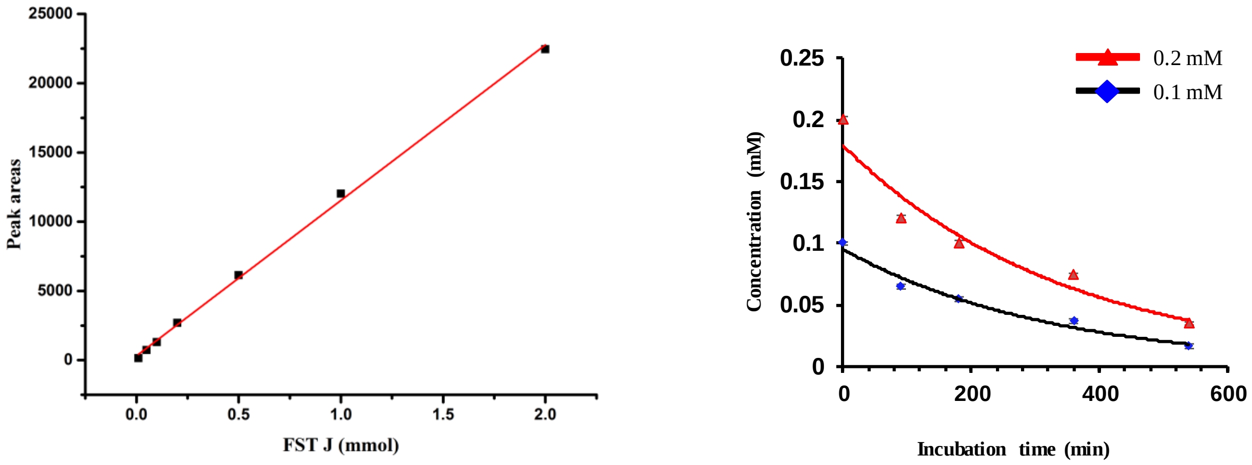

b

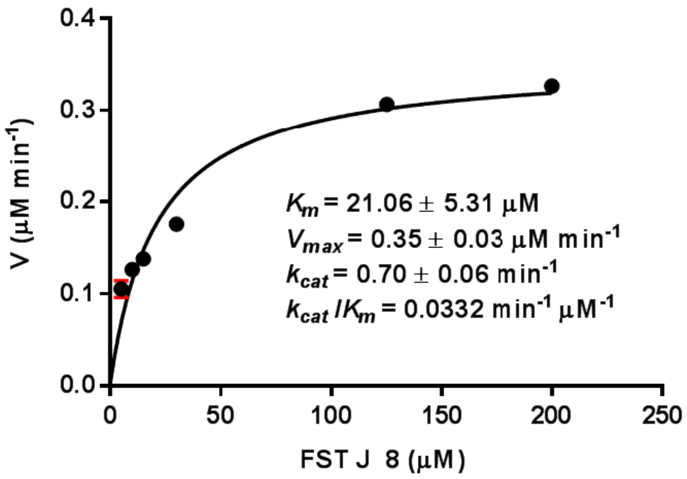

Supplementary Figure 86. Kinetics analyses of spontaneous and FlsH-catalyzed deacylation reactions. a Kinetics analysis of spontaneous deacylation reaction. The standard curve of the concentrations of FST J (**8**) (on the X axis) versus the peak areas (on the Y axis); Time-course analysis of degradation of FST J (**8**) and the curve fitting. A standard curve was first plotted. At first, the stock solution of FST J (**8**) was prepared in DMSO at 40 mM and diluted to different concentrations (2, 1, 0.5, 0.2, 0.1, 0.05, and 0.01 mM), and then subjected to HPLC analysis. The standard curve of the concentrations (on the X axis) versus the peak areas (on the Y axis) was plotted: $y = 11227x + 307.4$, $R^2 = 0.9987$. Secondly, **8** standard from the stock solution was dissolved in water with the final concentrations of 0.1 and 0.2 mM, and subsequently divided into five 1.5 mL tubes, respectively. Each group was performed in triplicate. All samples were incubated at 30 °C. At each time point (0, 90, 180, 360, and 540 min), a tube of reaction mixture was collected and stored at -80 °C before use. After the last sampling, these samples were subjected to HPLC analysis. The content of FST J (**8**) at each time point was determined by the peak areas which were fit to the standard curve. The curve of incubation time versus the concentrations of FST J (**8**) was obtained by fitting experimental data. Linear correlation coefficients and equations were shown for each curve ($C_t = 0.0952\ e^{-0.003x}$ ($R^2 = 0.9759$) for 0.1 mM; $C_t = 0.1794\ e^{-0.003x}$ ($R^2 = 0.9609$) for 0.2 mM), and then the constant (k_{non}) for nonenzymatic degradation was determined. Error bars represent ± SD (standard deviation). **b** Kinetics analysis of FlsH-catalyzed deacylation reaction. For the substrate **8** with 5-250 μM.

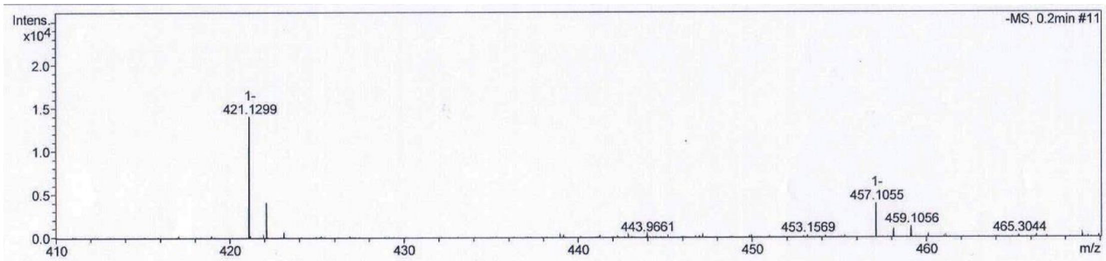

Meas. m/z	#	Ion Formula	Score	m/z	err [ppm]	err [mDa]	mSigma	rdb	e⁻ Conf	N-Rule
421.129884	1	C24H21O7	100.00	421.129277	1.4	0.6	14.5	14.5	even	ok
457.105506	1	C24H22ClO7	100.00	457.105954	-1.0	-0.4	24.2	13.5	even	ok
843.264547	1	C48H43O14	100.00	843.265830	-1.5	-1.3	44.3	27.5	even	ok
879.240838	1	C48H44ClO14	100.00	879.242507	1.9	1.7	23.2	26.5	even	ok

Supplementary Figure 87. HRESIMS spectrum of compound **27**.

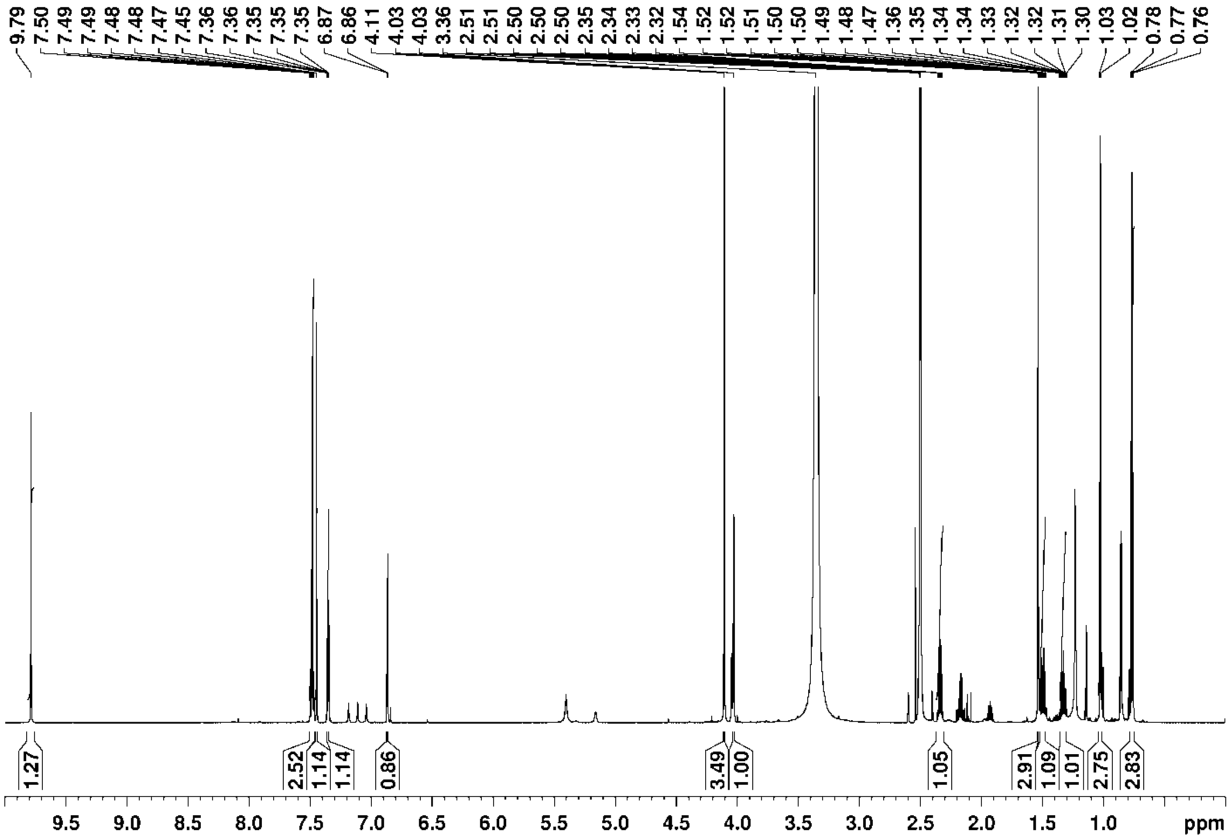

Supplementary Figure 88. The ^{1}H NMR spectrum of compound **27** in DMSO-d_6.

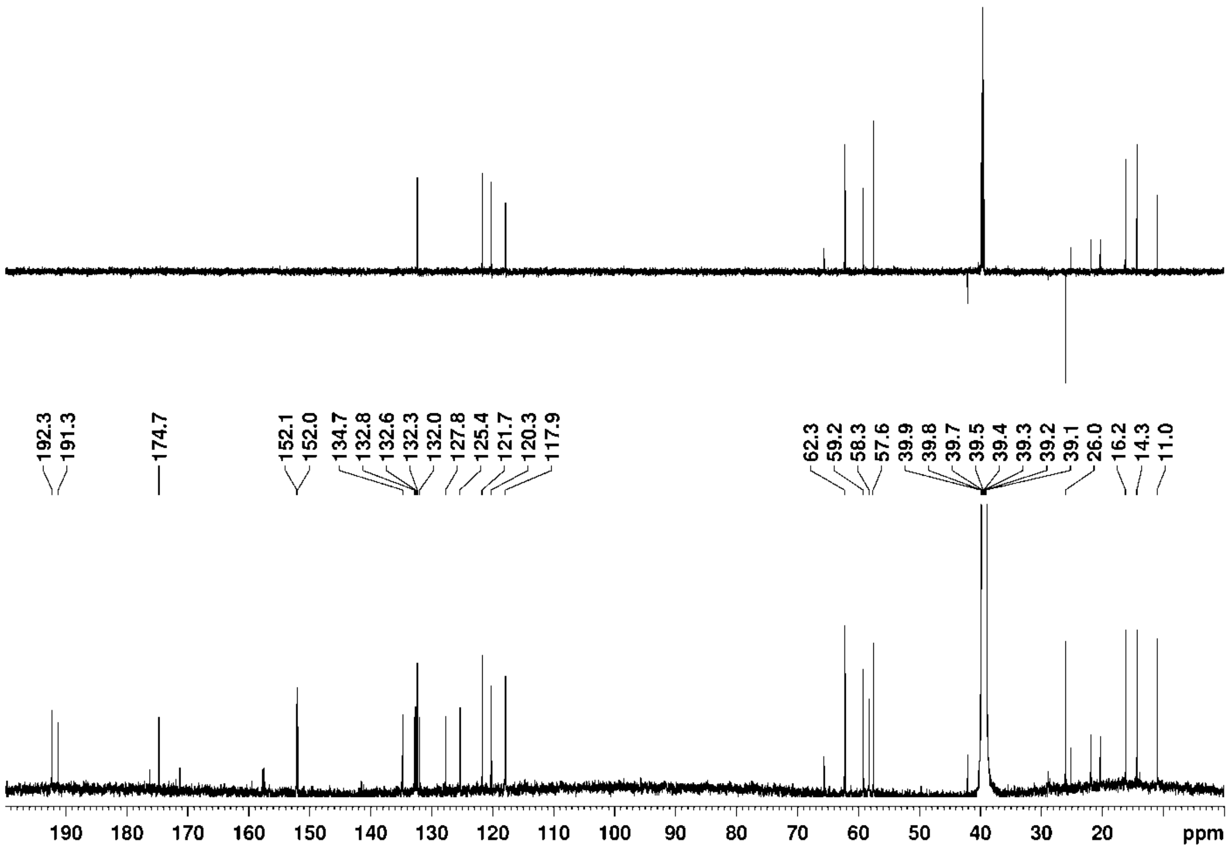

Supplementary Figure 89. The ^{13}C and DEPT 135 NMR spectrum of compound **27** in DMSO-d_6.

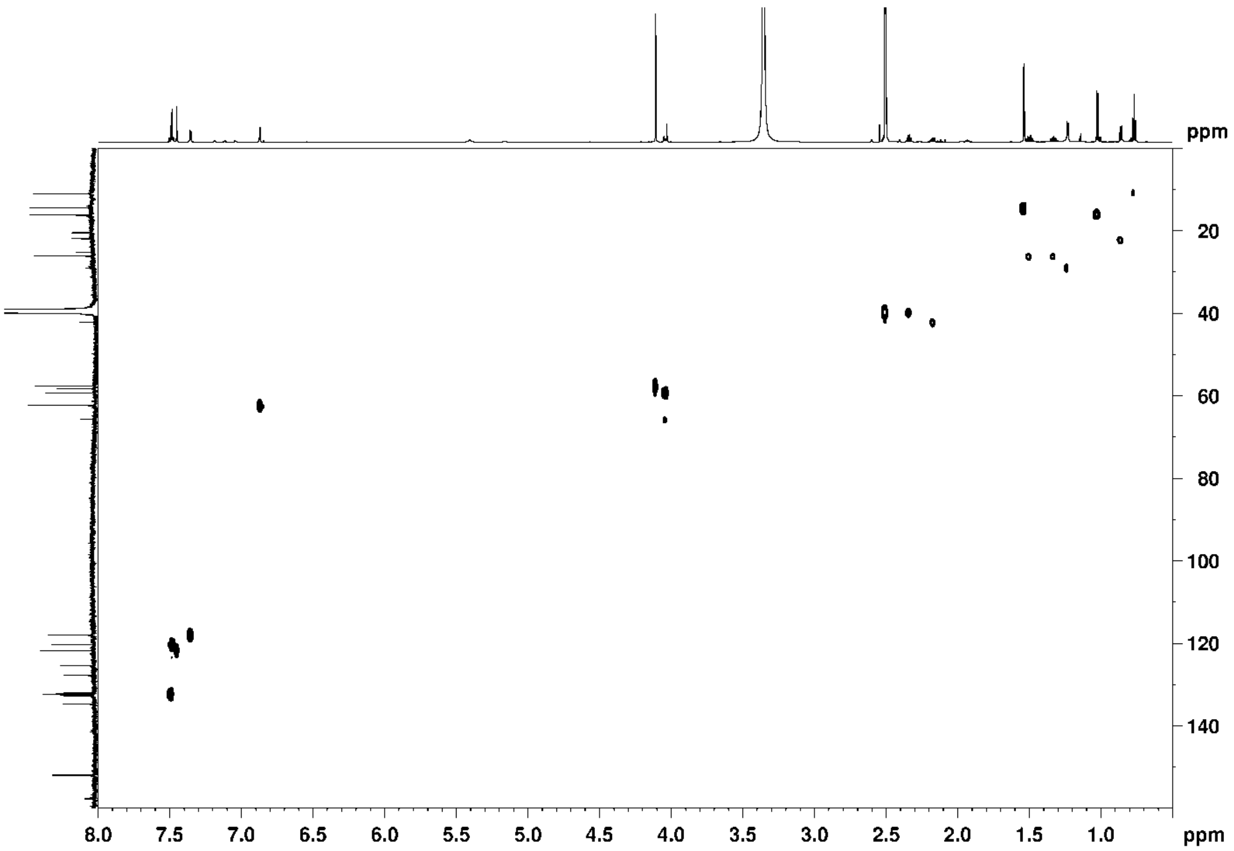

Supplementary Figure 90. The HSQC spectrum of compound **27** in DMSO-d_6.

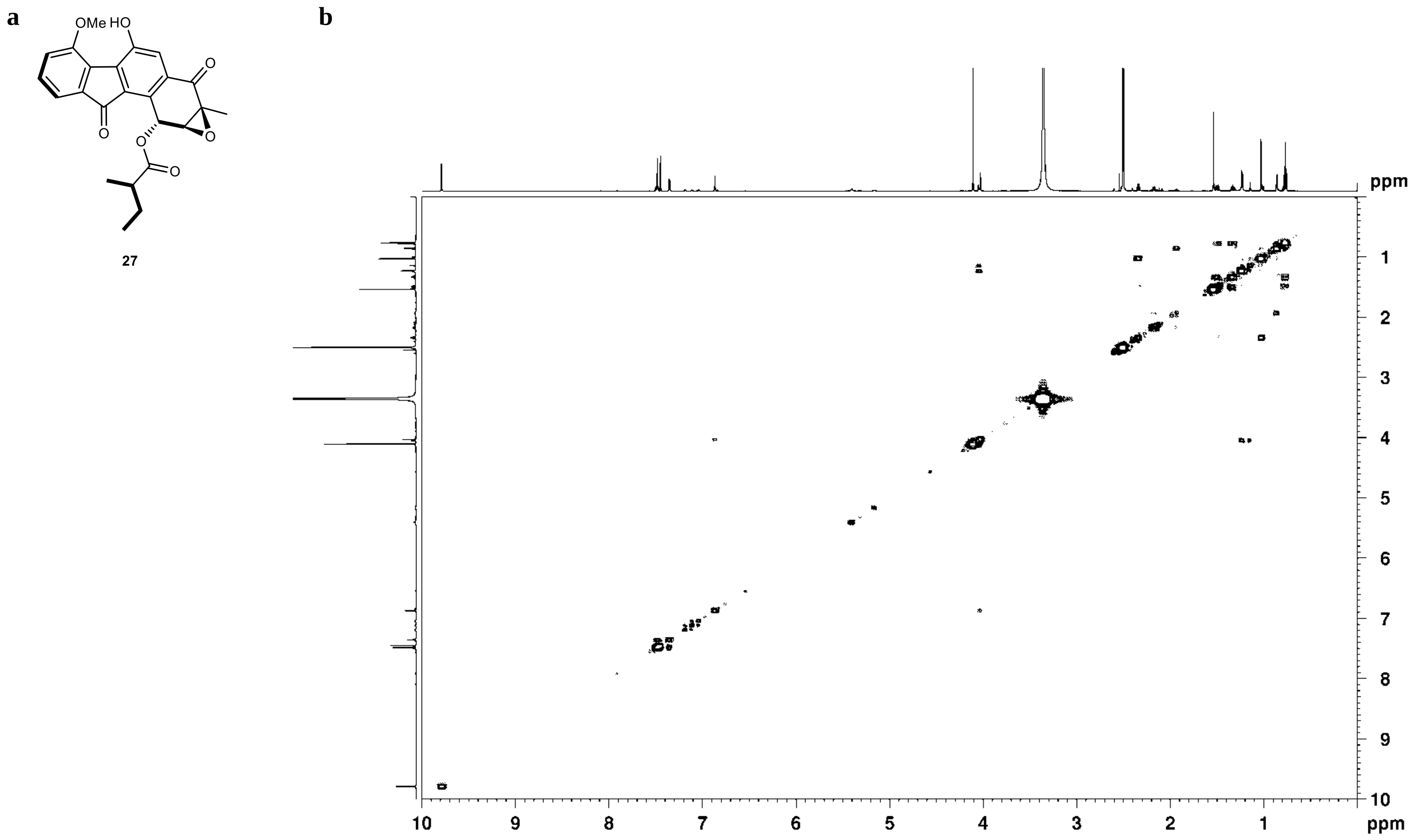

Supplementary Figure 91. The COSY spectrum of compound **27** in DMSO-d_6. **a** COSY correlations are indicated by boldface bonds. **b** The COSY spectrum.

a **b**

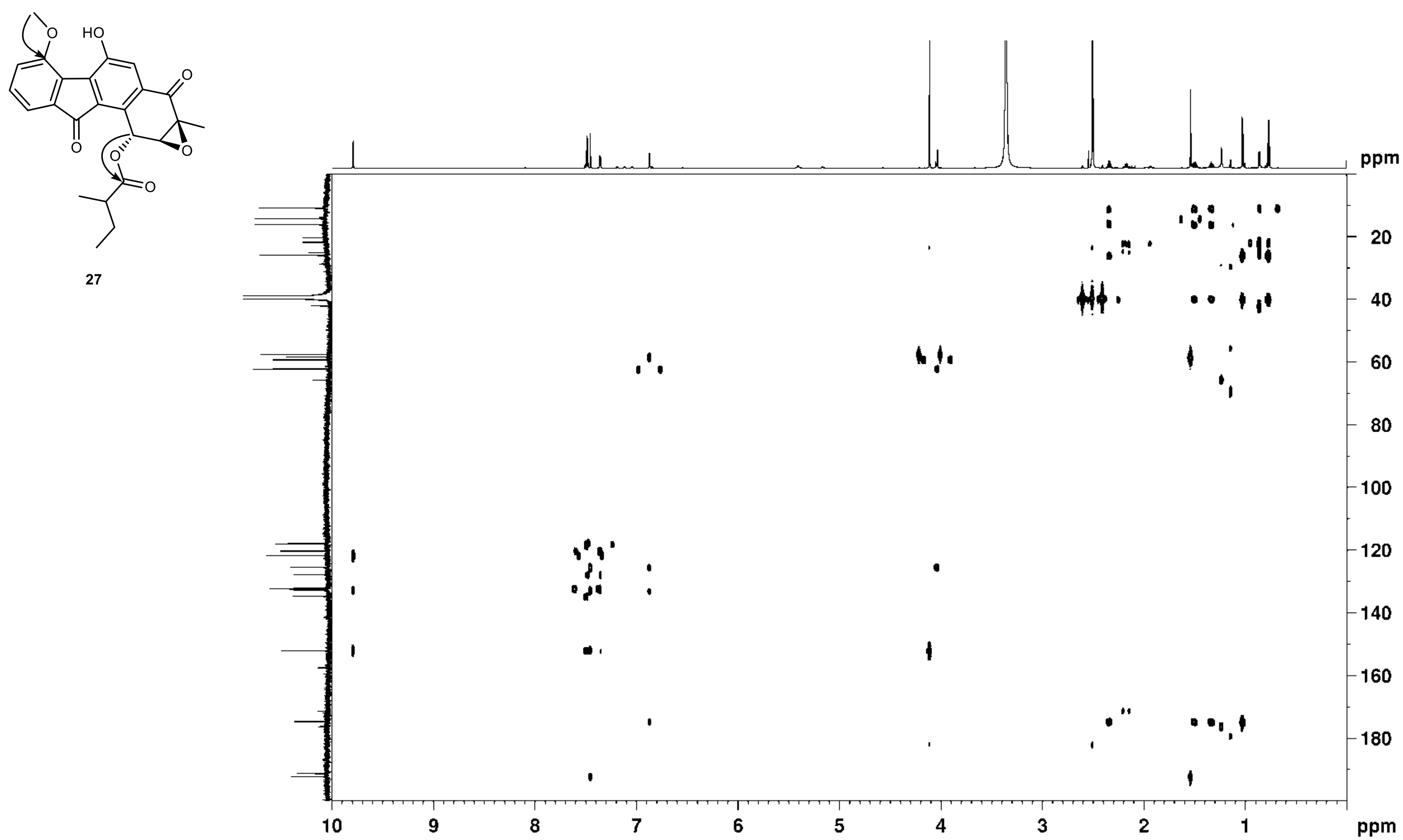

Supplementary Figure 92. The HMBC spectrum of compound **27** in DMSO-d_6. **a** Selected key HMBC correlations are indicated by the curved arrows. **b** The HMBC spectrum.

a

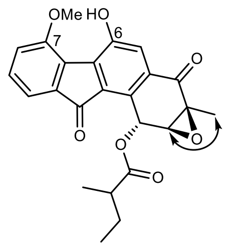

27

b

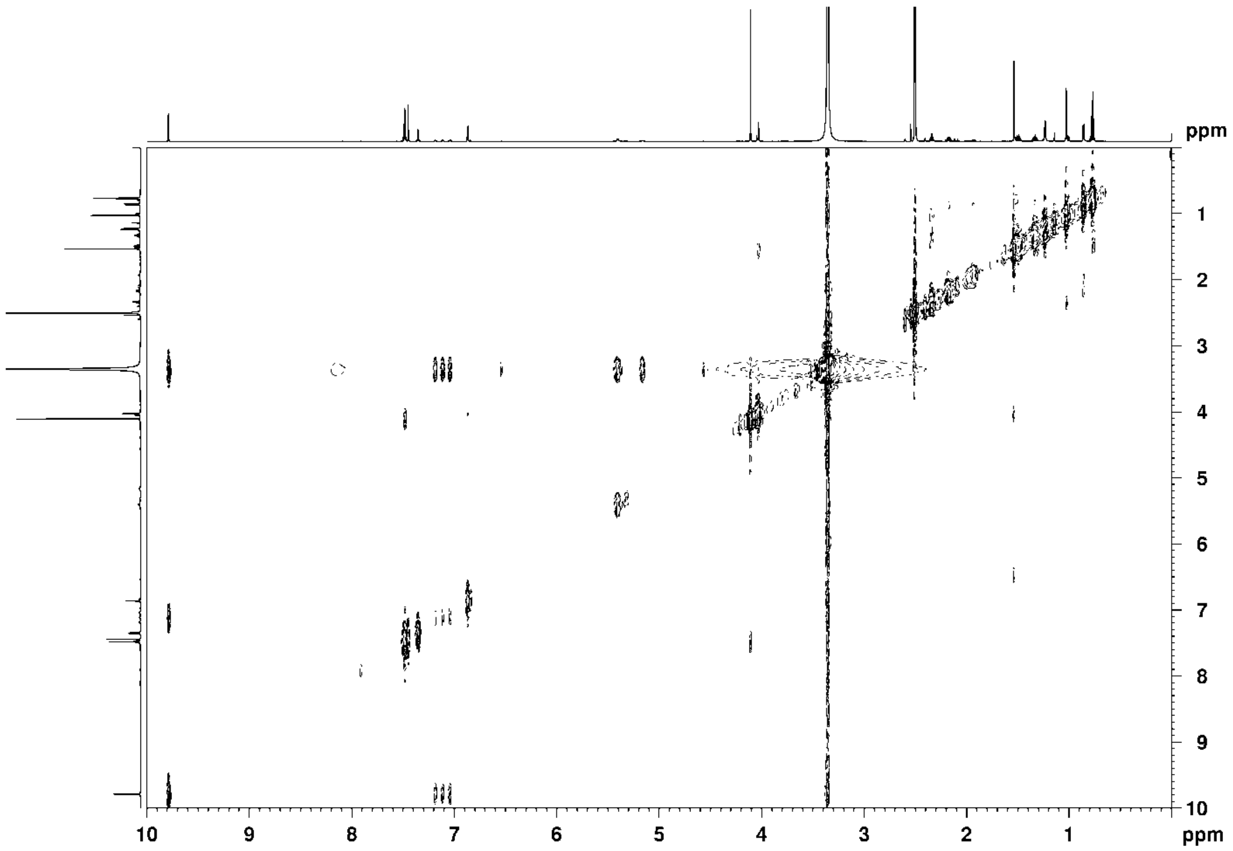

Supplementary Figure 93. The NOESY spectrum of compound **27** in DMSO-d_6. **a** Selected key NOESY correlations are indicated by the curved, double-headed arrows. **b** The NOESY spectrum.

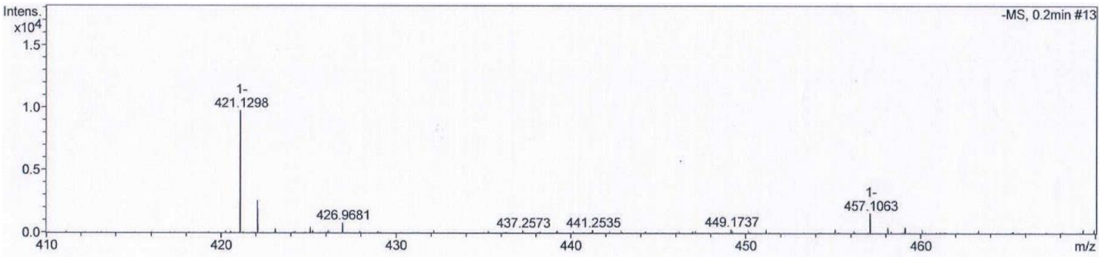

Meas. m/z	#	Ion Formula	Score	m/z	err [ppm]	err [mDa]	mSigma	rdb	e⁻ Conf	N-Rule
421.129752	1	C24H21O7	100.00	421.129277	-1.1	-0.5	6.6	14.5	even	ok
457.106258	1	C24H22ClO7	100.00	457.105954	0.7	0.3	38.4	13.5	even	ok

Supplementary Figure 94. HRESIMS spectrum of compound **28**.

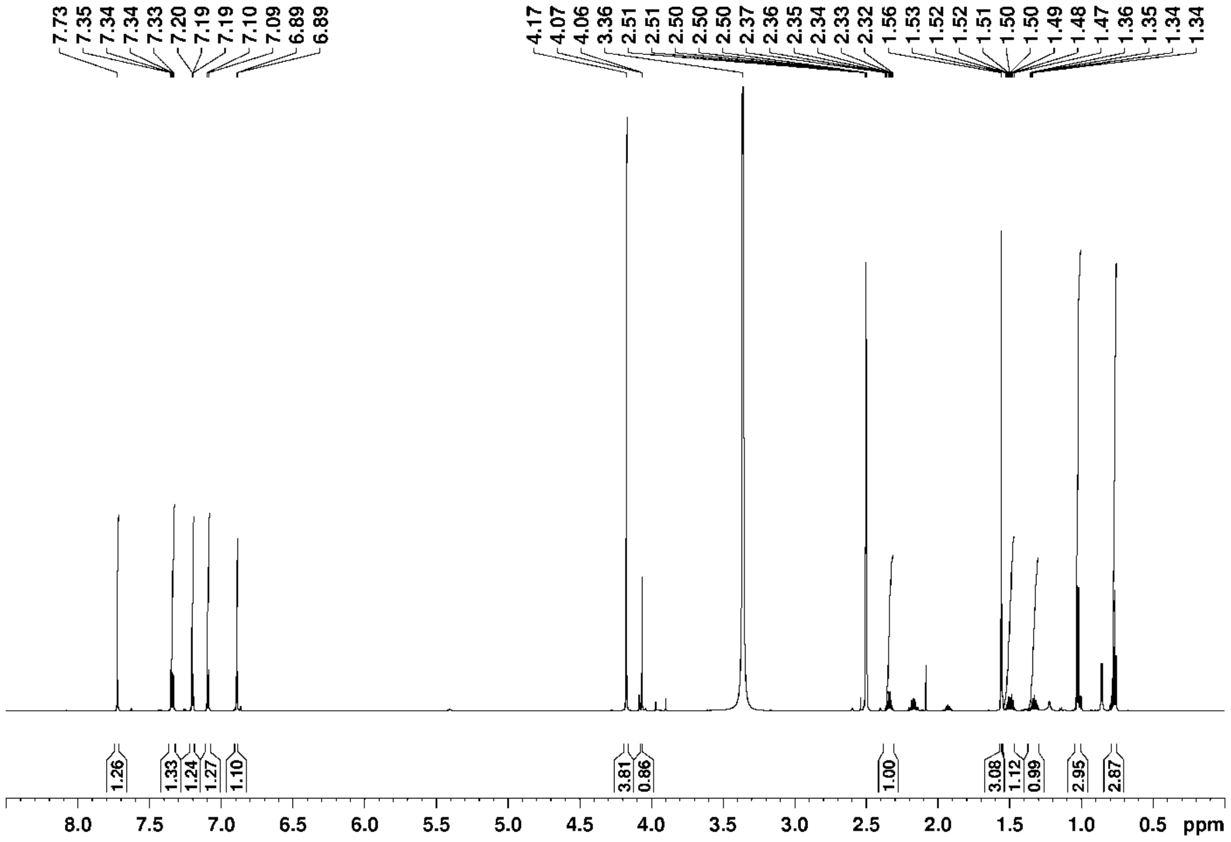

Supplementary Figure 95. The ^{1}H NMR spectrum of compound **28** in DMSO-d_6.

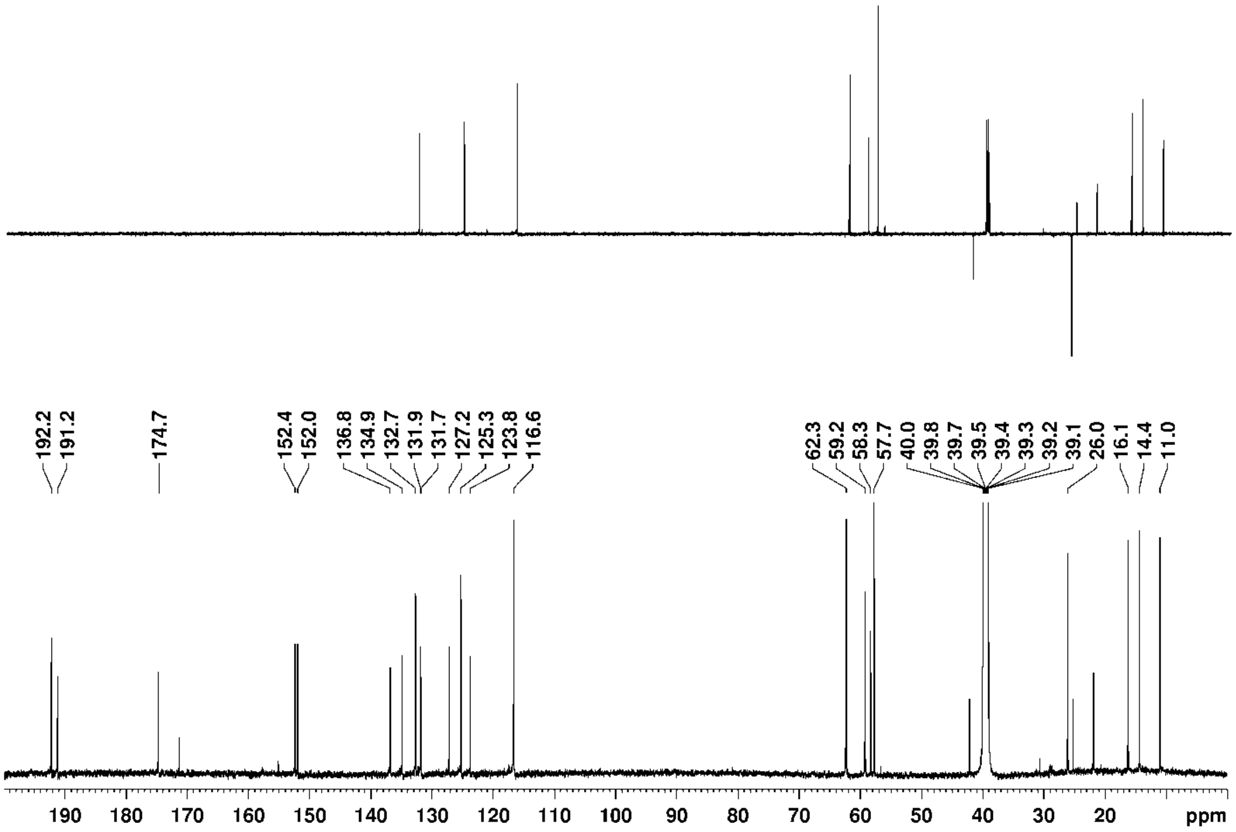

Supplementary Figure 96. The ^{13}C and DEPT 135 NMR spectrum of compound **28** in DMSO-d_6.

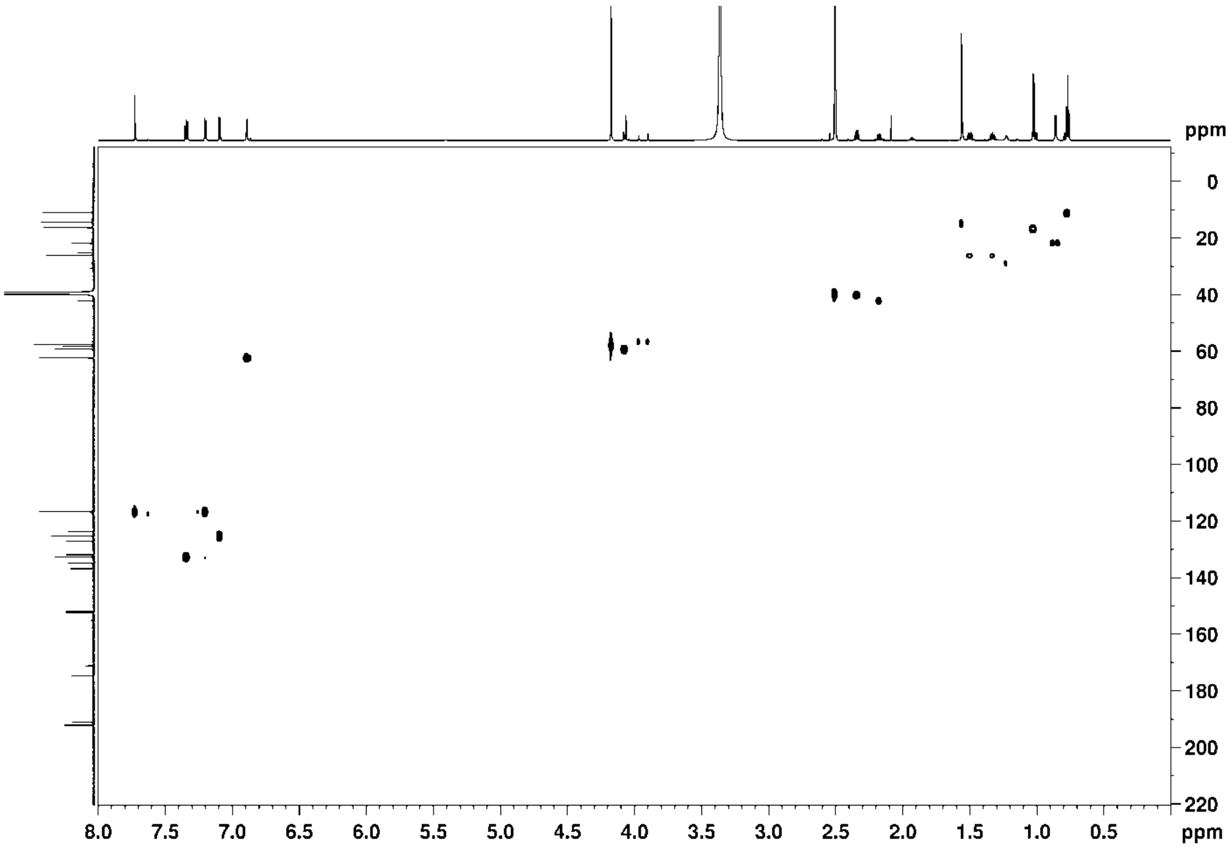

Supplementary Figure 97. The HSQC spectrum of compound **28** in DMSO-d_6.

a

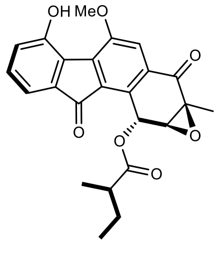

28

b

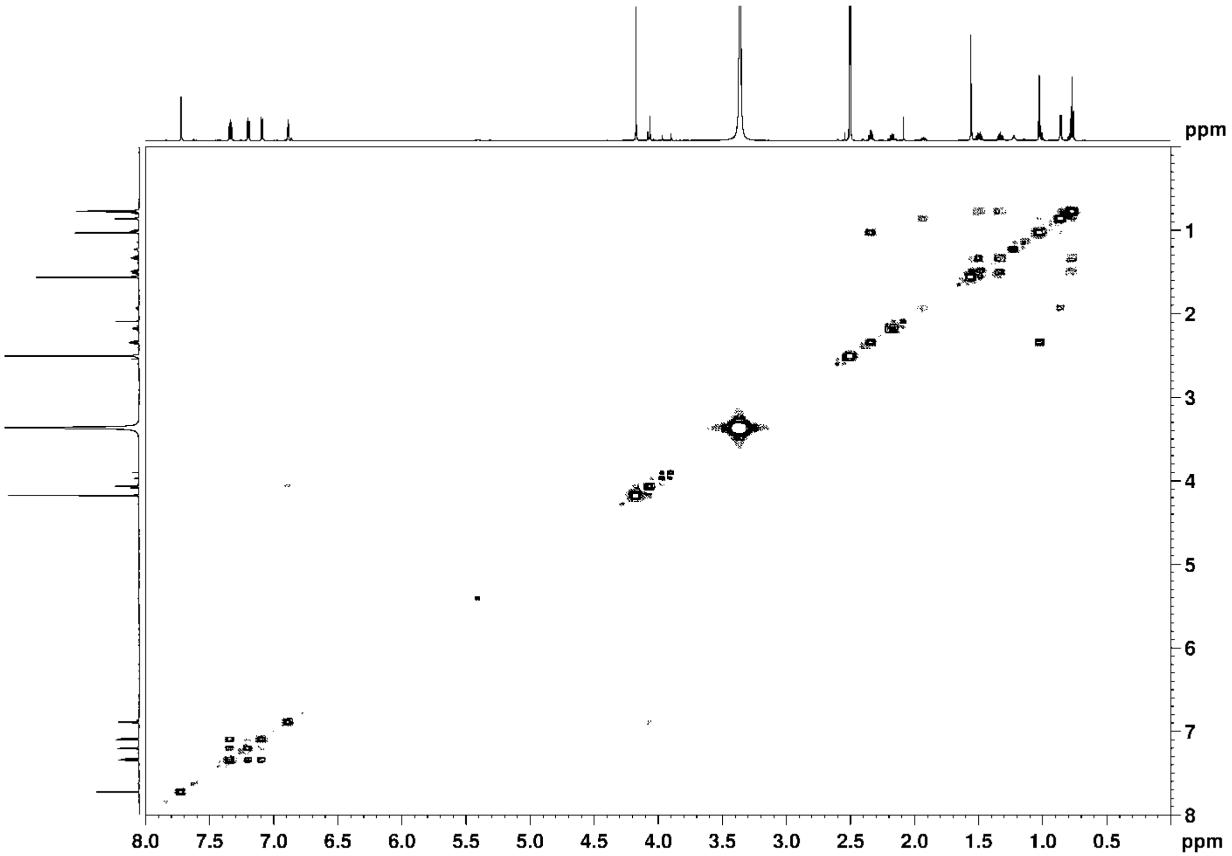

Supplementary Figure 98. The COSY spectrum of compound **28** in DMSO-d_6. **a** COSY correlations are indicated by boldface bonds. **b** The COSY spectrum.

a

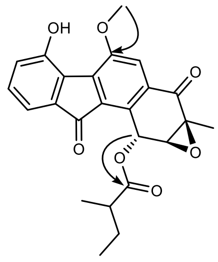

28

b

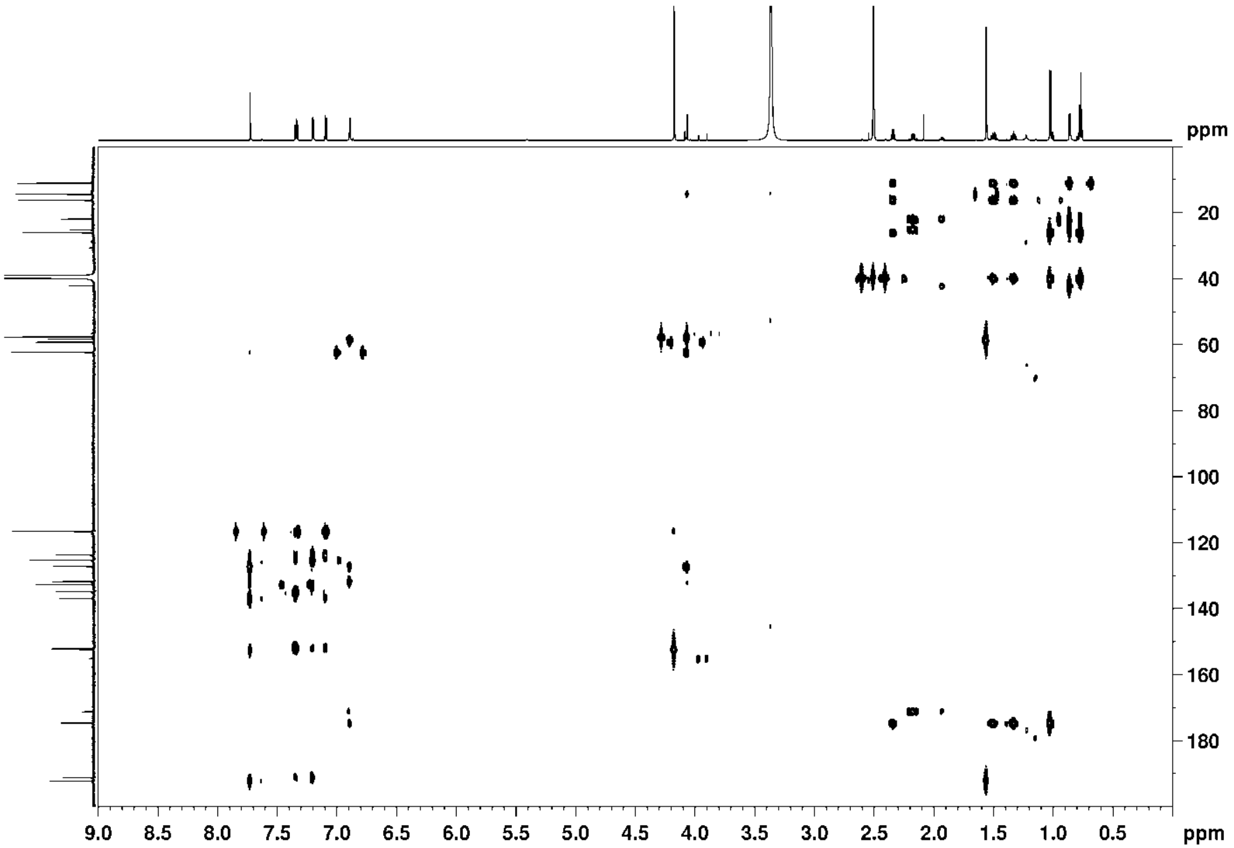

Supplementary Figure 99. The HMBC spectrum of compound **28** in DMSO-d_6. **a** Selected key HMBC correlations are indicated by the curved arrows. **b** The HMBC spectrum.

a

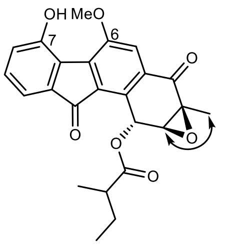

28

b

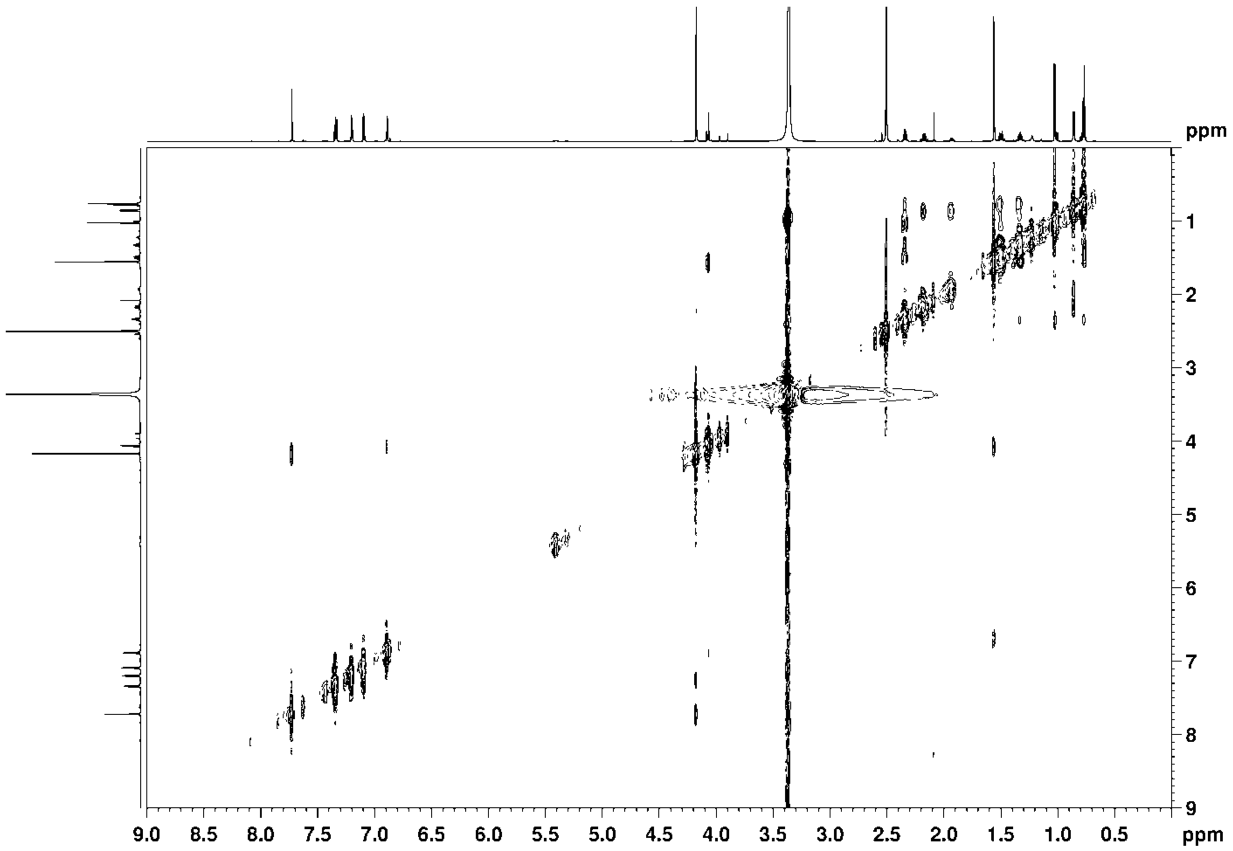

Supplementary Figure 100. The NOESY spectrum of compound **28** in DMSO-d_6. **a** Selected key NOESY correlations are indicated by the curved, double-headed arrows. **b** The NOESY spectrum.

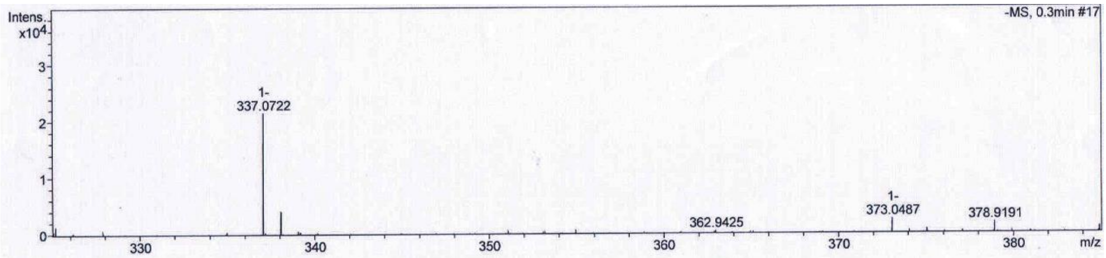

Meas. m/z	#	Ion Formula	Score	m/z	err [ppm]	err [mDa]	mSigma	rdb	e⁻ Conf	N-Rule
337.072245	1	C19H13O6	100.00	337.071762	-1.4	-0.5	10.1	13.5	even	ok
373.048720	1	C19H14ClO6	100.00	373.048439	0.8	0.3	28.1	12.5	even	ok
675.151086	1	C38H27O12	100.00	675.150800	0.4	0.3	45.5	25.5	even	ok
711.127252	1	C38H28ClO12	100.00	711.127478	0.3	0.2	31.1	24.5	even	ok

Supplementary Figure 101. HRESIMS spectrum of compound **29**.

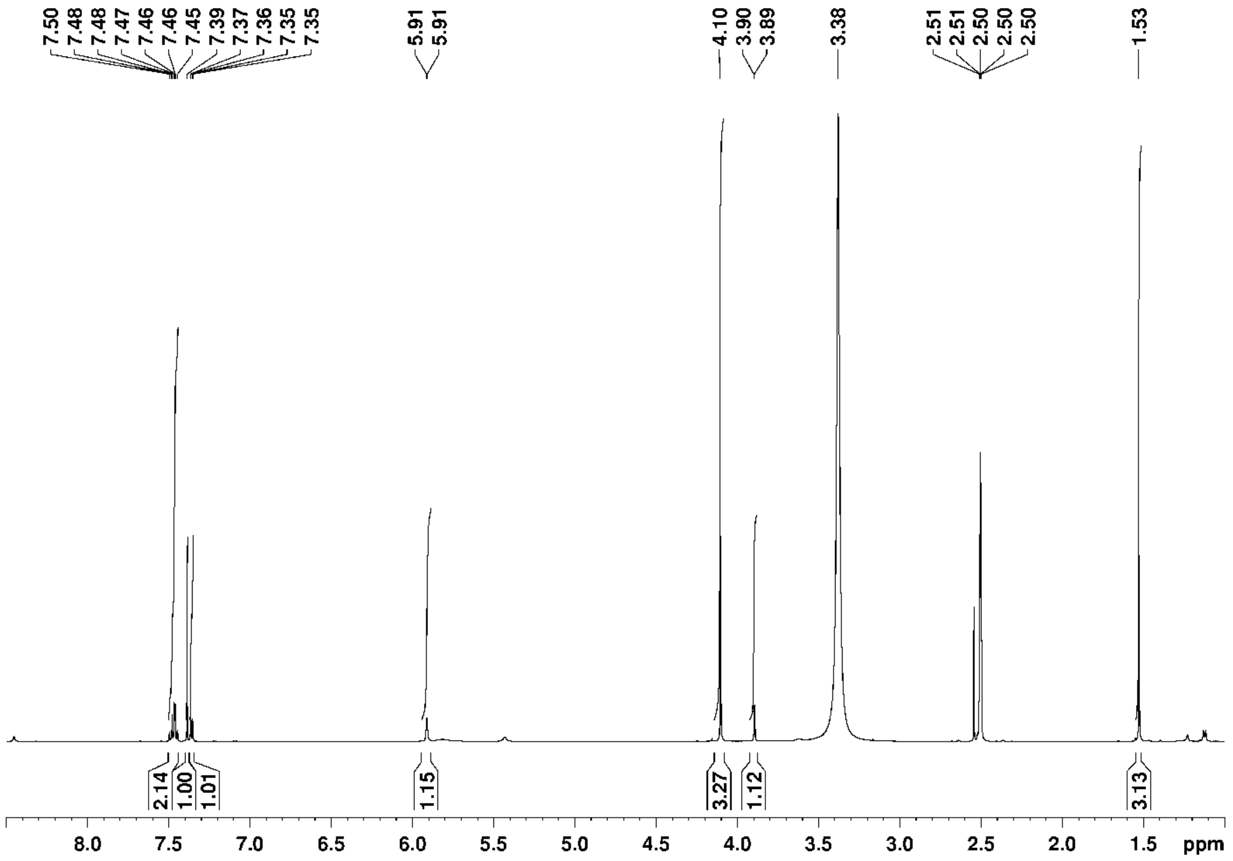

Supplementary Figure 102. The ^{1}H NMR spectrum of compound **29** in DMSO-d_6.

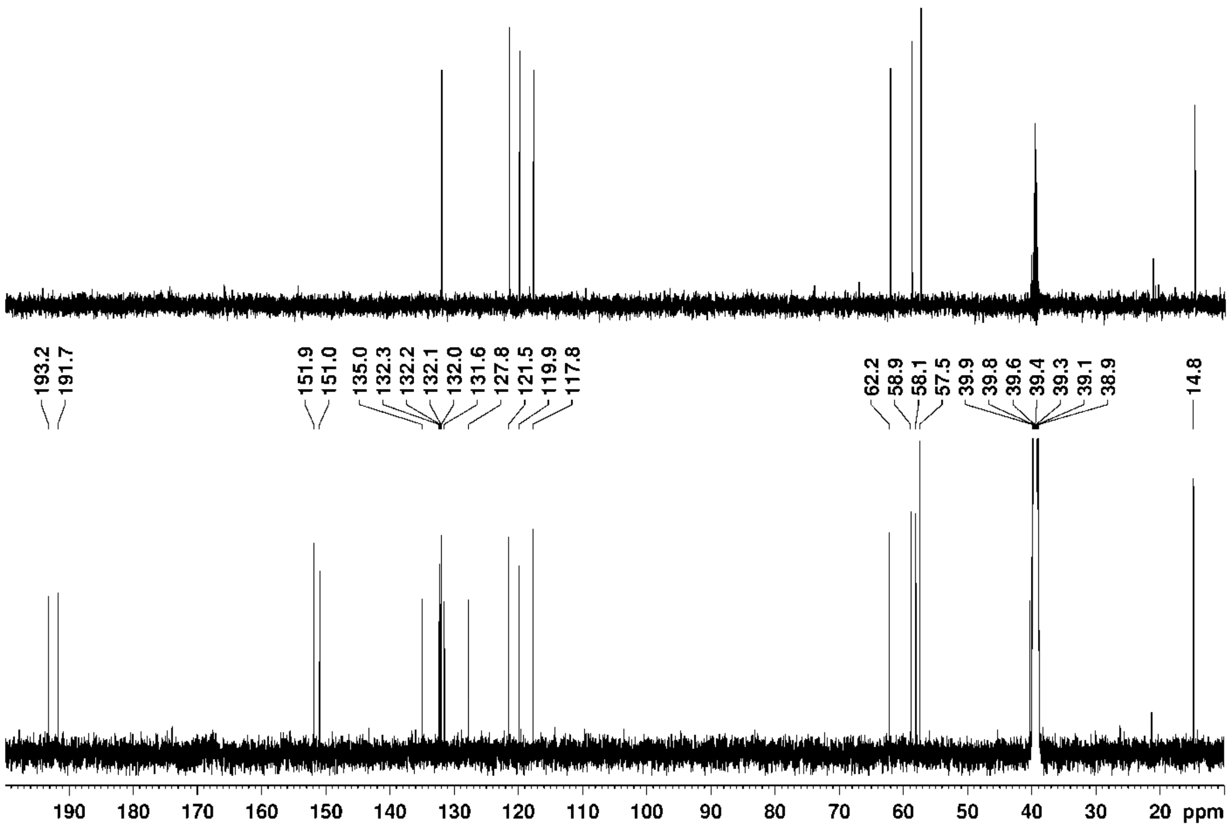

Supplementary Figure 103. The ^{13}C and DEPT 135 NMR spectrum of compound **29** in DMSO-d_6.

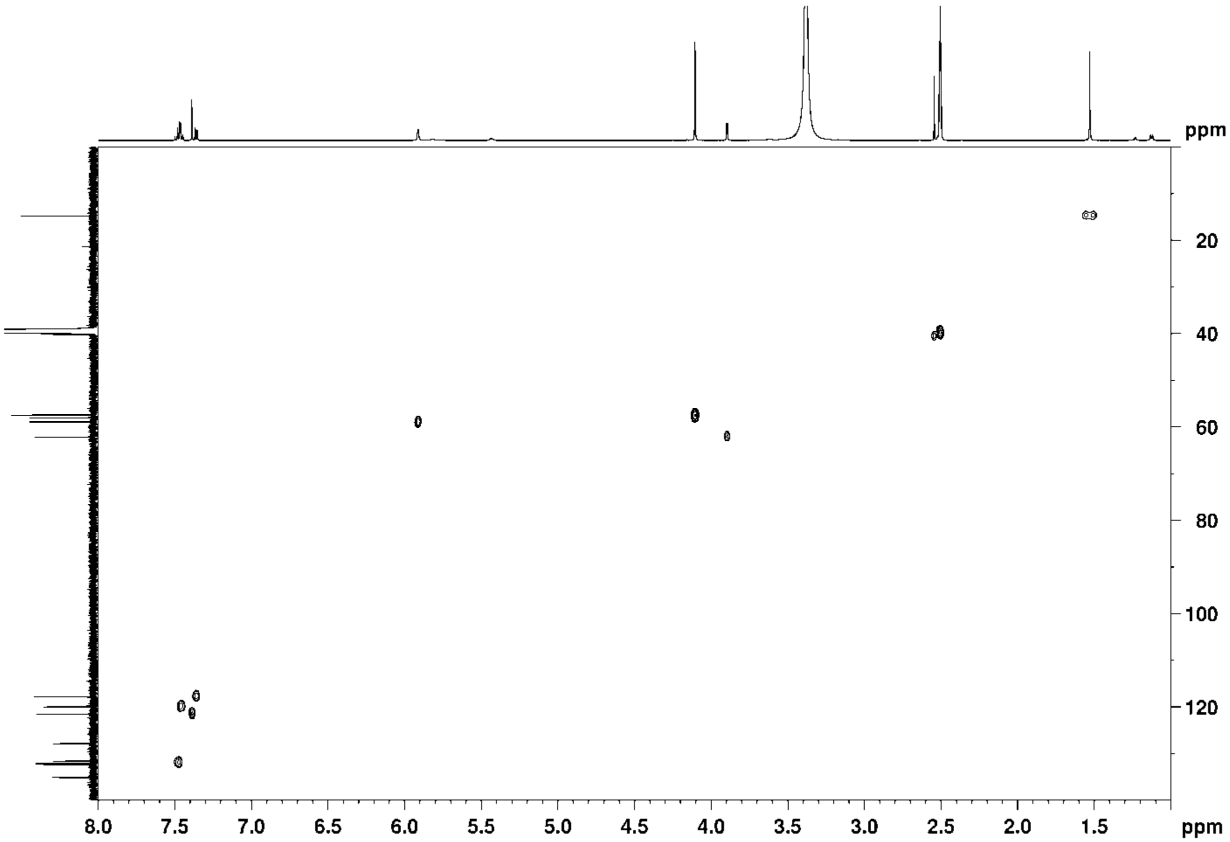

Supplementary Figure 104. The HSQC spectrum of compound **29** in DMSO-d_6.

a

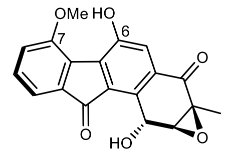

29

b

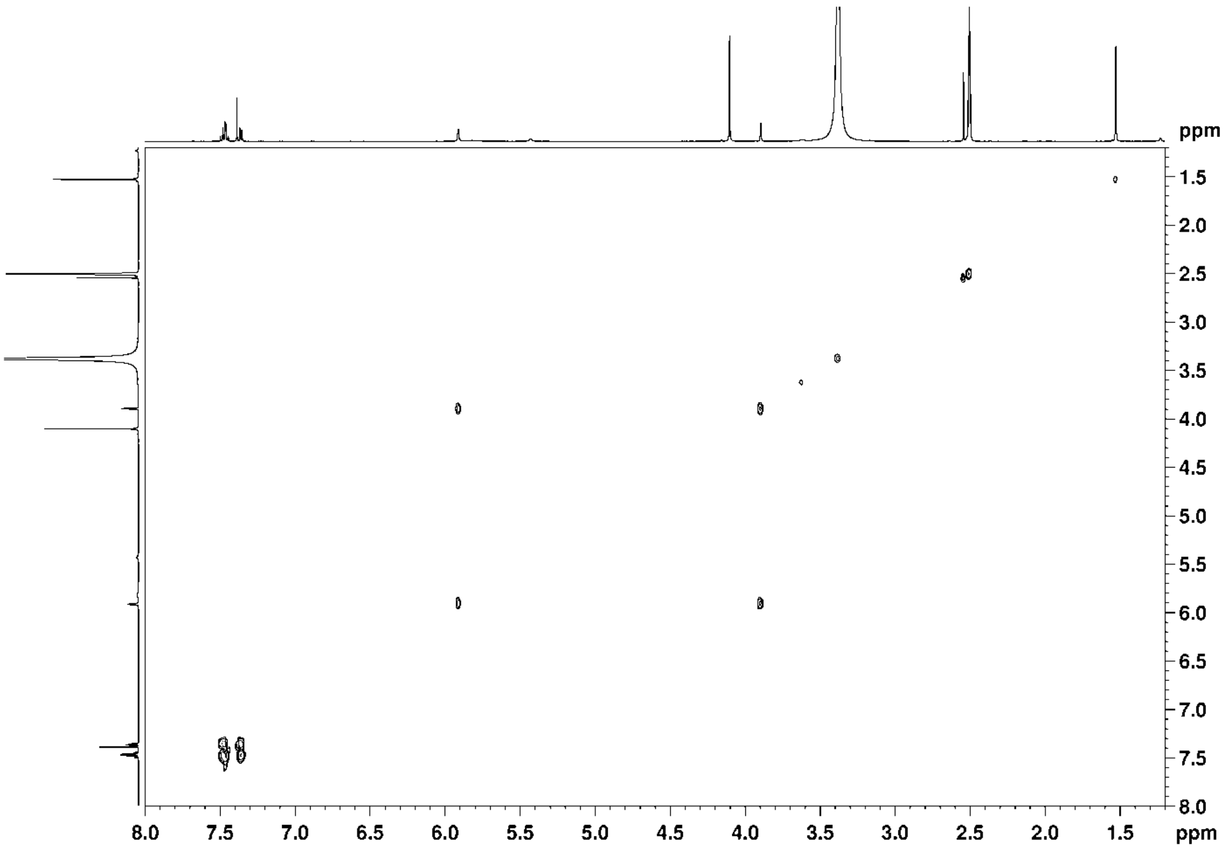

Supplementary Figure 105. The COSY spectrum of compound **29** in DMSO-d_6. **a** COSY correlations are indicated by boldface bonds. **b** The COSY spectrum.

a

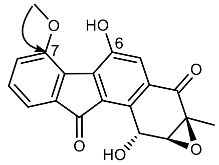

29

b

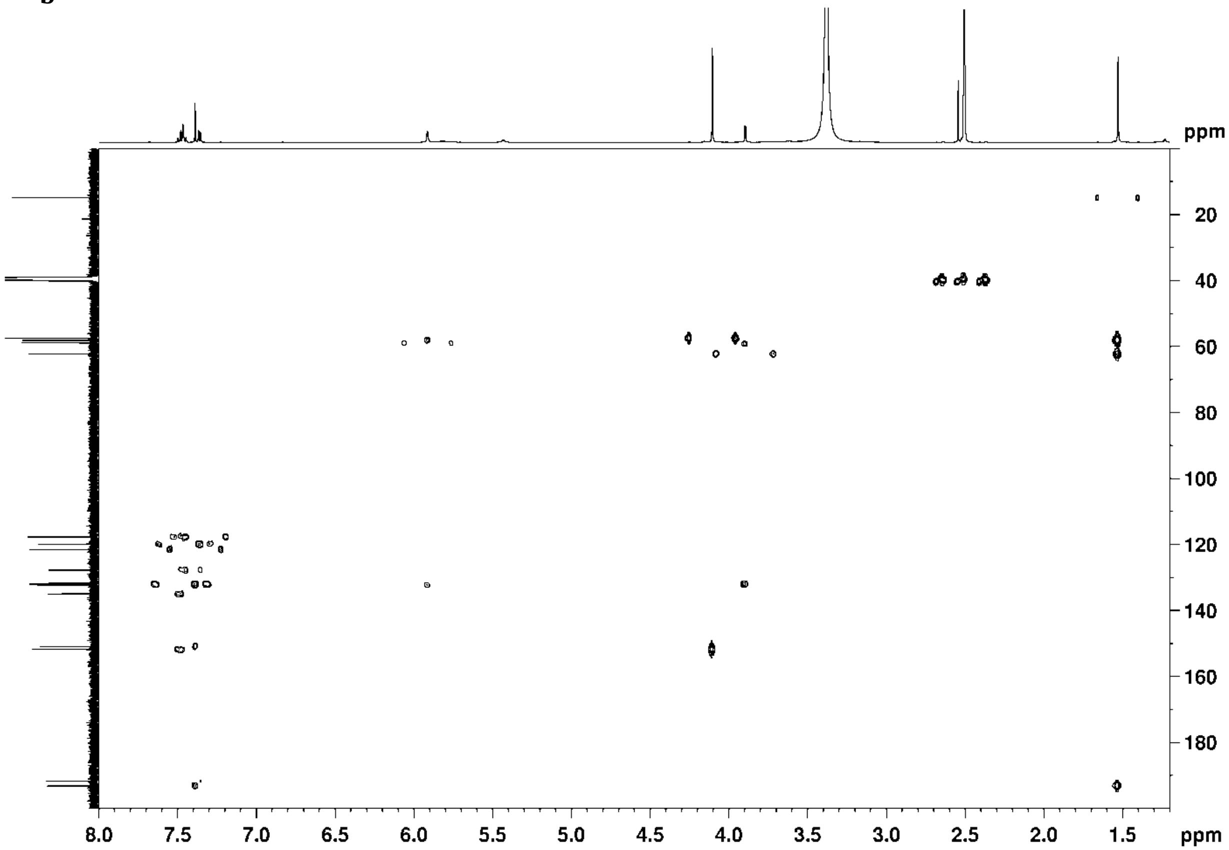

Supplementary Figure 106. The HMBC spectrum of compound **29** in DMSO-d_6. **a** Selected key HMBC correlations are indicated by the curved arrows. **b** The HMBC spectrum.

a

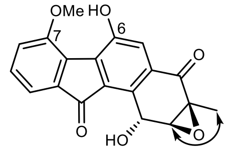

29

b

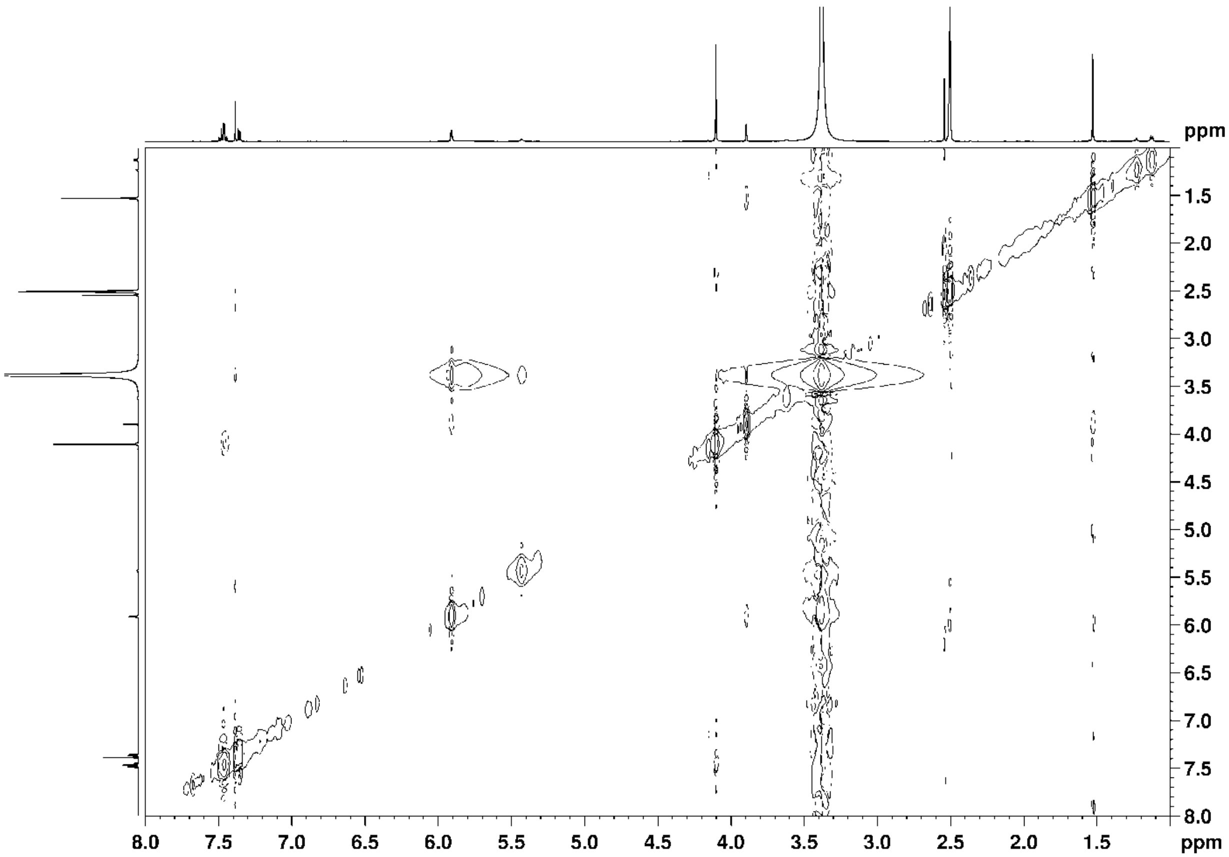

Supplementary Figure 107. The NOESY spectrum of compound **29** in DMSO-d_6. **a** Selected key NOESY correlations are indicated by the curved, double-headed arrows. **b** The NOESY spectrum.

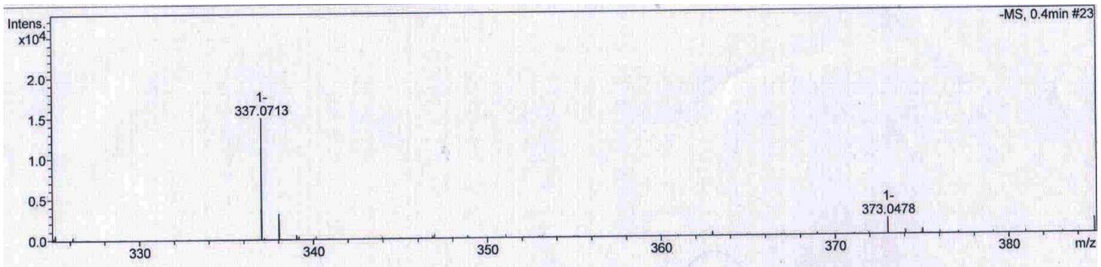

Meas. m/z	#	Ion Formula	Score	m/z	err [ppm]	err [mDa]	mSigma	rdb	e⁻ Conf	N-Rule
337.071266	1	C19H13O6	100.00	337.071762	-1.5	-0.5	3.1	13.5	even	ok
373.047812	1	C19H14ClO6	100.00	373.048439	-1.7	-0.6	30.9	12.5	even	ok
675.150606	1	C38H27O12	100.00	675.150800	0.3	0.2	99.2	25.5	even	ok
711.125749	1	C38H28ClO12	100.00	711.127478	-2.4	-1.7	68.7	24.5	even	ok

Supplementary Figure 108. HRESIMS spectrum of compound **30**.

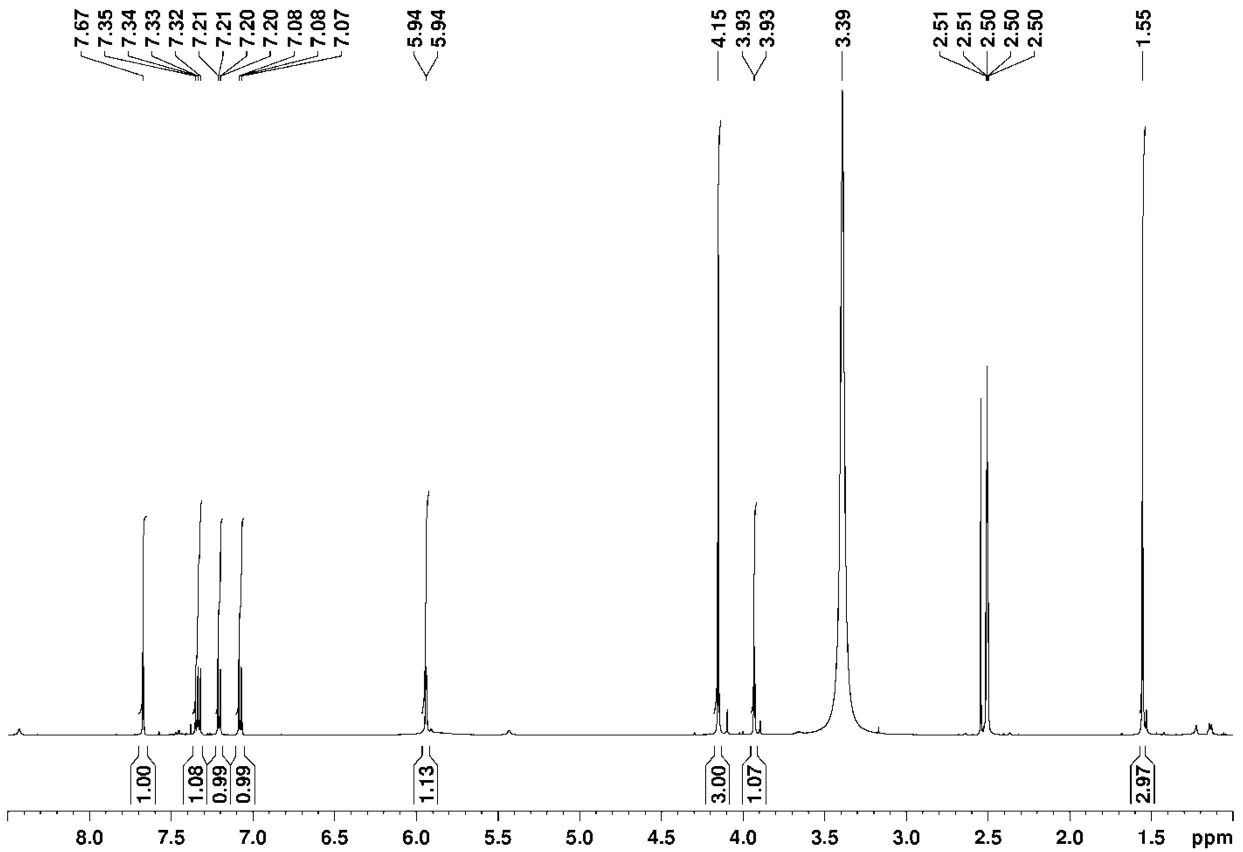

Supplementary Figure 109. The ^{1}H NMR spectrum of compound **30** in DMSO-d_6.

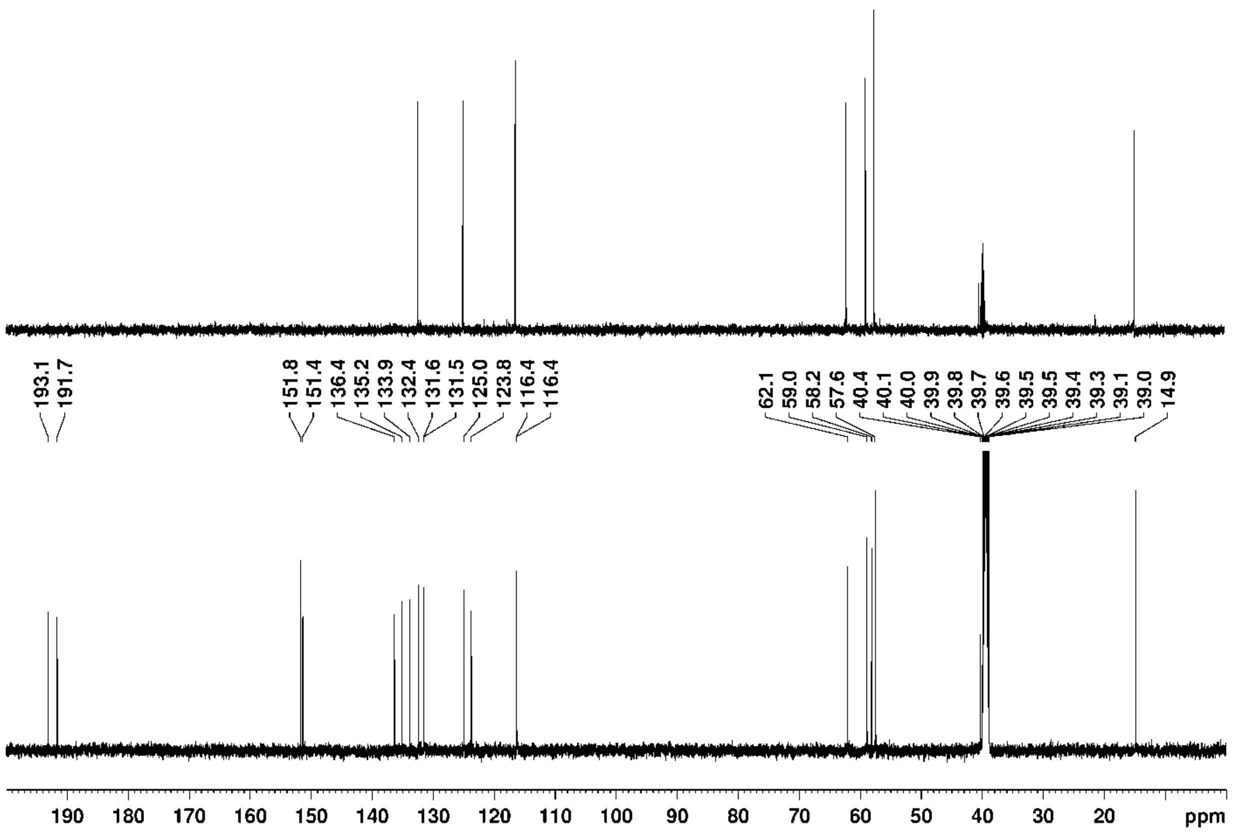

Supplementary Figure 110. The ^{13}C and DEPT 135 NMR spectrum of compound **30** in DMSO-d_6.

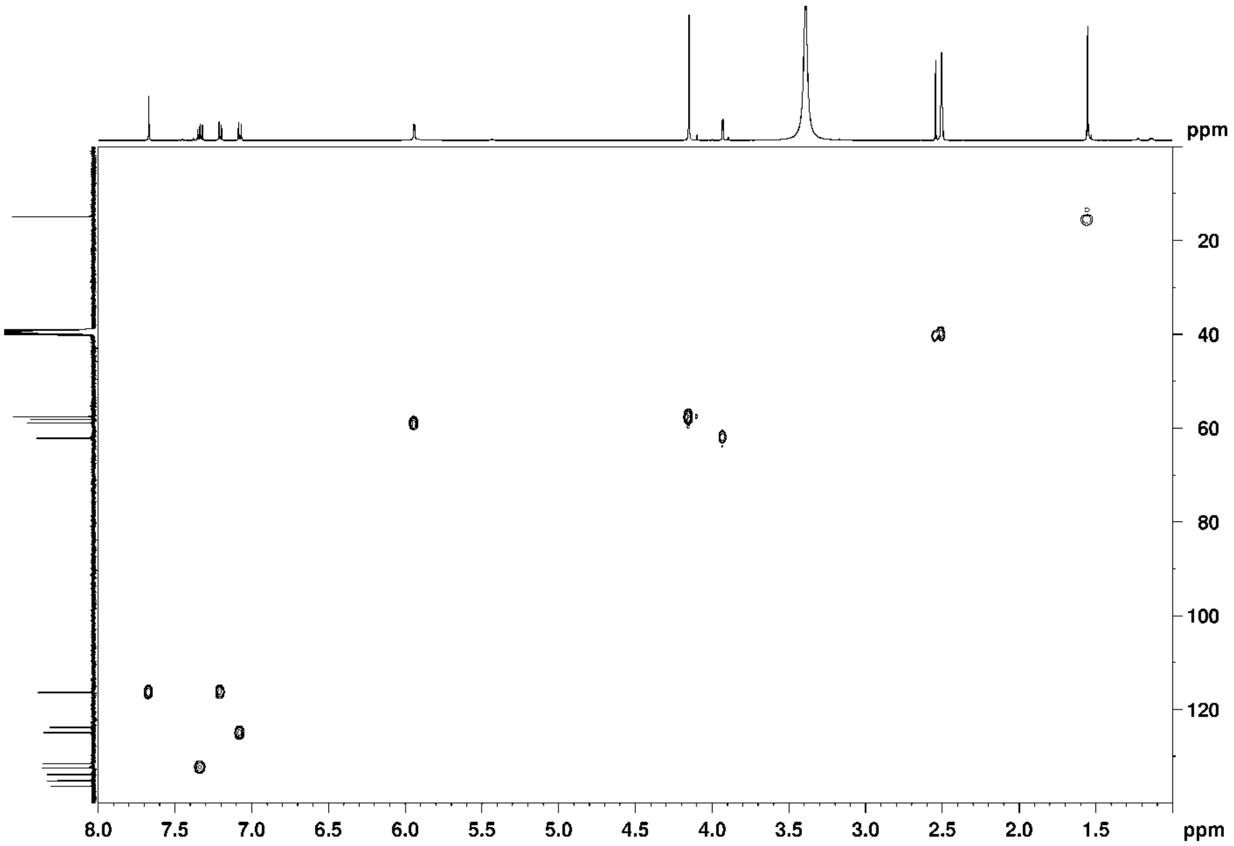

Supplementary Figure 111. The HSQC spectrum of compound **30** in DMSO-d_6.

a

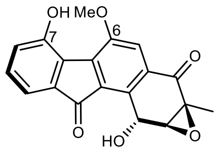

30

b

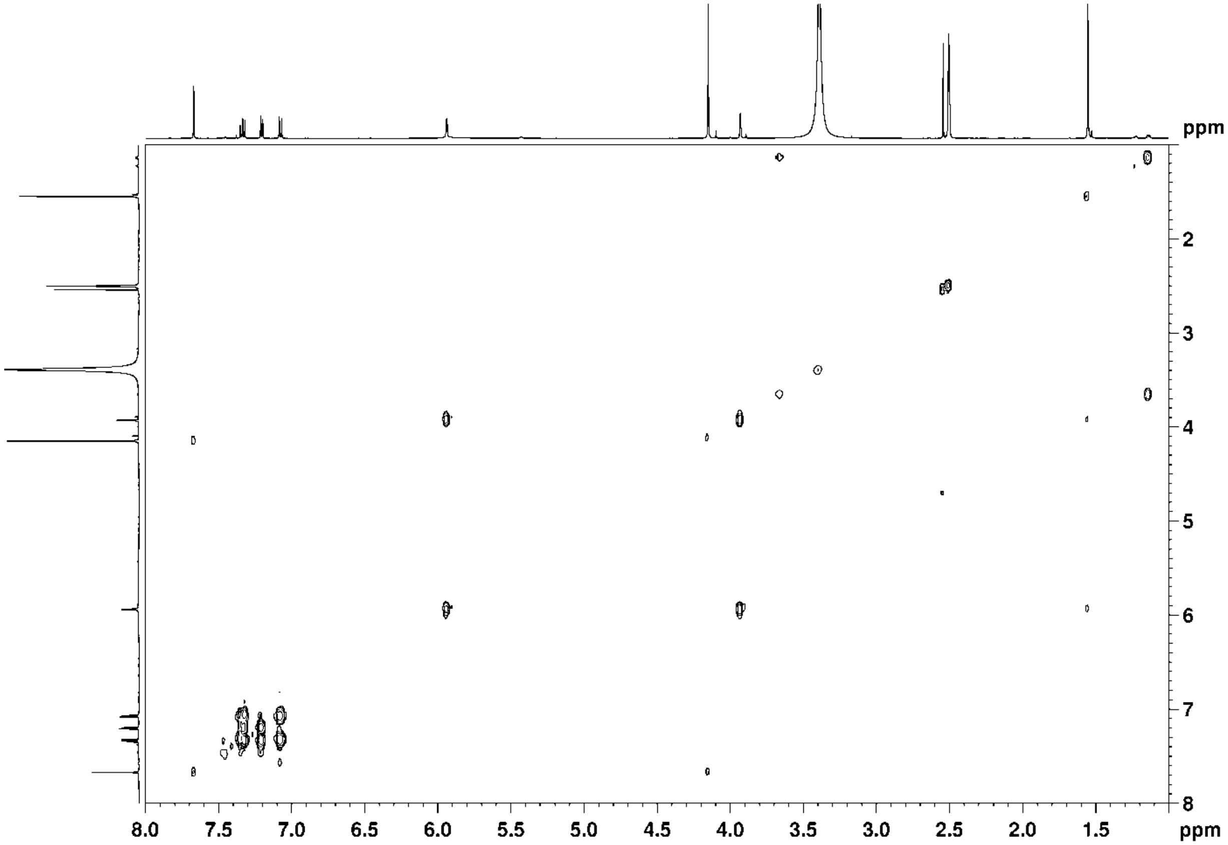

Supplementary Figure 112. The COSY spectrum of compound **30** in DMSO-d_6. **a** COSY correlations are indicated by boldface bonds. **b** The COSY spectrum.

a **b**

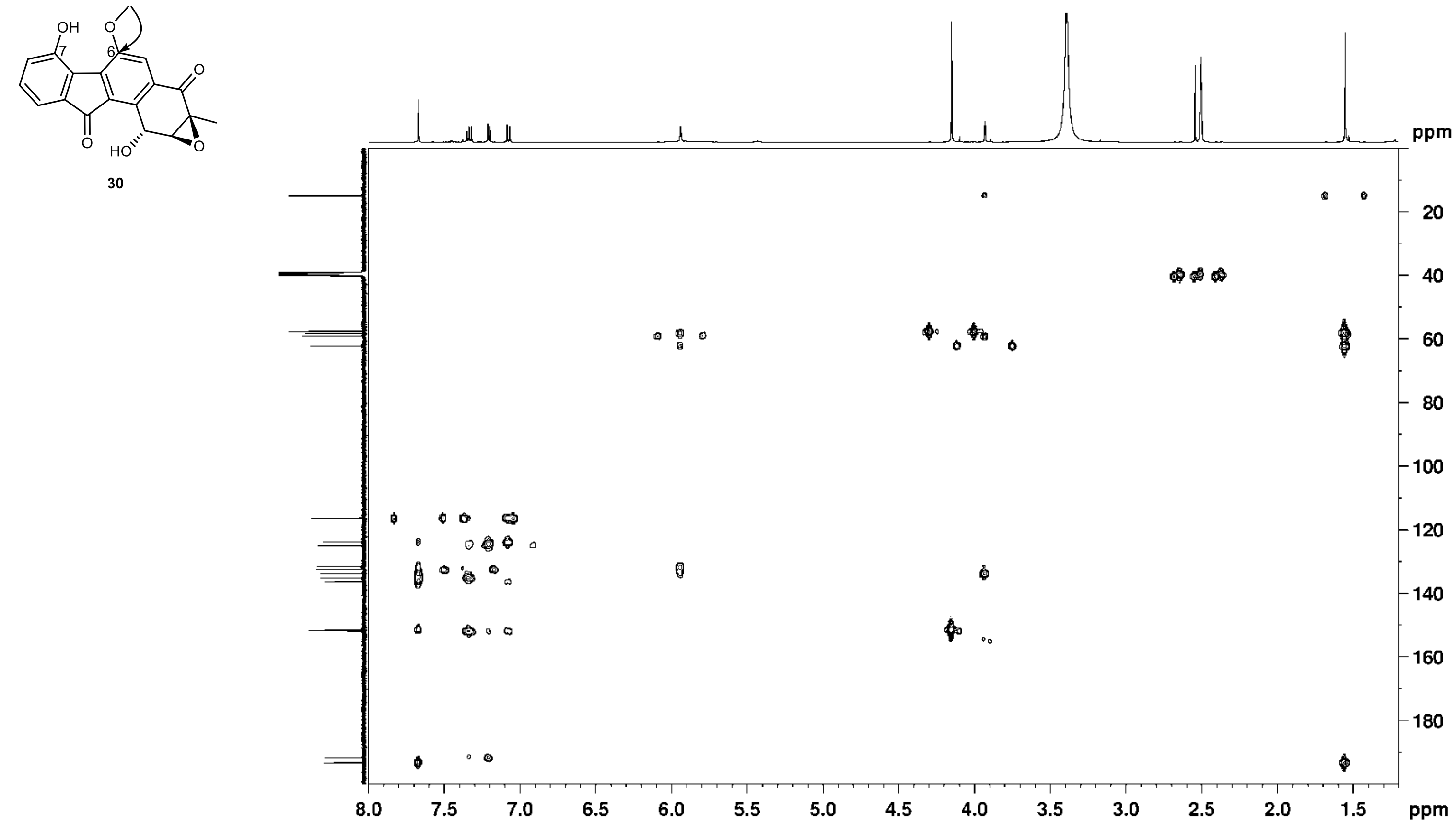

Supplementary Figure 113. The HMBC spectrum of compound **30** in DMSO-d_6. **a** Selected key HMBC correlations are indicated by the curved arrows. **b** The HMBC spectrum.

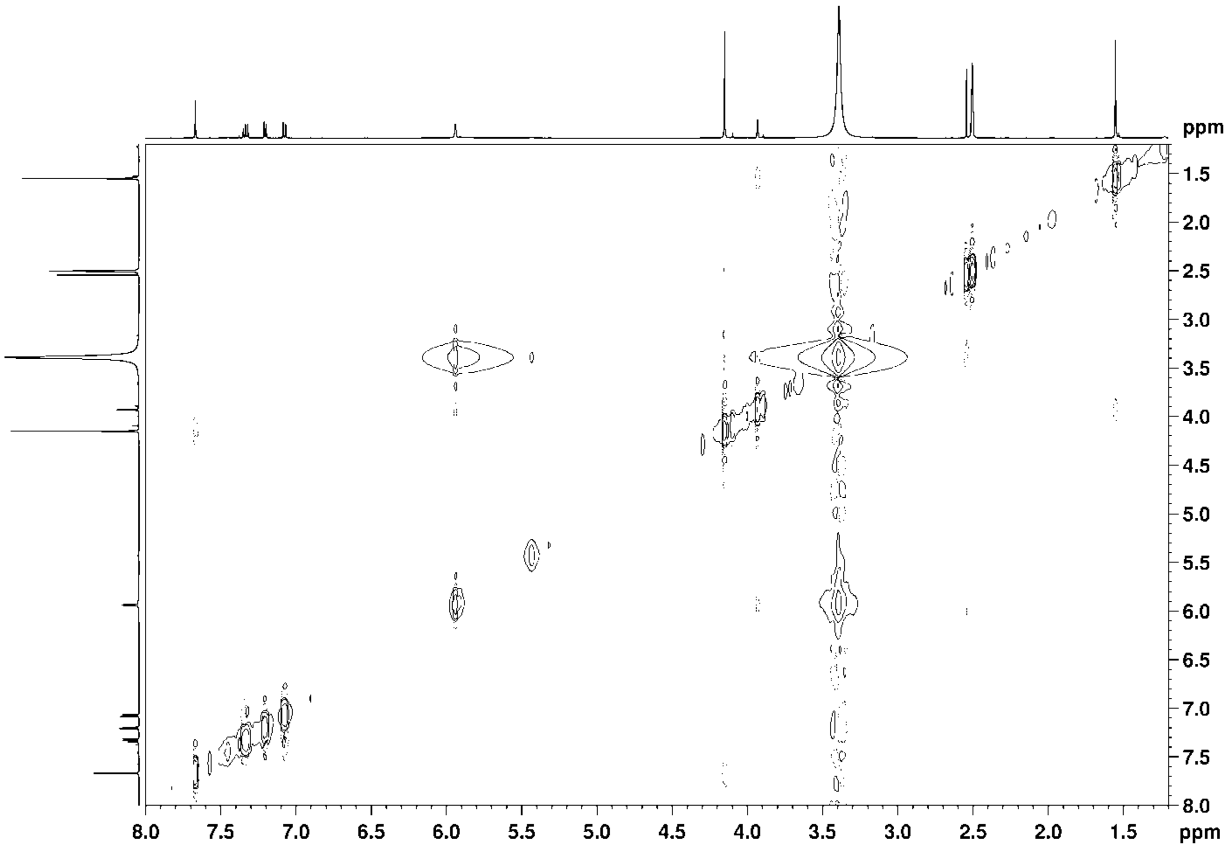

Supplementary Figure 114. The NOESY spectrum of compound **30** in DMSO-d_6.

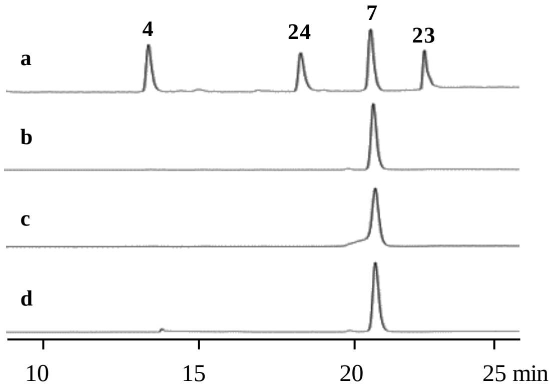

Supplementary Figure 115. Comparison of stability of FST D (7) in H_2O and aprotic organic solvents. **a** H_2O; **b** dimethyl sulfoxide (DMSO); **c** acetone; **d** chloroform. FST D (**7**, 10 µM) was incubated in different solvents (50 µL) overnight at 30 ºC.

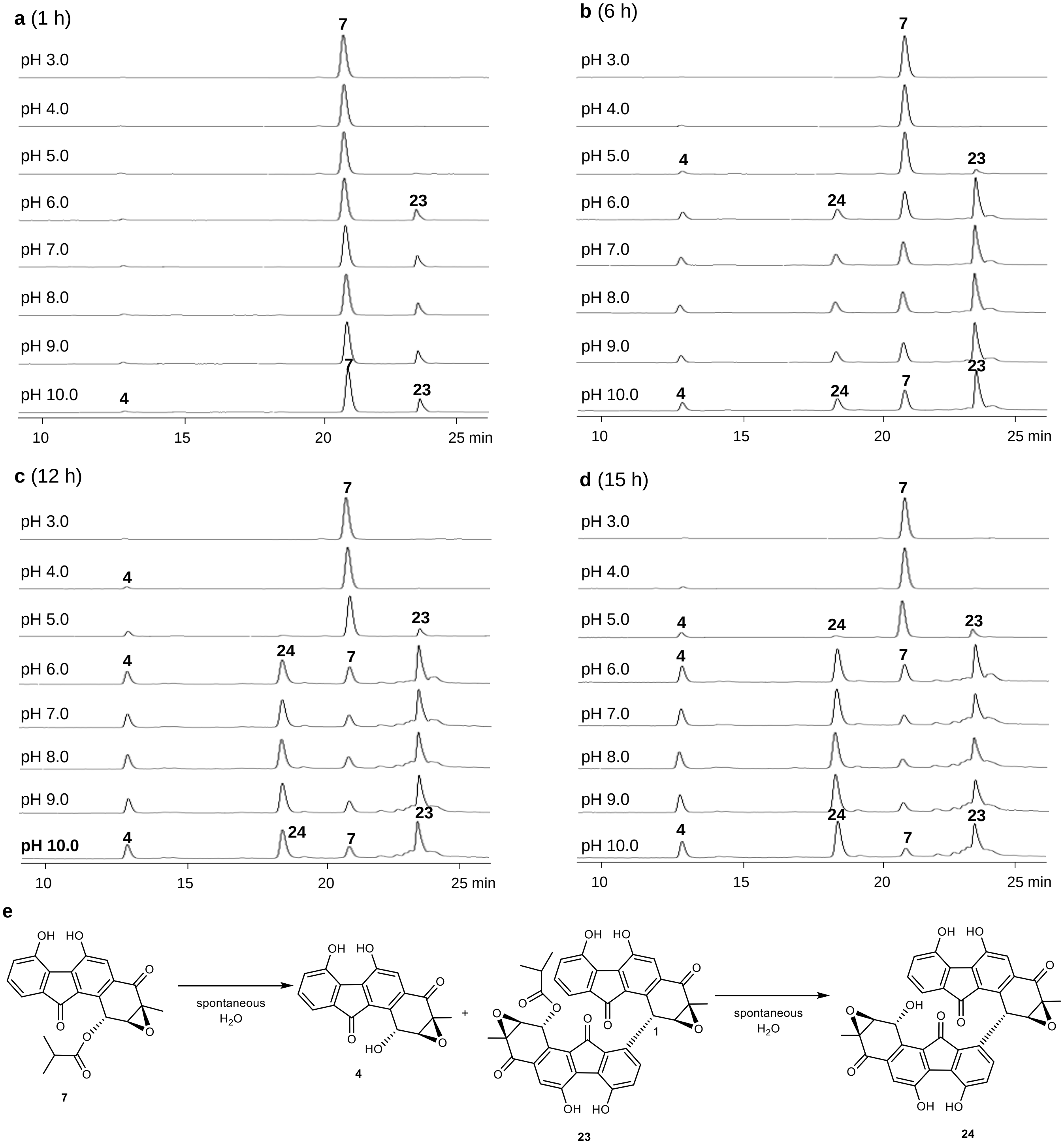

Supplementary Figure 116. The pH effects on the spontaneous deacylation and dimerization. HPLC analysis of spontaneous deacylation and dimerization of FST D (**7**) in PBS (phosphate buffer saline) with different pH values at 30 ºC for **a** 1 h, **b** 6 h, **c** 12 h, and **d** 15 h, and **e** a reaction scheme for the spontaneous conversion of **7** to **4**, **23** and **24** in H_2O.

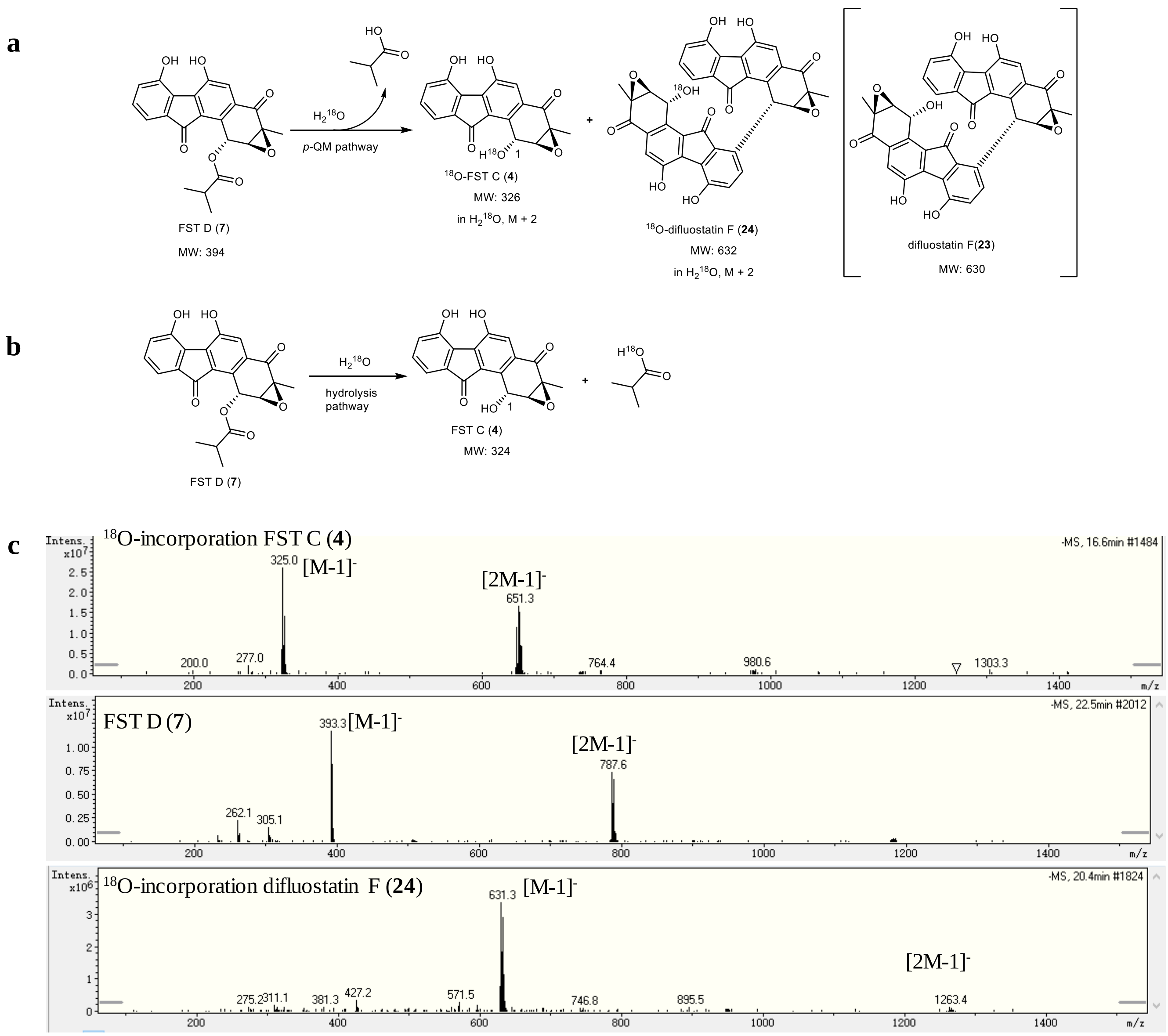

Supplementary Figure 117. LC-MS analysis of products of reaction of FST D (7) in $H_2{}^{18}O$. The proposed ^{18}O incorporation pattern of products when FST D (**7**) is incubated in $H_2{}^{18}O$ at room temperature for overnight via: **a** the *p*-QM pathway, or **b** the hydrolysis pathway; **c** LC-MS analysis of the products of incubation of FST D (**7**) in $H_2{}^{18}O$.

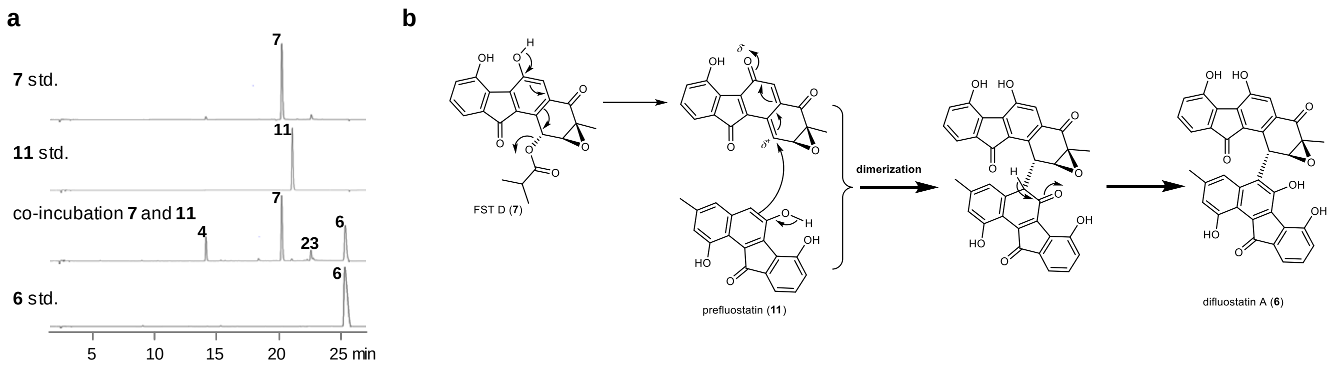

Supplementary Figure 118. Synthesis of difluostatin A (6) by co-incubation of 7 and 11. a HPLC analysis of the coupling reaction. **b** Proposed mechanism for the coupling product. Prefluostatin (**11**, 0.01 mmol, 3.0 mg) and FST D (**7**, 0.02 mmol, 8.0 mg) were mixed in water. After an overnight incubation at room temperature, the mixture was extracted with equal volume of EtOAc and concentrated under vacuum to yield a crude extract. The crude extract was analyzed by HPLC and LC-MS to indicate a yield of **6** around 40%, the identity of which was confirmed by co-elution with the standard **6**.

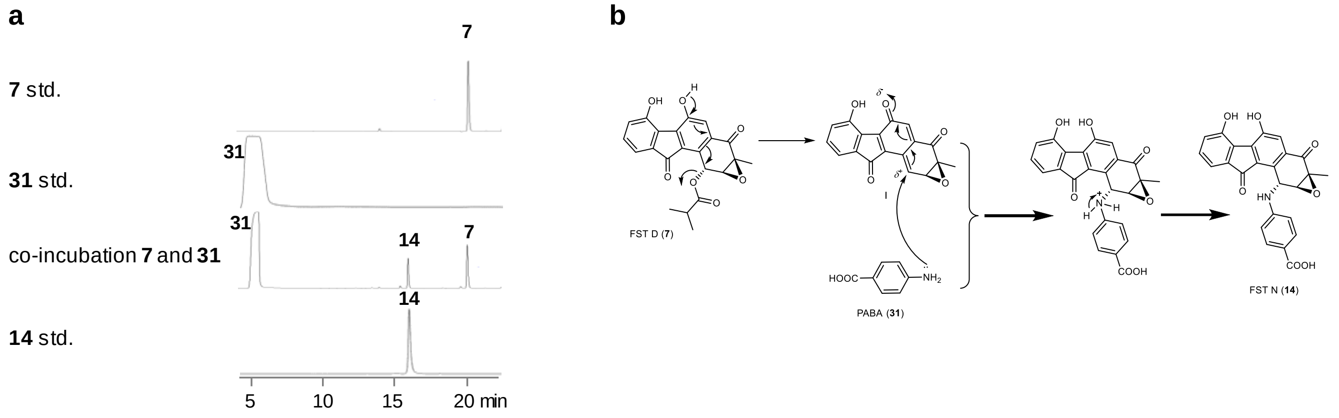

Supplementary Figure 119. Synthesis of FST N (14) by co-incubation of 7 and PABA (31). a HPLC analysis of the coupling reaction. **b** Proposed mechanism for the coupling product. *p*-Aminobenzoic acid (PABA, **31**, 0.04 mmol, 6.0 mg) and FST D (**7**, 0.01 mmol, 4.0 mg) were mixed in water. After an overnight incubation at room temperature, the mixture was extracted with equal volume of EtOAc and concentrated under vacuum to yield a crude extract. The crude extract was analyzed by HPLC and LC-MS to indicate a yield of **14** around 35%, the identity of which was confirmed by co-elution with the standard **14**.

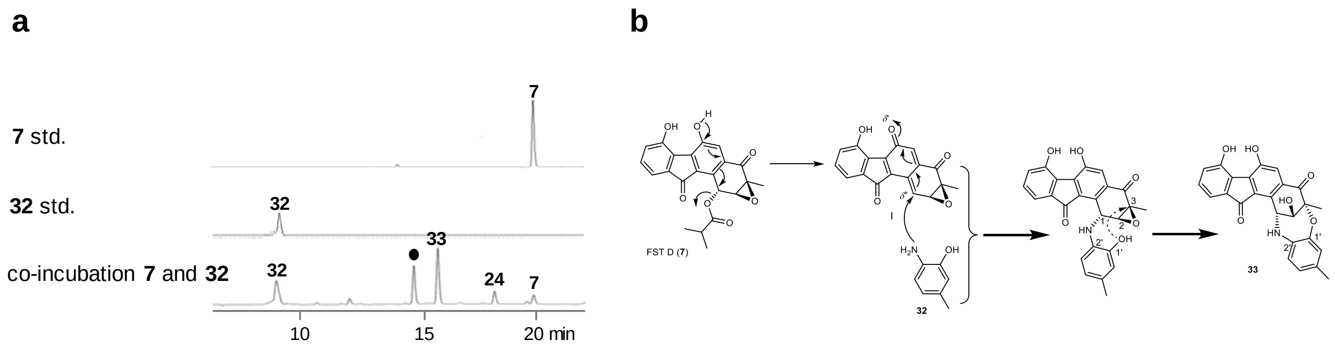

Supplementary Figure 120. Synthesis of compound 33 by co-incubation of 7 and 32. a HPLC analysis of the coupling reaction. **b** Proposed mechanism for the coupling product.The compound 2-amino-5-methylphenol (**32**, 0.16 mmol, 20.0 mg) and **7** (0.04 mmol, 16.0 mg) were co-incubated in water at room temperature for overnight. Then the mixture was extracted with equal volume of EtOAc and concentrated under vacuum to give a crude extract. The crude extract was purified via semi-preparative HPLC to yield **33** (7.8 mg, 45%). The symbol "●" denotes an uncharacterized product.

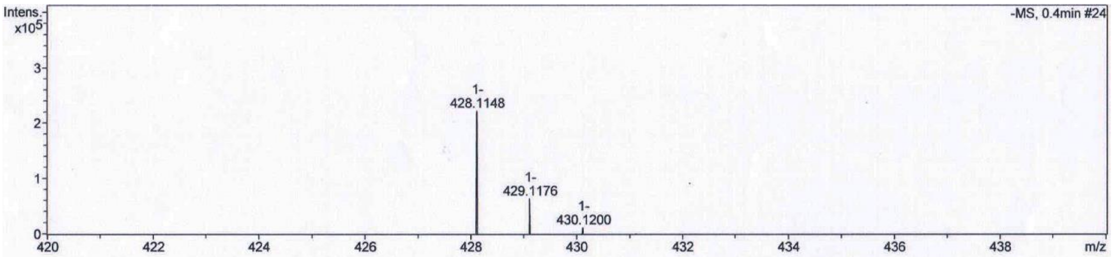

Meas. m/z	#	Ion Formula	Score	m/z	err [ppm]	err [mDa]	mSigma	rdb	e⁻ Conf	N-Rule
428.114766	1	C25H18NO6	100.00	428.113961	-1.9	-0.8	6.1	17.5	even	ok
857.234651	1	C50H37N2O12	100.00	857.235198	-0.6	-0.5	39.5	33.5	even	ok

Supplementary Figure 121. HRESIMS spectrum of compound **33**.

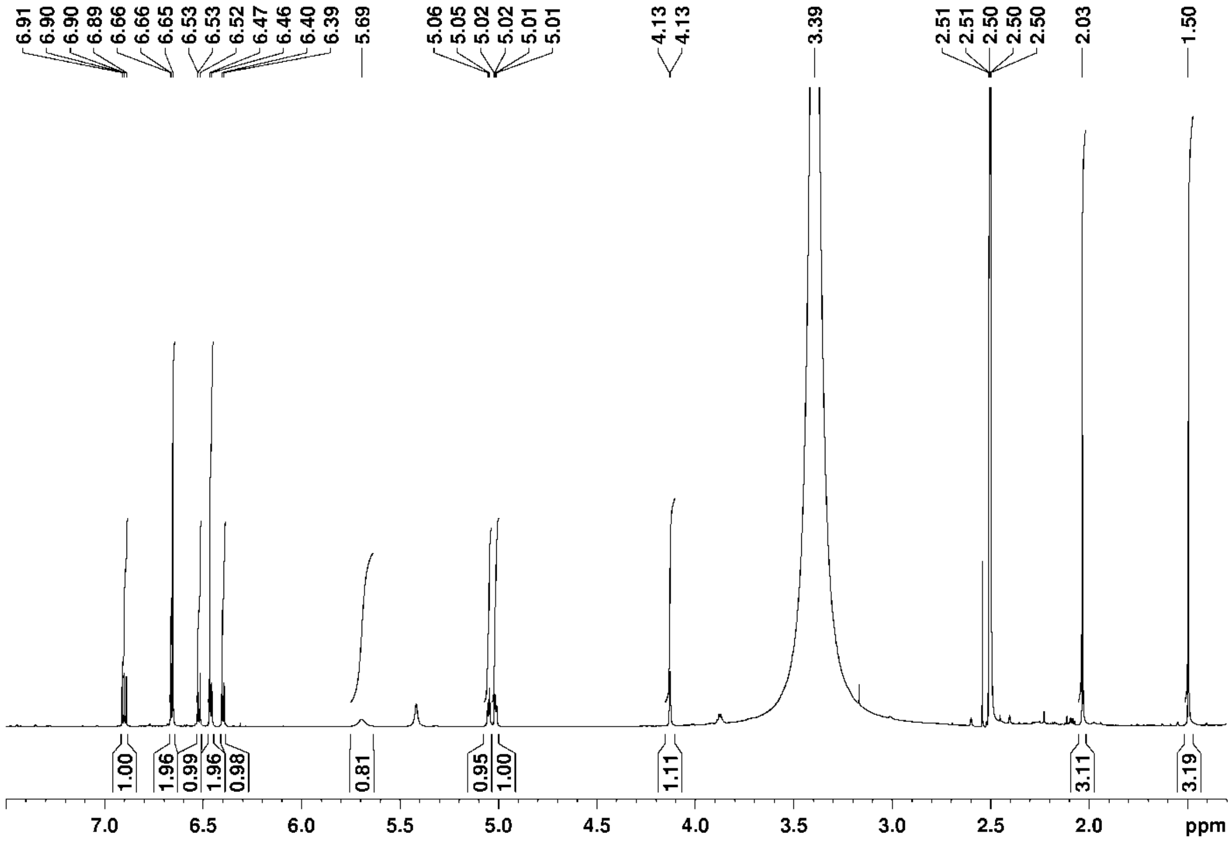

Supplementary Figure 122. The ^{1}H NMR spectrum of compound **33** in DMSO-d_6.

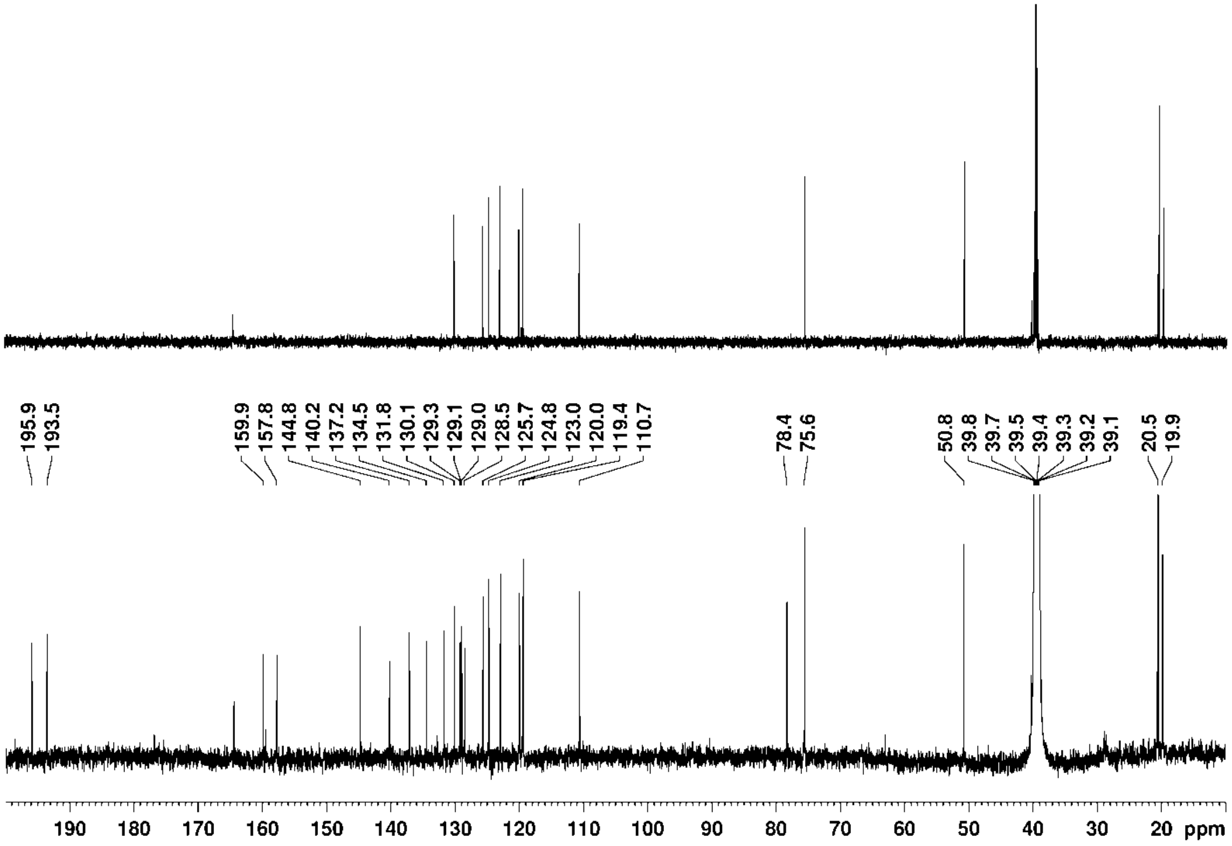

Supplementary Figure 123. The ^{13}C and DEPT 135 NMR spectrum of compound **33** in DMSO-d_6.

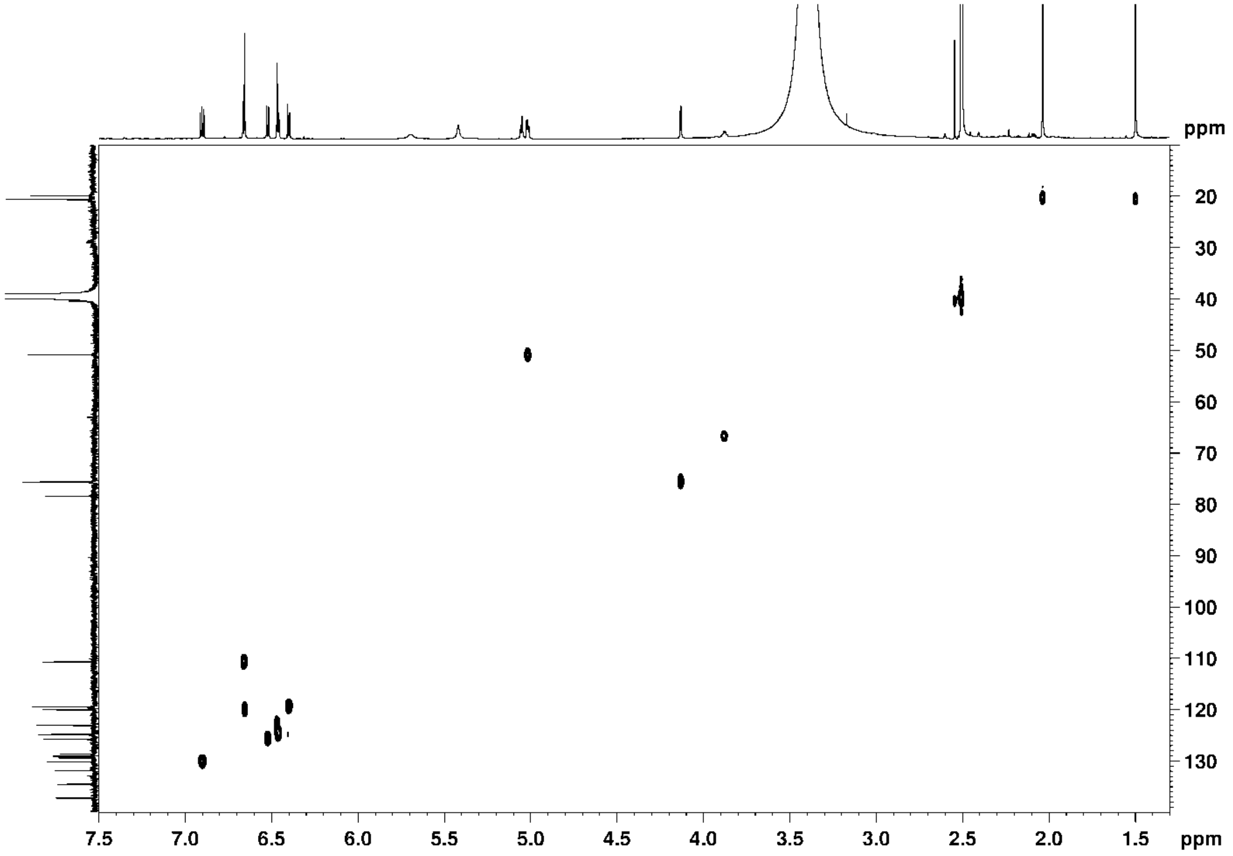

Supplementary Figure 124. The HSQC spectrum of compound **33** in DMSO-d_6.

a

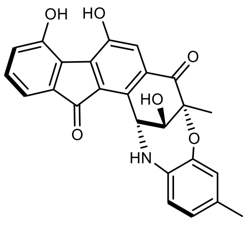

33

b

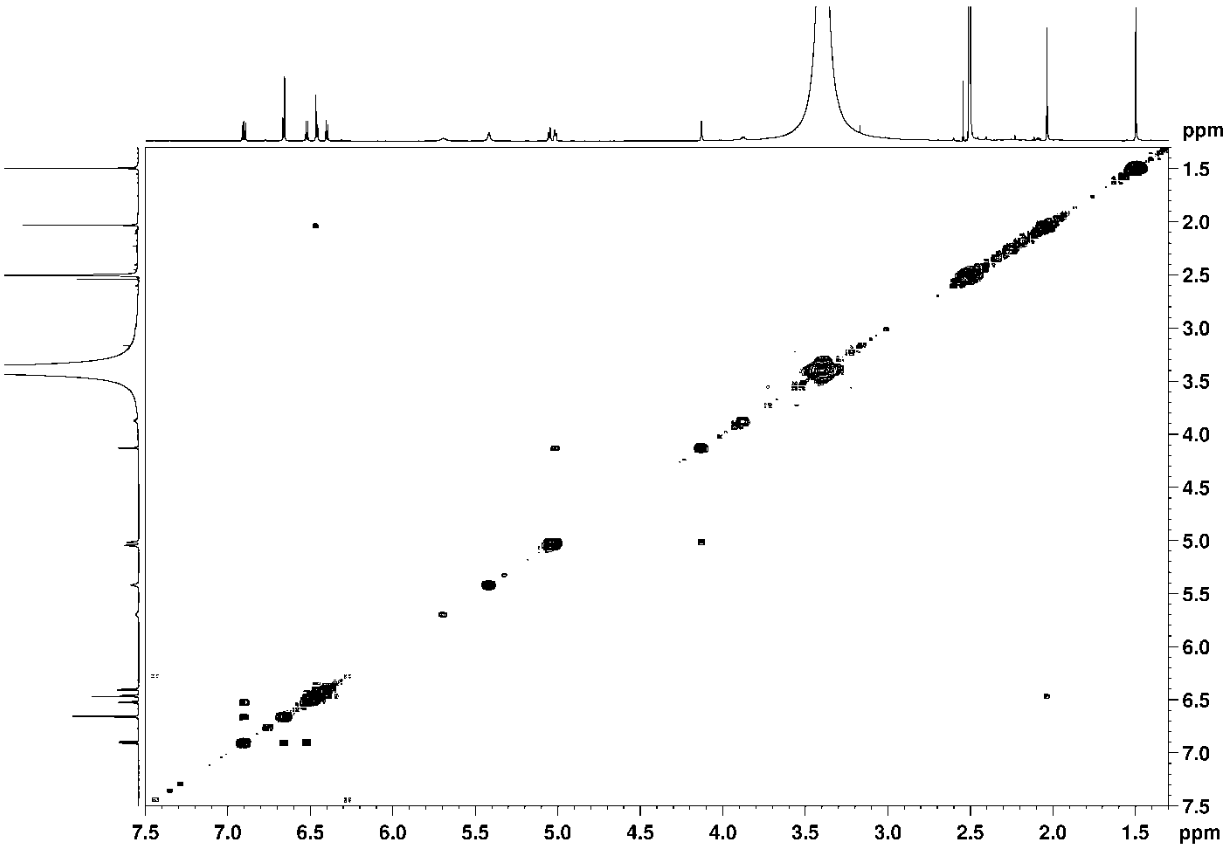

Supplementary Figure 125. The COSY spectrum of compound **33** in DMSO-d_6. **a** COSY correlations are indicated by boldface bonds. **b** The COSY spectrum.

a

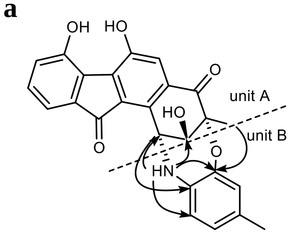

33

b

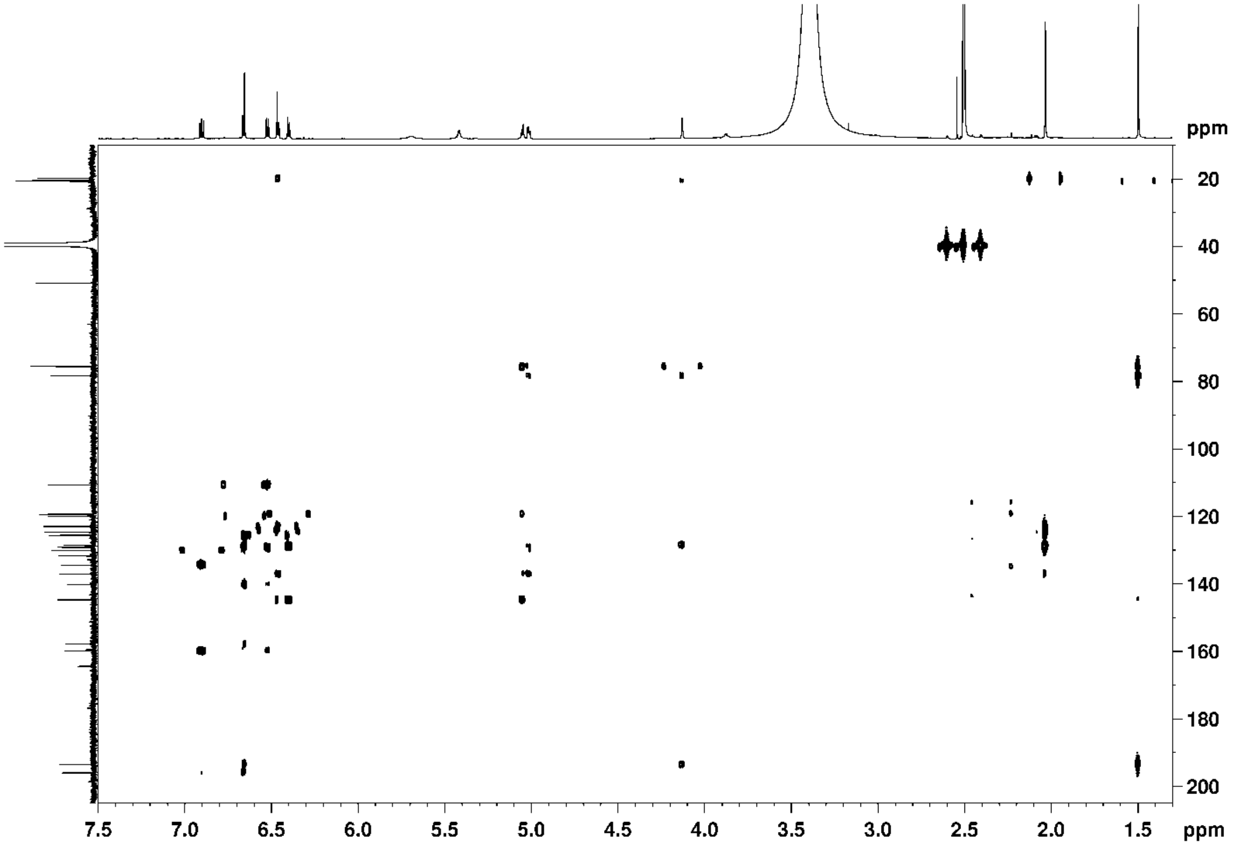

Supplementary Figure 126. The HMBC spectrum of compound **33** in DMSO-d_6. **a** Selected key HMBC correlations are indicated by the curved arrows. **b** The HMBC spectrum.

a

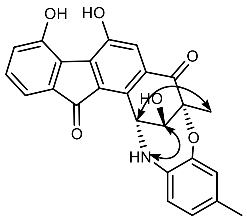

33

b

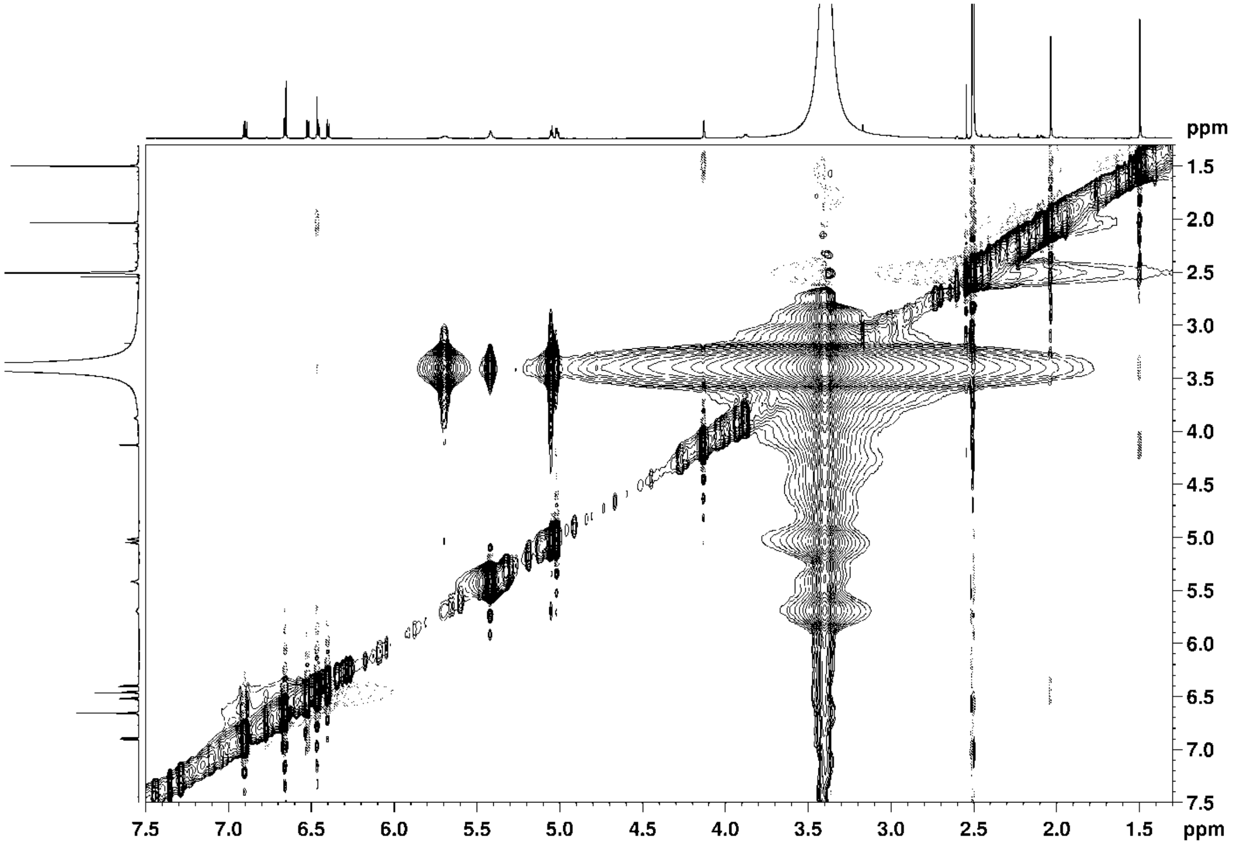

Supplementary Figure 127. The NOESY spectrum of compound **33** in DMSO-d_6. **a** Selected key NOESY correlations are indicated by the curved, double-headed arrows. **b** The NOESY spectrum.

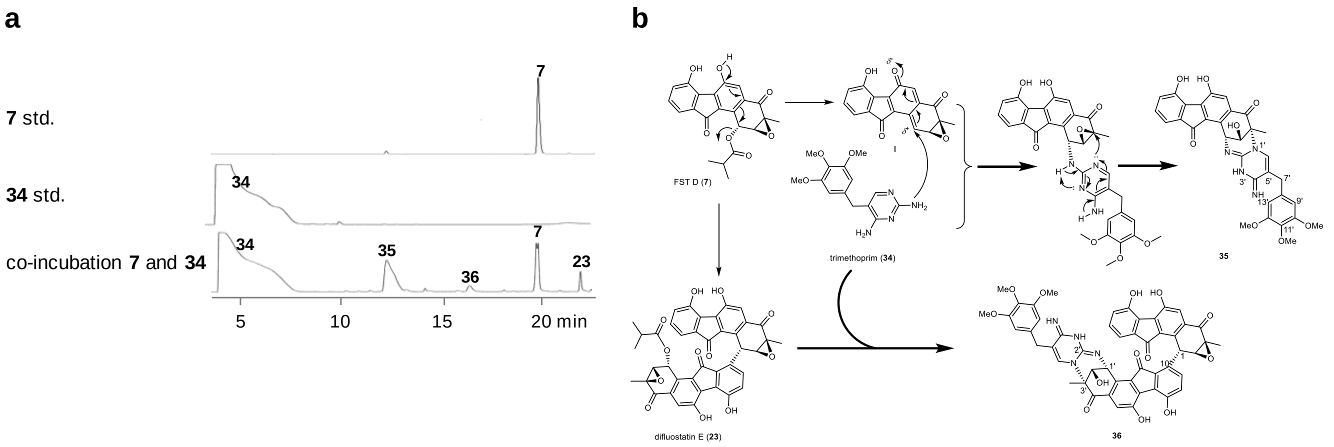

Supplementary Figure 128. Synthesis of 35 and 36 by co-incubation of 7 and trimethoprim (34). a HPLC analysis of the coupling reaction. **b** Proposed mechanism for the coupling products. Trimethoprim (**34**, 0.16 mmol, 47.0 mg) and **7** (0.04 mmol, 16.0 mg) were co-incubated in water at room temperature for overnight. Then the mixture was extracted with equal volume of EtOAc and concentrated under vacuum to give a crude extract. The crude extract was purified via semi-preparative HPLC to afford compounds **35** (14.0 mg, 59%) and **36** (4.0 mg, 22%).

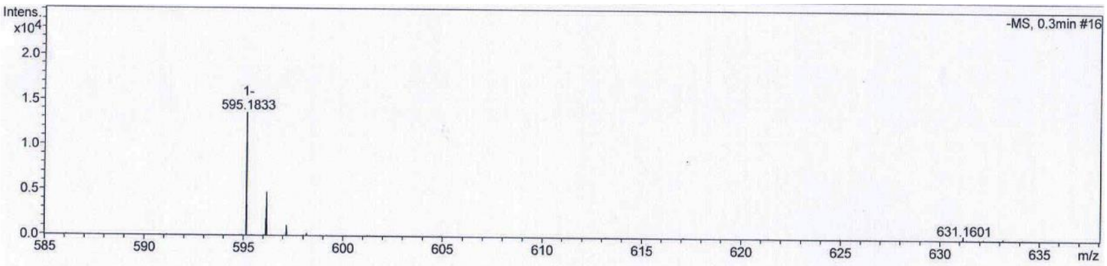

Meas. m/z	#	Ion Formula	Score	m/z	err [ppm]	err [mDa]	mSigma	rdb	e¯ Conf	N-Rule
595.183322	1	C32H27N4O8	100.00	595.183437	0.2	0.1	6.6	21.5	even	ok
631.160097	1	C32H28ClN4O8	100.00	631.160115	0.0	0.0	43.1	20.5	even	ok

Supplementary Figure 129. HRESIMS spectrum of compound **35**.

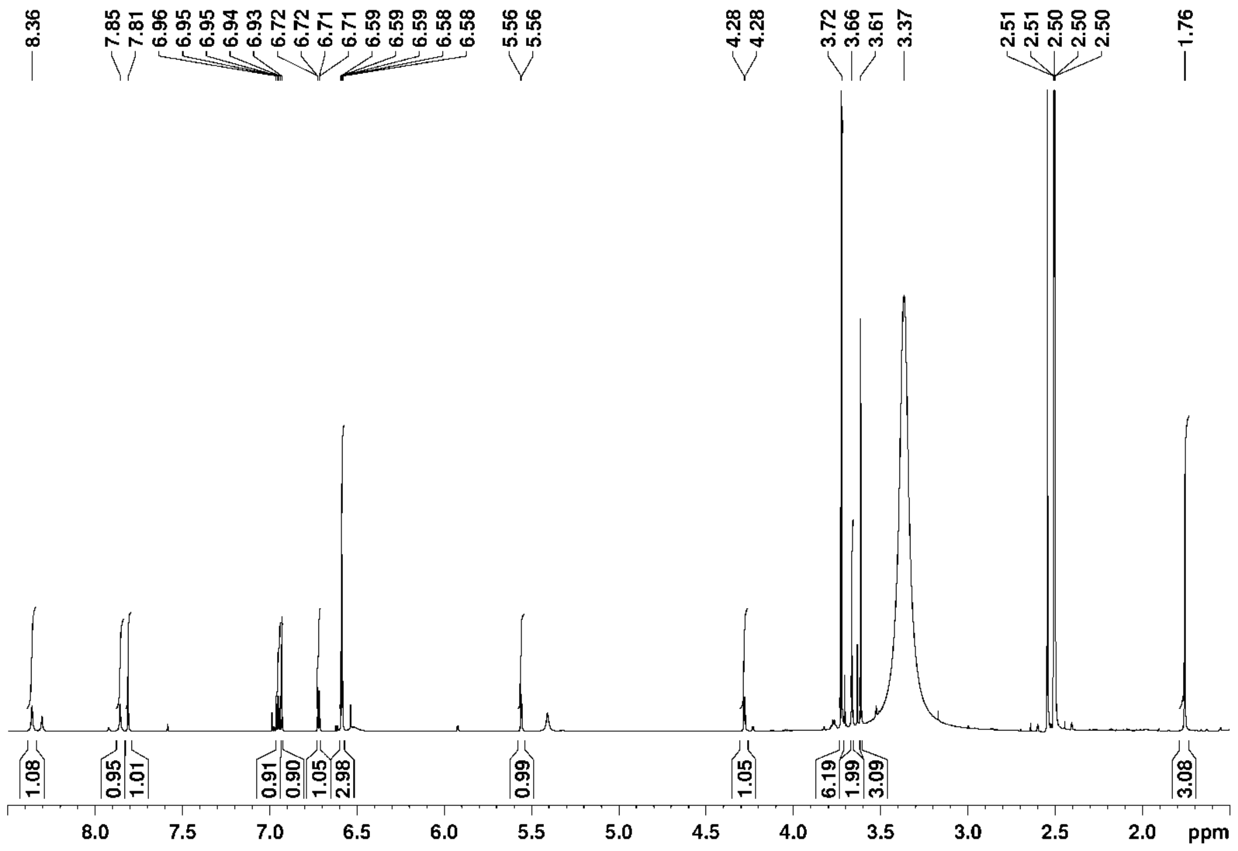

Supplementary Figure 130. The ^{1}H NMR spectrum of compound **35** in DMSO-d_6.

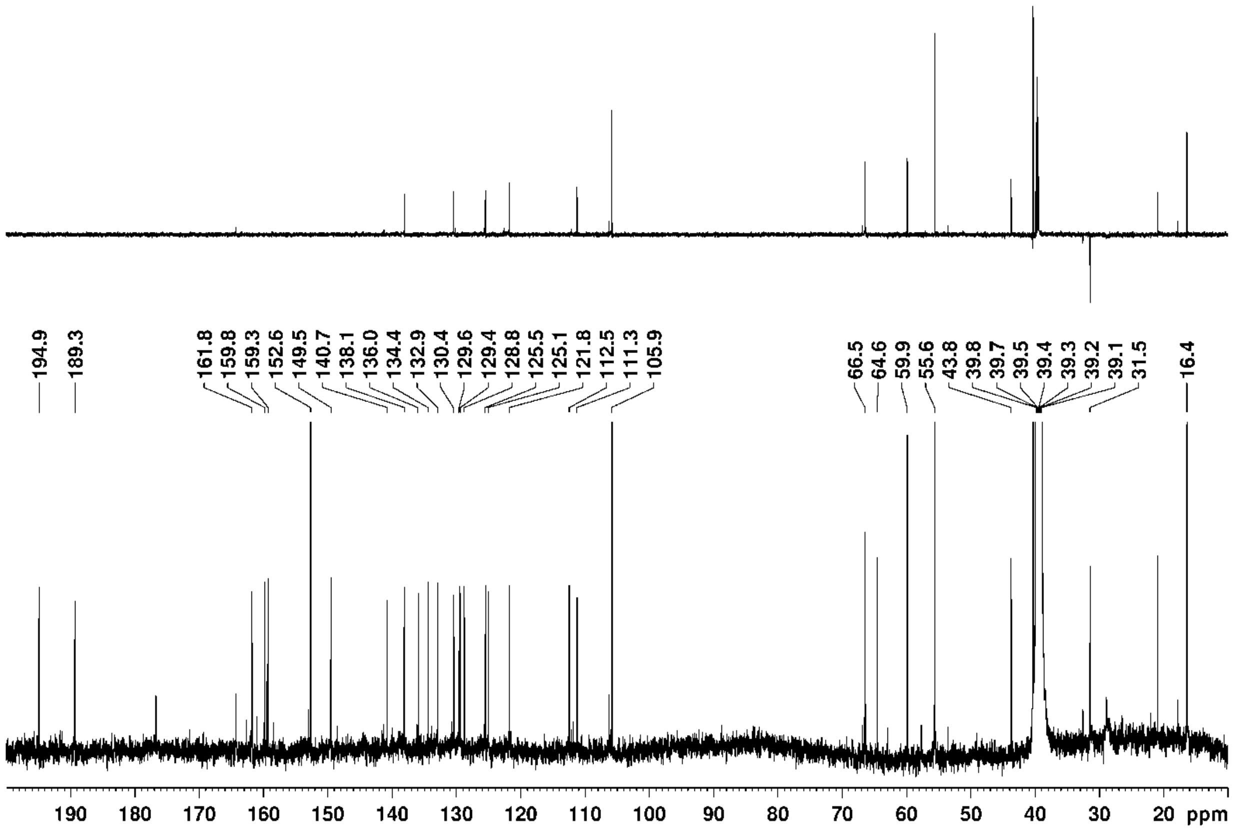

Supplementary Figure 131. The ^{13}C and DEPT 135 NMR spectrum of compound **35** in DMSO-d_6.

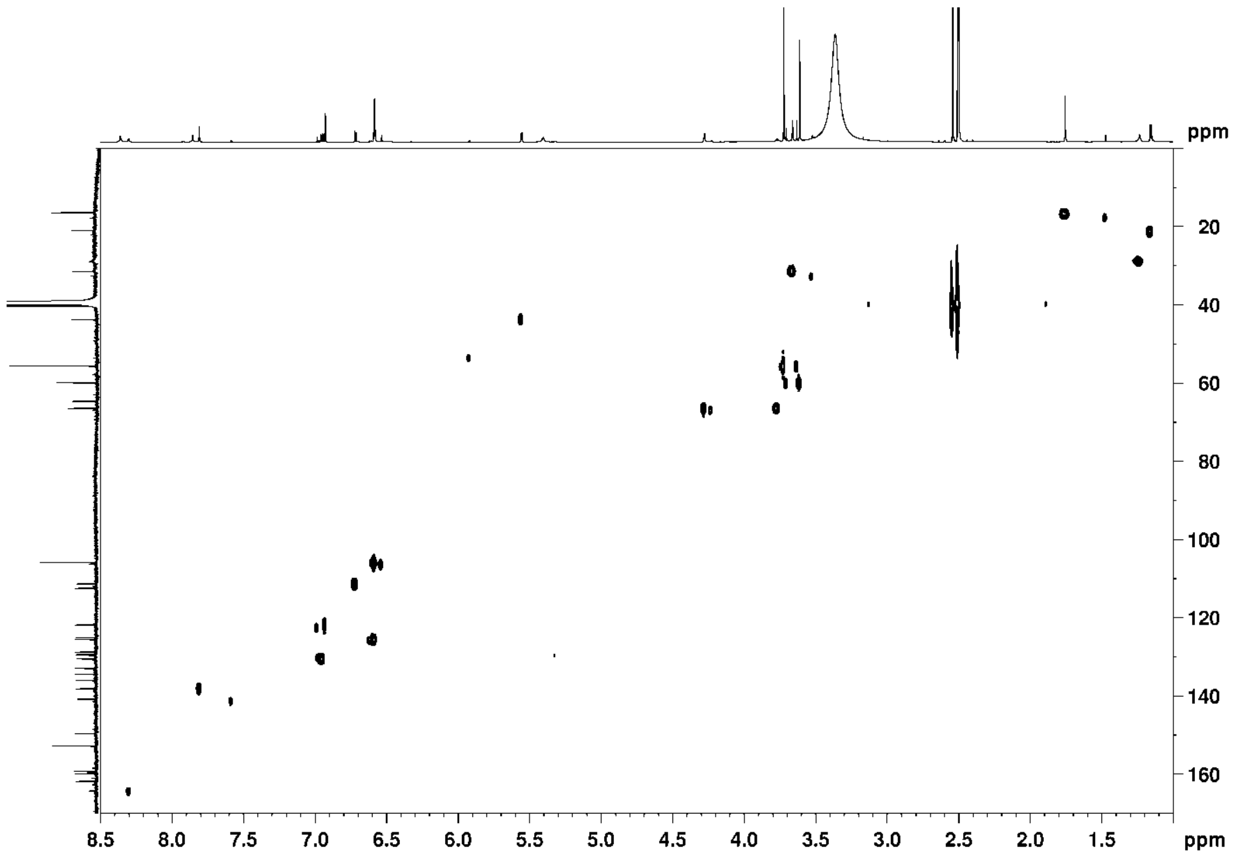

Supplementary Figure 132. The HSQC spectrum of compound **35** in DMSO-d_6.

a

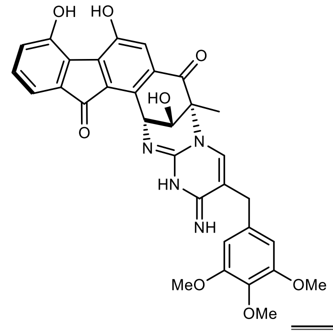

35

b

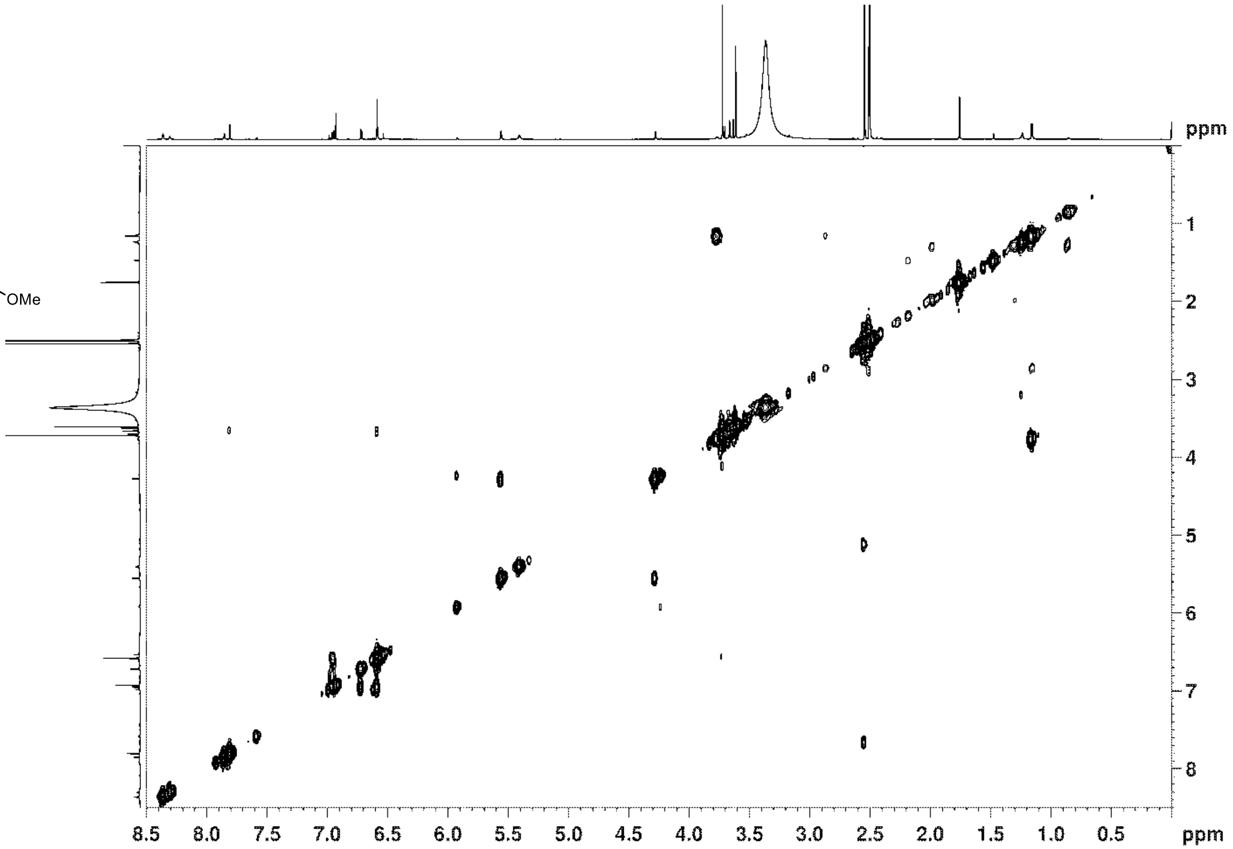

Supplementary Figure 133. The COSY spectrum of compound **35** in DMSO-d_6. **a** COSY correlations are indicated by boldface bonds. **b** The COSY spectrum.

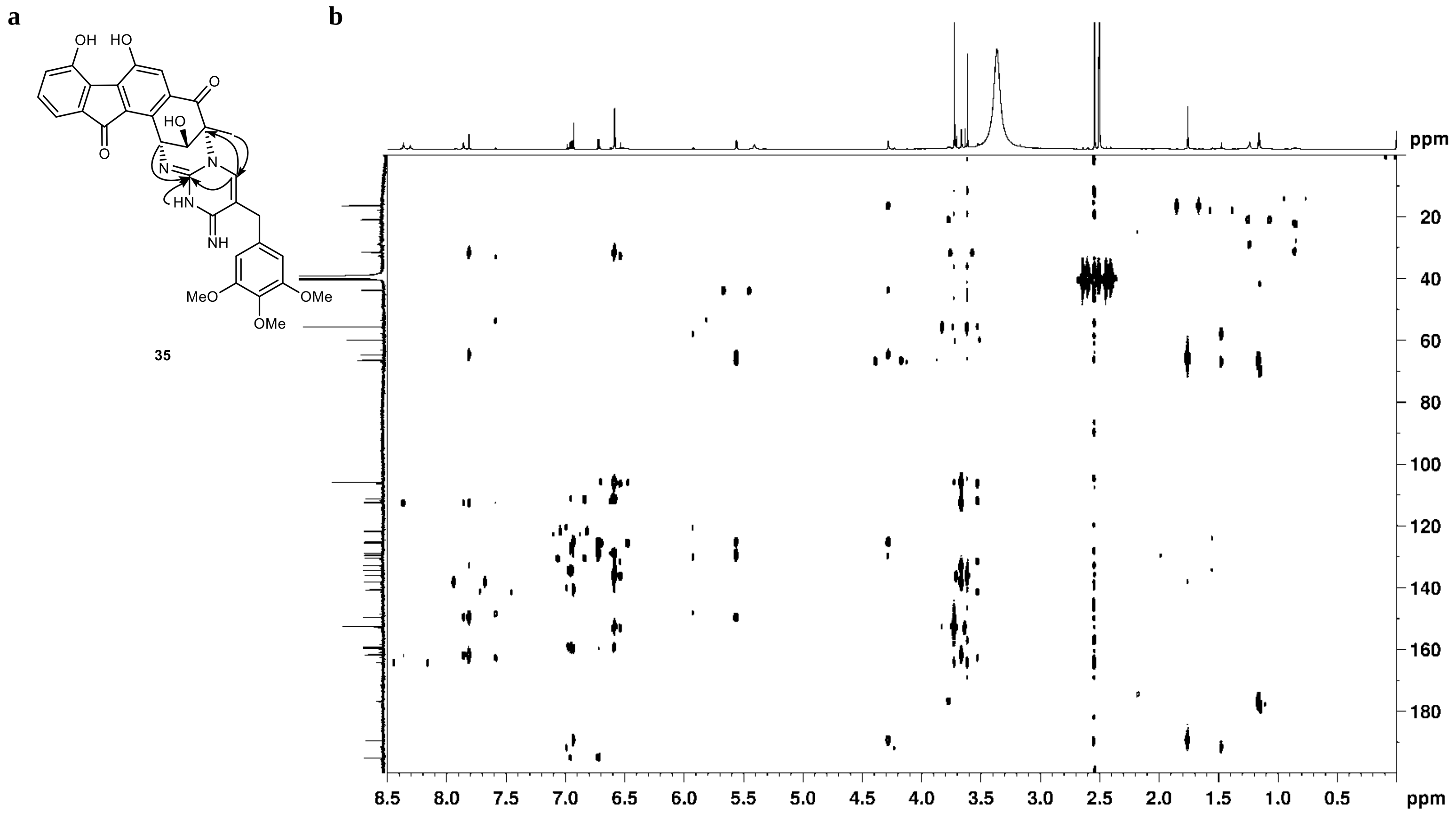

Supplementary Figure 134. The HMBC spectrum of compound **35** in DMSO-d_6. **a** Selected key HMBC correlations are indicated by the curved arrows. **b** The HMBC spectrum.

a

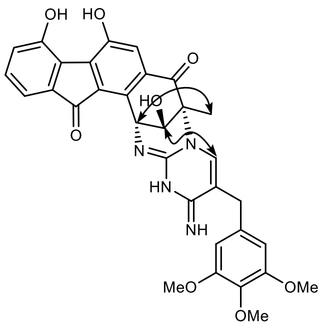

35

b

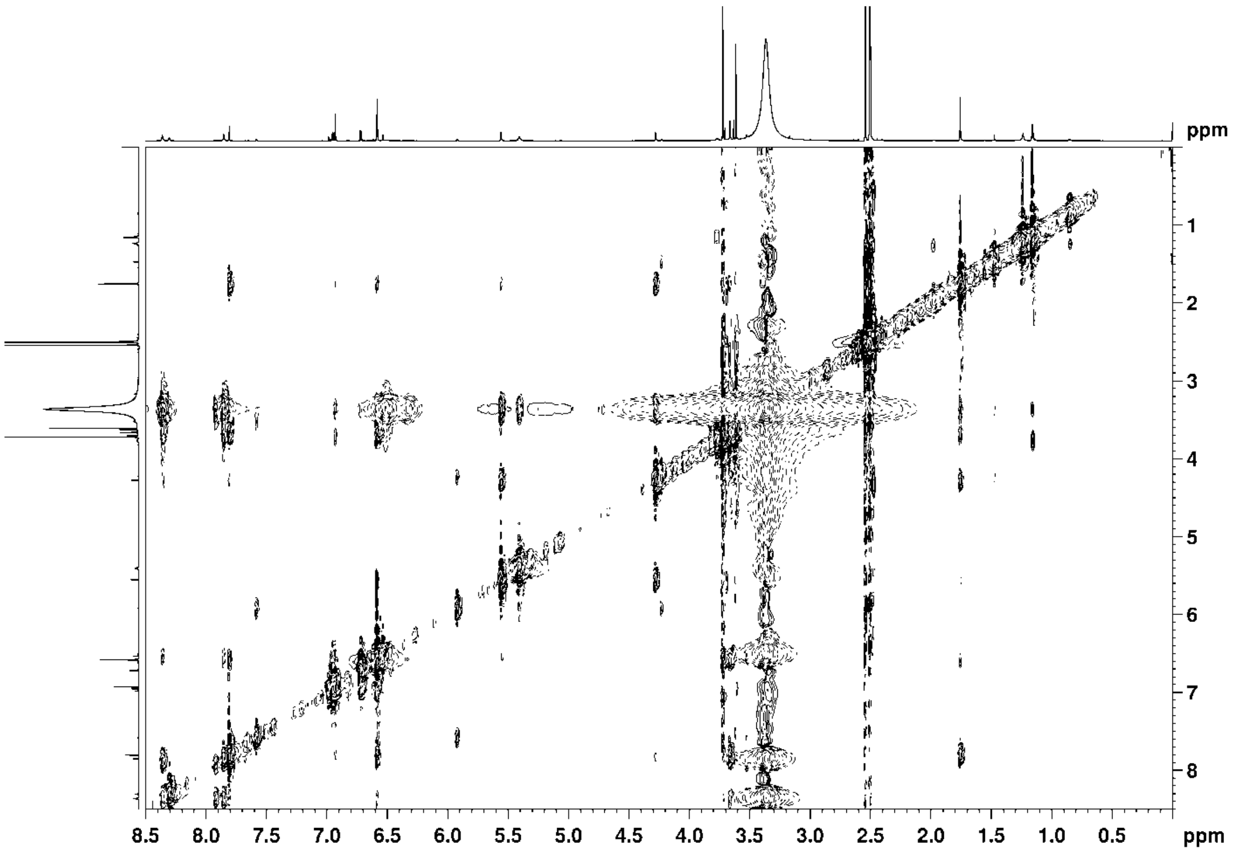

Supplementary Figure 135. The NOESY spectrum of compound **35** in DMSO-d_6. **a** Selected key NOESY correlations are indicated by the curved, double-headed arrows. **b** The NOESY spectrum.

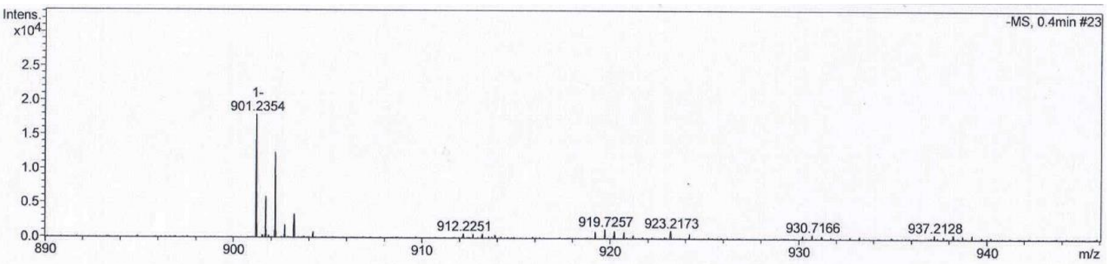

Meas. m/z	#	Ion Formula	Score	m/z	err [ppm]	err [mDa]	mSigma	rdb	e⁻ Conf	N-Rule
901.235440	1	C50H37N4O13	100.00	901.236261	0.9	0.8	58.2	34.5	even	ok
937.212795	1	C50H38ClN4O13	100.00	937.212939	0.2	0.1	301.5	33.5	even	ok

Supplementary Figure 136. HRESIMS spectrum of compound **36**.

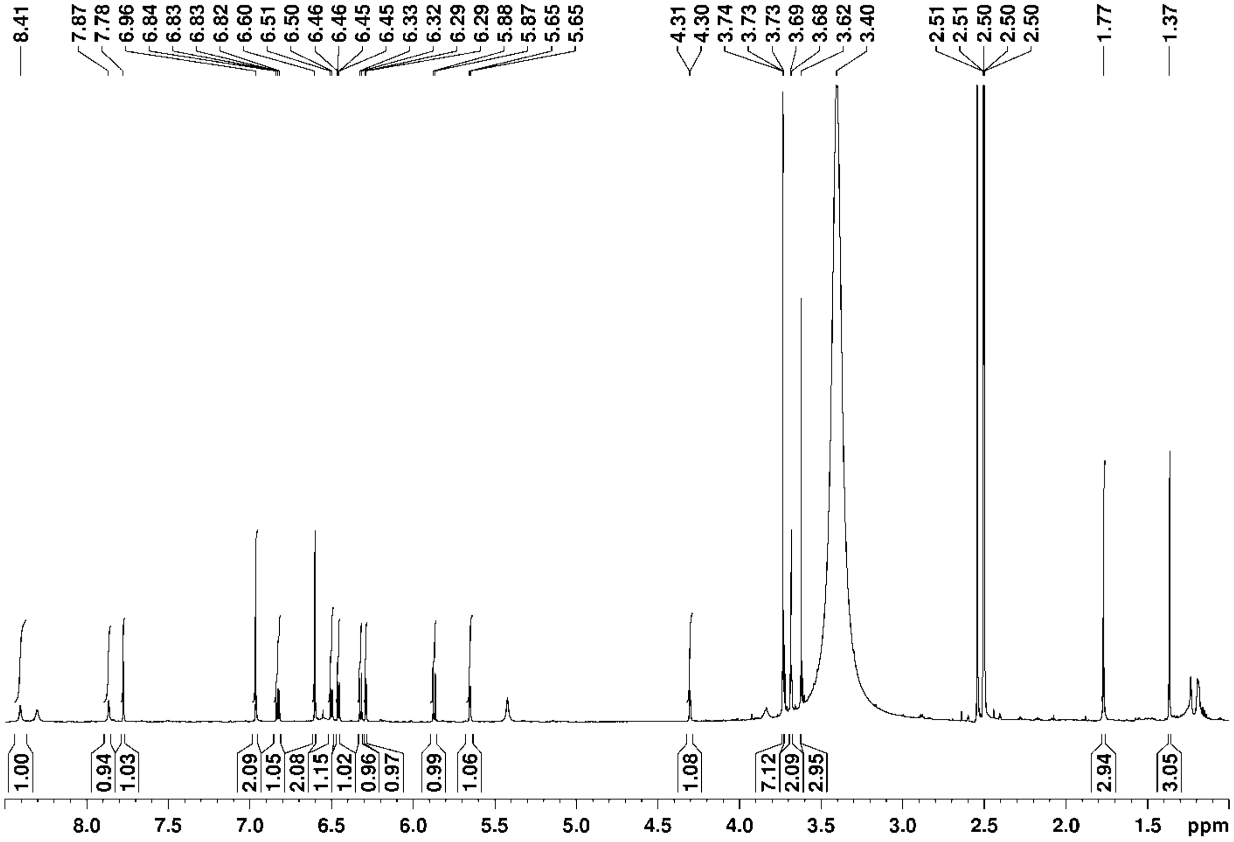

Supplementary Figure 137. The ^{1}H NMR spectrum of compound **36** in DMSO-d_6.

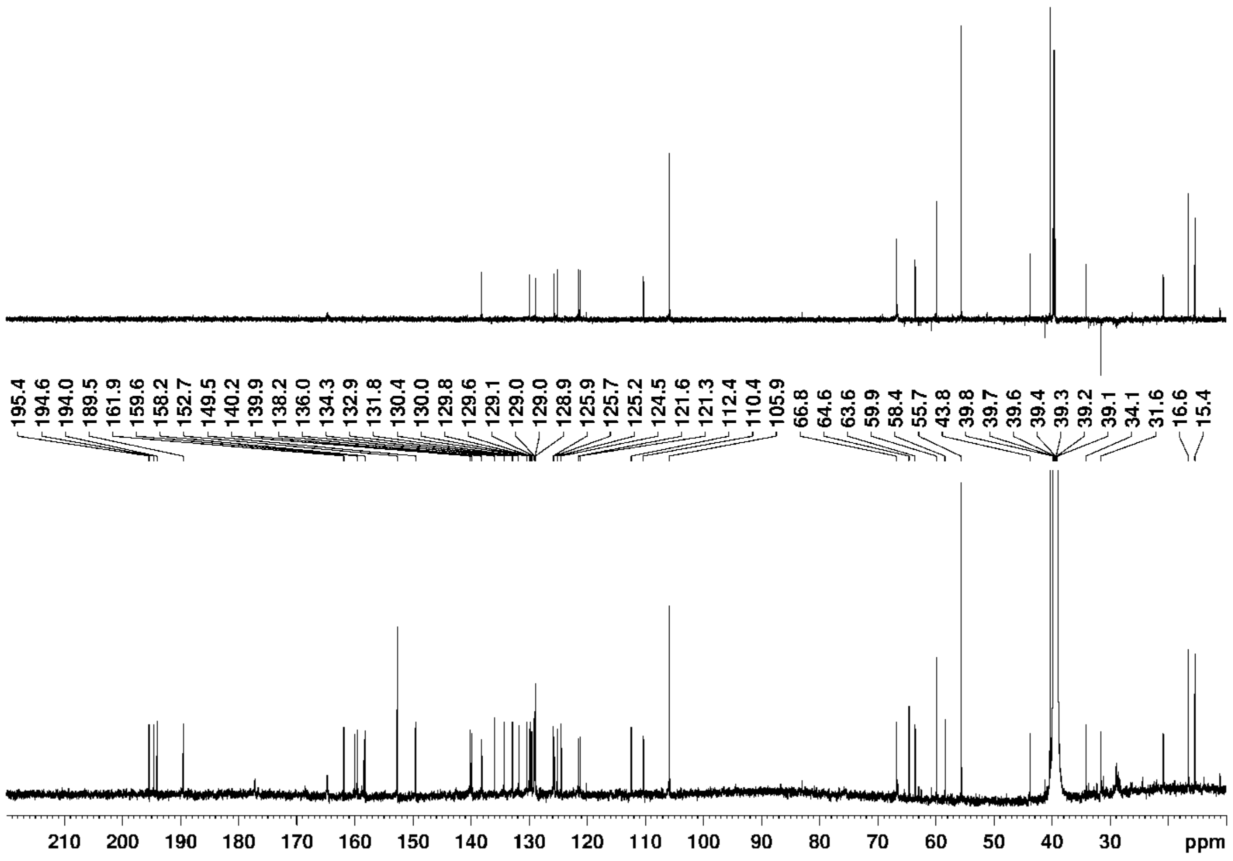

Supplementary Figure 138. The ^{13}C and DEPT 135 NMR spectrum of compound **36** in DMSO-d_6.

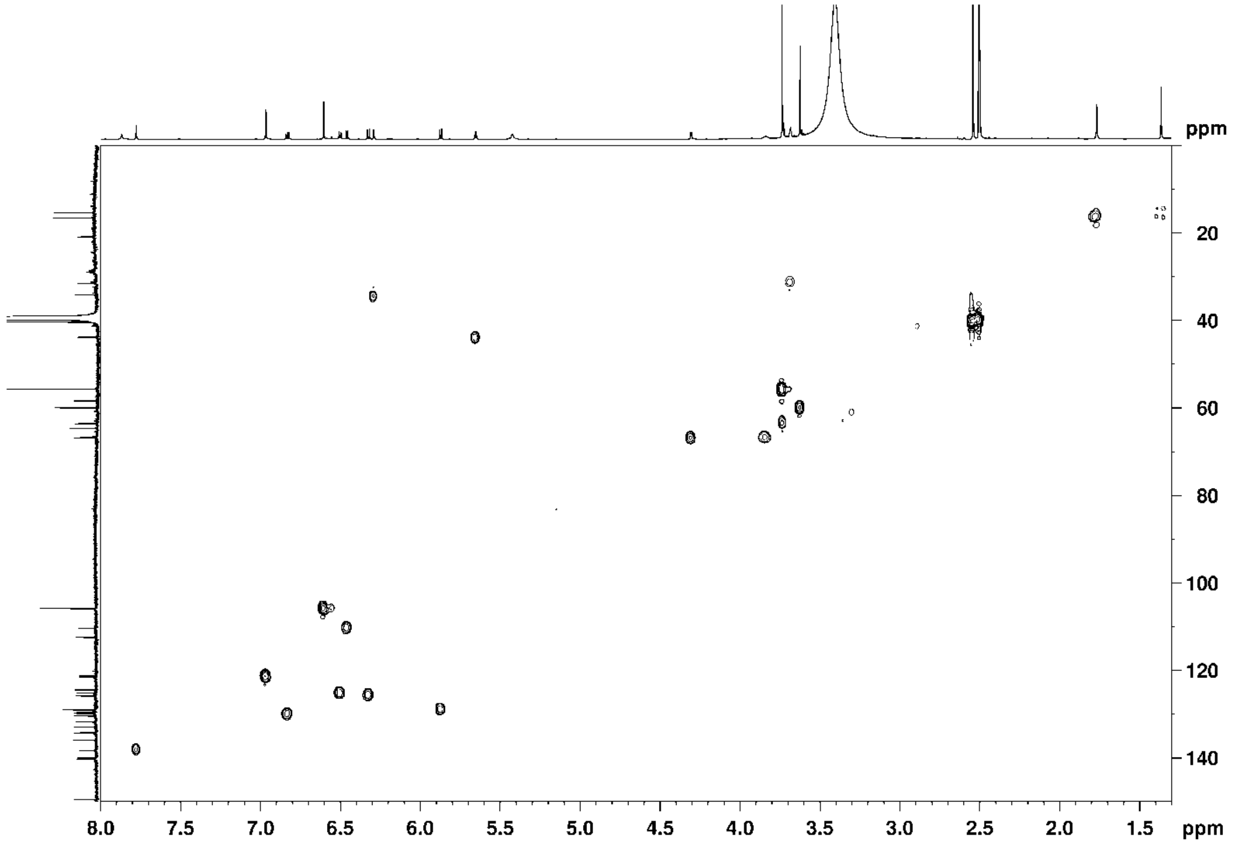

Supplementary Figure 139. The HSQC spectrum of compound **36** in DMSO-d_6.

a **b**

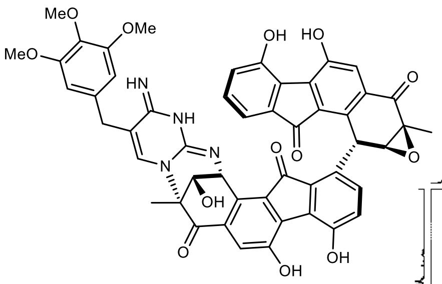

36

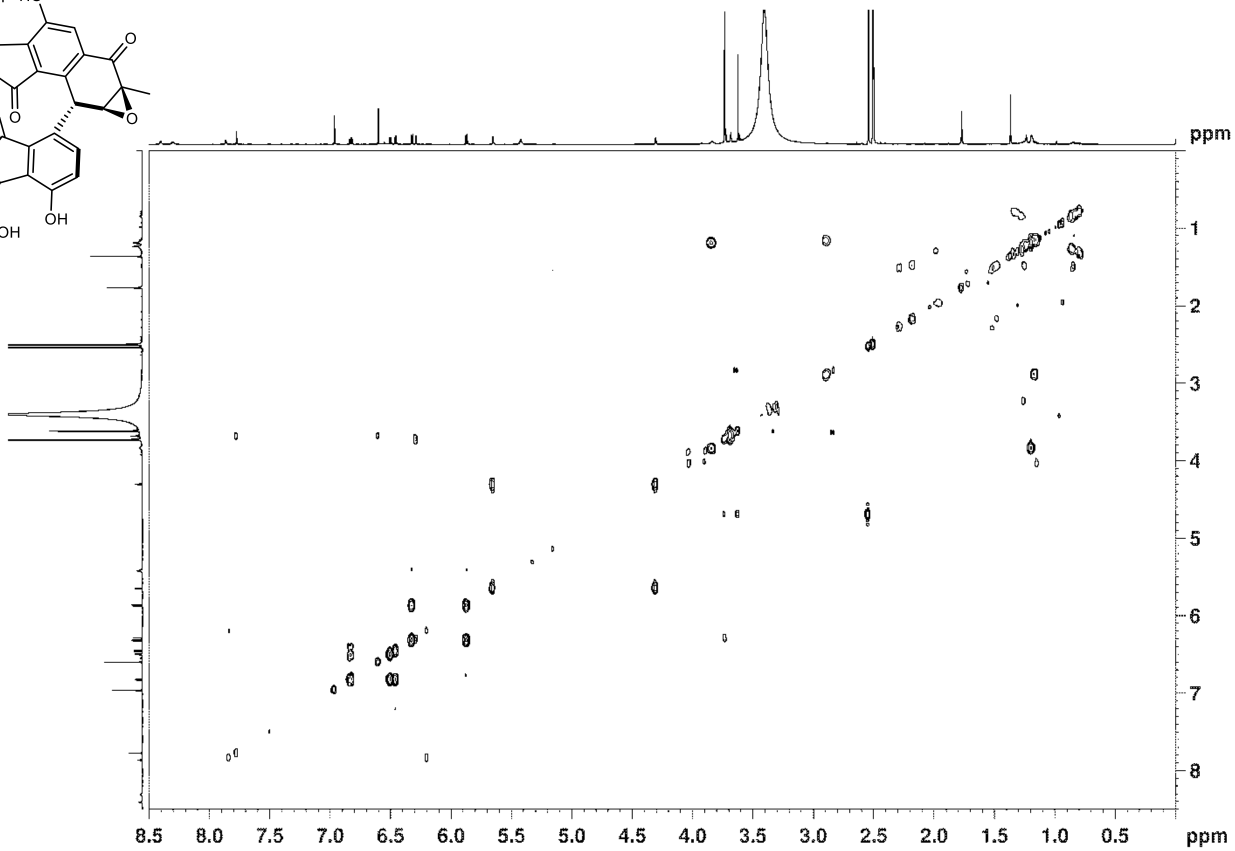

Supplementary Figure 140. The COSY spectrum of compound **36** in DMSO-d_6. **a** COSY correlations are indicated by boldface bonds. **b** The COSY spectrum.

a

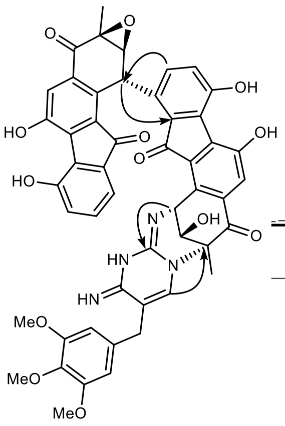

36

b

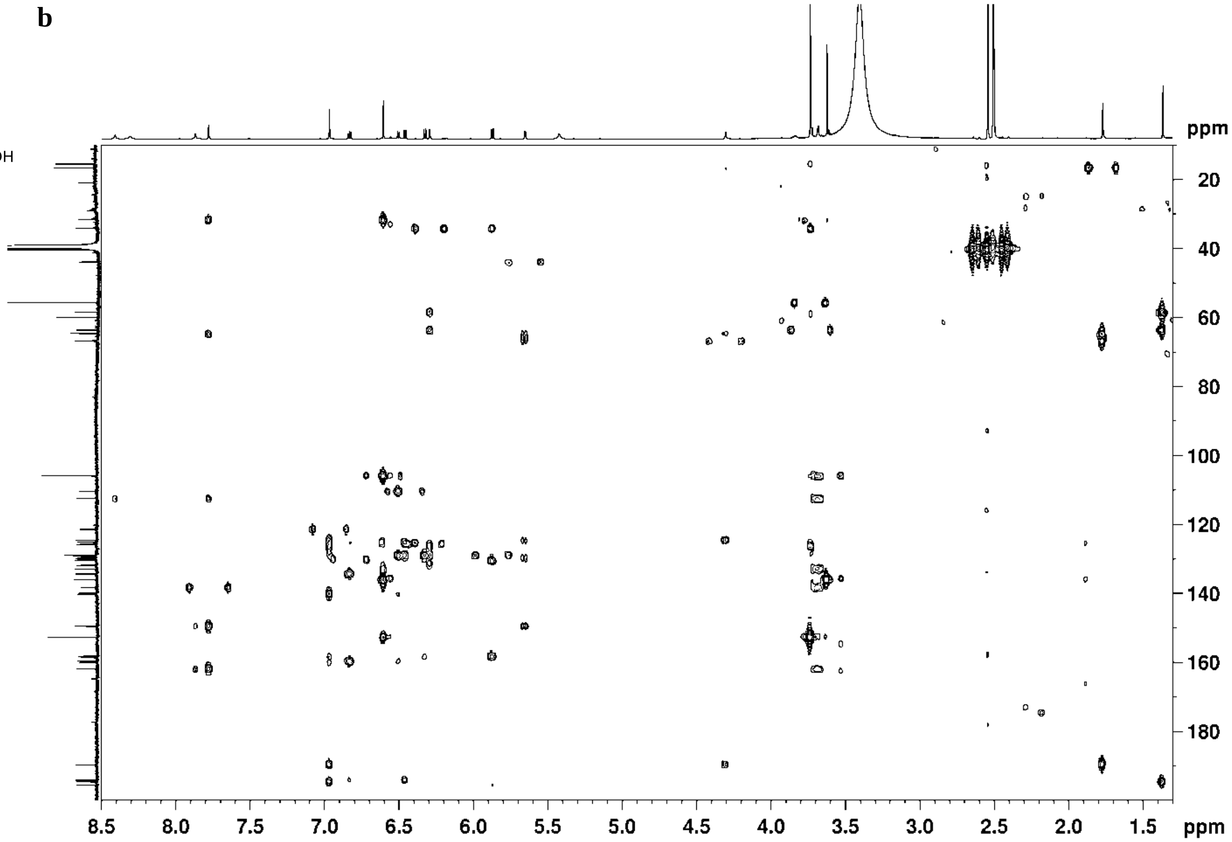

Supplementary Figure 141. The HMBC spectrum of compound **36** in DMSO-d_6. **a** Selected key HMBC correlations are indicated by the curved arrows. **b** The HMBC spectrum.

a

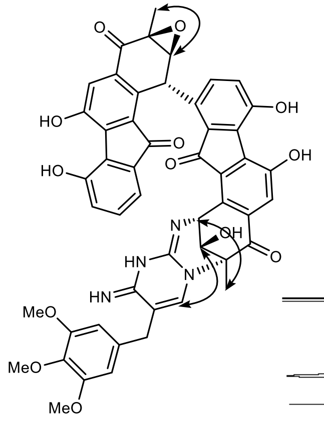

36

b

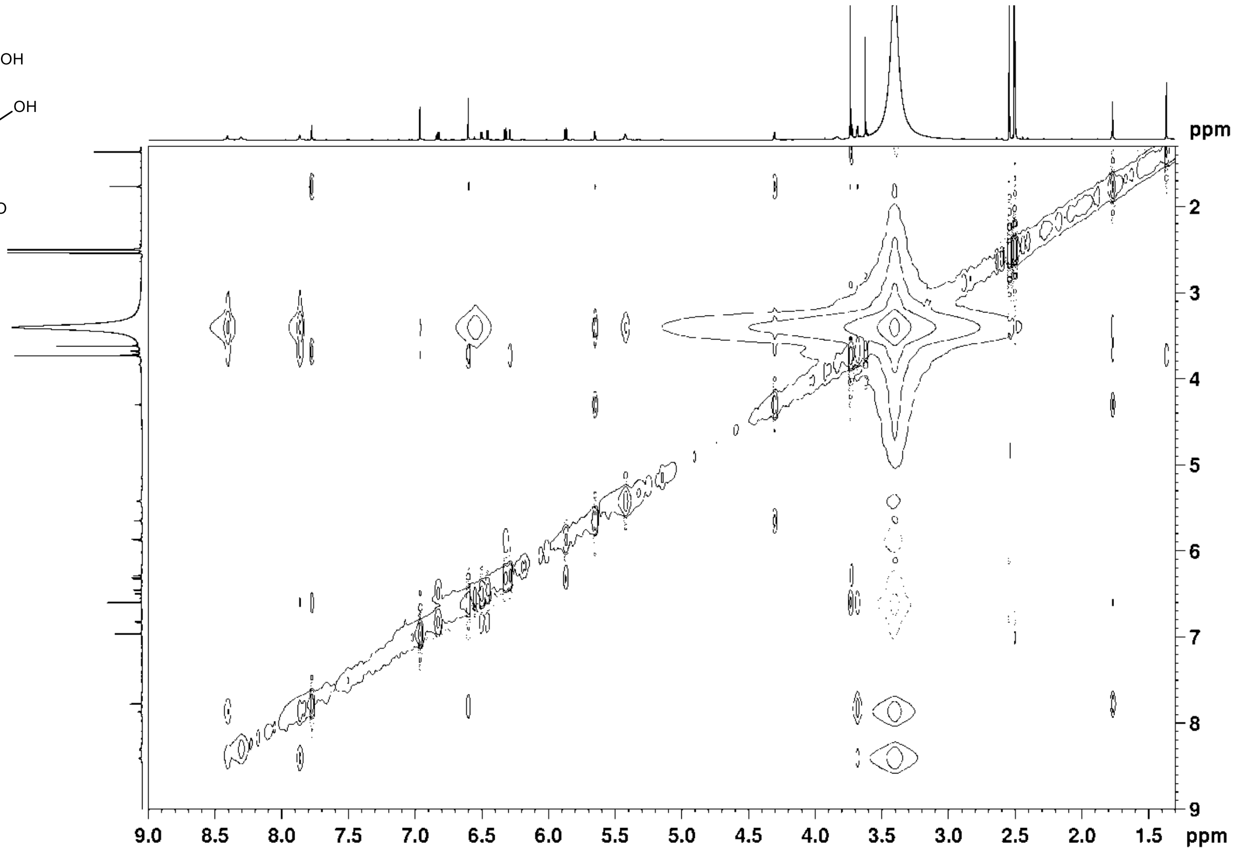

Supplementary Figure 142. The NOESY spectrum of compound **36** in DMSO-d_6. **a** Selected key NOESY correlations are indicated by the curved, double-headed arrows. **b** The NOESY spectrum.

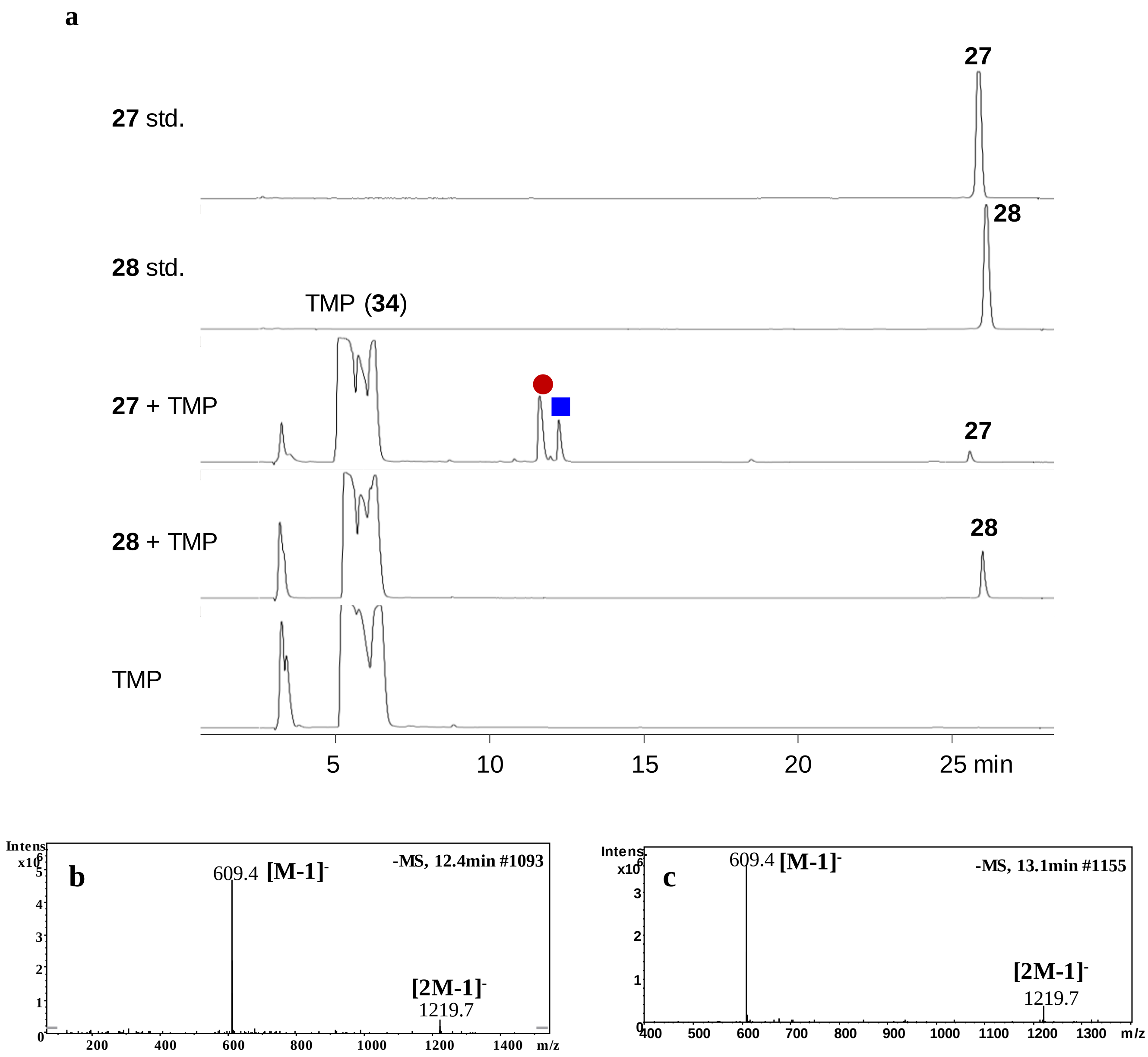

Supplementary Figure 143. HPLC traces of co-incubation of TMP (34) with 27 and 28. **a** HPLC analysis of the coupling reaction; **b** Negative mode ESI-MS data for the product with the symbol "●"; **c** Negative mode ESI-MS data for the product with the symbol "■".

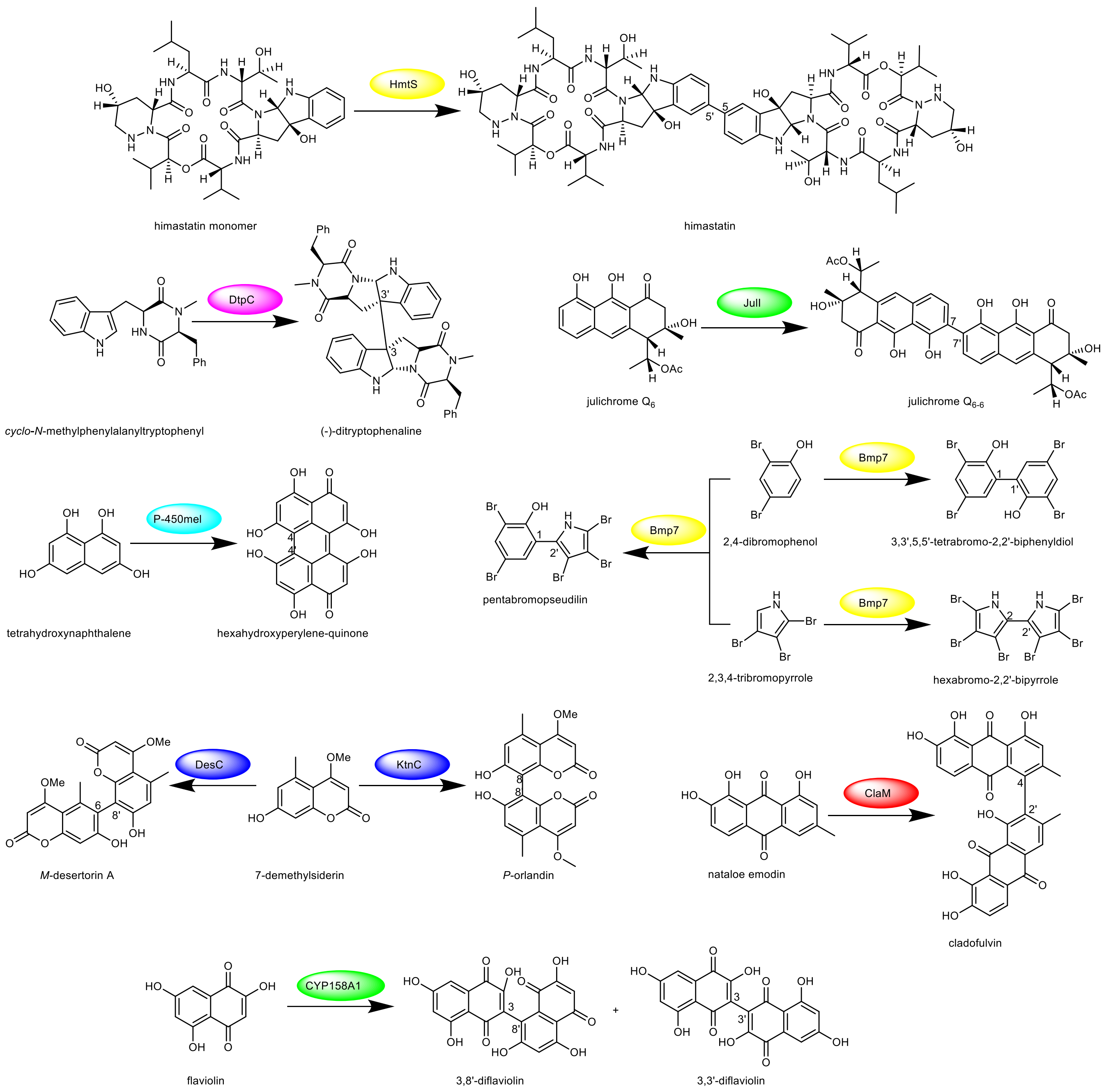

Supplementary Figure 144. The cytochrome P450 enzymes mediating radical-based dimerization to form the symmetric or asymmetric C−C bond.

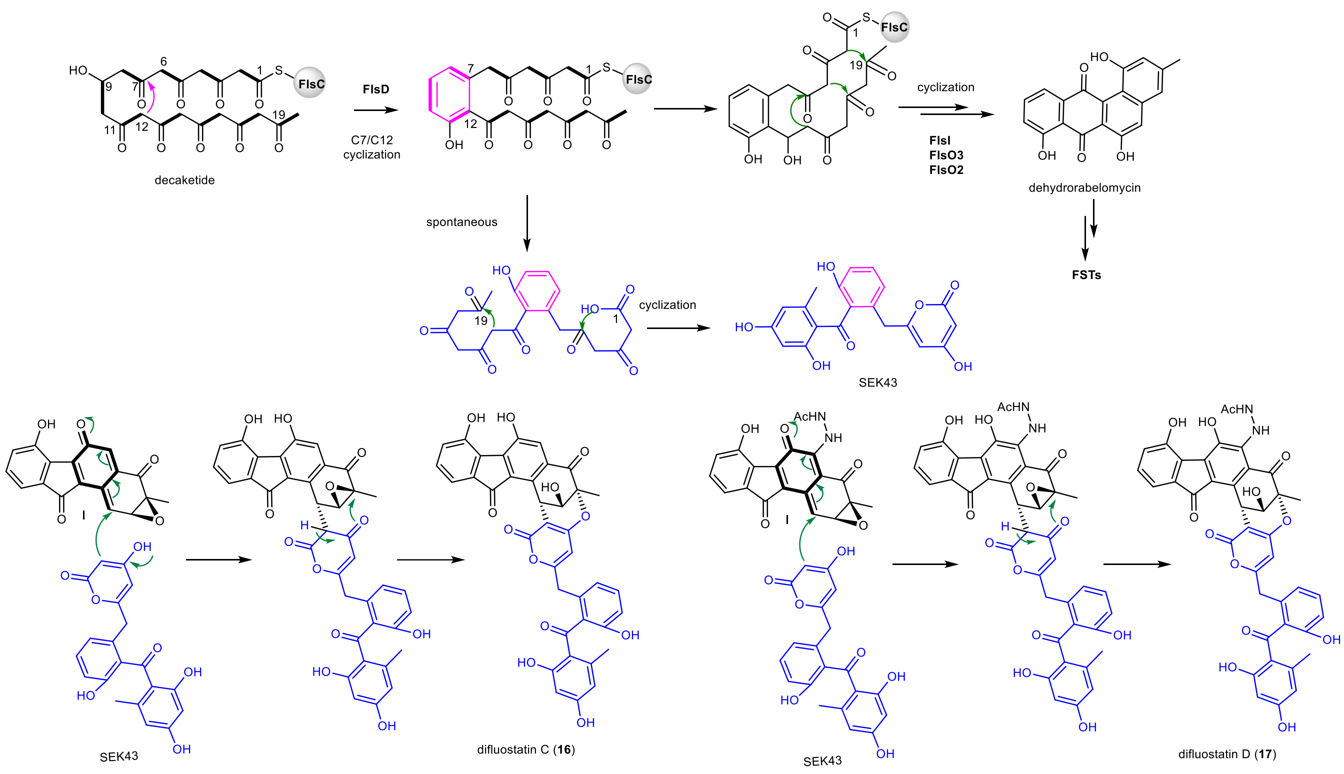

Supplementary Figure 145. Proposed the aberrant cyclization process to form SEK43 and reaction mechanisms leading to difluostatins C (**16**) and D (**17**).

Supplementary Tables

Supplementary Table 1. Strains and plasmids used and constructed in this study.

Strains/Plasmids	Characteristic(s)	Sources
E.coli		
BW25113	Host strain for PCR targeting	[1]
ET12567	Donor strain for conjugation	[2]
BL21(DE3)	Host strain for protein expression	Novagen
DH5α	Host strain for cloning	Invitrogen
Streptomyces and Micromonospora		
S. albus J1074	Host strain for heterologous expression	
S. coelicolor YF11	Host strain for heterologous expression	
S. lividans TK64	Host strain for heterologous expression	[3]
S. pactum SCSIO 02999 XM47i	Host strain for heterologous expression	
M. rosaria SCSIO N160	The producing strain of fluostatins	
FLS12	A mutant of *M. rosaria* SCSIO N160 where the *flsQ1* gene was inactivated	This study
**Plasmids**		
pUZ8002	Km^r, includeing *tra* for conjugation	[4]
pSET152	*OriT*RK2 (origin of conjugal transfer), *attP* (φC31 attachment	[5]
pSET152AB	Amp^r, *aac(3)IV*, *oriT* fragment, intφC31	[1]
pCSG5001	A cosmid of SuperCos1-based genomic library of strain SCSIO N160	
pCSG5003	A cosmid of SuperCos1-based genomic library of strain SCSIO N160	
pCSG5033	pCSG5003 derivative where Km^r gene was replaced by sequence from pSET152AB with *Bam*HI and *Eco*RI digesting	
pCSG5017	pCSG5001 derivative where *flsQ1* was replaced with *aac(3)IV* by insertional mutagenesis	This study
pCSG5209	A 771 bp *Nde*I/*Bam*HI fragment of *flsQ1* by PCR from genomic DNA of *M. rosaria* SCSIO N160 inserted into pET28a	This study
pCSG5213	A 780 bp *Nde*I/*Bam*HI fragment of *flsH* by PCR from genomic DNA of *M. rosaria* SCSIO N160 inserted into pET28a	This study
pCSG5225	A 957 bp *alp*1U fragment was synthesized, after digesting with *Nde*I/*Eco*RI and then was inserted into pET28a	This study
pCSG5226	A 774 bp *lom6* fragment was synthesized, after digesting with *Nde*I/*Eco*RI and then was inserted into pET28a	This study
pCSG5227	pCSG5213 containing *flsH* S92A via site-directed mutagenesis	This study
pCSG5228	pCSG5213 containing *flsH* E115A via site-directed mutagenesis	This study
pCSG5229	pCSG5213 containing *flsH* H241F via site-directed mutagenesis	This study

Supplementary Table 2. Primers used in this study.

Primers	Usage targeted	Sequences
**For screening cosmid library and confirmation the heterologous expression strains**		
flsFDTF		5’-CACGCAACGTCAGCACGCCC-3’
flsFDTR		5’-GGTGATGACGTCGTCGACCA-3’
flsGEF		5’-GAGGAGACATATGCCCAAGGTATCGCCG-3’
flsGER		5’-CAGGGGATCCTCAGCGCACCGGGTCGG-3’
flsSEF		5’-AGGTGCCCATATGATTCCCCGTTATACG-3’
flsSER		5’-GTCTGGATCCTCATCGCAGGGACTC-3’
ORF2EF		5’-AGGAGTACATATGACCCGGGCAATCCGC-3’
ORF2ER		5’-CGGGGGATCCTCAGCTCCTCGCCCA-3’
**For gene expression**		
FlsHEF	*flsH* expression	5’- GGAGAACCATATGCACAAGGTCGTTTCC -3’ (*Nde*I)
FlsHER		5’- GGGGGGATCCTCAGTCCTCCAGGAC -3’ (*Bam*HI)
FlsQ1EF	*flsQ1* expression	5’- CGGGAGACATATGACGCAATCAAACCTG -3’ (*Nde*I)
FlsQ1ER		5’- CGCCGGATCCTCAGCGCAGCTTCTC -3’ (*Bam*HI)
**For FlsH mutagenesis**		
S92AF	FlsH S92A	5’- GCGGTCTTCGGTG**GCG**CCAGCGGCGC -3’
S92AR	mutagenesis	5’- **CGC**CACCGAAGACCGCCGCCTCGCCG -3’
E115AF	FlsH E115A	5’- GCCTGGCGCTGCT**GGC**GCCGCCCTAC -3’
E115AR	mutagenesis	5’- **GCC**AGCAGCGCCAGGCGGGTGATCGG -3’
H241FF	FlsH H241F	5’- TGCCCGGGGTGTG**GTT**CCGGGTTCCG -3’
H241FR	mutagenesis	5’- **AAC**CACACCCCGGGCAGGGTCGTCAG -3’
**For *flsQ1* disruption and confirmation of mutants’ genotype**		
FlsQ1DF		5’-AACCTGCCCATCCTGGTCACCGGCGGTACCGGTACCCTCattccggggatccgtcgacc-3’
FlsQ1DR		5’-GGCCGTTTCAGGTGTGCCGCCGGACGGTCAGCGCAGCTTtgtaggctggagctgcttc-3’
FlsQ1DTF		5’-TGAGCCGGTGGTTCGGGGCG -3’
FlsQ1DTR		5’-AAGGGCCTGCAGGCCGGCCA -3’

Supplementary Table 3. ^{1}H and ^{13}C NMR data for compounds **12−14** in DMSO-d_6 (δ in ppm).

	**12**[a]		**13**[b]		**14**[b]	
no.	δ_C multi	δ_H multi (*J* in Hz)	δ_C multi	δ_H multi (*J* in Hz)	δ_C multi	δ_H multi (*J* in Hz)
1	114.9, CH	8.17, d (8.5)	67.4, CH	5.03, t (3.3)	44.6, CH	6.03, d (8.3)
2	131.6, CH	7.17, d (9.0)	75.8, CH	3.66, d (4.1)	61.7, CH	3.79, d (2.0)
3	119.1, C		75.4, C		58.5, C	
4	149.2, C		200.4, C		194.0, C	
4a	126.8, C		106.7†, C		130.7, C	
5	114.4, CH	7.79, s	143.1, C		121.6, CH	7.10, s
6	147.6, C		N		151.6†, C	
6a	134.6, C		128.2, C		137.8†, C	
6b	125.9, C		129.3, C		128.3†, C	
7	150.8, C		154.7, C		156.5†, C	
8	124.6, CH	6.98, d (7.5)	121.6, CH	6.68, d (8.0)	125.4, CH	6.71, d (7.4)
9	131.7, CH	7.20, dd (7.0, 7.0)	129.0, CH	6.99, dd (7.1, 8.0)	131.2, CH	7.00, dd (6.2, 7.4)
10	116.4, CH	7.08, d (7.0)	112.6, CH	6.85, d (7.1)	113.2†, CH	6.74, d (6.2)
10a	135.8, C		136.3, C		135.1, C	
11	194.7, C		192.2, C		192.5, C	
11a	128.7, C		119.6, C		131.9†, C	
11b	125.5, C		135.4, C		125.7†, C	
12	16.8, CH_3	2.28, s	23.6, CH_3	1.30, s	15.7, CH_3	1.47, s
15			160.0, C			
16			20.6, CH_3	1.94, s		
1'					152.1, C	
2'					111.9, CH	6.87, d (8.6)
3'					131.6, CH	7.72, d (8.8)
4'					118.1, C	
5'					131.6, CH	7.72, d (8.8)
6'					111.9, CH	6.87, d (8.6)
7'					167.9, C	
1-OH				5.66, brs		
1-NH						6.60, d (8.4)

[a] ^{1}H and ^{13}C NMR were recorded at 500 and 125 MHz, respectively; [b] ^{1}H and ^{13}C NMR were recorded at 700 and 176 MHz, respectively; †Chemical shift observed in HSQC or HMBC spectrum; N = no signal.

Supplementary Table 4. ^{1}H and ^{13}C NMR data for compounds **15−17** (δ in ppm).

	**15**[a,c]		**16**[b,d]		**17**[a,c]	
no.	δ_C multi	δ_H multi (*J* in Hz)	δ_C multi	δ_H multi (*J* in Hz)	δ_C multi	δ_H multi (*J* in Hz)
1	58.0, CH	5.37, d (3.4)	30.2, CH	5.57, d (3.2)	30.5, CH	5.43, d (3.9)
2	79.8, CH	5.19, d (3.4)	69.3, CH	4.32, brs	68.8, CH	3.92, t (4.1)
3	73.3, C		79.0, C		80.2, C	
4	193.6, C		190.6, C		190.4, C	
4a	132.7, C		133.4, C		108.3, C	
5	121.5, CH	7.31, s	121.9, CH	7.63 s	144.3, C	
6	154.5, C		148.7, C		149.1, C	
6a	137.6, C		134.2, C		127.9, C	
6b	127.0, C		125.4, C		129.4, C	
7	154.5, C		151.4, C		155.7, C	
8	125.1, CH	6.88, d (8.0)	123.4, CH	7.06, d (8.1)	121.4, CH	6.64, d (8.0)
9	131.7, CH	7.19, dd (7.0, 8.0)	131.7, CH	7.29, dd (7.0, 8.1)	129.5, CH	6.99, dd (7.2, 8.0)
10	115.4, CH	7.15, d (7.0)	116.3, CH	7.23, d (7.0)	112.6, CH	6.82, d (7.2)
10a	135.6, C		136.5, C		137.8, C	
11	194.1, C		191.0, C		191.0, C	
11a	138.0, C		132.1, C		120.6, C	
11b	127.5, C		137.1, C		135.3, C	
12	20.7, CH_3	1.65, s	18.4, CH_3	1.69, s	19.7, CH_3	1.55, s
15					160.3, C	
16					21.1, CH_3	1.93, s
1'	118.5, CH	7.58, d (9.9)	161.1, C		163.0[†], C	
2'	142.8, CH	6.20, d (9.9)	101.8, C		102.4, C	
3'	76.6, C		161.9, C		162.1, C	
4'	203.2, C		100.2, CH	5.66, s	100.4, CH	5.66, s
4a'	118.1, C					
5'	140.5, C		162.0, C		161.4, C	
6'	133.6, C		36.7, CH_2	3.59, brs	36.6, CH_2	3.49, dd (16.1, 16.1)
6a'	133.3, C					
6b'	123.9, C					
7'	153.3, C		132.6, C		133.1, C	
8'	124.7, CH	7.11, dd (1.0, 8.3)	122.1, CH	6.84, d (7.7)	121.3, CH	6.68, d (7.6)
9'	132.3, CH	7.26, dd (7.1, 8.3)	130.3, CH	7.21, dd (7.7, 8.2)	130.5, CH	7.17, dd (7.6, 8.1)
10'	115.6, CH	7.08, dd (1.0 7.1)	114.9, CH	6.86, d (8.2)	115.0, CH	6.76, d (8.1)
10a'	137.4, C					
11'	191.9, C		153.5, C		154.2, C	
11a'	120.3, C					
11b'	132.4, C					
12'	25.3, CH_3	1.20, s	131.0, C		131.3, C	
13'			200.5, C		200.4, C	
14'			115.4, C		116.1, C	
15'	168.0, C		167.1, C		165.6[†], C	
16'	21.2, CH_3	1.90, s	101.0, CH	6.24, brs	101.2, CH	6.12, d (2.3)
17'			163.7, C		163.7, C	
18'			112.0, CH	6.14, s	112.1, CH	6.07, d (2.0)
19'			144.1, C		143.5, C	
20'			21.4, CH_3	1.79, s	21.9, CH_3	1.79, s
2-OH						5.84, d (4.8)
3-OH		6.38, s				
6-OH						10.43, s
7-OH						15.65, s
13-NH						12.95, s
14-NH						14.86, s
3'-OH		5.48, s				
7'-OH		9.56, s				
11'-OH						9.78, s
14'-NH		9.34, s				
15'-OH						12.71, brs
17'-OH						10.43, s

[a] ^{1}H and ^{13}C NMR were recorded at 700 and 176 MHz, respectively; [b] ^{1}H and ^{13}C NMR were recorded at 500 and 125 MHz, respectively; [c] Measured in DMSO-d_6; [d] Measured in acetone-d_6; [†] Chemical shift observed in HMBC spectrum.

Supplementary Table 5. ^{1}H and ^{13}C NMR data for compound **18** (δ in ppm).

	**18**	
no.	δ_C multi	δ_H multi (J in Hz)
1	35.5, CH	5.94, d (4.1)
2	70.8, CH	4.74, d (4.1)
3	79.4, C	
4	193.3, C	
4a	133.2, C	
5	123.5, CH	7.77, s
6	150.2, C	
6a	135.6, C	
6b	126.5, C	
7	152.6, C	
8	125.0, CH	7.19, d (8.1)
9	132.7, CH	7.35, dd (7.0, 8.1)
10	117.2, CH	7.31, d (7.0)
10a	137.0, C	
11	193.8, C	
11a	134.9, C	
11b	138.5, C	
12	19.5, CH_3	1.95, s
1'	146.2, C	
2'	125.2, C	
3'	139.4, C	
4'	121.4, CH	7.25, s
4a'	143.2, C	
5'	148.7, C	
5a'	131.7, C	
6'	186.0, C	
6a'	116.5, C	
7'	164.0, C	
8'	120.7, CH	6.77, d (8.1)
9'	136.7, CH	7.45, dd (7.6, 8.1)
10'	117.3, CH	7.40, d (7.6)
10a'	135.0, C	
11'	150.7, C	
11a'	114.8, C	
11b'	122.2, C	
12'	22.2, CH_3	2.45, s
1"	129.1, C	
2"	142.1, CH	6.46, s
3"	76.2, C	
4"	202.0, C	
4a"	128.1, C	
5"	122.6, CH	7.70, s
6"	151.0, C	
6a"	134.3, C	
6b"	126.5, C	
7"	151.7, C	
8"	124.2, CH	7.02, d (8.1)
9"	132.1, CH	7.07, dd (7.2, 8.1)
10"	117.3, CH	6.97, d (7.2)
10a"	136.4, C	
11"	191.2, C	
11a"	132.4, C	
11b"	129.4, C	
12"	28.8, CH_3	1.65, s

^{1}H and ^{13}C NMR were recorded at 600 and 150 MHz, respectively; Measured in acetone-d_6.

Supplementary Table 6. Crystal data and structure refinement for difluostatin B (**15**)

Identification code	difluostatin B
Empirical formula	$C_{38}H_{26}N_2O_{11}$
Formula weight	686.61
Temperature/K	100.00 (11)
Crystal system	trigonal
Space group	$P3_121$
a/Å	23.9445(6)
b/Å	23.9445(6)
c/Å	14.8499(3)
α/°	90
β/°	90
γ/°	120
Volume/Å^3	7373.4(4)
Z	6
ρ_{calc}g/cm^3	0.928
μ/mm^{-1}	0.580
F(000)	2136.0
Crystal size/mm^3	0.1 × 0.03 × 0.03
Radiation	Cu Kα (λ = 1.54184)
2Θ range for data collection/°	7.322 to 124.564
Index ranges	$-23 \leq h \leq 19$, $-27 \leq k \leq 23$, $-15 \leq l \leq 17$
Reflections collected	24297
Independent reflections	7584 [R_{int} = 0.0353, R_{sigma} = 0.0389]
Data/restraints/parameters	7584/3/468
Goodness-of-fit on F^2	1.005
Final R indexes [I>=2σ (I)]	R_1 = 0.0601, wR_2 = 0.1737
Final R indexes [all data]	R_1 = 0.0878, wR_2 = 0.1953
Largest diff. peak/hole / e Å^{-3}	0.27/-0.18

Supplementary Table 7. ^{1}H and ^{13}C NMR data for compounds **24-26** (δ in ppm).

	**24**^{a,c}		**25**^{a,c}		**26**^{b,d}	
no.	δ_C multi	δ_H multi (J in Hz)	δ_C multi	δ_H multi (J in Hz)	δ_C multi	δ_H multi (J in Hz)
1	34.2, CH	6.60, d (1.4)	33.9, CH	6.52, d (1.9)	35.4, CH	6.78, overlapped
2	63.1, CH	3.99, d (2.0)	63.0, CH	3.96, d (2.1)	64.5, CH	3.97, d (2.1)
3	58.8, C		58.9, C		59.8, C	
4	193.8, C		193.8, C		195.0, C	
4a	133.2, C		133.2, C		133.6, C	
5	120.9, CH	7.57, s	120.9, CH	7.58, s	122.0, CH	7.62, s
6	149.9, C		149.6, C		151.9, C	
6a	135.4, C		135.2, C		136.9, C	
6b	125.4, C		125.3, C		127.3, C	
7	151.3, C		151.1, C		153.1, C	
8	124.1, CH	7.04, d (8.1)	124.1, CH	7.04, dd (0.6, 8.2)	124.8, CH	6.99, d (8.1)
9	131.9, CH	7.24, dd (7.1, 8.1)	132.0, CH	7.24, dd (7.3, 8.1)	132.2, CH	7.15, dd (7.1, 8.1)
10	116.1, CH	7.01, d (7.1)	116.0, CH	6.93, dd (0.8, 7.2)	116.4, CH	6.93, d (7.1)
10a	134.8, C		134.8, C		136.5, C	
11	191.5, C		191.1, C		192.8, C	
11a	131.6, C		N		132.2, C	
11b	130.7, C		130.7, C		131.9, C	
12	14.7, CH_3	1.40, s	14.8, CH_3	1.41, s	15.5, CH_3	1.46, s
1'	58.8, CH	6.04, brs	62.3, CH	7.00, d (2.0)	68.8, CH	6.08, d (2.6)
2'	62.3, CH	3.93, d (2.4)	59.5, CH	4.07, d (2.1)	61.0, CH	4.04, d (2.7)
3'	58.1, C		58.3, C		58.8, C	
4'	193.4, C		192.3, C		194.8, C	
4a'	N		N		133.9, C	
5'	120.4, CH	7.42, s	120.7, CH	7.49, s	122.2, CH	7.44, s
6'	150.6, C		151.9, C		154.0, C	
6a'	133.6, C		134.3, C		135.0, C	
6b'	126.5, C		126.3, C		128.4, C	
7'	150.4, C		150.6, C		153.0, C	
8'	124.3, CH	6.80, d (8.5)	124.5, CH	6.81, d (8.5)	125.3, CH	6.77, overlapped
9'	129.9, CH	6.14, d (8.5)	130.3, CH	6.16, d (8.6)	131.1, CH	6.21, d (8.7)
10'	132.0, C		131.9, C		134.7, C	
10a'	131.2, C		131.2, C		132.9, C	
11'	192.6, C		192.1, C		195.1, C	
11a'	130.7, C		131.9, C		N	
11b'	131.9, C		125.4, C		128.6, C	
12'	14.9, CH_3	1.55, s	14.5, C	1.57, s	15.2, CH_3	1.58, s
13'			175.1, C		57.8, CH_3	3.63, s
14'			40.1, CH	2.43, m		
15'			26.3, CH_2	1.42, overlapped<br>1.67, m		
16'			11.3, CH_3	0.84, t (7.4)		
17'			16.0, CH_3	1.11, d (6.9)		
1'-OH		5.92, brs				

[a] ^{1}H and ^{13}C NMR were recorded at 700 and 176 MHz, respectively; [b] ^{1}H and ^{13}C NMR were recorded at 500 and 125 MHz, respectively; [c] Measured in DMSO-d_6; [d] Measured in acetone-d_6; N = no signal.

Supplementary Table 8. ^{1}H and ^{13}C NMR data for compounds **27** and **28** in DMSO-d_6 (δ in ppm)

	**27**		**28**	
no.	δ_C multi	δ_H multi (J in Hz)	δ_C multi	δ_H multi (J in Hz)
1	62.3, CH	6.87, d (2.2)	62.3, CH	6.89, d (2.1)
2	59.2, CH	4.03, d (2.3)	59.2, CH	4.06, d (2.2)
3	58.3, C		58.3, C	
4	192.3, C		192.2, C	
4a	132.6†, C		131.7†, C	
5	121.7, CH	7.45, s	116.6, CH	7.73, s
6	152.0, C		152.4, C	
6a	132.0, C		136.8, C	
6b	127.8, C		123.8, C	
7	152.1, C		152.0, C	
8	120.3, CH	7.48, overlapped	125.3, CH	7.09, d (8.2)
9	132.3, CH	7.49, overlapped	132.7, CH	7.34, dd (7.1, 8.2)
10	117.9, CH	7.35, dd (2.0, 6.0)	116.6, CH	7.20, d (7.1)
10a	134.7, C		134.9, C	
11	191.3, C		191.2, C	
11a	132.8†, C		131.9†, C	
11b	125.4, C		127.2, C	
12	14.3, CH_3	1.54, s	14.4, CH_3	1.56, s
13	174.7, C		174.7, C	
14	39.9, CH	2.34, m	40.0, CH	2.34, m
15	26.0, CH_2	1.33, m	26.0, CH_2	1.33, m
		1.50, m		1.50, m
16	11.0, CH_3	0.77, t (7.4)	11.0, CH_3	0.77, t (7.5)
17	16.2, CH_3	1.03, d (6.9)	16.1, CH_3	1.02, d (6.9)
6-OCH_3			57.7, CH_3	4.17, s
7-OCH_3	57.6, CH_3	4.11, s		
6-OH		9.79, s		

^{1}H and ^{13}C NMR were recorded at 700 and 176 MHz, respectively; †Chemical shift can be exchanged.

Supplementary Table 9. ^{1}H and ^{13}C NMR data for compounds **29** and **30** in DMSO-d_6 (δ in ppm)

	**29**		**30**	
no.	δ_C multi	δ_H multi (J in Hz)	δ_C multi	δ_H multi (J in Hz)
1	58.9, CH	5.91, d (2.0)	59.0, CH	5.94, d (2.1)
2	62.2, CH	3.90, d (2.4)	62.1, CH	3.93, d (2.1)
3	58.1, C		58.2, C	
4	193.2, C		193.1, C	
4a	132.1†, C		131.5†, C	
5	121.5, CH	7.39, s	116.4, CH	7.67, s
6	151.0, C		151.4, C	
6a	132.3, C		136.4, C	
6b	127.8, C		123.8, C	
7	151.9, C		151.8, C	
8	120.3, CH	7.45, overlapped	125.0, CH	7.08, dd (0.5, 8.2)
9	132.3, CH	7.48, overlapped	132.7, CH	7.33, dd (7.2, 8.0)
10	117.9, CH	7.36, dd (1.4, 6.6)	116.4, CH	7.20, dd (0.5, 6.9)
10a	135.0, C		135.2, C	
11	191.7, C		191.7, C	
11a	132.2†, C		131.6†, C	
11b	131.6, C		133.9, C	
12	14.8, CH_3	1.53, s	14.9, CH_3	1.55, s
6-OCH_3			57.6, CH_3	4.15, s
7-OCH_3	57.5, CH_3	4.10, s		

^{1}H and ^{13}C NMR were recorded at 500 and 125 MHz, respectively; †Chemical shift can be exchanged.

Supplementary Table 10. ^{1}H and ^{13}C NMR data for compounds and **33**, **35**, and **36** in DMSO-d_6 (δ in ppm).

no.	**33**		**35**		**36**	
	δ_C multi	δ_H multi (*J* in Hz)	δ_C multi	δ_H multi (*J* in Hz)	δ_C multi	δ_H multi (*J* in Hz)
1	50.7, CH	5.01, dd (2.8, 6.7)	43.7, CH	5.55, d (3.9)	34.1, CH	6.29, d (1.8)
2	75.6, CH	4.13, d (2.8)	66.4, CH	4.27, d (3.9)	63.5, CH	3.73, d (2.2)
3	78.3, C		64.5, C		58.4, C	
4	193.5, C		189.3, C		194.5, C	
4a	131.7, C		129.5†, C		N	
5	120.0, CH	6.65, s	121.7, CH	6.92, s	121.2, CH	6.96, s
6	157.8, C		159.8, C		158.2, C	
6a	140.2, C		140.7, C		140.1, C	
6b	129.2, C		128.7, C		128.9, C	
7	159.8, C		159.2, C		159.5, C	
8	125.6, CH	6.52, d (7.9)	125.5, CH	6.58, dd (0.6, 8.1)	125.1, CH	6.50, d (8.0)
9	130.1, CH	6.90, dd (7.1, 8.1)	130.4, CH	6.95, dd (7.1, 8.2)	129.9, CH	6.82, dd (7.1, 8.0)
10	110.6, CH	6.66, d (7.2)	111.2, CH	6.71, dd (0.6, 7.0)	110.3, CH	6.45, dd (0.5, 7.0)
10a	134.4, C		134.4, C		134.3, C	
11	195.8, C		194.8, C		194.0, C	
11a	129.1, C		129.3†, C		131.8, C	
11b	128.5, C		125.0, C		125.8, C	
12	20.5, CH_3	1.49, s	16.3, CH_3	1.75, s	15.4, CH_3	1.36, s
1'	144.7, C				43.8, CH	5.65, d (3.8)
2'	137.1, C		149.4, C		66.8, CH	4.30, d (3.8)
3'	119.4, CH	6.40 d (8.3)			64.6, C	
4'	124.7, CH	6.46, d (8.3)	161.7, C		189.5, C	
4a'					129.8, C	
5'	128.9, C		112.4, C		121.5, CH	6.96, s
6'	122.9, CH	6.47, s	138.1, CH	7.80, s	160.0, C	
6a'					139.8, C	
6b'					129.0, C	
7'	19.8, CH_3	2.03, s	31.4, CH_2	3.66, brs	158.3, C	
8'			132.8, C		125.7, CH	6.32, d (8.5)
9'			105.8, CH	6.58, s	128.9, CH	5.87, d (8.5)
10'			152.6, C		129.1, C	
10a'					130.4, C	
11'			135.9, C		195.4, C	
11a'					129.5, C	
11b'					124.5, C	
12'			152.6, C		16.5, CH_3	1.76, s
13'			105.8, CH	6.58, s		
10'-OCH_3			55.6, CH_3	3.72, s		
11'-OCH_3			59.9, CH_3	3.61, s		
12'-OCH_3			55.6, CH_3	3.72, s		
2"					149.5, C	
4"					161.8, C	
5"					112.4, C	
6"					138.2, CH	7.77, s
7"					31.5, CH_2	3.68, brs
8"					132.8, C	
9"/13"					105.9, CH	6.60, s
10"/12"					152.6, C	
11"					135.9, C	
10"/12"-OCH_3					55.6, CH_3	3.73, s
11"-OCH_3					59.9, CH_3	3.62, s
1-NH		5.05, d (7.0)				
2-OH		5.69, brs				
3'-NH				7.85, s		
4'-NH				8.36, s		
3"-NH						7.86, s
4"-NH						8.40, s

^{1}H and ^{13}C NMR were recorded at 700 and 176 MHz, respectively; †Chemical shift can be exchanged; N = no signal.

Supplementary Table 11. Antimicrobial activities of FST analogues.

	MIC (μg mL^{-1})						
	S. aureus ATCC 29213	MRSA[a] ATCC 43300	*B. subtilis* SCSIO BS01	*M. Luteus* SCSIO ML01	*E. coli* ATCC 25922	*E. faecalis* ATCC 29212	*A. baumannii* ATCC 19606
**12**	64	64	32	64	>64	>64	>64
**14**	>64	>64	>64	>64	>64	>64	>64
**15**	>64	>64	>64	>64	>64	>64	>64
**16**	>64	>64	>64	>64	>64	>64	>64
**17**	64	64	64	>64	>64	>64	>64
**18**	>64	>64	>64	>64	>64	>64	>64
**24**	>64	>64	>64	>64	>64	>64	>64
**25**	>64	>64	64	>64	>64	>64	>64
**26**	>64	>64	64	>64	>64	>64	>64
**33**	>64	>64	>64	>64	>64	>64	>64
**35**	>64	16	16	>64	>64	>64	>64
**36**	>64	32	32	>64	>64	>64	>64
**Trimethoprim**	0.5	0.5	0.5	1	0.25	2	64

[a] methicillin resistant *S. aureus*

Supplementary Methods

General experimental procedures. Optical rotation was determined on a 341 polarimeter (Perkin Elmer, Inc.). UV spectrum was recorded with a U-2900 spectrophotometer (Hitachi). IR spectrum was obtained using a Nicolet*6700 FT-IR spectrometer (Thermo Scientific). ECD spectra was recorded on a Chirascan circular dichroism spectrometer (Applied Photophysics Co.). ^{1}H NMR, ^{13}C NMR, and 2D NMR spectra were recorded on either Bruker Avance 500, 600, or Bruker 700 spectrometer with tetramethylsilane (TMS) as the internal standard. Low-resolution mass spectrometric data were determined using an amaZon SL ion trap mass spectrometer. High-resolution electrospray ionization mass spectrometric (HRESIMS) data were measured on a MaXis 4G UHR-TOFMS spectrometer (Bruker Daltonics Inc.). Sephadex LH-20 (40–70 μm; Amersham Pharmacia Biotech AB, Uppsala, Sweden), and YMC*gel ODS-A (12 nm S-50 μm; Japan). TLC (0.1–0.2 or 0.3–0.4 mm) was conducted with precoated silica gel GF254 (10–40 nm, Yantai) glass plates. Column chromatography (CC) was performed with silica gel (100–200 mesh, Jiangyou Silica Gel Development, Inc., Yantai, P. R. China). Medium pressure liquid chromatography (MPLC) was performed on automatic flash chromatography (CHEETAHTM MP 200, Bonna-Agela Technologies Co., Ltd.) with the monitoring wavelength at 220 nm and the collecting wavelength at 254 nm. Semi-preparative HPLC was carried out on a Hitachi-L2130 HPLC (equipped with a Hitachi L-2455 diode array detector) or an Agilent 1260 Infinity series instrument (equipped with a quaternary pump, a vacuum degasser, an autosampler, a thermostatic column compartment, and a diode array detector). Preparative TLC was conducted with precoated glass plates (silica gel GF254, 10–40 nm).

Compounds isolation and structures elucidation. A total of 40 L fermentation cultures were prepared and separated to supernatants and mycelium by centrifugation. The mycelium was extracted 3 times with equal volume of acetone. After removal of the acetone by evaporation, the extracts of mycelium were merged into the supernatants. The combined broths were extracted 3 times with equal volume of butanone. The butanone extract was evaporated to dryness under reduced pressure to obtain the crude extract (30.0 g). The crude extract was subjected to silica gel (100–200 mesh) column chromatography by eluting with a gradient solvent system of chloroform/methanol (from 100:0 to 0:100, v/v) to afford seven fractions (Fr.1–Fr.7) on the basis of initial assessment by thin-layer chromatography (TLC). Fr.1 was subjected to Sephadex LH-20 column chromatography, eluting with $CHCl_3$/MeOH (1:1) to give six fractions (Fr.1.L1–Fr.1.L6). Subfraction Fr.1.L3 was further passed through C18 reversed phase MPLC (40 × 2.5 cm ID), eluting with a linear gradient of H_2O/CH_3CN (100:0 → 0:100, 15 mL min^{-1}, 200 min) to give six fractions Fr.1.L3.O1–Fr.1.L3.O6. Fr.1.L3.O2, Fr.1.L3.O3, Fr.1.L3.O4, and Fr.1.L3.O5 were subjected to Sephadex LH-20 column chromatography, respectively, eluting with $CHCl_3$/MeOH (1:1), further purified by preparative TLC (pTLC) and reversed-phase semi-preparative HPLC (H_2O/CH_3CN) to yield FST D (**7**, 35.0 mg), FST F (**5**, 12.8 mg), G/H (**9**, 32.0 mg), FST J (**8**, 65.0 mg), FST L (**10**, 27.0 mg), prekinamycin (**F2**, 10.8 mg), prefluostatin (**11**, 5.0 mg), rabelomycin (**F6**, 23.0 mg), and dehydrorabelomycin (**F7**, 56.0 mg). Subfraction Fr.1-L6 was chromatographed on the pTLC and purified by Sephadex LH-20 column ($CHCl_3$/MeOH, 1:1, v/v) to afford isoprefluostatin (**12**, 8.0 mg). Fr.4 was chromatographed on a MCI gel column (CH_3CN/H_2O, from 0:10 to 10:0) to get seven subfractions Fr.4.M1–Fr.4.M7. FST C (**4**, 43.0 mg) and FST K (**F1**, 26.0 mg) were obtained from Fr.4.M2, together with compound FST S (**14**, 4.0 mg). Difluostatin B (**15**, 7.3 mg), difluostatin C (**16**, 54.0 mg), and trifluostatin A (**18**, 2.8 mg) were yielded from Fr.4.M4. Pyrazolofluostatins A−C (**F3-F5**, detected but not isolated), FST R (**13**, 0.5 mg), and difluostatin D (**17**, 9.0 mg) were obtained from subfraction Fr.7.

The molecular formula of isoprefluostatin (**12**) was assigned as $C_{18}H_{12}O_4$ by HRESIMS (*m/z* 291.0670

[M - H]$^-$, calcd. for 291.0663, Supplementary Figure 3). The NMR data (Supplementary Table 3, Supplementary Figures 4–8) of **12** were very similar to those of prefluostatin (**11**).[6] A hydroxyl group was present at C1 in prefluostatin (**11**). However, a hydroxyl group was found to be located at C4 in **12**, which was supported by detailed 2D NMR analysis. The oxygenated carbon was assigned at C4 (δ_C 149.2) in **12** by the observed HMBC correlations from H_3-12 (δ_H 2.28) and H5 (δ_H 7.79) to C4 (Supplementary Figure 8). Therefore, the structure of compound **12** was determined and designated as isoprefluostatin.

Isoprefluostain (**12**). Red solid; ^{1}H and ^{13}C NMR: see Supplementary Table 3; IR: 3372, 2918, 1016, 667 cm^{-1}; UV/Vis: λ_{max} 456, 291, 262, and 220 nm; HRMS (m/z): (ESI/[M - H]$^-$) calcd. for $C_{18}H_{11}O_4$, 291.0663; found, 291.0670.

Fluostatin R (**13**) was isolated as a red crystal with the molecular formula $C_{20}H_{18}N_2O_8$ as established by HRESIMS (*m/z* 413.1008 [M - H]$^-$, calcd. for 413.0990, Supplementary Figure 9). Detailed analyses of 1D and 2D NMR spectra (Supplementary Table 3, Supplementary Figures 10–15) of **13** revealed that it was highly similar to those of pyrazolofluostatin A (**F3**, Supplementary Figure 2). However, a carbonyl was assigned at C4 in **13**. Also, an additional acetyl group was assigned at N14. The chemical shifts of the quaternary carbons at C5 (δ_C 143.1) and C15 (δ_C 160.0) in compound **13** (Supplementary Table 3, Supplementary Figure 11) and C5 (δ_C 144.3) and C15 (δ_C 160.3) in compound **17** (Supplementary Table 4, Supplementary Figure 39) match well with those of their counterparts in compound **15** (C5′ δ_C 140.5 and C15′ δ_C 168.0), Supplementary Table 4, Supplementary Figure 25). These data strongly support the presence of an acetylhydrazine ($CH_3CONHNH$-) moiety at C5 in compounds **13** and **17**. The small *J* value of H1/H2 ($^3J_{H1-H2}$ 4.1 Hz) assigned the *trans* configuration between H1 and H2 in **13**. Considering that **13** was a derivative of FST C (**4**), the absolute configurations of **13** were assigned as 1*R*, 2*S*, and 3*R*.

FST R (**13**). Red solid; ^{1}H and ^{13}C NMR: see Supplementary Table 3; HRMS (m/z): (ESI/[M - H]$^-$) calcd. for $C_{20}H_{17}N_2O_8$, 413.0990; found, 413.1008.

The molecular formula of fluostatin S (**14**) was assigned as $C_{25}H_{17}NO_7$ by HRESIMS (*m/z* 442.0932 [M - H]$^-$, calcd. for 442.0932, Supplementary Figure 16), requiring eighteen degrees of unsaturation. Careful analysis of NMR spectral data of **14** (Supplementary Table 3, Supplementary Figures 17–22) established the presence of subunit A (highly similar to fluostatin C, **4**) and subunit B (highly similar to *para*-aminobenzoic acid, PABA, **31**). HMBC correlations (Supplementary Figure 21) from H1 to C1′ and from NH to C1/C11b confirmed the location of the PABA moiety at C1. The small coupling constant ($^3J_{H1-H2}$, 2.0 Hz) between H1 and H2, and the NOESY correlation between H2 and NH of **14** indicated that the relative configurations of the chiral centers in **14** and fluostatin C (**4**) were identical. Consequently, the absolute configuration of **14** was assigned as 1*R*, 2*S*, and 3*S*.

FST S (**14**). Yellowish solid; ^{1}H and ^{13}C NMR: see Supplementary Table 3; optical rotation $[\alpha]_D^{25}$ – 40.5 (*c* 0.04, MeOH); IR: 3360, 2924, 1682, 1206 cm^{-1}; UV/Vis: λ_{max} 296 and 203 nm; HRMS (m/z): (ESI/[M - H]$^-$) calcd. for $C_{25}H_{16}NO_7$, 442.0932; found, 442.0932.

Difluostatin B (**15**). Structure elucidation was present in the main text. Red crystal; ^{1}H and ^{13}C NMR: see Supplementary Table 4; optical rotation $[\alpha]_D^{25}$ + 443.0 (*c* 0.04, MeOH); IR: 3360, 1682, 1248 cm^{-1}; UV/Vis: λ_{max} 324, 260, and 204 nm; HRMS (m/z): (ESI/[M - H]$^-$) calcd. for $C_{38}H_{25}N_2O_{11}$, 685.1464; found 685.1460.

Difluostatin C (**16**) was obtained as a red solid. The molecular formula of **16** was established to be $C_{38}H_{26}O_{12}$ by HRESIMS (*m/z* 673.1353 [M - H]$^-$, calcd. for 673.1351, Supplementary Figure 30), corresponding to 26 degrees of unsaturation. The ^{1}H-^{1}H COSY data of **16** (Supplementary Table 4, Supplementary Figure 34) showed two groups of characteristic aromatic ABC spin systems (δ_H 7.06/7.29/7.23 and δ_H 6.84/7.21/6.86), indicating that **16** was a heterodimer. Careful analysis of 2D

NMR data of **16** allowed the construction of the two subunits A and B, which accounted for 12 and 13 degrees of unsaturation, respectively. NMR data of subunits A and B were very similar to those of fluostatin B, and SEK43, respectively. The molecular formula of **16** still needed one more degree of unsaturation, indicating that the subunits A and B were linked by a ring. The connection of the two subunits through C1–C2′ was supported by the key HMBC correlations from H1 (δ_H 5.57) to C1′/C2′/C3′. The linkage of the two subunits through C3–O–C3′ was deduced by HMBC correlation from Me-12 (δ_H 1.69) to C3′ (δ_C 161.9) (Supplementary Figure 35). Thus the planar structure of **16** was established as a heterodimer coupled by a pyran ring. According to the coupling constant ($^3J_{H1-H2}$, 3.2 Hz) between H1 and H2 in **16**, the *trans* configuration was assigned. Considering the biosynthetic origin of **16**, the absolute configuration of **16** was tentatively assigned as 1*S*, 2*S*, and 3*R*.

Difluostatin C (**16**). Red solid; ^1H and ^{13}C NMR: see Supplementary Table 4; optical rotation $[\alpha]_D^{25}$ + 195.4 (*c* 0.16, MeOH); IR: 3242, 1682, 1582, 1275 cm^{-1}; UV/Vis: λ_{max} 448, 288, and 216 nm; ECD (*c* 2.97 × 10^{-4} M, MeCN), λ_{max} ($\Delta\varepsilon$): 204 (48.93) nm, 248 (8.46) nm, and 361 (−4.98) nm; HRMS (m/z): (ESI/$[M - H]^-$) calcd. for $C_{38}H_{25}O_{12}$, 673.1351; found, 673.1353.

The molecular formula of difluostatin D (**17**) was determined as $C_{40}H_{30}N_2O_{13}$ (*m/z* 745.1692 $[M - H]^-$, calcd. for 745.1675) by HRESIMS (Supplementary Figure 37). The ^1H and ^{13}C NMR spectral data of **17** (Supplementary Table 4, Supplementary Figures 38 and 39) and **16** were highly similar. The only difference was the presence of a $CH_3CONHNH$- fragment in **17**, which was confirmed by the COSY correlation (NHNH, δ_H 12.95/14.86), and the HMBC correlation from Me-16 (δ_H 1.93) to C15 (δ_C 160.3) (Supplementary Figures 41 and 42). It should be noted that the sp^2 methine (δ_H 7.63/δ_C 121.9) in **16** was not observed in **17**. The NOE correlations of NH-14/OH-6/OH-7 (Supplementary Figure 43) indicated that the $CH_3CONHNH$- fragment was located at C5 of **17**. Consistent with this assignment, changes in chemical shift of those carbons surrounding C5 were also observed, when compared to those corresponding data in **16** (Supplementary Table 4). Finally, the 1*S*, 2*S*, and 3*R* absolute configuration was tentatively assigned to **17** on the basis of the similar experimental ECD spectra of **17** and **16** (Supplementary Figure 37).

Difluostatin D (**17**). Red solid; ^1H and ^{13}C NMR: see Supplementary Table 4; optical rotation $[\alpha]_D^{25}$ + 40.0 (*c* 0.02, MeOH); IR: 2924, 1589, 1269 cm^{-1}; UV/Vis: λ_{max} 324, 296, and 204 nm; ECD (*c* 2.68 × 10^{-4} M, MeCN), λ_{max} ($\Delta\varepsilon$): 205 (40.86) nm, 244 (3.92) nm, 307 (16.25) nm, and 345 (−6.91) nm; HRMS (m/z): (ESI/$[M - H]^-$) calcd. for $C_{40}H_{29}N_2O_{13}$, 745.1675; found, 745.1692.

Trifluostatin A (**18**) was obtained as a red solid. The molecular formula of **18** was established to be $C_{54}H_{32}O_{14}$ by HRESIMS (*m/z* 903.1691 $[M - H]^-$, calcd. for 903.1791, Supplementary Figure 44), corresponding to 39 degrees of unsaturation. Detailed analyses of the 1D and 2D NMR spectra (Supplementary Table 5, Supplementary Figures 45−50) of **18** clearly revealed that it was an unusual trimer containing two typical fluostatin units (FST C-like unit A and FST K-like unit C) and one kinamycin-like unit B. The HMBC correlations (Supplementary Table 5, Supplementary Figure 49) from H1/H4′/Me-12′ to C2′ (δ_C 125.2) and from H1 to C1′ (δ_C 146.2) established the connection of units A and B through C1–C2′. The further linkage of units A and B via C3–O–C1′ to form a similar pyran ring as that in difluostatin C was deduced by comparing the corresponding chemical shifts surrounding the pyran ring (Tables S4 and S5). The linkage of units B and C via C5′–C1″ was determined on the basis of the HMBC correlations from H4′/H2″ to C5′ (δ_C 148.7). Thus, the planar structure of **18** was determined to be a trimer. Finally, the absolute configuration was tentatively assigned as shown above by considering that the NOESY correlations (Supplementary Figure 50) of H2/Me-12′/H2″ and its biosynthetic origin from fluostatin C (**4**).

Trifluostatin A (**18**). Red solid; ^1H and ^{13}C NMR: see Supplementary Table 5; HRMS (m/z): (ESI/[M - H]$^-$) calcd. for $C_{54}H_{31}O_{14}$, 903.1719; found, 903.1691.

The molecular formula of difluostatin F (**24**) was established to be $C_{36}H_{22}O_{11}$ by HRESIMS (*m*/*z* 629.1093 $[M - H]^-$, calcd. for 629.1089, Supplementary Figure 65). Careful analyses of the 1D and 2D NMR spectral data (Supplementary Table 7, Supplementary Figures 66−71) revealed that **24** was a C−C coupled dimer of FST C (**4**) through C1/C10′. This connection was supported by HMBC correlations from H1 to C9′/C10′/C10a′ and from H9′ to C1 (Supplementary Figure 70). Since **24** was a spontaneous derivative of fluostatin C (**4**), the absolute configuration of **24** was assigned as 1*R*, 2*S*, 3*S*, 1′*R*, 2′*S*, and 3′*S*.

The molecular formula of difluostatin G (**25**) was determined as $C_{41}H_{30}O_{12}$ by HRESIMS (*m*/*z* 713.1662 $[M - H]^-$, calcd. for 713.1665, Supplementary Figure 72). Careful analysis of the ^1H and ^{13}C NMR data of **25** (Supplementary Table 7, Supplementary Figures 73 and 74) indicated that it was also a dimer, and was highly similar to **24**. The difference was that a 2-methyl butyryl group was found to be located at C1′ in **25**. These assignments were supported by the COSY correlations (Supplementary Figure 76) of the spin system: H_3-17′/H14′/H_2-15′/H_3-16′ and the HMBC correlation (Supplementary Figure 77) from H1′ (δ_H 7.00) to C13′ (δ_C 175.1). In addition, the NOESY spectrum (Supplementary Figure 78) of **25** showed that the configurations of the chiral centers in the skeleton of **25** were identical to those of **24**. Accordingly, the absolute configuration of **25** was assigned as 1*R*, 2*S*, 3*S*, 1′*R*, 2′*S*, and 3′*S*.

Difluostatin H (**26**) was isolated as a brick red solid with the molecular formula of $C_{37}H_{24}O_{11}$, which was determined by HRESIMS (*m*/*z* 643.1239 $[M - H]^-$, calcd. for 643.1246, Supplementary Figure 79). The 1D and 2D NMR data (Supplementary Table 7, Supplementary Figures 80−85) suggested that **26** was also a dimer that contains two monomeric FST C-like units A and B. The connection of units A and B via C1/C10′ was deduced by HMBC correlations from H1 to C9′/C10′/C10a′ and from H9′ to C1 (Supplementary Figures 84). The oxygenated methyl was located at C1′ in **26** by the HMBC correlations (Supplementary Figure 84) from H1′ to C13′ and from H_3-13′ to C1′. The NOESY spectra of **26** were closely similar to those of FST C (**4**). Therefore, the absolute configuration of **26** was determined to be 1*R*, 2*S*, 3*S*, 1′*R*, 2′*S*, and 3′*S*.

Compound **27** (7-*O*-methyl-FST J) was isolated as a yellow solid. The molecular formula of **27** was determined as $C_{24}H_{22}O_7$ by HRESIMS (*m*/*z* 421.1299 $[M - H]^-$, calcd. for 421.1293, Supplementary Figure 87). ^1H-NMR and ^{13}C-NMR spectra (Supplementary Table 8, Supplementary Figures 88 and 89) of **27** were similar to those of FST J (**8**). The only difference was the presence of a 7-*O*-methyl group in **27**. This assignment was confirmed by the HMBC correlation from oxygenated methyl 7-OCH_3 (δ_H 4.11) to C7.

The molecular formula of **28** (6-*O*-methyl-FST J) was established to be the same as that of **27** by HRESIMS (*m*/*z* 421.1298 $[M - H]^-$, calcd. for 421.1293, Supplementary Figure 94). The 1D and 2D NMR spectra (Supplementary Table 8, Supplementary Figures 95−100) of **28** showed a high similarity to those of FST J. The only difference was the presence of a 6-*O*-methyl group in **28**. This assignment was supported by the HMBC correlation from oxygenated methyl 6-OCH_3 (δ_H 4.17) to C6. Consequently, the structure of **28** was determined as 6-*O*-methyl-FST J.

Compound **29** (7-*O*-methyl-FST C) was obtained as a yellow solid. Its molecular formula was determined as $C_{19}H_{14}O_6$ by HRESIMS (*m*/*z* 337.0722 $[M - H]^-$, calcd. for 337.0718, Supplementary Figure 101). The ^1H and ^{13}C NMR spectra (Supplementary Table 9, Supplementary Figures 102 and 103) of **29** were highly similar to those of FST C. The only difference was the presence of a 7-*O*-methyl group in **29**. This assignment was confirmed by the key HMBC correlation (Supplementary Figure 106) from the oxygenated methyl 7-OCH_3 (δ_H 4.10) to C7 (δ_C 151.9). Thus, the structure of **29** was established to be 7-*O*-methyl-FST C.

Compound **30** (6-*O*-methyl-FST C) has the molecular formula of $C_{19}H_{14}O_6$ as established by HRESIMS (*m/z* 337.0713 $[M - H]^-$, calcd. for 337.0718, Supplementary Figure 108). Detailed analyses of 1D and 2D NMR spectra (Supplementary Table 9, Supplementary Figures 109–114) of **30** showed a strikingly similarity to those of fluostatin C (**4**). The only difference was the presence of a 6-*O*-methyl group in **30**. The HMBC correlation from oxygenated methyl 6-OCH_3 (δ_H 4.15) to C6 (δ_C 151.4) supported this assignment. Therefore, the structure of **30** was determined as 6-*O*-methyl-FST C.

The molecular formula of **33** was determined as $C_{25}H_{19}NO_6$ by HRESIMS (*m/z* 428.1148 $[M - H]^-$, calcd. for 428.1140, Supplementary Figure 121). Careful analysis of 1D and 2D NMR spectra of **33** (Supplementary Table 10, Supplementary Figures 122–127) confirmed the presence of a FST C-like moiety and a 2-amino-5-methylphenol moiety. The connection of these two moieties via C1–N–C2′ and C3–O–C1′ were confirmed by HMBC correlations (Supplementary Figure 126) from H1 (δ_H 5.01) to C2′ (δ_C 137.1), from NH (δ_H 5.05) to C1/C2/C1′/C2′/C3′, and from H_3-12 (δ_H 1.49) to C1′ (δ_C 144.7). The *trans* configuration between H1 and H2 in **33** was assigned by the small coupling constant ($^3J_{H1-H2}$, 2.8 Hz). This was supported by the NOESY correlation of H2 (δ_H 4.13) and NH (δ_H 5.05). Finally, the absolute configuration of **33** was assigned as 1*R*, 2*S*, and 3*R* given that it was a chemical derivative of FST D (**7**).

Compound **35** was obtained as a red solid with the molecular formula $C_{32}H_{28}N_4O_8$ established by HRESIMS (*m/z* 595.1833 $[M - H]^-$, calcd. for 595.1834, Supplementary Figure 129). Carful analysis of the NMR spectra of **35** (Supplementary Table 10, Supplementary Figures 130–135) allowed the establishment of the presence of a FST C-like uint A and a rearranged trimethoprim-like unit B. The two units were connected through C1–N–C2′ and C3–N1′ were confirmed by HMBC correlations from H1/NH-3′ to C2′ (δ_C 149.4), from H6′ (δ_H 7.80) to C3 (δ_C 64.5), and from H_3-12 (δ_H 1.75) to C6′ (δ_C 138.1). Thus, the planar structure of **35** was determined. The NOESY correlations (Supplementary Figure 135) of H1/12-CH_3 and H2/H6′ deduced the stereo-configuration. The absolute configuration of **35** was presumed to be 1*R*, 2*S*, and 3*R* given that it was a chemical derivative of FST D (**7**).

The molecular formula $C_{50}H_{38}N_4O_{13}$ of **36** was determined by HRESIMS (*m/z* 901.2354 $[M - H]^-$, calcd. for 901.2363, Supplementary Figure 136). Extensive analysis of ^1H and ^{13}C NMR of **36** (Supplementary Table 10, Supplementary Figures 137 and 138) indicated the presence of a FST C (**4**)-like unit and a **35**-like unit. The connection of the two units via C1–C10′ was deduced by the HMBC correlations (Supplementary Figure 141) from H1 (δ_H 6.29) to C9′/C10′/C10a′ and from H9′ (δ_H 5.87) to C1 (δ_C 34.1). Therefore, the planar structure of **36** was established. Finally, the absolute configurations for **36** were assigned as 1*R*, 2*S*, 3*S*, 1′*R*, 2′*S*, and 3′*R* given that it was a chemical derivative of FST D (**7**).

Supplementary References

1. Datsenko KA, Wanner BL. One-step inactivation of chromosomal genes in *Escherichia coli* K-12 using PCR products. *Proc. Natl. Acad. Sci. USA* **97**, 6640-6645 (2000)
2. Macneil DJ, Gewain KM, Ruby CL, Dezeny G, Gibbons PH, Macneil T. Analysis of *Streptomyces avermitilis* genes required for avermectin biosynthesis utilizing a novel integration vector. *Gene* **111**, 61-68 (1992)
3. Zhang G, *et al.* Characterization of the amicetin biosynthesis gene cluster from *Streptomyces vinaceusdrappus* NRRL 2363 implicates two alternative strategies for amide bond formation. *Appl. Environ. Micro.* **78**, 2393-2401 (2012)
4. Paget MSB, Chamberlin L, Atrih A, Foster SJ, Buttner MJ. Evidence that the extracytoplasmic function sigma factor ς^E is required for normal cell wall structure in *Streptomyces coelicolor* A3(2). *J. Bacteriol.* **181**, 204-211 (1999)
5. Bierman M, Logan R, Obrien K, Seno ET, Rao RN, Schoner BE. Plasmid cloning vectors for the conjugal transfer of DNA from *Escherichia coli* to *Streptomyces* spp. *Gene* **116**, 43-49 (1992)
6. Yao CBF, Schiebel M, Helmke E, Anke H, Laatsch H. Prefluostatin and new urauchimycin derivatives produced by *Streptomycete* isolates. *Z. Naturforsch. B.* **61**, 320-325 (2006)
